# Design, synthesis, and antiprotozoal evaluation of new 2,4-bis[(substituted-aminomethyl)phenyl]quinoline, 1,3-bis[(substituted-aminomethyl)phenyl]isoquinoline and 2,4-bis[(substituted-aminomethyl)phenyl]quinazoline derivatives

**DOI:** 10.1080/14756366.2019.1706502

**Published:** 2020-01-03

**Authors:** Jean Guillon, Anita Cohen, Clotilde Boudot, Alessandra Valle, Vittoria Milano, Rabindra Nath Das, Aurore Guédin, Stéphane Moreau, Luisa Ronga, Solène Savrimoutou, Maxime Demourgues, Elodie Reviriego, Sandra Rubio, Sandie Ferriez, Patrice Agnamey, Cécile Pauc, Serge Moukha, Pascale Dozolme, Sophie Da Nascimento, Pierre Laumaillé, Anne Bouchut, Nadine Azas, Jean-Louis Mergny, Catherine Mullié, Pascal Sonnet, Bertrand Courtioux

**Affiliations:** a INSERM U1212, UMR CNRS 5320, ARNA Laboratory, UFR des Sciences Pharmaceutiques, Université de Bordeaux, Bordeaux, France; b IRD, AP-HM, SSA, VITROME, Aix-Marseille University, Marseille, France; c INSERM U1094, Tropical Neuroepidemiology, Institute of Neuroepidemiology and Tropical Neurology, Université de Limoges, Limoges, France; d PREM UMR5254 – UPPA/CNRS, Technopole Hélioparc, Université de Pau, Pau, France; e UFR de Pharmacie, AGIR (Agents Infectieux, Résistance et chimiothérapie), Université de Picardie Jules Verne, Amiens,France; f Université de Bordeaux, Laboratoire de Toxicologie et d'Hygiène Appliquée - INRA, UFR des Sciences Pharmaceutiques, Bordeaux, France; g Institut Curie, Université Paris-Saclay, CNRS-UMR 9187, INSERM U1196, Université Paris-Saclay, Orsay, France; h Institute of Biophysics of the CAS, Brno, Czech Republic

**Keywords:** Antimalarial activity, quinoline-like derivatives, antitrypanosomal activity, antileishmanial activity, G-quadruplex

## Abstract

A series of new 2,4-bis[(substituted-aminomethyl)phenyl]quinoline, 1,3-bis[(substituted-aminomethyl)phenyl]isoquinoline, and 2,4-bis[(substituted-aminomethyl)phenyl]quinazoline derivatives was designed, synthesised, and evaluated *in vitro* against three protozoan parasites (*Plasmodium falciparum*, *Leishmania donovani*, and *Trypanosoma brucei brucei*). Biological results showed antiprotozoal activity with IC_50_ values in the µM range. In addition, the *in vitro* cytotoxicity of these original molecules was assessed with human HepG2 cells. The quinoline **1c** was identified as the most potent antimalarial candidate with a ratio of cytotoxic to antiparasitic activities of 97 against the *P. falciparum* CQ-sensitive strain 3D7. The quinazoline **3h** was also identified as the most potent trypanosomal candidate with a selectivity index (SI) of 43 on *T. brucei brucei* strain. Moreover, as the telomeres of the parasites *P. falciparum* and *Trypanosoma* are possible targets of this kind of nitrogen heterocyclic compounds, we have also investigated stabilisation of the *Plasmodium* and *Trypanosoma* telomeric G-quadruplexes by our best compounds through FRET melting assays.

## Graphical Abstract

**Figure:**
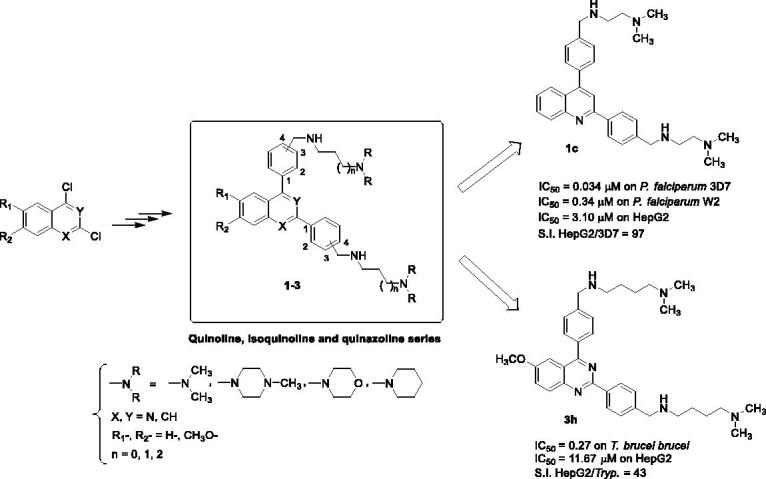

## Introduction

According to WHO, malaria remains a major public health problem, all the more worrying nowadays as epidemiological data show that no significant progress in reducing malaria cases was registered for the period 2015–2017. Indeed, in 2017, an estimated 219 million cases of malaria occurred worldwide compared with 239 million cases in 2010 and 217 million cases in 2015^1^. Fortunately, progress in reducing mortality from malaria occurs, since in 2017, indeed 435 000 estimated deaths were globally recorded from malaria, compared with 451,000 estimated deaths in 2016 and 607,000 in 2010^1^. In this context, even if artemisinin and artemisinin-based combination therapies (ACTs) represent the most effective antimalarial drugs, a resistance of *Plasmodium falciparum* to artemisinin has been first observed on the Cambodia–Thailand border in 2009^2^^,^^3^, then spread in the Greater Mekong Subregion^1^. Recent studies have demonstrated that the mechanisms of resistance developed by the parasite against artemisinin affect only one stage of the malaria parasite cycle in humans, the ring stage, resulting in a “partial resistance,” that includes information on the genotype, since in 2013, the identification of the *Pf*Kelch13 (K13) mutations has been defined to be associated with reduced susceptibility to artemisinin^4^^,^^5^. ACTs composed of artemisinin or its derivatives and various partner drugs (mefloquine (MQ), lumefantrine, amodiaquine, sulfadoxine-pyrimethamine, or piperaquine) are then recommended for 3 d^6^. These ACTs are also recommended as the first-line treatment for uncomplicated malaria, caused by all *Plasmodium* species, except for the first trimester of pregnancy.

The increase in global drug resistance in the malaria-endemic areas has significantly reduced the potency of most current used antimalarial compounds. In order to solve this problem, the development of new antimalarial drug candidates with novel potential mechanisms of action is urgently needed^7–10^. Efforts to discover new 4-aminoquinoline derivatives are ongoing. In fact, it is unlikely that the parasite will be able to evolve resistance to drugs targeting the pathway involved in haemoglobin degradation. Previous studies have shown that modification and modulation of the lateral side chain of chloroquine (CQ) that led to original aminoquinoline compounds avoid the CQ resistance mechanism^11–14^.

**Figure 1.**
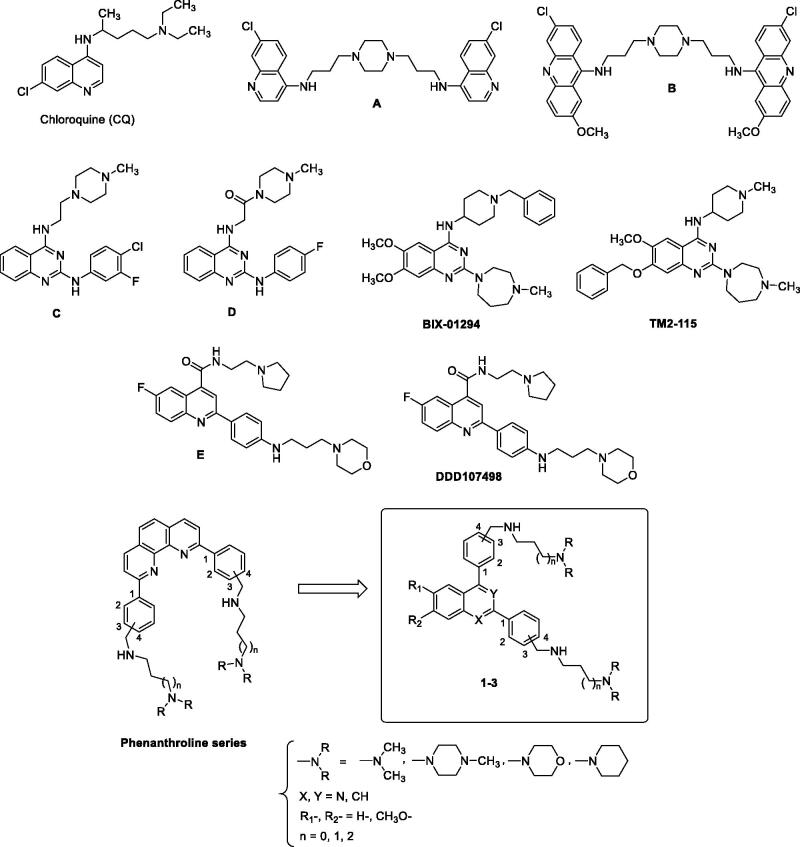
The structures of chloroquine (CQ), bisquinoline A, bisacridine B, 2-anilino-4-amino- quinazolines C, D, diamino-quinazolines BIX-01294 and TM2-115, and quinolines E and DDD107498, and newly synthesised 2,4-bis[(substituted-aminomethyl)phenyl]quinoline, 1,3-bis[(substituted-aminomethyl)phenyl]isoquinoline and 2,4-bis[(substituted-aminomethyl)phenyl]quinazoline derivatives **1–3**.

Another strategy is the design and the synthesis of new quinoline-based drugs that could not be recognised by the protein system involved in the drug efflux. By following this strategy, two original series of bisquinoline and bisacridine antiplasmodium drugs were designed and prepared (Figure 1(A,B))^15–18^. These new derivatives had much lower resistance indices than CQ, indicating that these original heterocyclic pharmacophores are less efficiently excluded by drug-resistant parasites. Recently, high throughput screens (HTS) followed by the design and synthesis of new structures revealed several analogues possessing the 2-anilino quinazoline scaffold, such as the disubstituted quinazolines **C-D**, **BIX-01294,** and **TM2-115** (Figure 1). In addition, the quinoline-4-carboxamide series was also identified from a screen against the *P. falciparum* 3D7 strain leading to the discovery of quinolines **E** and **DDD107498** (Figure 1)^19–23^.

**Figure 2.**
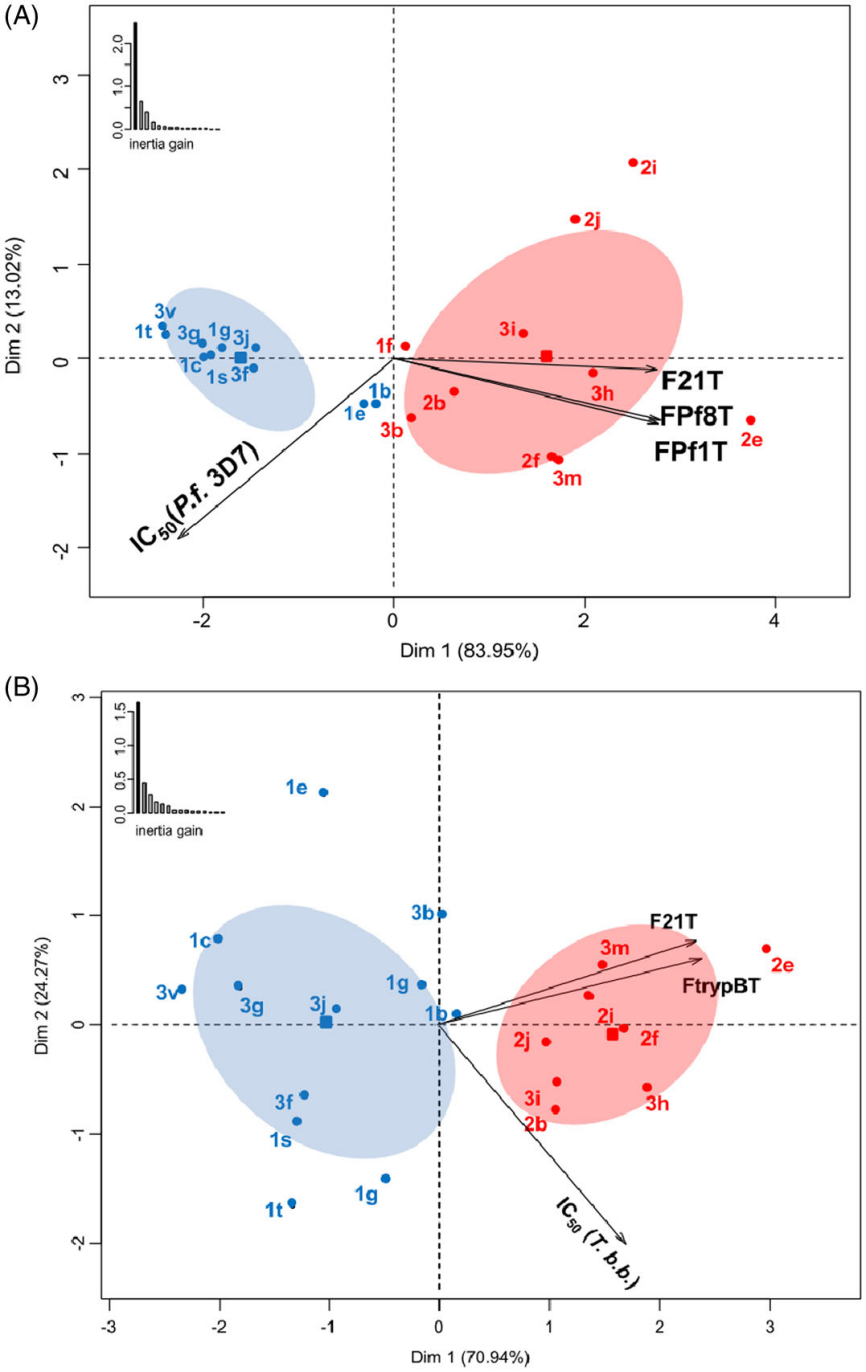
Principal Component Analysis biplots employed in Hierarchical Ascendant Classification (HAC) in relation to *P. falciparum* (A) and *T. brucei brucei* variables (B). Colours and confidence ellipses (for *α* = 0.05) define the attribution of the selected twenty ligands **1–3** based on their IC_50_ best results to the two groups defined by HAC.

Among other vector-borne parasitic diseases, those caused by parasites of the *Trypanosomatidae* family are also public health problems. Indeed, leishmaniases, caused by parasites of the *Leishmania* genus which are transmitted by the bite of infected female phlebotomine sandflies, are among the most neglected parasitic diseases in the world. An estimated 0.7–1 million new cases of leishmaniases per year are reported from nearly 100 endemic countries^24^. There are three main clinical forms of leishmaniases: cutaneous (the most common), mucocutaneous and visceral, also known as kala-azar and the most serious form of the disease since this is fatal if left untreated in over 95% of cases. Humans are the main reservoir for visceral leishmaniasis (VL) due to *Leishmania donovani*. Nevertheless, *Leishmania infantum* may also cause VL, but the domestic dog is the primary reservoir of this *Leishmania* species, although other mammalian reservoirs exist and sporadic non-vector transmission routes such as direct transmission between drug users co-infected with HIV through sharing needles^25^. VL is mainly characterised by irregular fever, enlargement of the spleen and liver, and anaemia. Most cases occur in Brazil, East Africa, and South-East Asia. An estimated 50,000–90,000 new cases of VL occur worldwide each year, less than half of which are reported to WHO^2^. In 2017, more than 95% of new cases reported to WHO occurred in only 10 countries: Bangladesh, Brazil, China, Ethiopia, India, Kenya, Nepal, Somalia, South Sudan, and Sudan. A limited number of drugs (meglumine antimoniate, sodium stibogluconate, pentamidine, amphotericin B, and miltefosine), all of which have high toxicities, resistances, and costs^24^^,^^26^^,^^27^, can be used to treat leishmaniases, and although efforts have been made by WHO, non-governmental organisations, and manufacturers to improve access to medicines, leishmaniases persist as poverty-related diseases.

Furthermore, another neglected disease caused by *Trypanosomatidae* parasites of the *Trypanosoma* genus is the human African trypanosomiasis (HAT), or sleeping sickness, almost invariably fatal unless treated. This infection is transmitted to humans through the bite of an infected tsetse fly. Brain involvement causes various neurological disturbances, including sleep disorders, progression to coma and, ultimately, death. There are two clinical forms: the slowly progressing form (gambiense HAT), caused by infection with *Trypanosoma brucei gambiense* (currently 98% of cases), and the faster progressing form (rhodesiense HAT), caused by infection with *Trypanosoma brucei rhodesiense*. As a neglected tropical disease targeted by the WHO for elimination, a historically low number of cases (<1000) was reported in 2018. The recent approval of a new medicine (fexinidazole) for the treatment of gambiense HAT has opened new possibilities for the management of cases and thus led to recent WHO interim guidelines for this treatment^27^. A veterinary form of this parasitic disease exists. Named Nagana, it is caused by *Trypanosoma brucei brucei* which contaminates African livestock, thus having a significant economic impact.

In the course of our work devoted to discovery of new heterocyclic compounds for use in antiprotozoal chemotherapy^28–34^, we previously prepared a series of substituted 2,9-bis[(substituted-aminomethyl)phenyl]-1,10-phenanthroline derivatives (Figure 1) designed as antimalarial candidates that could bind to *P. falciparum* DNA G-quadruplexes^35^. By taking into account our experience in the field of the synthesis of new antiprotozoal heterocyclic compounds, we describe here the design and synthesis of new 2,4-bis[(substituted-aminomethyl)phenyl]quinoline, 1,3-bis[(substituted-aminomethyl)phenyl]isoquinoline and 2,4-bis[(substituted-aminomethyl)phenyl]quinazoline derivatives **1–3** (Figure 1) that could be considered as new bioisoster analogues of our previously described phenanthroline compounds. We report on their *in vitro* antiplasmodial activity against the CQ -sensitive (3D7) and the CQ -resistant (W2) strains of the malaria parasite *P. falciparum*. As aza heterocyclic scaffolds are the fundamental units of many antiprotozoal candidates, these quinoline-like derivatives were also tested for *in vitro* efficacy against medically important protozoans *L. donovani* and *T. brucei brucei*.

In addition, the *in vitro* cytotoxicity of our new bis[(substituted-aminomethyl)phenyl]quinoline-like derivatives was assessed in human HepG2 cells, and an index of selectivity, the ratio of cytotoxic to antiparasitic activity, was determined for each derivative. The telomeres of the different protozoa could constitute attractive drug targets^36–39^ and telomerase activity is detected in gametocytes and during the transition to the erythrocytic stage of *P. falciparum*^40^. The telomeric 3′G-overhang region of *P. falciparum* is a repetition of degenerate unit 5′-GGGTTYA-3′ (where Y could be T or C)^41^ which can fold into intramolecular G-quadruplex^42^. This difference between parasitic and human (5′-GGGTTA-3′) G-quadruplexes is also observed with *L.* spp and *T. brucei brucei*, which augurs the possibility of developing antiparasitic ligands targeting G-quadruplexes found in these protozoal species. Thus, we investigated whether these derivatives could stabilise some parasitic telomeric DNA G-quadruplex structures. Consequently, potential stabilisation of *P. falciparum* and *T. brucei brucei* telomeric G-quadruplexes was evaluated using a FRET melting assay.

## Experimental

### Chemistry

The received commercial reagents were used without additional purification. Melting points were determined with a SM-LUX-POL Leitz hot-stage microscope and are uncorrected. IR spectra were recorded on a NICOLET 380FT-IR spectrophotometer. NMR spectra were recorded with tetramethylsilane as an internal standard using a BRUKER AVANCE 300 spectrometer. Splitting patterns have been designated as follows: s = singlet; bs = broad singlet; d = doublet; t = triplet; q = quartette; qt = quintuplet, dd = double doublet; ddd = double double doublet; and m = multiplet. Analytical TLC was carried out on 0.25 precoated silica gel plates (POLYGRAM SIL G/UV254) and visualisation of compounds after UV light irradiation. Silica gel 60 (70–230 mesh) was used for column chromatography. Microwave experiments were carried out at atmospheric pressure using a focussed microwave reactor (CEM Discover). High-resolution mass spectra (electrospray in positive mode, ESI + or MALDI-TOF MS) were recorded on a Waters Q-TOF Ultima apparatus. Elemental analyses were found within ±0.4% of the theoretical values.

### General procedure for 2,4-bis(4-formylphenyl)quinolines (4a–d), 1,3-bis(4-formylphenyl)isoquinolines(5a–c) and 2,4-bis(4-formylphenyl) quinazolines (6a–d)

To a solution of 4.37 mmol of the appropriate 2,4-dichloroquinoline, or 1,3-dichloroisoquinoline or 2,4-dichloroquinazoline, 1.44 g of 3- or 4-formylphenyl boronic acid (9.63 mmol, 2.2 eq.) and 506 mg (0.437 mmol, 0.1 eq.) of tetrakis(triphenylphosphine) palladium in 45 ml of 1,2-dimethoxyethane, 5 ml of 2 M K_2_CO_3_ aqueous solution, previously degassed for 10 min with nitrogen, were added at room temperature. Then, the mixture was warmed to reflux and stirred for 24 h under nitrogen positive pressure. The reaction mixture was cooled down to room temperature and the solvent was evaporated under vacuum. The organic layer was extracted with CH_2_Cl_2_ and the organic phase was filtered on filter paper, then washed with water (20 ml× 3 times), dried over anhydrous sodium sulphate and activated charcoal, filtered, and evaporated under vacuum. The residue was cooled and triturated with a minimum of EtOH and EtO_2_ and filtered on sintered glassware to give the crude product. The residue was purified by silica gel column chromatography (CH_2_Cl_2_/CH_3_OH 95:5), then cooled and triturated again in EtOH, filtered on sintered glassware, washed with a minimum of EtOH, EtO_2_ and petroleum ether and dried under pressure to give the solid product.

**2,4-bis(4-Formylphenyl)quinoline (4a)**

White crystals (60%); Mp = 186 °C; IR ν_max_ (KBr)/cm^−1^ 1695 (C=O);^1^H NMR δ (300 MHz, CDCl_3_) 10.19 (s, 1H, CHO), 10.14 (s, 1H, CHO), 8.41 (d, 2H, *J*= 9.00 Hz, H-3′′ and H-5′′), 8.33 (dd, 1H, *J*= 9.00 and 1.60 Hz, H-8), 8.12 (d, 2H, *J*= 9.00 Hz, H-3′ and H-5′), 8.08 (d, 2H, *J*= 9.00 Hz, H-2′′ and H-6′′), 7.90 (s, 1H, H-3), 7.87(dd, 1H, *J*= 9.00 and 1.60 Hz, H-5), 7.83 (ddd, *J*= 9.00, 7.20 and 1.60 Hz, H-7), 7.77 (d, 2H, *J*= 9.00 Hz, H-2′ and H-6'), 7.59 (ddd, 1H, *J*= 9.00, 7.20 and 1.60 Hz, H-6); HRMS-ESI m/z [M + H]^+^ Calcd for C_23_H_16_NO_2_: 338.1181, Found: 338.1182.

**6-Methoxy-2,4-bis(4-formylphenyl)quinoline (4b)**

Pale yellow crystals (44.1%); Mp = 212 °C; Rf = 0.84; IR ν_max_ (KBr)/cm^−1^ 1695 (C=O);^1^H NMR δ (300 MHz, CDCl_3_) 10.18 (s, 1H, CHO), 10.12 (s, 1H, CHO), 8.38 (d, 2H, *J*= 8.25 Hz, H-3′′ and H-5′′), 8.20 (d, 1H, *J*= 9.30 Hz, H-8), 8.12 (d, 2H, *J*= 8.25 Hz, H-3′ and H-5′), 8.00 (d, 2H, *J*= 8.25 Hz, H-2′′ and H-6′′), 7.84 (s, 1H, H-3), 7.79 (d, 2H, *J*= 8.25 Hz, H-2′ and H-6′), 7.47 (dd, 1H, *J*= 9.30 and 2.70 Hz, H-7), 7.10 (d, 1H, *J*= 2.70 Hz, H-5), 3.83 (s, 3H, CH_3_O); 13C NMR δ (75 MHz, CDCl_3_) 193.4 (CHO), 193.1 (CHO), 160.0 (C-6), 154.2 (C-2), 148.2 (C-4), 148.0 (C-8a), 146.3 (C-4′), 146.0 (C-4′′), 137.8 (C-1′), 137.6 (C-1′′), 133.4 (C-8), 131.5 (C-3′ and C-5′, C-3′′ and C-5′′, C-2′ and C-6′), 129.2 (C-2′′ and C-6′′), 127.9 (C-4a), 124.1 (C-7), 120.8 (C-3), 104.4 (C-5), 56.9 (CH_3_O); HRMS-ESI *m/z* [M + H]^+^ Calcd for C_24_H_18_NO_3_: 368.1287, Found: 368.1289.

**7-Methoxy-2,4-bis(4-formylphenyl)quinoline (4c)**

Pale yellow crystals (71.4%); Mp = 179 °C; Rf = 0.83; IR ν_max_ (KBr)/cm^−1^ 1696 (C=O); ^1^H NMR δ (300 MHz, CDCl_3_) 10.18 (s, 1H, CHO), 10.14 (s, 1H, CHO), 8.39 (d, 2H, *J*= 8.30 Hz, H-3′′ and H-5′′), 8.11 (d, 2H, *J*= 8.30 Hz, H-3′ and H-5′), 8.07 (d, 2H, *J*= 8.30 Hz, H-2′′ and H-6′′), 7.77 (d, 2H, *J*= 8.30 Hz, H-2′ and H-6′), 7.76 (s, 1H, H-3), 7.75 (d, 1H, *J*= 9.30 Hz, H-5), 7.63 (d, 1H, *J*= 2.60 Hz, H-8), 7.23 (dd, 1H, *J*= 9.30 and 2.60 Hz, H-6), 4.04 (s, 3H, CH_3_O); HRMS-ESI *m/z* [M + H]^+^ Calcd for C_24_H_18_NO_3_: 368.1287, Found: 368.1287.

**2,4-bis(3-Formylphenyl)quinoline (4d)**

White crystals (66%); Mp = 150 °C; IR ν_max_ (KBr)/cm^−1^1694 (C=O); ^1^H NMR δ (300 MHz, CDCl_3_) 10.19 (s, 1H, CHO), 10.18 (s, 1H, CHO), 8.74 (dd, 1H, *J*= 1.50 and 1.50 Hz, H-2′), 8.57 (ddd, 1H, *J*= 7.80, 1.50 and 1.50 Hz, H-6′), 8.33 (dd, 1H, *J*= 8.70 and 1.20 Hz, H-8), 8.13 (dd, 1H, *J*= 1.50 and 1.50 Hz, H-2′′), 8.09 (ddd, 1H, *J*= 7.80, 1.50 and 1.50 Hz, H-4′), 8.03 (ddd, 1H, *J*= 7.80, 1.50 and 1.50 Hz, H-4′′), 7.93 (s, 1H, H-3), 7.91–785 (m, 2H, H-5 and H-6′′), 7.83 (ddd, 1H, *J*= 8.70, 7.20 and 1.20 Hz, H-7), 7.81 (t, 1H, *J*= 7.80 Hz, H-5′), 7.75 (t, 1H, *J*= 7.80 Hz, H-5′′), 7.58 (ddd, 1H, *J*= 8.70, 7.20 and 1.20 Hz, H-6); 13C NMR δ (75 MHz, CDCl_3_) 193.5 (C=O), 193.1 (C=O), 156.5 (C-2), 150.0 (C-4), 149.5 (C-8a), 141.5 (C-1′′), 140.5 (C-3′), 138.3 (C-3′′), 138.2 (C-1′), 136.7 (C-6′), 134.7 (C-6′′), 131.9 (C-2′ and C-4′′), 131.7 (C-2′′), 131.5 (C-4′), 131.3 (C-7), 131.0 (C-5′′), 130.9 (C-5′), 130.1 (C-8), 128.6 (C-5), 127.0 (C-4a), 126.5 (C-6), 120.3 (C-3); HRMS-ESI *m/z* [M + H]^+^ Calcd for C_23_H_16_NO_2_: 338.1181, Found: 338.1182.

**1,3-bis(4-Formylphenyl)isoquinoline (5a)**

Beige crystals (72%); Mp = 194 °C; IR ν_max_ (KBr)/cm^−1^ 1695 (C=O); ^1^H NMR δ (300 MHz, CDCl_3_) 10.20 (s, 1H, CHO), 10.12 (s, 1H, CHO), 8.41 (d, 2H, *J*= 8.40 Hz, H-3′′ and H-5′′), 8.26 (s, 1H, H-4), 8.14–8.09 (m, 3H, H-8, H-3′ and H-5′), 8.06–8.00 (m, 5H, H-2′′, H-6′′, H-2′, H-6′ and H-5), 7.79 (ddd, 1H, *J*= 7.10, 6.90 and 1.50 Hz, H-6), 7.63 (ddd, 1H, *J*= 7.10, 6.90 and 1.50 Hz, H-7); HRMS-ESI *m/z* [M + H]^+^ Calcd for C_23_H_16_NO_2_: 338.1181, Found: 338.1183.

**7-Methoxy-1,3-bis(4-formylphenyl)isoquinoline (5b)**

Pale-yellow crystals (57%); Mp = 190 °C; IR ν_max_ (KBr)/cm^−1^ 1692 (C=O); ^1^H NMR δ (300 MHz, CDCl_3_) 10.19 (s, 1H, CHO), 10.10 (s, 1H, CHO), 8.38 (d, 2H, *J*= 8.40 Hz, H-2′′ and H-6′′), 8.19 (s, 1H, H-4), 8.13 (d, 2H, *J*= 8.40 Hz, H-2′ and H-6′), 8.04 (d, 2H, *J*= 8.40 Hz, H-3′′ and H-5′′), 8.02 (d, 2H, *J*= 8.40 Hz, H-3′ and H-5′), 7.94 (d, 1H, *J*= 9.00 Hz, H-5), 7.44 (dd, 1H, *J*= 9.00 and 2.40 Hz, H-6), 7.36 (d, 1H, *J*= 2.40 Hz, H-8), 3.87 (s, 3H, CH_3_O); HRMS-ESI *m/z* [M + H]^+^ Calcd for C_24_H_18_NO_3_: 368.1287, Found: 368.1286.

**6-Methoxy-1,3-bis(4-formylphenyl)isoquinoline (5c)**

White crystals (76%); Mp = 191 °C; IR ν_max_ (KBr)/cm^−1^ 1695 (C=O); ^1^H NMR δ (300 MHz, CDCl_3_) 10.20 (s, 1H, CHO), 10.10 (s, 1H, CHO), 8.37 (d, 2H, *J*= 8.40 Hz, H-2′′ and H-6′′), 8.12 (d, 1H, *J*= 9.00 Hz, H-8), 8.10 (d, 2H, *J*= 8.40 Hz, H-2′ and H-6′), 8.02 (d, 2H, *J*= 8.40 Hz, H-3′′ and H-5′′), 8.00 (s, 1H, H-4), 7.97 (d, 2H, *J*= 8.40 Hz, H-3′ and H-5′), 7.26 (d, 1H, *J*= 2.40 Hz, H-5), 7.23 (dd, 1H, *J*= 9.00 and 2.40 Hz, H-7), 4.02 (s, 3H, CH_3_O); HRMS-ESI *m/z* [M + H]^+^ Calcd for C_24_H_18_NO_3_: 368.1287, Found: 368.1293.

**2,4-bis(4-Formylphenyl)quinazoline (6a)**

White crystals (53%); Mp = 194 °C; IR ν_max_ (KBr)/cm^−1^ 1698 (C=O); ^1^H NMR δ (300 MHz, CDCl_3_) 10.22 (s, 1H, CHO), 10.16 (s, 1H, CHO), 8.89 (d, 2H, *J*= 8.40 Hz, H-3′′ and H-5′′), 8.25 (dd, 1H, *J*= 8.40 and 1.35 Hz, H-8), 8.17 (d, 2H, *J*= 8.40 Hz, H-3′ and H-5′), 8.14 (dd, 1H, *J*= 8.40 and 1.35 Hz, H-5), 8.10–8.06 (m, 4H, H-2′′, H-6′′, H-2′ and H-6′),8.00 (ddd, 1H, *J*= 8.40, 7.05 and 1.35 Hz, H-7), 7.67 (ddd, 1H, *J*= 8.40, 7.05 and 1.35 Hz, H-6); 13C NMR δ (75 MHz, CDCl_3_) 193.6 (C=O), 193.2 (C=O), 168.6 (C-2), 160.3 (C-4), 153.3 (C-8a), 144.7 (C-1′′), 144.4 (C-1′), 139.0 (C-4′′), 138.5 (C-4′), 135.6 (C-7), 132.2 (C-3′′ and C-5′′), 131.3 (C-2′′, C-6′′, C-3′ and C-5′), 131.0 (C-8), 130.5 (C-2′ and C-6′), 129.6 (C-5), 127.8 (C-6), 123.1 (C-4a); HRMS-ESI *m/z* [M + H]^+^ Calcd for C_22_H_15_N_2_O_2_: 339.1134, Found: 339.1126.

**6-Methoxy-2,4-bis(4-formylphenyl)quinazoline (6b)**

Pale yellow crystals (32%); Mp = 220 °C; Rf = 0.81; IR ν_max_ (KBr)/cm^−1^ 1697 (C=O); ^1^H NMR δ (300 MHz, CDCl_3_) 10.21 (s, 1H, CHO), 10.13 (s, 1H, CHO), 8.83 (d, 2H, *J*= 8.40 Hz, H-3′′ and H-5′′), 8.18–8.10 (m, 7H, H-8, H-3′ and H-5′, H-2′′ and H-6′′, H-2′ and H-6′), 7.63 (dd, 1H, *J*= 9.20 and 2.90 Hz, H-7), 7.31 (d, 1H, *J*= 2.90 Hz, H-5), 3.89 (s, 3H, CH_3_O); HRMS-ESI *m/z* [M + H]^+^ Calcd for C_23_H_17_N_2_O_3_: 369.1239, Found: 369.1236.

**7-Methoxy-2,4-bis(4-formylphenyl)quinazoline (6c)**

Yellow crystals (73%); Mp = 238 °C; Rf = 0.78; IR ν_max_ (KBr)/cm^−1^ 1697 (C=O); ^1^H NMR δ (300 MHz, CDCl_3_) 10.20 (s, 1H, CHO), 10.15 (s, 1H, CHO), 8.85 (d, 2H, *J*= 8.40 Hz, H-2′′ and H-6′′), 8.14 (d, 2H, *J*= 8.40 Hz, H-2′ and H-6′), 8.06 (d, 2H, *J*= 8.40 Hz, H-3′′ and H-5′′), 8.04 (d, 2H, *J*= 8.40 Hz, H-3′ and H-5′), 7.96 (d, 1H, *J*= 9.30 Hz, H-5), 7.51 (d, 1H, *J*= 2.55 Hz, H-8), 7.26 (d, 1H, *J*= 9.30 and 2.55 Hz, H-6), 4.07 (s, 3H, CH_3_O); HRMS-ESI *m/z* [M + H]^+^ Calcd for C_23_H_17_N_2_O_3_: 369.1239, Found: 369.1231.

**2,4-bis(3-Formylphenyl)quinazoline (6d)**

White crystals (54%); Mp = 174 °C; IR ν_max_ (KBr)/cm^−1^ 1697 (C=O); ^1^H NMR δ (300 MHz, CDCl_3_) 10.21 (s, 1H, CHO), 10.20 (s, 1H, CHO), 9.20 (dd, 1H, *J*= 1.35 and 1.35 Hz, H-2′), 8.99 (ddd, 1H, *J*= 7.70, 1.35 and 1.35 Hz, H-6′), 8.42 (dd, 1H, *J*= 1.35 and 1.35 Hz, H-2′′), 8.24–8.18 (m, 2H, H-8 and H-6′′), 8.15 (ddd, 1H, *J*= 7.70, 1.35 and 1.35 Hz, H-4′), 8.17–8.07 (m, 1H, H-5), 8.05 (ddd, 1H, *J*= 7.70, 1.35 and 1.35 Hz, H-4′′), 7.98 (ddd, 1H, *J*= 8.30, 7.20 and 1.50 Hz, H-7), 7.83 (dd, 1H, *J*= 7.70 and 7.70 Hz, H-5′′), 7.72 (dd, 1H, *J*= 7.70 and 7.70 Hz, H-5′), 7.65 (ddd, 1H, *J*= 8.30, 7.20 and 1.50 Hz, H-6); HRMS-ESI *m/z* [M + H]^+^ Calcd for C_22_H_15_N_2_O_2_: 339.1134, Found: 339.1128.

### General procedure for 2,4-bis[(substituted-iminomethyl)phenyl]quinolines (7a-t), 1,3-bis[(substituted-iminomethyl)phenyl]isoquinolines (8a–l), and 2,4-bis[(substituted-iminomethyl)phenyl]quinazolines (9a–v)

To a solution of diamine (0.126 mmol, 2.1 eq.) in ethanol (7 ml) was added 2,4-bis(4-formylphenyl)quinoline **4** or 1,3-bis(4-formylphenyl)isoquinoline **5** or 2,4-bis(4-diformylphenyl)quinazoline **6**(0.6 mmol). The reaction mixture was then heated under reflux for 5 h, and then evaporated to dryness under reduced pressure. After cooling, the residue was extracted with dichloromethane (40 ml). The organic layer was dried over sodium sulphate and activated charcoal and evaporated to dryness. Products were then used without further purification.

**2,4-Bis{4-[(4-dimethylaminobutyl)iminomethyl]phenyl}quinoline (7a)**

Yellow oil (97%); ^1^H NMR δ (300 MHz, CDCl_3_) 8.36 (s, 1H, CH=N), 8.32 (s, 1H, CH=N), 8.23 (d, 2H, *J*= 8.40 Hz, H-3′′ and H-5′′), 8.21 (dd, 1H, *J*= 8.50 and 1.35 Hz, H-8), 7.87 (d, 2H, *J*= 8.40 Hz, H-3′ and H-5′), 7.86 (s, 1H, H-3), 7.82 (dd, 1H, *J*= 8.50 and 1.35 Hz, H-5), 7.81 (d, 2H, *J*= 8.40 Hz, H-2′′ and H-6′′), 7.72 (ddd,1H, *J*= 8.50, 7.00 and 1.35 Hz, H-7), 7.57 (d, 2H, *J*= 8.40 Hz, H-2′ and H-6′′), 7.45 (ddd, 1H, *J*= 8.50, 7.00 and 1.35 Hz, H-6), 3.67 (t, 2H, *J*= 6.90 Hz, NCH_2_), 3.64 (t, 2H, *J*= 6.90 Hz, NCH_2_), 2.34–2.26 (m. 4H, 2NCH_2_), 2.21 (s, 6H, N(CH_3_)_2_), 2.20 (s, 6H, N(CH_3_)_2_), 1.80–1.68 (m, 4H, 2CH_2_), 1.61–1.52 (m, 4H, 2CH_2_).

**2,4-Bis{4-[(3-dimethylaminopropyl)iminomethyl]phenyl}quinoline (7b)**

Yellow oil (98%); ^1^H NMR δ (300 MHz, CDCl_3_) 8.42 (s, 1H, CH=N), 8.38 (s, 1H, CH=N), 8.28 (d, 2H, *J*= 9.00 Hz, H-3′′ and H-5′′), 8.26 (dd, 1H, *J*= 8.90 and 1.50 Hz, H-8), 7.94 (d, 2H, *J*= 9.00 Hz, H-3′ and H-5′), 7.89 (dd, 1H, *J*= 8.90 and 1.50 Hz, H-5), 7.88 (d, 2H, *J*= 9.00 Hz, H-2′′ and H-6′′), 7.85 (s, 1H, H-3), 7.76 (ddd,1H, *J*= 8.90, 7.20 and 1.50 Hz, H-7), 7.62 (d, 2H, *J*= 9.00 Hz, H-2′ and H-6′), 7.50 (ddd, 1H, *J*= 8.90, 7.20 and 1.50 Hz, H-6), 3.73 (t, 2H, *J*= 6.90 Hz, NCH_2_), 3.69 (t, 2H, *J*= 6.90 Hz, NCH_2_), 2.46–2.40 (m. 4H, 2NCH_2_), 2.30 (s, 6H, N(CH_3_)_2_), 2.29 (s, 6H, N(CH_3_)_2_), 2.00–1.89 (m, 4H, 2CH_2_).

**2,4-Bis{4-[(2-dimethylaminoethyl)iminomethyl]phenyl}quinoline (7c)**

Yellow oil (98%); ^1^H NMR δ (300 MHz, CDCl_3_) 8.43 (s, 1H, CH=N), 8.40 (s, 1H, CH=N), 8.27 (d, 2H, *J*= 9.00 Hz, H-3′′ and H-5′′), 8.24 (dd, 1H, *J*= 8.90 and 1.50 Hz, H-8), 7.92 (d, 2H, *J*= 9.00 Hz, H-3′ and H-5′), 7.89 (d, 2H, *J*= 9.00 Hz, H-2′′ and H-6′′),7.86 (dd, 1H, *J*= 8.90 and 1.50 Hz, H-5), 7.84 (s, 1H, H-3), 7.75 (ddd,1H, *J*= 8.90, 7.20 and 1.50 Hz, H-7), 7.61 (d, 2H, *J*= 9.00 Hz, H-2′ and H-6′), 7.49 (ddd, 1H, *J*= 8.90, 7.20 and 1.50 Hz, H-6), 3.82 (t, 2H, *J*= 7.00 Hz, NCH_2_), 3.80 (t, 2H, *J*= 7.00 Hz, NCH_2_), 2.71 (t, 2H, *J*= 7.00 Hz, NCH_2_), 2.69 (t, 2H, *J*= 7.00 Hz, NCH_2_), 2.36 (s, 6H, N(CH_3_)_2_), 2.35 (s, 6H, N(CH_3_)_2_).

**2,4-Bis{4-[(4–(4-methylpiperazin-1-yl)butyl)iminomethyl]phenyl}quinoline (7d)**

Yellow oil (97%); ^1^H NMR δ (300 MHz, CDCl_3_) 8.39 (s, 1H, CH=N), 8.35 (s, 1H, CH=N), 8.27–8.23 (m, 3H, H-3′′, H-5′′ and H-8), 7.90 (d, 2H, *J*= 8.10 Hz, H-3′ and H-5′), 7.88 (dd, 1H, *J*= 8.10 and 1.20 Hz, H-5), 7.87 (d, 2H, *J*= 8.10 Hz, H-2′′ and H-6′′), 7.85 (s, 1H, H-3), 7.75 (ddd,1H, *J*= 8.10, 6.90 and 1.20 Hz, H-7), 7.61 (d, 2H, *J*= 8.10 Hz, H-2′ and H-6′), 7.49 (ddd, 1H, *J*= 8.10, 6.90 and 1.20 Hz, H-6), 3.69 (t, 2H, *J*= 6.90 Hz, NCH_2_), 3.67 (t, 2H, *J*= 6.90 Hz, NCH_2_), 2.74–2.35 (m. 20H, 2NCH_2_ and 8NCH_2_pip.), 2.29 (s, 3H, NCH_3_), 2.28 (s, 3H, NCH_3_), 1.79–1.74 (m, 4H, 2CH_2_), 1.62–1.58 (m, 4H, 2CH_2_).

**2,4-Bis{4-[(3–(4-methylpiperazin-1-yl)propyl)iminomethyl]phenyl}quinoline (7e)**

Yellow oil (98%); ^1^H NMR δ (300 MHz, CDCl_3_) 8.41 (s, 1H, CH=N), 8.37 (s, 1H, CH=N), 8.27 (d, 2H, *J*= 8.40 Hz, H-3′′ and H-5′′), 8.25 (dd, 1H, *J*= 8.40 and 1.50 Hz, H-8), 7.91 (d, 2H, *J*= 8.40 Hz, H-3′ and H-5′), 7.88 (dd, 1H, *J*= 8.40 and 1.50 Hz, H-5), 7.87 (d, 2H, *J*= 8.40 Hz, H-2′′ and H-6′′), 7.85 (s, 1H, H-3), 7.76 (ddd,1H, *J*= 8.40, 6.90 and 1.50 Hz, H-7), 7.63 (d, 2H, *J*= 8.40 Hz, H-2′ and H-6′), 7.50 (ddd, 1H, *J*= 8.40, 6.90 and 1.50 Hz, H-6), 3.72 (t, 2H, *J*= 6.90 Hz, NCH_2_), 3.70 (t, 2H, *J*= 6.90 Hz, NCH_2_), 2.65–2.46 (m. 20H, 2NCH_2_ and 8NCH_2_pip.), 2.32 (s, 3H, NCH_3_), 2.31 (s, 3H, NCH_3_), 2.00–1.92 (m, 4H, 2CH_2_).

**2,4-Bis{4-[(2–(4-methylpiperazin-1-yl)ethyl)iminomethyl]phenyl}quinoline (7f)**

Yellow oil (97%); ^1^H NMR δ (300 MHz, CDCl_3_) 8.40 (s, 1H, CH=N), 8.36 (s, 1H, CH=N), 8.25 (d, 2H, *J*= 9.00 Hz, H-3′′ and H-5′′), 8.23 (dd, 1H, *J*= 8.50 and 1.40 Hz, H-8), 7.89 (d, 2H, *J*= 9.00 Hz, H-3′ and H-5′), 7.86 (d, 2H, *J*= 9.00 Hz, H-2′′ and H-6′′), 7.85 (dd, 1H, *J*= 8.50 and 1.40 Hz, H-5), 7.83 (s, 1H, H-3), 7.74 (ddd,1H, *J*= 8.50, 6.90 and 1.20 Hz, H-7), 7.60 (d, 2H, *J*= 9.00 Hz, H-2′ and H-6′), 7.48 (ddd, 1H, *J*= 8.50, 6.90 and 1.20 Hz, H-6), 3.85–3.66 (m, 4H, 2NCH_2_), 2.83–2.72 (m, 4H, 2NCH_2_), 2.62–2.42 (m. 20H, 2NCH_2_ and 8NCH_2_pip.), 2.29 (s, 3H, NCH_3_), 2.28 (s, 3H, NCH_3_).

**2,4-Bis{4-[(3-morpholinopropyl)iminomethyl]phenyl}quinoline (7g)**

Yellow oil (98%); ^1^H NMR δ (300 MHz, CDCl_3_) 8.43 (s, 1H, CH=N), 8.39 (s, 1H, CH=N), 8.28 (d, 2H, *J*= 8.40 Hz, H-3′′ and H-5′′), 8.26 (dd, 1H, *J*= 8.00 and 1.50 Hz, H-8), 7.93 (d, 2H, *J*= 8.40 Hz, H-3′ and H-5′), 7.90 (dd, 1H, *J*= 8.00 and 1.50 Hz, H-5), 7.88 (d, 2H, *J*= 8.40 Hz, H-2′′ and H-6′′), 7.86 (s, 1H, H-3), 7.77 (ddd,1H, *J*= 8.00, 7.20 and 1.50 Hz, H-7), 7.64 (d, 2H, *J*= 8.40 Hz, H-2′ and H-6′), 7.51 (ddd, 1H, *J*= 8.00, 7.20 and 1.50 Hz, H-6), 3.78–3.69 (m, 12H, 2NCH_2_ and 4 OCH_2_), 2.52–2.45 (m, 12H, 6NCH_2_), 2.02–1.90 (m, 4H, 2CH_2_).

**2,4-Bis{4-[(2-morpholinoethyl)iminomethyl]phenyl}quinoline (7h)**

Orange oil (98%); ^1^H NMR δ (300 MHz, CDCl_3_) 8.43 (s, 1H, CH=N), 8.39 (s, 1H, CH=N), 8.27 (d, 2H, *J*= 8.40 Hz, H-3′′ and H-5′′), 8.25 (dd, 1H, *J*= 8.70 and 1.50 Hz, H-8), 7.93 (d, 2H, *J*= 8.40 Hz, H-3′ and H-5′), 7.90 (dd, 1H, *J*= 8.70 and 1.50 Hz, H-5), 7.88 (d, 2H, *J*= 8.40 Hz, H-2′′ and H-6′′), 7.86 (s, 1H, H-3), 7.77 (ddd,1H, *J*= 8.70, 7.10 and 1.50 Hz, H-7), 7.63 (d, 2H, *J*= 8.40 Hz, H-2′ and H-6′), 7.51 (ddd, 1H, *J*= 8.70, 7.10 and 1.50 Hz, H-6), 3.86 (t, 2H, *J*= 6.90 Hz, NCH_2_), 3.83 (t, 2H, *J*= 6.90 Hz, NCH_2_), 3.78 (m, 8H, 4 OCH_2_), 2.80–2.73 (m, 4H, 2NCH_2_), 2.62–2.57 (m, 8H, 4NCH_2_).

**6-Methoxy-2,4-bis{4-[(4-dimethylaminobutyl)iminomethyl]phenyl}quinoline (7i)**

Yellow oil (98%); ^1^H NMR δ (300 MHz, CDCl_3_) 8.35 (s, 1H, CH=N), 8.30 (s, 1H, CH=N), 8.19 (d, 2H, *J*= 8.10 Hz, H-3′′ and H-5′′), 8.10 (d, 1H, *J*= 9.30 Hz, H-8), 7.87 (d, 2H, *J*= 8.10 Hz, H-3′ and H-5′), 7.82 (d, 2H, *J*= 8.10 Hz, H-2′′ and H-6′′), 7.75 (s, 1H, H-3), 7.58 (d, 2H, *J* = 8.10 Hz, H-2′ and H-6′), 7.35 (dd,1H, *J*= 9.30 and 2.60 Hz, H-7), 7.10 (m, 1H, H-5), 3.74 (s, 3H, CH_3_O), 3.67–3.62 (m, 4H, 2NCH_2_), 2.33–2,25 (m. 4H, 2NCH_2_), 2.21 (s, 6H, N(CH_3_)_2_), 2.19 (s, 6H, N(CH_3_)_2_), 1.76–1.70 (m, 4H, 2CH_2_), 1.56–1.50 (m, 4H, 2CH_2_).

**6-Methoxy-2,4-bis{4-[(3-dimethylaminopropyl)iminomethyl]phenyl}quinoline (7j)**

Yellow oil (98%); ^1^H NMR δ (300 MHz, CDCl_3_) 8.38 (s, 1H, CH=N), 8.32 (s, 1H, CH=N), 8.20 (d, 2H, *J*= 8.25 Hz, H-3′′ and H-5′′), 8.11 (d, 1H, *J*= 9.00 Hz, H-8), 7.89 (d, 2H, *J*= 8.25 Hz, H-3′ and H-5′), 7.84 (d, 2H, *J*= 8.25 Hz, H-2′′ and H-6′′), 7.76 (s, 1H, H-3), 7.60 (d, 2H, *J* = 8.25 Hz, H-2′and H-6′), 7.36 (dd, 1H, *J*= 9.00 and 2.70 Hz, H-7), 7.12 (d, 1H, *J*= 2.70 Hz, H-5), 3.74 (s, 3H, CH_3_O) 3.69 (t, 2H, *J*= 6.90 Hz, NCH_2_), 3.65 (t, 2H, *J*= 6.90 Hz, NCH_2_), 2.37 (t, 2H, *J*= 6.90 Hz, NCH_2_), 2.35 (t, 2H, *J*= 6.90 Hz, NCH_2_), 2.24 (s, 6H, N(CH_3_)_2_), 2.22 (s, 6H, N(CH_3_)_2_), 1.95–1.80 (m, 4H, 2CH_2_).

**6-Methoxy-2,4-bis{4-[(4–(4-methylpiperazin-1-yl)butyl)iminomethyl]phenyl}quinoline (7k)**

Pale yellow oil (98%); ^1^H NMR δ (300 MHz, CDCl_3_) 8.31 (s, 1H, CH=N), 8.25 (s, 1H, CH=N), 8.16 (d, 2H, *J*= 8.50 Hz, H-3′′ and H-5′′), 8.07 (d, 1H, *J*= 9.30 Hz, H-8), 7.84 (d, 2H, *J*= 8.50 Hz, H-3′ and H-5′), 7.78 (d, 2H, *J*= 8.50 Hz, H-2′′ and H-6′′), 7.72 (s, 1H, H-3), 7.55 (d, 2H, *J*= 8.50 Hz, H-2′ and H-6′), 7.32 (dd,1H, *J*= 9.30 and 2.80 Hz, H-7), 7.07 (d, 1H, *J*= 2.80 Hz, H-5), 3.70 (s, 3H, CH_3_O), 3.62 (t, 2H, *J*= 6.60 Hz, NCH_2_), 3.58 (t, 2H, *J*= 6.60 Hz, NCH_2_), 2.50–2.29 (m, 20H, 2NCH_2_ and 8NCH_2_ pip), 2.21 (s, 3H, NCH_3_), 2.20 (s, 3H, NCH_3_), 1.75–1.63 (m, 4H, 2CH_2_), 1.59–1.46 (m, 4H, 2CH_2_).

**6-Methoxy-2,4-bis{4-[(3–(4-methylpiperazin-1-yl)propyl)iminomethyl]phenyl}quinoline (7l)**

Yellow oil (98%); ^1^H NMR δ (300 MHz, CDCl_3_) 8.35 (s, 1H, CH=N), 8.30 (s, 1H, CH=N), 8.18 (d, 2H, *J*= 8.10 Hz, H-3′′ and H-5′′), 8.09 (d, 1H, *J*= 9.30 Hz, H-8), 7.86 (d, 2H, *J*= 8.10 Hz, H-3′ and H-5′), 7.81 (d, 2H, *J*= 8.10 Hz, H-2′′ and H-6′′), 7.74 (s, 1H, H-3), 7.58 (d, 2H, *J*= 8.10 Hz, H-2′ and H-6′), 7.34 (dd,1H, *J*= 9.30 and 2.60 Hz, H-7), 7.09 (d, 1H, *J*= 2.60 Hz, H-5), 3.73 (s, 3H, CH_3_O), 3.67 (t, 2H, *J*= 6.60 Hz, NCH_2_), 3.63 (t, 2H, *J*= 6.60 Hz, NCH_2_), 2.47–2.38 (m, 20H, 2NCH_2_ and 8NCH_2_ pip), 2.25 (s, 3H, NCH_3_), 2.24 (s, 3H, NCH_3_), 1.93–1.85 (m, 4H, 2CH_2_).

**7-Methoxy-2,4-bis{4-[(4-dimethylaminobutyl)iminomethyl]phenyl}quinoline (7m)**

Yellow oil (98%); ^1^H NMR δ (300 MHz, CDCl_3_) 8.34 (s, 1H, CH=N), 8.31 (s, 1H, CH=N), 8.20 (d, 2H, *J*= 8.20 Hz, H-3′′ and H-5′′), 7.86 (d, 2H, *J*= 8.20 Hz, H-3′ and H-5′), 7.83 (d, 2H, *J*= 8.20 Hz, H-2′′ and H-6′′), 7.72 (d, 1H, *J*= 9.15 Hz, H-5), 7.66 (s, 1H, H-3), 7.55 (d, 2H, *J*= 8.20 Hz, H-2′ and H-6′), 7.53 (d, 1H, *J*= 2,70 Hz, H-8), 7.09 (dd, 1H, *J*= 9.15 and 2.70 Hz, H-6), 3.94 (s, 3H, CH_3_O) 3.65 (t, 2H, *J*= 7.10 Hz, NCH_2_), 3.63 (t, 2H, *J*= 7.10 Hz, NCH_2_), 2.30 (t, 2H, *J*= 7.10 Hz, NCH_2_), 2.39 (t, 2H, *J*= 7.10 Hz, NCH_2_), 2.20 (s, 6H, N(CH_3_)_2_), 2.19 (s, 6H, N(CH_3_)_2_), 1.78–1.67 (m, 4H, 2CH_2_), 1.60–1.48 (m, 4H, 2CH_2_).

**7-Methoxy-2,4-bis{4-[(3-dimethylaminopropyl)iminomethyl]phenyl}quinoline (7n)**

Yellow oil (98%); ^1^H NMR δ (300 MHz, CDCl_3_) 8.36 (s, 1H, CH=N), 8.33 (s, 1H, CH=N), 8.20 (d, 2H, *J*= 8.40 Hz, H-3′′ and H-5′′), 7.89–7.83 (m, 4H, H-3′ and H-5′, H-2′′ and H-6′′), 7.73 (d, 1H, *J*= 9.30 Hz, H-5), 7.67 (d, 1H, *J*= 2,10 Hz, H-8), 7.57–7.53 (m, 3H, H-2′ and H-6′, H-3), 7.10 (dd, 1H, *J*= 9.30 and 2.10 Hz, H-6), 3.95 (s, 3H, CH_3_O) 3.68 (t, 2H, *J*= 6.90 Hz, NCH_2_), 3.65 (t, 2H, *J*= 6.90 Hz, NCH_2_), 2.36–2,32 (m, 4H, 2NCH_2_), 2.23 (s, 6H, N(CH_3_)_2_), 2.22 (s, 6H, N(CH_3_)_2_), 1.95–1.80 (m, 4H, 2CH_2_).

**7-Methoxy-2,4-bis{4-[(4–(4-methylpiperazin-1-yl)butyl)iminomethyl]phenyl}quinoline (7o)**

Yellow oil (98%); ^1^H NMR δ (300 MHz, CDCl_3_) 8.32 (s, 1H, CH=N), 8.29 (s, 1H, CH=N), 8.19 (d, 2H, *J*= 8.10 Hz, H-3′′and H-5′′), 7.84 (d, 2H, *J*= 8.10 Hz, H-3′ and H-5′), 7.81 (d, 2H, *J*= 8.20 Hz, H-2′′and H-6′′), 7.71 (d, 1H, *J*= 9.30 Hz, H-5), 7.65 (s, 1H, H-3), 7.54 (d, 2H, *J*= 8.10 Hz, H-2′ and H-6′), 7.52 (d, 1H, *J*= 2.40 Hz, H-8), 7.09 (dd,1H, *J*= 9.30 and 2.40 Hz, H-6), 3.94 (s, 3H, CH_3_O), 3.62 (t, 2H, *J*= 6.60 Hz, NCH_2_), 3.61 (t, 2H, *J*= 6.60 Hz, NCH_2_), 2.42–2.29 (m, 20H, 2NCH_2_ and 8NCH_2_ pip), 2.23 (s, 3H, NCH_3_), 2.22 (s, 3H, NCH_3_), 1.71–1.67 (m, 4H, 2CH_2_), 1.59–1.52 (m, 4H, 2CH_2_).

**7-Methoxy-2,4-bis{4-[(3–(4-methylpiperazin-1-yl)propyl)iminomethyl]phenyl}quinoline (7p)**

Yellow oil (98%); ^1^H NMR δ (300 MHz, CDCl_3_) 8.34 (s, 1H, CH=N), 8.31 (s, 1H, CH=N), 8.19 (d, 2H, *J*= 8.20 Hz, H-3′′ and H-5′′), 7.85 (d, 2H, *J*= 8.20 Hz, H-3′ and H-5′), 7.83 (d, 2H, *J*= 8.20 Hz, H-2′′and H-6′′), 7.72 (d, 1H, *J*= 9.25 Hz, H-5), 7.66 (s, 1H, H-3), 7.55 (d, 2H, *J*= 8.20 Hz, H-2′ and H-6′), 7.53 (d, 1H, *J*= 2.60 Hz, H-8), 7.10 (dd,1H, *J*= 9.25 and 2.60 Hz, H-6), 3.95 (s, 3H, CH_3_O), 3.67 (t, 2H, *J*= 6.90 Hz, NCH_2_), 3.64 (t, 2H, *J*= 6.90 Hz, NCH_2_), 2.46–2.33 (m, 20H, 2NCH_2_ and 8NCH_2_ pip), 2.25 (s, 3H, NCH_3_), 2.24 (s, 3H, NCH_3_), 1.93–1.86 (m, 4H, 2CH_2_).

**2,4-Bis{3-[(3-dimethylaminopropyl)iminomethyl]phenyl}quinoline (7q)**

Yellow oil (98%); ^1^H NMR δ (300 MHz, CDCl_3_) 8.53 (dd, 1H, *J*= 1.50 and 1.50 Hz, H-2′), 8.44 (s, 1H, CH=N), 8.41 (s, 1H, CH=N), 8.31 (ddd, 1H, *J*= 7.80, 1.50 and 1.50 Hz, H-6′), 8.26 (dd, 1H, *J*= 8.50 and 1.20 Hz, H-8), 7.94–7.85 (m, 5H, H-2′′, H-4′, H-4′′, H-5 and H-3), 7.76 (ddd,1H, *J*= 8.50, 7.20 and 1.20 Hz, H-7), 7.65–7.57 (m, 3H, H-5′, H-5′′ and H-6′′), 7.51 (ddd, 1H, *J*= 8.50, 7.20 and 1.20 Hz, H-6), 3.72 (t, 4H, *J*= 7.00 Hz, 2NCH_2_), 2.43 (t, 4H, *J*= 7.00 Hz, 2NCH_2_), 2.29 (s, 6H, N(CH_3_)_2_), 2.28 (s, 6H, N(CH_3_)_2_), 1.97–1.89 (m, 4H, 2CH_2_).

**2,4-Bis{3-[(3–(4-methylpiperazin-1-yl)propyl)iminomethyl]phenyl}quinoline (7r)**

Yellow oil (98%); ^1^H NMR δ (300 MHz, CDCl_3_) 8.48 (dd, 1H, *J*= 1.50 and 1.50 Hz, H-2′), 8.37 (s, 1H, CH=N), 8.34 (s, 1H, CH=N), 8.26 (ddd, 1H, *J*= 7.80, 1.50 and 1.50 Hz, H-6′), 8.22 (dd, 1H, *J*= 8.10 and 1.20 Hz, H-8), 7.88–7.78 (m, 5H, H-2′′, H-4′, H-4′′, H-5 and H-3), 7.70 (ddd,1H, *J*= 8.10, 7.20 and 1.50 Hz, H-7), 7.57–7.50 (m, 3H, H-5′, H-5′′ and H-6′′), 7.44 (ddd, 1H, *J*= 8.10, 7.20 and 1.50 Hz, H-6), 3.67–3.60 (m, 4H, 2NCH_2_), 2.56–2.33 (m, 20H, 10NCH_2_), 2.24 (s, 3H, NCH_3_), 2.23 (s, 3H, NCH_3_), 1.94–1.82 (m, 4H, 2CH_2_).

**2,4-Bis{3-[(3-morpholinopropyl)iminomethyl]phenyl}quinoline (7s)**

Yellow oil (99%); ^1^H NMR δ (300 MHz, CDCl_3_) 8.48 (dd, 1H, *J*= 1.50 and 1.50 Hz, H-2′), 8.35 (s, 1H, CH=N), 8.32 (s, 1H, CH=N), 8.25 (ddd, 1H, *J*= 8.10, 1.50 and 1.50 Hz, H-6′), 8.20 (dd, 1H, *J*= 8.10 and 1.20 Hz, H-8), 7.87–7.65 (m, 5H, H-2′′, H-4′, H-4′′, H-5 and H-3), 7.66 (ddd,1H, *J*= 8.10, 6.90 and 1.50 Hz, H-7), 7.56–7.48 (m, 3H, H-5′, H-5′′ and H-6′′), 7.44 (ddd, 1H, *J*= 8.10, 6.90 and 1.50 Hz, H-6), 3.67–3.60 (m, 12H, 2NCH_2_ and 4OCH_2_), 2.40–2.35 (m, 12H, 6NCH_2_), 1.91–1.79 (m, 4H, 2CH_2_).

**2,4-Bis{3-[(2-morpholinoethyl)iminomethyl]phenyl}quinoline (7t)**

Yellow oil (98%); ^1^H NMR δ (300 MHz, CDCl_3_) 8.48 (dd, 1H, *J*= 1.50 and 1.50 Hz, H-2′), 8.36 (s, 1H, CH=N), 8.33 (s, 1H, CH=N), 8.25 (ddd, 1H, *J*= 7.80, 1.50 and 1.50 Hz, H-6′), 8.20 (dd, 1H, *J*= 8.40 and 1.50 Hz, H-8), 7.88–7.76 (m, 5H, H-2′′, H-4′, H-4′′, H-5 and H-3), 7.68 (ddd,1H, *J*= 8.40, 6.90 and 1.50 Hz, H-7), 7.56–7.49 (m, 3H, H-5′, H-5′′ and H-6′′), 7.43 (ddd, 1H, *J*= 8.40, 6.90 and 1.50 Hz, H-6), 3.75 (t, 4H, *J*= 7.20 Hz, 2NCH_2_), 3.68–3.64 (m, 8H, 4OCH_2_), 2.70–2.64 (m, 4H, 2NCH_2_), 2.52–2.47 (m, 8H, 4NCH_2_).

**1,3-Bis{4-[(4-dimethylaminobutyl)iminomethyl]phenyl}isoquinoline (8a)**

Yellow oil (98%); ^1^H NMR δ (300 MHz, CDCl_3_) 8.37 (s, 1H, CH=N), 8.30 (s, 1H, CH=N), 8.25 (d, 2H, *J*= 8.40 Hz, H-3′′ and H-5′′), 8.08 (s, 1H, H-4), 8.06 (dd, 1H, *J*= 8.10 and 1.20 Hz, H-8), 7.91–7.87 (m, 3H, H-5, H-3′ and H-5′), 7.85–7.80 (m, 4H, H-2′′, H-6′′, H-2′, and H-6′), 7.64 (ddd, 1H, *J*= 8.10, 7.20 and 1.20 Hz, H-6), 7.48 (ddd, 1H, *J*= 8.10, 7.20 and 1.20 Hz, H-7), 3.70–3.62 (m, 4H, 2NCH_2_), 2.32 (t, 2H, *J*= 6.90 Hz, NCH_2_), 2.29 (t, 2H, *J*= 6.90 Hz, NCH_2_), 2.22 (s, 6H, N(CH_3_)_2_), 2.21 (s, 6H, N(CH_3_)_2_), 1.78–1.68 (m, 4H, 2CH_2_), 1.62–1.52 (m, 4H, 2CH_2_).

**1,3-Bis{4-[(3-dimethylaminopropyl)iminomethyl]phenyl}isoquinoline (8 b)**

Yellow oil (99%); ^1^H NMR δ (300 MHz, CDCl_3_) 8.38 (s, 1H, CH=N), 8.31 (s, 1H, CH=N), 8.23 (d, 2H, *J*= 8.10 Hz, H-3′′ and H-5′′), 8.10 (s, 1H, H-4), 8.07 (d, 1H, *J*= 8.00 Hz, H-8), 7.93–7.75 (m, 7H, H-5, H-3′, H-5′, H-2′′, H-6′′, H-2′ and H-6′), 7.66 (t, 1H, *J*= 8.00 Hz, H-6), 7.49 (t, 1H, *J*= 8.00 Hz, H-7), 3.68–3.62 (m, 4H, 2NCH_2_), 2.35–2.24 (m, 4H, 2NCH_2_), 2.21 (s, 6H, N(CH_3_)_2_), 2.18 (s, 6H, N(CH_3_)_2_), 1.96–1.78 (m, 4H, 2CH_2_).

**1,3-Bis{4-[(4–(4-methylpiperazin-1-yl)butyl)iminomethyl]phenyl}isoquinoline (8c)**

Yellow oil (80%); ^1^H NMR δ (300 MHz, CDCl_3_) 8.34 (s, 1H, CH=N), 8.27 (s, 1H, CH=N), 8.22 (d, 2H, *J*= 8.40 Hz, H-3′′ and H-5′′), 8.06 (s, 1H, H-4), 8.04 (dd, 1H, *J*= 8.10 and 1.20 Hz, H-8), 8.04 (dd, 1H, *J*= 8.10 and 1.20 Hz, H-5), 7.85 (d, 2H, *J*= 8.40 Hz, H-3′ and H-5′), 7.80 (d, 2H, *J*= 8.40 Hz, H-2′′ and H-6′′), 7.78 (d, 2H, *J*= 8.40 Hz, H-2′ and H-6′), 7.62 (ddd, 1H, *J*= 8.10, 7.80 and 1.20 Hz, H-6), 7.46 (ddd, 1H, *J*= 8.10, 7.80 and 1.20 Hz, H-7), 3.65 (t, 2H, *J*= 6.90 Hz, NCH_2_), 3.60 (t, 2H, *J*= 6.90 Hz, NCH_2_), 2.45–2.35 (m, 20H, 2NCH_2_ and 8 NCH_2_pip.), 2.24 (s, 3H, NCH_3_), 2.23 (s, 3H, NCH_3_), 1.75–1.65 (m, 4H, 2CH_2_), 1.61–1.51 (m, 4H, 2CH_2_).

**1,3-Bis{4-[(3–(4-methylpiperazin-1-yl)propyl)iminomethyl]phenyl}isoquinoline (8d)**

Yellow oil (99%); ^1^H NMR δ (300 MHz, CDCl_3_) 8.36 (s, 1H, CH=N), 8.29 (s, 1H, CH=N), 8.22 (d, 2H, *J*= 8.10 Hz, H-3′′ and H-5′′), 8.07 (s, 1H, H-4), 8.05 (d, 1H, *J*= 8.00 Hz, H-8), 7.88 (d, 1H, *J*= 8.00 Hz, H-5), 7.87 (d, 2H, *J*= 8.10 Hz, H-3′, and H-5′), 7.82 (d, 2H, *J*= 8.10 Hz, H-2′′ and H-6′′), 7.79 (d, 2H, *J*= 8.10 Hz, H-2′ and H-6′), 7.63 (t, 1H, *J*= 8.00 Hz, H-6), 7.47 (t, 1H, *J*= 8.00 Hz, H-7), 3.66 (t, 2H, *J*= 6.90 Hz, NCH_2_), 3.61 (t, 2H, *J*= 6.90 Hz, NCH_2_), 2.46–2.38 (m, 20H, 2NCH_2_ and 8 NCH_2_pip.), 2.25 (s, 3H, NCH_3_), 2.23 (s, 3H, NCH_3_), 1.95–1.83 (m, 4H, 2CH_2_).

**7-Methoxy-1,3-bis{4-[(4-dimethylaminobutyl)iminomethyl]phenyl}isoquinoline (8e)**

Yellow oil (88%); ^1^H NMR δ (300 MHz, CDCl_3_) 8.41 (s, 1H, CH=N), 8.33 (s, 1H, CH=N), 8.23 (d, 2H, *J*= 8.10 Hz, H-3′′ and H-5′′), 8.05 (s, 1H, H-4), 7.94–7.81 (m, 7H, H-5, H-3′, H-5′, H-2′′, H-6′′, H-2′ and H-6′), 7.38 (d, 1H, *J*= 2.40 Hz, H-8), 7.34 (dd, 1H, *J*= 9.00 and 2.40 Hz, H-6), 3.81 (s, 3H, CH_3_O), 3.70 (t, 2H, *J*= 6.90 Hz, NCH_2_), 3.66 (t, 2H, *J*= 6.90 Hz, NCH_2_), 2.35 (t, 2H, *J*= 6.90 Hz, NCH_2_), 2.30 (t, 2H, *J*= 6.90 Hz, NCH_2_), 2.23 (s, 6H, N(CH_3_)_2_), 2.21 (s, 6H, N(CH_3_)_2_), 1.81–1.70 (m, 4H, 2CH_2_), 1.65–1.54 (m, 4H, 2CH_2_).

**7-Methoxy-1,3-bis{4-[(3-dimethylaminopropyl)iminomethyl]phenyl}isoquinoline (8f)**

Orange-yellow oil (91%); ^1^H NMR δ (300 MHz, CDCl_3_) 8.44 (s, 1H, CH=N), 8.36 (s, 1H, CH=N), 8.25 (d, 2H, *J*= 8.40 Hz, H-3′′ and H-5′′), 8.08 (s, 1H, H-4), 7.98–7.88 (m, 6H, H-3′, H-5′, H-2′′, H-6′′, H-2′ and H-6′), 7.84 (d, 1H, *J*= 8.40 Hz, H-5), 7.41 (d, 1H, *J*= 2.40 Hz, H-8), 7.37 (dd, 1H, *J*= 8.40 and 2.40 Hz, H-6), 3.83 (s, 3H, CH_3_O), 3.73 (t, 2H, *J*= 7.20 Hz, NCH_2_), 3.69 (t, 2H, *J*= 7.20 Hz, NCH_2_), 2.42 (t, 2H, *J*= 7.20 Hz, NCH_2_), 2.39 (t, 2H, *J*= 7.20 Hz, NCH_2_), 2.28 (s, 6H, N(CH_3_)_2_), 2.26 (s, 6H, N(CH_3_)_2_), 2.00–1.87 (m, 4H, 2CH_2_).

**7-Methoxy-1,3-bis{4-[(4–(4-methylpiperazin-1-yl)butyl)iminomethyl]phenyl}isoquinoline (8g)**

Yellow oil (98%); ^1^H NMR δ (300 MHz, CDCl_3_) 8.30 (s, 1H, CH=N), 8.22 (s, 1H, CH=N), 8.14 (d, 2H, *J*= 8.40 Hz, H-3′′ and H-5′′), 7.94 (s, 1H, H-4), 7.83 (d, 2H, *J*= 8.40 Hz, H-3′ and H-5′), 7.78 (d, 2H, *J*= 8.40 Hz, H-2′′ and H-6′′), 7.73 (d, 2H, *J*= 8.40 Hz, H-2′ and H-6′), 7.71 (d, 1H, *J*= 9.00 Hz, H-5), 7.28 (d, 1H, *J*= 2.40 Hz, H-8), 7.24 (dd, 1H, *J*= 9.00 and 2.40 Hz, H-6), 3.71 (s, 3H, CH_3_O), 3.58 (t, 2H, *J*= 7.20 Hz, NCH_2_), 3.54 (t, 2H, *J*= 7.20 Hz, NCH_2_), 2.40–2.25 (m, 20H, 2NCH_2_ and 8NCH_2_pip.), 2.20 (s, 3H, NCH_3_), 2.19 (s, 3H, NCH_3_), 1.72–1.63 (m, 4H, 2CH_2_), 1.54–1.47 (m, 4H, 2CH_2_).

**7-Methoxy-1,3-bis{4-[(3–(4-methylpiperazin-1-yl)propyl)iminomethyl]phenyl}isoquinoline (8h)**

Yellow oil (98%); ^1^H NMR δ (300 MHz, CDCl_3_) 8.37 (s, 1H, CH=N), 8.29 (s, 1H, CH=N), 8.20 (d, 2H, *J*= 8.40 Hz, H-3′′ and H-5′′), 8.01 (s, 1H, H-4), 7.90–7.78 (m, 7H, H-3′, H-5′, H-2′′, H-6′′, H-2′, H-6′ and H-5), 7.33 (d, 1H, *J*= 2.40 Hz, H-8), 7.28 (dd, 1H, *J*= 8.40 and 2.40 Hz, H-6), 3.76 (s, 3H, CH_3_O), 3.68–3.61 (m, 4H, 2NCH_2_), 32.47–2.38 (m, 20H, 2NCH_2_ and 8NCH_2_pip.), 2.26 (s, 3H, NCH_3_), 2.25 (s, 3H, NCH_3_), 1.96–1.84 (m, 4H, 2CH_2_).

**6-Methoxy-1,3-bis{4-[(4-dimethylaminobutyl)iminomethyl]phenyl}isoquinoline (8i)**

Pale-orange oil (98%); ^1^H NMR δ (300 MHz, CDCl_3_) 8.35 (s, 1H, CH=N), 8.29 (s, 1H, CH=N), 8.21 (d, 2H, *J*= 8.40 Hz, H-3′′ and H-5′′), 7.98 (s, 1H, H-4), 7.94 (d, 1H, *J*= 9.30 Hz, H-8), 7.87 (d, 2H, *J*= 8.40 Hz,H-3′ and H-5′), 7.79 (d, 4H, *J*= 8.40 Hz, H-2′′, H-6′′, H-2′ and H-6′), 7.14 (d, 1H, *J*= 2.50 Hz, H-5), 7.09 (dd, 1H, *J*= 9.30 and 2.50 Hz, H-7), 3.91 (s, 3H, CH_3_O),3.65 (t, 2H, *J*= 6.80 Hz, NCH_2_), 3.63 (t, 2H, *J*= 6.80 Hz, NCH_2_), 2.46–2.38 (m, 4H, 2NCH_2_), 2.28 (s, 6H, N(CH_3_)_2_), 2.26 (s, 6H, N(CH_3_)_2_), 1.76–1.67 (m, 4H, 2CH_2_), 1.64–1.55 (m, 4H, 2CH_2_).

**6-Methoxy-1,3-bis{4-[(3-dimethylaminopropyl)iminomethyl]phenyl}isoquinoline (8j)**

Pale-yellow oil (98%); ^1^H NMR δ (300 MHz, CDCl_3_) 8.34 (s, 1H, CH=N), 8.28 (s, 1H, CH=N), 8.20 (d, 2H, *J*= 8.40 Hz, H-3′′ and H-5′′), 7.93 (s, 1H, H-4), 7.91 (d, 1H, *J*= 9.00 Hz, H-8), 7.86 (d, 2H, *J*= 8.40 Hz, H-3′ and H-5′), 7.78 (d, 2H, *J*= 8.40 Hz, H-2′′ and H-6′′), 7.76 (d, 2H, *J*= 8.40 Hz, H-2′ and H-6′), 7.09 (d, 1H, *J*= 2.40 Hz, H-5), 7.06 (dd, 1H, *J*= 9.00 and 2.40 Hz, H-7), 3.87 (s, 3H, CH_3_O), 3.66 (t, 2H, *J*= 7.20 Hz, NCH_2_), 3.63 (t, 2H, *J*= 7.20 Hz, NCH_2_), 2.37–2.30 (m, 4H, 2NCH_2_), 2.22 (s, 6H, N(CH_3_)_2_), 2.21 (s, 6H, N(CH_3_)_2_), 1.91–1.85 (m, 4H, 2CH_2_).

**6-Methoxy-1,3-bis{4-[(4–(4-methylpiperazin-1-yl)butyl)iminomethyl]phenyl}isoquinoline (8k)**

Orange-yellow oil (98%); ^1^H NMR δ (300 MHz, CDCl_3_) 8.29 (s, 1H, CH=N), 8.23 (s, 1H, CH=N), 8.17 (d, 2H, *J*= 8.40 Hz, H-3′′ and H-5′′), 7.92 (s, 1H, H-4), 7.88 (d, 1H, *J*= 9.00 Hz, H-8), 7.82 (d, 2H, *J*= 8.40 Hz, H-3′ and H-5′), 7.74 (d, 4H, *J*= 8.40 Hz, H-2′′, H-6′′, H-2′ and H-6′), 7.08 (d, 1H, *J*= 2.40 Hz, H-5), 7.03 (dd, 1H, *J*= 9.00 and 2.40 Hz, H-7), 3.85 (s, 3H, CH_3_O), 3.62–3.54 (m, 4H, 2NCH_2_), 2.48–2.30 (m, 20H, 2NCH_2_ and 8NCH_2_pip.), 2.21 (s, 3H, NCH_3_), 2.19 (s, 3H, NCH_3_), 1.71–1.64 (m, 4H, 2CH_2_), 1.56–1.49 (m, 4H, 2CH_2_).

**6-Methoxy-1,3-bis{4-[(3–(4-methylpiperazin-1-yl)propyl)iminomethyl]phenyl}isoquinoline (8l)**

Yellow oil (95%); ^1^H NMR δ (300 MHz, CDCl_3_) 8.38 (s, 1H, CH=N), 8.33 (s, 1H, CH=N), 8.23 (d, 2H, *J*= 8.40 Hz, H-3′′ and H-5′′), 8.01 (s, 1H, H-4), 7.97 (d, 1H, *J*= 9.20 Hz, H-8), 7.89 (d, 2H, *J*= 8.40 Hz, H-3′ and H-5′), 7.82 (d, 4H, *J*= 8.40 Hz, H-2′′, H-6′′, H-2′ and H-6′), 7.18 (d, 1H, *J*= 2.40 Hz, H-5), 7.11 (dd, 1H, *J*= 9.20 and 2.40 Hz, H-7), 3.96 (s, 3H, CH_3_O),3.69 (t, 2H, *J*= 6.90 Hz, NCH_2_), 3.66 (t, 2H, *J*= 6.90 Hz, NCH_2_), 2.50–2.38 (m, 20H, 2NCH_2_ and 8NCH_2_pip.), 2.27 (s, 3H, NCH_3_), 2.26 (s, 3H, NCH_3_), 1.99–1.90 (m, 4H, 2CH_2_).

**2,4-Bis{4-[(4-dimethylaminobutyl)iminomethyl]phenyl}quinazoline (9a)**

Yellow oil (97%); ^1^H NMR δ (300 MHz, CDCl_3_) 8.72 (d, 2H, *J*= 8.40 Hz, H-3′′ and H-5′′), 8.39 (s, 1H, CH=N), 8.35 (s, 1H, CH=N), 8.13 (dd, 1H, *J*= 8.60 and 1.50 Hz, H-8), 8.07 (dd, 1H, *J*= 8.60 and 1.50 Hz, H-5), 7.96–7.84 (m, 7H, H-3′, H-5′, H-2′′, H-6′′ H-2′, H-6′ and H-7), 7.54 (ddd,1H, *J*= 8.60, 7.20 and 1.50 Hz, H-6), 3.69 (t, 2H, *J*= 7.00 Hz, NCH_2_), 3.67 (t, 2H, *J*= 7.00 Hz, NCH_2_), 2.38–2.31 (m. 4H, 2NCH_2_), 2.26 (s, 6H, N(CH_3_)_2_), 2.24 (s, 6H, N(CH_3_)_2_), 1.82–1.70 (m, 4H, 2CH_2_), 1.64–1.52 (m, 4H, 2CH_2_).

**2,4-Bis{4-[(3-dimethylaminopropyl)iminomethyl]phenyl}quinazoline (9b)**

Yellow oil (97%); ^1^H NMR δ (300 MHz, CDCl_3_) 8.76 (d, 2H, *J*= 8.80 Hz, H-3′′ and H-5′′), 8.44 (s, 1H, CH=N), 8.39 (s, 1H, CH=N), 8.18 (dd, 1H, *J*= 8.60 and 1.50 Hz, H-8), 8.12 (dd, 1H, *J*= 8.60 and 1.50 Hz, H-5), 7.97–7.85 (m, 7H, H-3′, H-5′, H-2′′, H-6′′ H-2′, H-6′ and H-7), 7.58 (ddd,1H, *J*= 8.60, 7.20 and 1.50 Hz, H-6), 3.74–3.70 (m, 4H, 2NCH_2_), 2.43 (t, 2H, *J*= 7.20 Hz, NCH_2_), 2.41 (t, 2H, *J*= 7.20 Hz, NCH_2_), 2.30 (s, 6H, N(CH_3_)_2_), 2.29 (s, 6H, N(CH_3_)_2_), 1.99–1.92 (m, 4H, 2CH_2_).

**2,4-Bis{4-[(2-dimethylaminoethyl)iminomethyl]phenyl}quinazoline (9c)**

Yellow oil (97%); ^1^H NMR δ (300 MHz, CDCl_3_) 8.72 (d, 2H, *J*= 8.40 Hz, H-3′′ and H-5′′), 8.47 (s, 1H, CH=N), 8.42 (s, 1H, CH=N), 8.18 (dd, 1H, *J*= 8.10 and 1.50 Hz, H-8), 8.08 (dd, 1H, *J*= 8.10 and 1.50 Hz, H-5), 7.95–7.88 (m, 7H, H-3′, H-5′, H-2′′, H-6′′ H-2′, H-6′ and H-7), 7.58 (ddd,1H, *J*= 8.10, 6.90 and 1.50 Hz, H-6), 3.84 (t, 2H, *J*= 6.90 Hz, NCH_2_), 3.82 (t, 2H, *J*= 6.90 Hz, NCH_2_), 2.73 (t, 2H, *J*= 6.90 Hz, NCH_2_), 2.71 (t, 2H, *J*= 6.90 Hz, NCH_2_), 2.37 (s, 6H, N(CH_3_)_2_), 2.35 (s, 6H, N(CH_3_)_2_).

**2,4-Bis{4-[(4–(4-methylpiperazin-1-yl)butyl)iminomethyl]phenyl}quinazoline (9d)**

Yellow oil (98%); ^1^H NMR δ (300 MHz, CDCl_3_) 8.74 (d, 2H, *J*= 8.40 Hz, H-3′′ and H-5′′), 8.41 (s, 1H, CH=N), 8.37 (s, 1H, CH=N), 8.18 (dd, 1H, *J*= 8.10 and 1.20 Hz, H-8), 8.18 (dd, 1H, *J*= 8.10 and 1.20 Hz, H-5), 7.96–7.86 (m, 7H, H-3′, H-5′, H-2′′, H-6′′ H-2′, H-6′ and H-7), 7.60 (ddd,1H, *J*= 8.10, 6.90 and 1.20 Hz, H-6), 3.72–3.66 (m, 4H, 2NCH_2_), 2.65–2.38 (m, 20H, 2NCH_2_ and 8 NCH_2_pip.), 2.29 (s, 3H, NCH_3_), 2.28(s, 3H, NCH_3_), 1.83–1.71 (m, 4H, 2CH_2_), 1.63–1.58 (m, 4H, 2CH_2_).

**2,4-Bis{4-[(3–(4-methylpiperazin-1-yl)propyl)iminomethyl]phenyl}quinazoline (9e)**

Yellow oil (98%); ^1^H NMR δ (300 MHz, CDCl_3_) 8.75 (d, 2H, *J*= 8.40 Hz, H-3′′ and H-5′′), 8.43 (s, 1H, CH=N), 8.38 (s, 1H, CH=N), 8.17 (dd, 1H, *J*= 8.10 and 1.20 Hz, H-8), 8.13 (dd, 1H, *J*= 8.10 and 1.20 Hz, H-5), 7.96–7.86 (m, 7H, H-3′, H-5′, H-2′′, H-6′′ H-2′, H-6′ and H-7), 7.58 (ddd,1H, *J*= 8.10, 6.90 and 1.20 Hz, H-6), 3.76–3.67 (m, 4H, 2NCH_2_), 2.65–2.41 (m, 20H, 2NCH_2_ and 8 NCH_2_pip.), 2.31 (s, 3H, NCH_3_), 2.30 (s, 3H, NCH_3_), 1.99–1.91 (m, 4H, 2CH_2_).

**2,4-Bis{4-[(3-morpholinopropyl)iminomethyl]phenyl}quinazoline (9f)**

Yellow oil (93%); ^1^H NMR δ (300 MHz, CDCl_3_) 8.67 (d, 2H, *J*= 8.40 Hz, H-3′′ and H-5′′), 8.32 (s, 1H, CH=N), 8.28 (s, 1H, CH=N), 8.06 (dd, 1H, *J*= 8.10 and 1.20 Hz, H-8), 7.99 (dd, 1H, *J*= 8.10 and 1.20 Hz, H-5), 7.89–7.77 (m, 7H, H-3′, H-5′, H-2′′, H-6′′, H-2′, H-6′ and H-7), 7.46 (ddd, 1H, *J*= 8.10, 6.90 and 1.20 Hz, H-6), 3.67–3.57 (m, 12H, 2NCH_2_ and 4 OCH_2_), 2.40–2.32 (m, 12H, 6NCH_2_), 1.92–1.81 (m, 4H, 2CH_2_).

**2,4-Bis{4-[(2-morpholinoethyl)iminomethyl]phenyl}quinazoline (9g)**

Yellow oil (88%); ^1^H NMR δ (300 MHz, CDCl_3_) 8.76 (d, 2H, *J*= 8.40 Hz, H-3′′ and H-5′′), 8.45 (s, 1H, CH=N), 8.41 (s, 1H, CH=N), 8.17 (dd, 1H, *J*= 8.10 and 1.20 Hz, H-8), 8.12 (dd, 1H, *J*= 8.10 and 1.20 Hz, H-5), 7.97–7.75 (m, 7H, H-3′, H-5′, H-2′′, H-6′′, H-2′, H-6′ and H-7), 7.60 (ddd, 1H, *J*= 8.10, 6.90 and 1.20 Hz, H-6), 3.87 (t, 2H, *J*= 6.90 Hz, NCH_2_), 3.84 (t, 2H, *J*= 6.90 Hz, NCH_2_), 3.78–3.71 (m, 8H, 4OCH_2_), 2.84–2.74 (m, 4H, 2NCH_2_), 2.61–2.57 (m, 8H, 4NCH_2_).

**6-Methoxy-2,4-bis{4-[(4-dimethylaminobutyl)iminomethyl]phenyl}quinazoline (9h)**

Yellow oil (98%); ^1^H NMR δ (300 MHz, CDCl_3_) 8.64 (d, 2H, *J*= 8.25 Hz, H-3′′ and H-5′′), 8.35 (s, 1H, CH=N), 8.30 (s, 1H, CH=N), 7.99 (s, 1H, *J*= 9.30 Hz, H-8), 7.90–7.88 (m, 4H, H-3′ and H-5′, H-2′′ and H-6′′), 7.80 (d, 2H, *J*= 8.25 Hz, H-2′ and H-6′), 7.46 (dd, 1H, *J*= 9.30 and 2.70 Hz, H-7), 7.25 (d, 1H, *J*= 2.70 Hz, H-5), 3.76 (s, 3H, CH_3_O and 2NCH_2_), 3.65 (t, 2H, *J*= 7.10 Hz, NCH_2_), 3.62 (t, 2H, *J*= 7.10 Hz, NCH_2_), 2.28 (t, 2H, *J*= 7.10 Hz, NCH_2_), 2.27 (t, 2H, *J*= 7.10 Hz, NCH_2_), 2.19 (s, 6H, N(CH_3_)_2_), 2.18 (s, 6H, N(CH_3_)_2_), 1.76–1.68 (m, 4H, 2CH_2_), 1.57–1.50 (m, 4H, 2CH_2_).

**6-Methoxy-2,4-bis{4-[(3-dimethylaminopropyl)iminomethyl]phenyl}quinazoline (9i)**

Yellow oil (98%); ^1^H NMR δ (300 MHz, CDCl_3_) 8.66 (d, 2H, *J*= 8.25 Hz, H-3′′ and H-5′′), 8.39 (s, 1H, CH=N), 8.33 (s, 1H, CH=N), 8.00 (d, 1H, *J*= 9.15 Hz, H-8), 7.92–7.90 (m, 4H, H-3′ and H-5′, H-2′′ and H-6′′), 7.82 (d, 2H, *J*= 8.25 Hz, H-2′ and H-6′), 7.48 (dd,1H, *J*= 9.15 and 2.40 Hz, H-7), 7.28 (d, 1H, *J*= 2.40 Hz, H-5), 3.77 (s, 3H, CH_3_O), 3.69–3.62 (m, 4H, 2NCH_2_), 2.39–2.32 (m, 4H, 2NCH_2_), 2.23 (s, 6H, N(CH_3_)_2_), 2.22 (s, 6H, N(CH_3_)_2_), 1.95–1.83 (m, 4H, 2CH_2_).

**6-Methoxy-2,4-bis{4-[(2-dimethylaminoethyl)iminomethyl]phenyl}quinazoline (9j)**

Yellow oil (98%); ^1^H NMR δ (300 MHz, CDCl_3_) 8.66 (d, 2H, *J*= 8.40 Hz, H-3′′ and H-5′′), 8.36 (s, 1H, CH=N), 8.30 (s, 1H, CH=N), 7.95–7.76 (m, 7H, H-8, H-3′ and H-5′, H-2′′ and H-6′′, H-2 and H-6′), 7.41 (dd, 1H, *J*= 9.10 and 2.80 Hz, H-7), 7.20 (d, 1H, *J*= 2.80 Hz, H-5), 3.77–3.70 (m, 7H, CH_3_O and 2NCH_2_), 2.64 (t, 2H, *J*= 6.60 Hz, NCH_2_), 2.62 (t, 2H, *J*= 6.60 Hz, NCH_2_), 2.27 (s, 6H, N(CH_3_)_2_), 2.26 (s, 6H, N(CH_3_)_2_).

**6-Methoxy-2,4-bis{4-[(3–(4-methylpiperazin-1-yl)propyl)iminomethyl]phenyl}quinazoline (9k)**

Yellow oil (97%); ^1^H NMR δ (300 MHz, CDCl_3_) 8.61 (d, 2H, *J*= 8.40 Hz, H-3′′ and H-5′′), 8.33 (s, 1H, CH=N), 8.27 (s, 1H, CH=N), 7.96 (d, 1H, *J*= 9.15 Hz, H-8), 7.89–7.83 (m, 4H, H-3′ and H-5′, H-2′′ and H-6′′), 7.77 (d, 2H, *J*= 8.40 Hz, H-2′ and H-6′), 7.43 (dd,1H, *J*= 9.15 and 2.70 Hz, H-7), 7.22 (d, 1H, *J*= 2.70 Hz, H-5), 3.72 (s, 3H, CH_3_O), 3.64 (t, 2H, *J*= 6.60 Hz, NCH_2_), 3.60 (t, 2H, *J*= 6.60 Hz, NCH_2_), 2.50–2.30 (m, 20H, 2NCH_2_ and 8NCH_2_ pip.), 2.21 (s, 3H, NCH_3_), 2.20 (s, 3H, NCH_3_), 1.90–1.82 (m, 4H, 2CH_2_).

**6-Methoxy-2,4-bis{4-[(2–(4-methylpiperazin-1-yl)ethyl)iminomethyl]phenyl}quinazoline (9l)**

Yellow oil (98%); ^1^H NMR δ (300 MHz, CDCl_3_) 8.64 (d, 2H, *J*= 8.40 Hz, H-3′′ and H-5′′), 8.38 (s, 1H, CH=N), 8.32 (s, 1H, CH=N), 8.00 (d, 1H, *J*= 9.15 Hz, H-8), 7.90 (S, 4H, H-3′ and H-5′, H-2′′ and H-6′′), 7.80 (d, 2H, *J*= 8.40 Hz, H-2′ and H-6′), 7.48 (dd,1H, *J*= 9.15 and 2.70 Hz, H-7), 7.27 (d, 1H, *J*= 2.70 Hz, H-5), 3.82–3.73 (m, 7H, CH_3_O and 2NCH_2_), 2.73 (t, 2H, *J*= 7.20 Hz, NCH_2_), 2.71 (t, 2H, *J*= 7.20 Hz, NCH_2_), 2.58–2.38 (m, 16H, 8NCH_2_ pip.), 2.25 (s, 3H, NCH_3_), 2.24 (s, 3H, NCH_3_).

**7-Methoxy-2,4-bis{4-[(4-dimethylaminobutyl)iminomethyl]phenyl}quinazoline (9m)**

Yellow oil (98%); ^1^H NMR δ (300 MHz, CDCl_3_) 8.72 (d, 2H, *J*= 8.40 Hz, H-3′′ and H-5′′), 8.41 (s, 1H, CH=N), 8.37 (s, 1H, CH=N), 8.00–7.85 (m, 7H, H-5, H3′ and H5′, H-2′′ and H-6′′, H-2′ and H-6′), 7.45 (d, 1H, *J*= 2.70 Hz, H-8), 7.18 (dd,1H, *J*= 9.30 and 2.70 Hz, H-6), 4.02 (s, 3H, CH_3_O), 3.71 (t, 2H, *J*= 6.90 Hz, NCH_2_), 3.62 (t, 2H, *J*= 6.90 Hz, NCH_2_), 2.35 (t, 2H, *J*= 6.90 Hz, NCH_2_), 2.32 (t, 2H, *J*= 6.90 Hz, NCH_2_), 2.25 (s, 6H, N(CH_3_)_2_), 2.23 (s, 6H, N(CH_3_)_2_), 1.81–1.71 (m, 4H, 2CH_2_), 1.64–1,54 (m, 4H, 2CH_2_).

**7-Methoxy-2,4-bis{4-[(3-dimethylaminopropyl)iminomethyl]phenyl}quinazoline (9n)**

Yellow oil (98%); ^1^H NMR δ (300 MHz, CDCl_3_) 8.73 (d, 2H, *J*= 8.40 Hz, H-3′′ and H-5′′), 8.44 (s, 1H, CH=N), 8.40 (s, 1H, CH=N), 8.00 (d, 1H, *J*= 9.25 Hz, H-5), 7.97 (d, 2H, *J*= 8.40 Hz, H-3′ and H-5′), 7.92 (d, 2H, *J*= 8.40 Hz, H-2′′ and H-6′′), 7.88 (d, 2H, *J*= 8.40 Hz, H-2′ and H-6′), 7.46 (d, 1H, *J*= 2.50 Hz, H-8), 7.19 (dd,1H, *J*= 9.25 and 2.50 Hz, H-6), 4.04 (s, 3H, CH_3_O), 3.74 (t, 2H, *J*= 7.10 Hz, NCH_2_), 3.71 (t, 2H, *J*= 7.10 Hz, NCH_2_), 2.40 (t, 2H, *J*= 7.10 Hz, NCH_2_), 2.39 (t, 2H, *J*= 7.10 Hz, NCH_2_), 2.28 (s, 6H, N(CH_3_)_2_), 2.27 (s, 6H, N(CH_3_)_2_),1.96–1.83 (m, 4H, 2CH_2_).

**7-Methoxy-2,4-bis{4-[(2-dimethylaminoethyl)iminomethyl]phenyl}quinazoline (9o)**

Yellow oil (98%); ^1^H NMR δ (300 MHz, CDCl_3_) 8.69 (d, 2H, *J*= 8.10 Hz, H-3′′ and H-5′′), 8.42 (s, 1H, CH=N), 8.38 (s, 1H, CH=N), 7.95–7.84 (m, 7H, H-5, H3′ and H5′, H-2′′ and H-6′′, H-2′ and H-6′), 7.41 (d, 1H, *J*= 2.40 Hz, H-8), 7.14 (dd,1H, *J*= 9.30 and 2.40 Hz, H-6), 3.98 (s, 3H, CH_3_O), 3.80 (t, 2H, *J*= 7.00 Hz, NCH_2_), 3.78 (t, 2H, *J*= 7.00 Hz, NCH_2_), 2.68 (t, 2H, *J*= 7.00 Hz, NCH_2_), 2.67 (t, 2H, *J*= 7.00 Hz, NCH_2_), 2.33 (s, 6H, N(CH_3_)_2_), 2.32 (s, 6H, N(CH_3_)_2_).

**7-Methoxy-2,4-bis{4-[(4–(4-methylpiperazin-1-yl)butyl)iminomethyl]phenyl}quinazoline (9p)**

Yellow oil (98%); ^1^H NMR δ (300 MHz, CDCl_3_) 8.59 (d, 2H, *J*= 8.40 Hz, H-3′′ and H-5′′), 8.26 (s, 1H, CH=N), 8.22 (s, 1H, CH=N), 7.84–7.72 (m, 7H, H-5, H3′ and H5′, H-2′′ and H-6′′, H-2′ and H-6′), 7.28 (d, 1H, *J*= 2.40 Hz, H-8), 7.12 (dd,1H, *J*= 9.30 and 2.40 Hz, H-6), 3.86 (s, 3H, CH_3_O), 3.57 (t, 2H, *J*= 6.60 Hz, NCH_2_), 3.53 (t, 2H, *J*= 6.60 Hz, NCH_2_), 2.50–2.20 (m, 20H, 2NCH_2_ and 8NCH_2_ pip.), 2.17 (s, 3H, NCH_3_), 2.16 (s, 3H, NCH_3_), 1.66–1.62 (m, 4H, 2CH_2_), 1.51–1.47 (2CH_2_).

**7-Methoxy-2,4-bis{4-[(3–(4-methylpiperazin-1-yl)propyl)iminomethyl]phenyl}quinazoline(9q)**

Yellow oil (98%); ^1^H NMR δ (300 MHz, CDCl_3_) 8.70 (d, 2H, *J*= 8.40 Hz, H-3′′ and H-5′′), 8.40 (s, 1H, CH=N), 8.36 (s, 1H, CH=N), 7.96 (d, 1H, *J*= 9.30 Hz, H-5), 7.93 (d, 2H, *J*= 8.40 Hz, H-3′ and H-5′), 7.88 (d, 2H, *J*= 8.40 Hz, H-2′′ and H-6′′), 7.85 (d, 2H, *J*= 8.40 Hz, H-2′ and H-6′), 7.43 (d, 1H, *J*= 2.70 Hz, H-8), 7.16 (dd,1H, *J*= 9.30 and 2.70 Hz, H-6), 4.00 (s, 3H, CH_3_O), 3.73–3.65 (m, 4H, 2NCH_2_), 2.49–2,42 (m, 20H, 2NCH_2_ and 8NCH_2_ pip.), 2.28 (s, 3H, NCH_3_), 2.27 (s, 3H, NCH_3_), 1.99–1.87 (m, 4H, 2CH_2_).

**7-Methoxy-2,4-bis{4-[(2–(4-methylpiperazin-1-yl)ethyl)iminomethyl]phenyl}quinazoline (9r)**

Orange oil (98%); ^1^H NMR δ (300 MHz, CDCl_3_) 8.68 (d, 2H, *J*= 8.40 Hz, H-3′′ and H-5′′), 8.39 (s, 1H, CH=N), 8.35 (s, 1H, CH=N), 7.94–7.82 (m, 7H, H-5, H3′ and H5′, H-2′′ and H-6′′, H-2′ and H-6′), 7.41 (d, 1H, *J*= 2.60 Hz, H-8), 7.14 (dd,1H, *J*= 9.25 and 2.60 Hz, H-6), 3.98 (s, 3H, CH_3_O), 3.82 (t, 2H, *J*= 7.20 Hz, NCH_2_), 3.79 (t, 2H, *J*= 7.20 Hz, NCH_2_), 2.76 (t, 2H, *J*= 7.20 Hz, NCH_2_), 2.73 (t, 2H, *J*= 7.20 Hz, NCH_2_), 2.65–2,30 (m, 16H, 8NCH_2_ pip), 2.28 (s, 3H, NCH_3_), 2.27 (s, 3H, NCH_3_).

**2,4-Bis{3-[(3-dimethylaminopropyl)iminomethyl]phenyl}quinazoline (9s)**

Pale-yellow oil (98%); ^1^H NMR δ (300 MHz, CDCl_3_) 8.94 (dd, 1H, *J*= 1.40 and 1.40 Hz, H-2′), 8.77 (ddd, 1H, *J*= 7.80, 1.40 and 1.40 Hz, H-6′), 8.48 (s, 1H, CH=N), 8.45 (s, 1H, CH=N), 8.21–8.17 (m, 2H, H-2′′ and H-8), 8.11 (dd, 1H, *J*= 8.10 and 1.20 Hz, H-5), 8.03–7.90 (m, 4H, H-6′′, H-4′, H-4′′ and H-7), 7.67 (dd,1H, *J*= 7.80 and 7.80 Hz, H-5′′), 7.62–7.56 (m, 2H, H-5′ and H-6), 3.74–3.69 (m, 4H, 2NCH_2_), 2.43–2.33 (m, 4H, 2NCH_2_), 2.27 (s, 6H, N(CH_3_)_2_), 2.26 (s, 6H, N(CH_3_)_2_), 1.98–1.87 (m, 4H, 2CH_2_).

**2,4-Bis{3-[(3–(4-methylpiperazin-1-yl)propyl)iminomethyl]phenyl}quinazoline(9t)**

Yellow oil (98%); ^1^H NMR δ (300 MHz, CDCl_3_) 8.93 (dd, 1H, *J*= 1.35 and 1.35 Hz, H-2′), 8.76 (ddd, 1H, *J*= 7.80, 1.35 and 1.35 Hz, H-6′), 8.47 (s, 1H, CH=N), 8.44 (s, 1H, CH=N), 8.21–8.18 (m, 2H, H-2′′ and H-8), 8.11 (dd, 1H, *J*= 8.40 and 1.50 Hz, H-5), 8.02–7.90 (m, 4H, H-6′′, H-4′, H-4′′ and H-7), 7.67 (dd,1H, *J*= 7.80 and 7.80 Hz, H-5′′), 7.62–7.56 (m, 2H, H-5′ and H-6), 3.76–3.68 (m, 4H, 2NCH_2_), 2.50–2.38 (m, 20H, 2NCH_2_ and 8NCH_2_pip.), 2.30 (s, 3H, NCH_3_), 2.29 (s, 3H, NCH_3_), 1.98–1.90 (m, 4H, 2CH_2_).

**2,4-Bis{3-[(3-morpholinopropyl)iminomethyl]phenyl}quinazoline (9u)**

Yellow oil (98%); ^1^H NMR δ (300 MHz, CDCl_3_) 8.93 (dd, 1H, *J*= 1.50 and 1.50 Hz, H-2′), 8.76 (ddd, 1H, *J*= 7.80, 1.50 and 1.50 Hz, H-6′), 8.46 (s, 1H, CH=N), 8.43 (s, 1H, CH=N), 8.20–8.16 (m, 2H, H-2′′ and H-8), 8.09 (dd, 1H, *J*= 8.60 and 1.20 Hz, H-5), 8.00–7.91 (m, 4H, H-6′′, H-4′, H-4′′ and H-7), 7.66 (dd,1H, *J*= 7.80 and 7.80 Hz, H-5′′), 7.61–7.55 (m, 2H, H-5′ and H-6), 3.75–3.68 (m, 12H, 2NCH_2_ and 4OCH_2_), 2.48–2.37 (m, 12H, 2NCH_2_ and 4NCH_2_morph.), 1.96–1.87 (m, 4H, 2CH_2_).

**2,4-Bis{3-[(2-morpholinoethyl)iminomethyl]phenyl}quinazoline (9v)**

Yellow oil (98%); ^1^H NMR δ (300 MHz, CDCl_3_) 8.95 (dd, 1H, *J*= 1.35 and 1.35 Hz, H-2′), 8.76 (ddd, 1H, *J*= 7.80, 1.35 and 1.35 Hz, H-6′), 8.49 (s, 1H, CH=N), 8.46 (s, 1H, CH=N), 8.21–8.18 (m, 2H, H-2′′ and H-8), 8.10 (dd, 1H, *J*= 8.10 and 1.20 Hz, H-5), 8.02–7.91 (m, 4H, H-6′′, H-4′, H-4′′ and H-7), 7.68 (dd,1H, *J*= 7.80 and 7.80 Hz, H-5′′), 7.63–7.57 (m, 2H, H-5′ and H-6), 3.85 (t, 4H, *J*= 6.90 Hz, NCH_2_), 3.77–3.72 (m, 8H, 4OCH_2_), 2.79–2.73 (m, 4H, 2NCH_2_), 2.61–2.57–1.87 (m, 8H, 4NCH_2_).

### General procedure for 2,4-bis[(substituted-aminomethyl)phenyl]quinolines (1a-t), 1,3-bis[(substituted-aminomethyl)phenyl]isoquinolines (2a–l), and 2,4-bis[(substituted-aminomethyl)phenyl]quinazolines (3a–v)

To a solution of compound **7–9** (0.4 mmol) in methanol (10 ml) was added portion-wise at 0 °C sodium borohydride (3.2 mmol, 8 eq.). The reaction mixture was then stirred at room temperature for 1 h and subsequently heated under reflux for 1 h. Then it was evaporated to dryness under reduced pressure. After cooling, the residue was triturated in water and extracted with dichloromethane (40 ml). The organic layer was separated, dried over sodium sulphate and activated charcoal and evaporated to dryness. Oils were used without further purification to give compounds **1–3**.

**2,4-Bis{4-[(4-dimethylaminobutyl)aminomethyl]phenyl}quinoline (1a)**

Yellow oil (89%); ^1^H NMR δ (300 MHz, CDCl_3_) 8.23 (dd, 1H, *J*= 8.10 and 1.50 Hz, H-8), 8.15 (d, 2H, *J*= 8.10 Hz, H-2′ and H-6′), 7.91 (dd, 1H, *J*= 8.10 and 1.50 Hz, H-5), 7.80 (s, 1H, H-3), 7.72 (ddd,1H, *J*= 8.10, 6.70 and 1.50 Hz, H-7), 7.52–7.43 (m, 7H, H-3′, H-5′, H-2′′, H-6′′, H-3′′, H-5′′ and H-6), 3.91 (s, 2H, NCH_2_), 3.87 (s, 2H, NCH_2_),2.73 (t, 2H, *J*= 6.70 Hz, NCH_2_), 2.67 (t, 2H, *J*= 6.70 Hz, NCH_2_), 2.29 (t, 2H, *J*= 6.70 Hz, NCH_2_), 2.26 (t, 2H, *J* = 6.70 Hz, NCH_2_), 2.22 (s, 6H, N(CH_3_)_2_), 2.20 (s, 6H, N(CH_3_)_2_), 1.58–1.52 (m, 8H, 4CH_2_).13C NMR δ (75 MHz, CDCl_3_) 158.0 (C-2), 150.3 (C-4), 150.2 (C-8a), 143.1 (C-1′′), 142.2 (C-1′), 139.7 (C-4′′), 138.4 (C-4′), 131.4 (C-7), 131.0(C-3′′ and C-5′′), 130.9 (C-8), 130.0 (C-3′ and C-5′), 129.7(C-2′′ and C-6′′), 129.0 (C-2′ and C-6′), 127.6 (C-5),127.2 (C-4a), 127.0 (C-6), 120.6 (C-3), 61.0 (NCH_2_), 55.0 (NCH_2_), 54.8 (NCH_2_), 50.8 (NCH_2_), 50.6 (NCH_2_), 46.8 (2N(CH_3_)_2_), 29.4 (2CH_2_), 26.9 (2CH_2_); MALDI-TOF MS *m/z* [M + H]^+^ Calc for C_35_H_48_N_5_: 538.391, Found: 538.250.

**2,4-Bis{4-[(3-dimethylaminopropyl)aminomethyl]phenyl}quinoline (1b)**

Pale-yellow oil (97%); ^1^H NMR δ (300 MHz, CDCl_3_) 8.22 (dd, 1H, *J*= 8.40 and 1.50 Hz, H-8), 8.15 (d, 2H, *J*= 8.40 Hz, H-2′ and H-6′), 7.91 (dd, 1H, *J*= 8.40 and 1.50 Hz, H-5), 7.80 (s, 1H, H-3), 7.71 (ddd,1H, *J*= 8.40, 6.90 and 1.50 Hz, H-7), 7.55–7.42 (m, 7H, H-3′, H-5′, H-2′′, H-6′′, H-3′′, H-5′′ and H-6), 3.91 (s, 2H, NCH_2_), 3.88 (s, 2H, NCH_2_), 2.76 (t, 2H, *J*= 6.90 Hz, NCH_2_), 2.71 (t, 2H, *J*= 6.90 Hz, NCH_2_), 2.37 (t, 2H, *J*= 6.90 Hz, NCH_2_), 2.33 (t, 2H, *J* = 6.90 Hz, NCH_2_), 2.24 (s, 6H, N(CH_3_)_2_), 2.22 (s, 6H, N(CH_3_)_2_), 1.80–1.68 (m, 4H, 2CH_2_). 13C NMR δ (75 MHz, CDCl_3_) 158.0 (C-2), 150.3 (C-4), 150.2 (C-8a), 142.6 (C-1′′), 141.9 (C-1′), 139.9 (C-4′′), 138.4 (C-4′), 131.5 (C-7), 131.0 (C-3′′ and C-5′′), 130.9 (C-8), 130.0 (C-3′ and C-5′), 129.7 (C-2′′ and C-6′′), 129.0 (C-2′ and C-6′), 127.6 (C-5), 127.1(C-4a), 127.0 (C-6), 120.6 (C-3), 59.6 (NCH_2_), 55.0 (NCH_2_), 54.9 (NCH_2_), 49.5 (NCH_2_), 49.2 (NCH_2_), 46.9 (2N(CH_3_)_2_), 29.3 (CH_2_), 29.0 (CH_2_); MALDI-TOF MS m/z [M + H]^+^ Calc for C_33_H_44_N_5_: 510.360, Found: 510.384.

**2,4-Bis{4-[(2-dimethylaminoethyl)aminomethyl]phenyl}quinoline (1c)**

Yellow oil (76%); ^1^H NMR δ (300 MHz, CDCl_3_) 8.23 (dd, 1H, *J*= 8.10 and 1.20 Hz, H-8), 8.16 (d, 2H, *J*= 8.40 Hz, H-2′ and H-6′), 7.91 (dd, 1H, *J*= 8.10 and 1.20 Hz, H-5), 7.80 (s, 1H, H-3), 7.72 (ddd,1H, *J*= 8.10, 6.90 and 1.20 Hz, H-7), 7.52–7.42 (m, 7H, H-3′, H-5′, H-2′′, H-6′′, H-3′′, H-5′′ and H-6), 3.94 (s, 2H, NCH_2_), 3.90 (s, 2H, NCH_2_), 2.79 (t, 2H, *J*= 6.30 Hz, NCH_2_), 2.72 (t, 2H, *J*= 6.30 Hz, NCH_2_), 2.50 (t, 2H, *J*= 6.30 Hz, NCH_2_), 2.46 (t, 2H, *J*= 6.30 Hz, NCH_2_), 2.25 (s, 6H, N(CH_3_)_2_), 2.22 (s, 6H, N(CH_3_)_2_). 13C NMR δ (75 MHz, CDCl_3_) 156.7 (C-2), 149.0 (C-4), 148.8 (C-8a), 141.6 (C-1′′), 140.7 (C-1′), 138.3 (C-4′′), 137.0 (C-4′), 130.0 (C-7), 129.6 (C-3′′ and C-5′′), 129.7 (C-8), 128.6 (C-3′ and C-5′), 128.4 (C-2′′ and C-6′′), 127.6 (C-2′ and C-6′), 126.2 (C-5), 125.8 (C-4a), 125.6 (C-6), 119.2 (C-3), 59.0 (NCH_2_), 53.7 (NCH_2_), 53.6 (NCH_2_), 46.7 (NCH_2_), 46.4 (NCH_2_), 45.5 (2 N(CH_3_)_2_); MALDI-TOF MS *m/z* [M + H]^+^ Calc for C_31_H_40_N_5_: 482.328, Found: 482.584.

**2,4-Bis{4-[(4–(4-methylpiperazin-1-yl)butyl)aminomethyl]phenyl}quinoline (1d)**

Yellow oil (59%); ^1^H NMR δ (300 MHz, CDCl_3_) 8.21 (dd, 1H, *J*= 8.40 and 1.20 Hz, H-8), 8.15 (d, 2H, *J*= 8.40 Hz, H-3′′ and H-5′′), 7.91 (dd, 1H, *J*= 8.40 and 1.20 Hz, H-5), 7.80 (s, 1H, H-3), 7.72 (ddd,1H, *J*= 8.40, 6.60 and 1.20 Hz, H-7), 7.55–7.43 (m, 7H, H-3′, H-5′, H-2′′, H-6′′, H-2′, H-6′ and H-6), 3.90 (s, 2H, NCH_2_), 3.87 (s, 2H, NCH_2_), 2.73 (t, 2H, *J*= 6.90 Hz, NCH_2_), 2.66 (t, 2H, *J*= 6.90 Hz, NCH_2_), 2.55–2.32 (m. 20H, 2NCH_2_ and 8 NCH_2_pip.), 2.28 (s, 3H, NCH_3_), 2.27 (s, 3H, NCH_3_), 1.61–1.51 (m, 8H, 4CH_2_). 13C NMR δ (75 MHz, CDCl_3_) 158.1 (C-2), 150.3 (C-4), 150.2 (C-8a), 143.1 (C-1′′), 142.2 (C-1′), 139.7 (C-4′′), 138.4 (C-4′), 131.4 (C-7), 131.0 (C-3′′ and C-5′′), 130.9 (C-8), 129.9 (C-3′ and C-5′), 129.7 (C-2′′ and C-6′′), 129.0 (C-2′ and C-6′), 127.6 (C-5), 127.2 (C-4a), 127.0 (C-6), 120.6 (C-3), 59.9 (NCH_2_), 56.5 (NCH_2_), 55.1 (NCH_2_), 54.6 (NCH_2_), 50.8 (NCH_2_), 47.4 (2NCH_3_), 29.5 (2CH_2_), 26.1 (2CH_2_); MALDI-TOF MS *m/z* [M + H]^+^ Calc for C_41_H_58_N_7_: 648.475, Found: 648.451.

**2,4-Bis{4-[(3–(4-methylpiperazin-1-yl)propyl)aminomethyl]phenyl}quinoline (1e)**

Yellow oil (78%); ^1^H NMR δ (300 MHz, CDCl_3_) 8.21(dd, 1H, *J*= 8.40 and 1.50 Hz, H-8), 8.15 (d, 2H, *J*= 8.10 Hz, H-3′′ and H-5′′), 7.88(dd, 1H, *J*= 8.40 and 1.50 Hz, H-5), 7.79 (s, 1H, H-3), 7.71 (ddd,1H, *J*= 8.40, 6.90 and 1.50 Hz, H-7), 7.54–7.41 (m, 7H, H-3′, H-5′, H-2′′, H-6′′, H-2′, H-6′ and H-6), 3.90 (s, 2H, NCH_2_), 3.87 (s, 2H, NCH_2_),) , 2.76 (t, 2H, *J*= 6.90 Hz, NCH_2_), 2.70 (t, 2H, *J*= 6.90 Hz, NCH_2_), 2.48–2.37 (m. 20H, 2NCH_2_ and 8NCH_2_pip.), 2.26 (s, 3H, NCH_3_), 2.25 (s, 3H, NCH_3_), 1.81–1.68 (m, 4H, 2CH_2_). 13C NMR δ (75 MHz, CDCl_3_) 158.0 (C-2), 150.3 (C-4), 150.2 (C-8a), 142.7 (C-1′′), 142.0 (C-1′), 139.8 (C-4′′), 138.4 (C-4′), 131.4 (C-7), 131.0 (C-3′′ and C-5′′), 130.9 (C-8), 130.0 (C-3′ and C-5′), 129.7 (C-2′′ and C-6′′), 129.0 (C-2′ and C-6′), 127.6 (C-5), 127.1 (C-4a), 127.0 (C-6), 120.6 (C-3), 58.4 (NCH_2_), 56.5 (NCH_2_), 55.0 (NCH_2_), 54.9 (NCH_2_), 54.6 (NCH_2_), 49.7 (NCH_2_), 49.5 (NCH_2_), 47.4 (2NCH_3_), 28.2 (CH_2_), 28.0 (CH_2_); MALDI-TOF MS *m/z* [M + H]^+^ Calc for C_39_H_54_N_7_: 620.444, Found: 620.560.

**2,4-Bis{4-[(2–(4-methylpiperazin-1-yl)ethyl)aminomethyl]phenyl}quinoline (1f)**

Orange oil (65%); ^1^H NMR δ (300 MHz, CDCl_3_) 8.23 (dd, 1H, *J*= 8.50 and 1.20 Hz, H-8), 8.16 (d, 2H, *J*= 8.10 Hz, H-3′′′′ and H-5′′), 7.92 (dd, 1H, *J*= 8.50 and 1.20 Hz, H-5), 7.81 (s, 1H, H-3), 7.81 (ddd,1H, *J*= 8.50, 6.70 and 1.20 Hz, H-7), 7.54–7.44 (m, 7H, H-3′, H-5′, H-2′′, H-6′′, H-2′, H-6′ and H-6),) , 3.94 (s, 2H, NCH_2_), 3.91 (s, 2H, NCH_2_), 2.81 (t, 2H, *J*= 6.90 Hz, NCH_2_), 2.74 (t, 2H, *J*= 6.90 Hz, NCH_2_), 2.61–2.40 (m. 20H, 2NCH_2_ and 8NCH_2_pip.), 2.30 (s, 3H, NCH_3_), 2.29 (s, 3H, NCH_3_). 13C NMR δ (75 MHz, CDCl_3_) 158.0 (C-2), 150.3 (C-4), 150.2 (C-8a), 142.7 (C-1′′), 142.0 (C-1′), 139.9 (C-4′′), 138.5 (C-4′), 131.5 (C-7), 131.0 (C-3′′ and C-5′′), 130.9 (C-8), 130.1 (C-3′ and C-5′), 129.8 (C-2′′ and C-6′′), 129.0 (C-2′ and C-6′), 127.6 (C-5), 127.2 (C-4a), 127.0 (C-6), 120.6 (C-3), 59.0 (NCH_2_), 58.9(NCH_2_), 56.5 (NCH_2_), 55.0 (NCH_2_), 54.9 (NCH_2_), 54.5 (NCH_2_), 47.4 (NCH_3_), 47.1 (NCH_2_), 46.7 (NCH_2_); MALDI-TOF MS *m/z* [M + H]^+^ Calc for C_37_H_50_N_7_: 592.413, Found: 592.525.

**2,4-Bis{4-[(3-morpholinopropyl)aminomethyl]phenyl}quinoline (1g)**

Yellow oil (97%); ^1^H NMR δ (300 MHz, CDCl_3_) 8.21 (dd, 1H, *J*= 8.40 and 1.20 Hz, H-8), 8.14 (d, 2H, *J*= 8.10 Hz, H-3′′ and H-5′′), 7.89 (dd, 1H, *J*= 8.40 and 1.20 Hz, H-5), 7.79 (s, 1H, H-3), 7.70(ddd,1H, *J*= 8.40, 6.90 and 1.20 Hz, H-7), 7.53–7.41 (m, 7H, H-3′, H-5′, H-2′′, H-6′′, H-2′, H-6′ and H-6), 3.88 (s, 2H, NCH_2_), 3.84 (s, 2H, NCH_2_), 3.69 (t, 2H, *J*= 4.50 Hz, OCH_2_), 3.67 (t, 2H, *J*= 4.50 Hz, OCH_2_), 2.74 (t, 2H, *J*= 6.90 Hz, NCH_2_), 2.68 (t, 2H, *J*= 6.90 Hz, NCH_2_), 2.45–2.35 (m, 12H, 6NCH_2_), 1.85 (bs, 2H, 2NH), 1.78–1.64 (m, 4H, 2CH_2_). 13C NMR δ (75 MHz, CDCl_3_) 158.0 (C-2), 150.3 (C-4), 150.2 (C-8a), 143.2 (C-1′′), 142.3 (C-1′), 139.6 (C-4′′), 138.3 (C-4′), 131.4 (C-7), 131.0 (C-3′′ and C-5′′), 130.9 (C-8), 129.8 (C-3′ and C-5′), 129.6 (C-2′′ and C-6′′), 128.9 (C-2′ and C-6′), 127.6 (C-5), 127.1 (C-4a), 127.0 (C-6), 120.5 (C-3), 68.4 (OCH_2_), 58.8 (NCH_2_), 55.2 (NCH_2_), 49.5 (NCH_2_), 49.3 (NCH_2_), 28.1 (CH_2_); MALDI-TOF MS *m/z* [M + H]^+^ Calc for C_37_H_48_N_5_O_2_: 594.381, Found: 594.465.

**2,4-Bis{4-[(2-morpholinoethyl)aminomethyl]phenyl}quinoline (1h)**

Pale-yellow oil (76%); ^1^H NMR δ (300 MHz, CDCl_3_) 8.22 (dd, 1H, *J*= 8.10 and 1.20 Hz, H-8), 8.15 (d, 2H, *J*= 8.10 Hz, H-3′′ and H-5′′), 7.89 (dd, 1H, *J*= 8.10 and 1.20 Hz, H-5), 7.79 (s, 1H, H-3), 7.71 (ddd,1H, *J*= 8.10, 6.90 and 1.20 Hz, H-7), 7.54–7.41 (m, 7H, H-3′, H-5′, H-2′′, H-6′′, H-2′, H-6′ and H-6), 3.90 (s, 2H, NCH_2_), 3.87 (s, 2H, NCH_2_), 3.69 (t, 2H, *J*= 4.70 Hz, OCH_2_), 3.68 (t, 2H, *J*= 4.70 Hz, OCH_2_), 2.77 (t, 2H, *J*= 6.20 Hz, NCH_2_), 2.70 (t, 2H, *J*= 6.20 Hz, NCH_2_), 2.53 (t, 2H, *J*= 6.20 Hz, NCH_2_), 2.47 (t, 2H, *J*= 6.20 Hz, NCH_2_), 2.45–2.39 (m, 8H, 4NCH_2_), 2.20 (bs, 2H, 2NH). 13C NMR δ (75 MHz, CDCl_3_) 158.0 (C-2), 150.3 (C-4), 150.2 (C-8a), 143.1 (C-1′′), 142.2 (C-1′), 139.7 (C-4′′), 138.4 (C-4'), 131.4 (C-7), 131.0 (C-3′′ and C-5′′), 130.9 (C-8), 129.9 (C-3′ and C-5′), 129.7 (C-2′′ and C-6′′), 129.0 (C-2′ and C-6′), 127.6 (C-5), 127.1 (C-4a), 127.0 (C-6), 120.6 (C-3), 68.4 (OCH_2_), 63.6 (NCH_2_), 59.7 (NCH_2_), 59.6 (NCH_2_), 55.1 (NCH_2_), 55.0 (NCH_2_), 54.9 (NCH_2_), 46.8 (NCH_2_), 46.5 (NCH_2_); MALDI-TOF MS *m/z* [M + H]^+^ Calc for C_35_H_44_N_5_O_2_: 566.350, Found: 566.488.

**6-Methoxy-2,4-bis{4-[(4-dimethylaminobutyl)aminomethyl]phenyl}quinoline (1i)**

Yellow oil (97%); ^1^H NMR δ (300 MHz, CDCl_3_) 8.06 (m, 3H, H-2′′ and H-6′′, H-8), 7.69 (s, 1H, H-3), 7.46–7.37 (m, 6H, H-2′ and H-6′, H-3′′ and H-5′′, H-3′ and H-5′), 7.32–7.28 (m, 1H, H-7), 7.14–7.13 (m, 1H, H-5), 3.83 (s, 2H, NCH_2_), 3.78 (s, 2H, NCH_2_), 3.70 (s, 3H, CH_3_O), 2.70–2.56 (m, 4H, 2NCH_2_), 2.24–2.19 (m, 4H, 2NCH_2_), 2.15 (s, 6H, N(CH_3_)_2_), 2.13 (s, 6H, N(CH_3_)_2_), 1.52–1.46 (m, 8H, 4CH_2_); 13C NMR δ (75 MHz, CDCl_3_) 159.0 (C-6), 155.7 (C-2), 148.9 (C-4), 146.2 (C-8a), 142.7 (C-4′), 142.1 (C-4′′), 139.7 (C-1′), 138.6 (C-1′′), 132.9 (C-8), 130.7 (C-3′ and C-5′), 129.8 (C-3′′and C-5′′), 129.7 (C-2′ and C-6′), 128.6 (C-2′′ and C-6′′), 127.9 (C-4a), 123.1 (C-7), 120.8 (C-3), 105.0 (C-5), 61.0 (2NCH_2_), 56.8 (CH_3_O), 55.0 (2NCH_2_), 50.9 (NCH_2_), 50.6 (NCH_2_), 46.8 (2N(CH_3_)_2_), 29.3 (2CH_2_), 26.9 (2CH_2_); MALDI-TOF MS *m/z* [M + H]^+^ Calc for C_36_H_48_N_5_O: 566.386, Found: 566.285.

**6-Methoxy-2,4-bis{4-[(3-dimethylaminopropyl)aminomethyl]phenyl}quinoline (1j)**

Yellow oil (97%); ^1^H NMR δ (300 MHz, CDCl_3_) 8.07 (m, 3H, H-2′′and H-6′′, H-8), 7.71 (s, 1H, H-3), 7.49 (d, 2H, *J*= 8.20 Hz, H-2′ and H-6′), 7.45 (d, 2H, *J*= 8.20 Hz, H-3′′and H-5′′), 7.41 (d, 2H, *J*= 8.20 Hz, H-3′ and H-5′), 7.32 (dd, 1H, *J*= 9.30 and 2.70 Hz, H-7), 7.16 (d, 1H, *J*= 2.70 Hz, H-5), 3.85 (s, 2H, NCH_2_), 3.81 (s, 2H, NCH_2_), 3.73 (s, 3H, CH_3_O), 2.71 (t, 2H, *J*= 7.10 Hz, NCH_2_), 2.64 (t, 2H, *J*= 7.10 Hz, NCH_2_), 2.32 (t, 2H, *J*= 7.10 Hz, NCH_2_), 2.27 (t, 2H, *J =*  7.10 Hz, NCH_2_), 2.19 (s, 6H, N(CH_3_)_2_), 2.17 (s, 6H, N(CH_3_)_2_), 1.74–1.60 (m, 4H, 2CH_2_); 13C NMR δ (75 MHz, CDCl_3_) 159.0 (C-6), 155.7 (C-2), 148.9 (C-4), 146.2 (C-8a), 142.8 (C-4′), 142.2 (C-4′′), 139.7 (C-1′), 138.6 (C-1′′), 133.9 (C-8), 130.7 (C-3′ and C-5′), 129.8 (C-3′′and C-5′′), 129.7 (C-2′ and C-6′), 128.6 (C-2′′and C-6′′), 127.9 (C-4a), 123.1 (C-7), 120.9 (C-3), 105.0 (C-5), 59.5 (NCH_2_), 59.4 (NCH_2_), 56.8 (CH_3_O), 55.2 (NCH_2_), 55.1 (NCH_2_), 49.5 (NCH_2_), 49.2 (NCH_2_), 47.0 (N(CH_3_)_2_), 46.9 (N(CH_3_)_2_), 29.5 (CH_2_), 29.4 (CH_2_); MALDI-TOF MS *m/z* [M + H]^+^ Calc for C_34_H_44_N_5_O: 538.354, Found: 538.372.

**6-Methoxy-2,4-bis{4-[(4–(4-methylpiperazin-1-yl)butyl)aminomethyl]phenyl}quinoline (1k)**

Pale yellow oil (97%); ^1^H NMR δ (300 MHz, CDCl_3_) 8.03 (d, 2H, *J*= 8.20 Hz, H-2′′and H-6′′), 8.02 (d, 1H, *J*= 9.15 Hz, H-8), 7.66 (s, 1H, H-3), 7.45 (d, 2H, *J*= 8.20 Hz, H-2′ and H-6′), 7.41 (d, 2H, *J*= 8.20 Hz, H-3′′and H-5′′), 7.36 (d, 2H, *J*= 8.20 Hz, H-3′ and H-5′), 7.28 (dd, 1H, *J*= 9.15 and 2.70 Hz, H-7), 7.11 (d, 1H, *J*= 2.70 Hz, H-5), 3.81 (s, 2H, NCH_2_), 3.76 (s, 2H, NCH_2_), 3.69 (s, 3H, CH_3_O), 2.65–2.22 (m, 24H, 4NCH_2_ and 8NCH_2_ pip.), 2.18 (s, 3H, NCH_3_), 2.17 (s, 3H, NCH_3_), 1.49–1.42 (m, 8H, 4CH_2_); 13C NMR δ (75 MHz, CDCl_3_) 159.0 (C-6), 155.6 (C-2), 148.9 (C-4), 146.2 (C-8a), 142.7 (C-4′), 142.2 (C-4′′), 139.6 (C-1′), 138.5 (C-1′′), 132.8 (C-8), 130.7 (C-3′ and C-5′), 129.8 (C-3′′and C-5′′), 129.7 (C-2′ and C-6′), 128.5 (C-2′′and C-6′′), 127.9 (C-4a), 123.0 (C-7), 120.8 (C-3), 105.0 (C-5), 59.8 (2NCH_2_), 56.7 (CH_3_O), 56.5 (2NCH_2_ pip.), 55.0 (2NCH_2_), 54.5 (2NCH_2_ pip.), 50.9 (NCH_2_), 50.5 (NCH_2_), 47.4 (2NCH_3_), 29.4 (2CH_2_), 26.1 (2CH_2_); MALDI-TOF MS m/z [M + H]^+^ Calc for C_42_H_60_N_7_O: 678.486, Found: 678.487.

**6-Methoxy-2,4-bis{4-[(3–(4-methylpiperazin-1-yl)propyl)aminomethyl]phenyl}quinoline (1l)**

Yellow oil (97%); ^1^H NMR δ (300 MHz, CDCl_3_) 8.10 (d, 2H, *J*= 8.25 Hz, H-2′′ and H-6′′), 8.07 (d, 1H, *J*= 9.10 Hz, H-8), 7.72 (s, 1H, H-3), 7.51 (d, 2H, *J*= 8.25 Hz, H-2′ and H-6′), 7.47 (d, 2H, *J*= 8.25 Hz, H-3′′ and H-5′′), 7.41 (d, 2H, *J*= 8.25 Hz, H-3′ and H-5′), 7.34 (dd, 1H, *J*= 9.10 and 2.80 Hz, H-7), 7.17 (d, 1H, *J*= 2.80 Hz, H-5), 3.86 (s, 2H, NCH_2_), 3.82 (s, 2H, NCH_2_), 3.75 (s, 3H, CH_3_O), 2.73 (t, 2H, *J*= 6.70 Hz, NCH_2_), 2.65 (t, 2H, *J*= 6.70 Hz, NCH_2_), 2.45–2.34 (m, 16H, 8NCH_2_ pip), 2.24 (s, 3H, NCH_3_), 2.23 (s, 3H, NCH_3_), 1.78–1.63 (m, 4H, 2CH_2_); 13C NMR δ (75 MHz, CDCl_3_) 159.1 (C-6), 155.8 (C-2), 148.9 (C-4), 146.2 (C-8a), 142.7 (C-4′), 142.1 (C-4′′), 139.7 (C-1′), 138.6 (C-1′′), 132.8 (C-8), 130.7 (C-3′ and C-5′), 129.8 (C-3′′and C-5′′), 129.7 (C-2′ and C-6′), 128.6 (C-2′′and C-6′′), 128.0 (C-4a), 123.1 (C-7), 120.9 (C-3), 105.0 (C-5), 58.4 (2NCH_2_), 56.8 (CH_3_O), 56.5 (2NCH_2_ pip.), 55.1 (2NCH_2_), 54.6 (2NCH_2_ pip.), 49.8 (NCH_2_), 49.4 (NCH_2_), 47.4 (2NCH_3_), 28.3 (2CH_2_); MALDI-TOF MS m/z [M + H]^+^ Calc for C_40_H_56_N_7_O: 650.454, Found: 650.442.

**7-Methoxy-2,4-bis{4-[(4-dimethylaminobutyl)aminomethyl]phenyl}quinoline (1m)**

Yellow oil (97%); ^1^H NMR δ (300 MHz, CDCl_3_) 8.07 (d, 2H, *J*= 8.40 Hz, H-2′′and H-6′′), 7.73 (d, 1H, *J*= 9.25 Hz, H-5), 7.60 (s, 1H, H-3), 7.49 (d, 1H, *J*= 2.60 Hz, H-8), 7.42–7.38 (m, 6H, H-2′ and H-6′, H-3′′ and H-5′′, H-3′ and H-5′), 7.03 (dd, 1H, *J*= 9.25 and 2.60 Hz, H-6), 3.90 (s, 3H, CH_3_O), 3.81 (s, 2H, NCH_2_), 3.79 (s, 2H, NCH_2_), 2.64 (t, 2H, *J*= 6.60 Hz, NCH_2_), 2.59 (t, 2H, *J*= 6.60 Hz, NCH_2_), 2.21 (t, 2H, *J*= 6.60 Hz, NCH_2_), 2.19 (t, 2H, *J*= 6.60 Hz, NCH_2_), 2.15 (s, 6H, N(CH_3_)_2_), 2.13 (s, 6H, N(CH_3_)_2_), 1.50–1.44 (m, 8H, 4CH_2_); MALDI-TOF MS m/z [M + H]^+^ Calc for C_36_H_50_N_5_O: 568.402, Found: 568.860.

**7-Methoxy-2,4-bis{4-[(3-dimethylaminopropyl)aminomethyl]phenyl}quinoline (1n)**

Pale yellow oil (97%); ^1^H NMR δ (300 MHz, CDCl_3_) 8.09 (d, 2H, *J*= 8.10 Hz, H-2′′ and H-6′′), 7.75 (d, 1H, *J*= 9.15 Hz, H-5), 7.62 (s, 1H, H-3), 7.51 (d, 1H, *J*= 2.40 Hz, H-8), 7.46–7.40 (m, 6H, H-2′ and H-6′, H-3′′ and H-5′′, H-3′ and H-5′), 7.32 (dd, 1H, *J*= 9.15 and 2.40 Hz, H-6), 3.92 (s, 3H, CH_3_O), 3.84 (s, 2H, NCH_2_), 3.82 (s, 2H, NCH_2_), 2.70 (t, 2H, *J*= 7.20 Hz, NCH_2_), 2.64 (t, 2H, *J*= 7.20 Hz, NCH_2_), 2.31 (t, 2H, *J*= 7.20 Hz, NCH_2_), 2.28 (t, 2H, *J*= 7.20 Hz, NCH_2_), 2.19 (s, 6H, N(CH_3_)_2_), 2.17 (s, 6H, N(CH_3_)_2_), 1.73–1.60 (m, 4H, 2CH_2_); 13C NMR δ (75 MHz, CDCl_3_) 162.0 (C-7), 158.4 (C-2), 152.0 (C-4), 150.2 (C-8a), 142.9 (C-4′), 142.2 (C-4′′), 139.8 (C-1′), 138.4 (C-1′′), 130.9 (C-3′ and C-5′), 129.9 (C-3′′and C-5′′), 129.6 (C-2′ and C-6′), 128.9 (C-2′′and C-6′′), 128.1 (C-8), 122.2 (C-4a), 120.6 (C-6), 118.6 (C-3), 109.3 (C-5), 59.4 (2NCH_2_), 56.9 (CH_3_O), 55.0 (2NCH_2_), 49.4 (NCH_2_), 49.2 (NCH_2_), 46.9 (2N(CH_3_)_2_), 29.3 (2CH_2_); MALDI-TOF MS m/z [M + H]^+^ Calc for C_34_H_46_N_5_O: 540.370, Found: 540.292.

**7-Methoxy-2,4-bis{4-[(4–(4-methylpiperazin-1-yl)butyl)aminomethyl]phenyl}quinoline (1o)**

Yellow oil (73%); ^1^H NMR δ (300 MHz, CDCl_3_) 8.13 (d, 2H, *J*= 8.10 Hz, H-2′′and H-6′′), 7.81 (d, 1H, *J*= 9.25 Hz, H-5), 7.67 (s, 1H, H-3), 7.57 (d, 1H, *J*= 2.60 Hz, H-8), 7.51 (s, 4H, H-2′ and H-6′, H-3′′ and H-5′′), 7.47 (d, 2H, *J*= 8.10 Hz, H-3′ and H-5′), 7.13 (dd, 1H, *J*= 9.25 and 2.60 Hz, H-6), 4.00 (s, 3H, CH_3_O), 3.91 (s, 2H, NCH_2_), 3.88 (s, 2H, NCH_2_), 2.74 (t, 2H, *J*= 6.60 Hz, NCH_2_), 2.68 (t, 2H, *J*= 6.60 Hz, NCH_2_), 2.47–2.36 (m, 20H, 2NCH_2_ and 8NCH_2_ pip), 2.29 (s, 3H, NCH_3_), 2.28 (s, 3H, NCH_3_), 1.62–1.53 (m, 8H, 4CH_2_); 13C NMR δ (75 MHz, CDCl_3_) 162.7 (C-7), 157.1 (C-2), 150.7 (C-4), 148.3 (C-8a), 141.6 (C-4′), 140.7 (C-4′′), 138.6 (C-1′), 137.2 (C-1′′), 129.6 (C-3′ and C-5′), 128.6 (C-3′′ and C-5′′), 128.3 (C-2′ and C-6′), 127.6 (C-2′′ and C-6′′), 126.8 (C-8), 120.9 (C-4a), 119.2 (C-6), 117.3 (C-3), 107.9 (C-5), 58.5 (2NCH_2_), 55.6 (CH_3_O), 55.1 (2NCH_2_ pip.), 53.7 (NCH_2_), 53.6 (NCH_2_), 53.2 (2NCH_2_ pip.), 49.4 (NCH_2_), 49.2 (NCH_2_), 46.0 (2NCH_3_), 28.1 (2CH_2_), 24.8 (2CH_2_); MALDI-TOF MS m/z [M + H]^+^ Calc for C_42_H_60_N_7_O: 678.486, Found: 678.697.

**7-Methoxy-2,4-bis{4-[(3–(4-methylpiperazin-1-yl)propyl)aminomethyl]phenyl}quinoline (1p)**

Yellow oil (92%); ^1^H NMR δ (300 MHz, CDCl_3_) 8.03 (d, 2H, *J*= 8.25 Hz, H-2′′ and H-6′′), 7.69 (d, 1H, *J*= 9.20 Hz, H-5), 7.56 (s, 1H, H-3), 7.45 (d, 1H, *J*= 2.50 Hz, H-8), 7.39 (s, 4H, H-2′ and H-6′, H-3′′ and H-5′′), 7.36 (d, 2H, *J*= 8.25 Hz, H-3′ and H-5′), 7.00 (dd, 1H, *J*= 9.20 and 2.50 Hz, H-6), 3.86 (s, 3H, CH_3_O), 3.78 (s, 2H, NCH_2_), 3.75 (s, 2H, NCH_2_), 2.64 (t, 2H, *J*= 6.75 Hz, NCH_2_), 2.58 (t, 2H, *J*= 6.75 Hz, NCH_2_), 2.37–2.28 (m, 16H, 8NCH_2_ pip), 2.17 (s, 3H, NCH_3_), 2.16 (s, 3H, NCH_3_), 1.69–1.57 (m, 4H, 2CH_2_); 13C NMR δ (75 MHz, CDCl_3_) 162.0 (C-7), 158.3 (C-2), 152.0 (C-4), 150.1 (C-8a), 143.0 (C-4′), 142.2 (C-4′′), 139.7 (C-1′), 138.4 (C-1′′), 130.8 (C-3′ and C-5′), 129.8 (C-3′′ and C-5′′), 129.5 (C-2′ and C-6′), 128.8 (C-2′′ and C-6′′), 128.0 (C-8), 122.1 (C-4a), 120.5 (C-6), 118.5 (C-3), 109.2 (C-5), 58.3 (2NCH_2_), 56.8 (CH_3_O), 56.5 (2NCH_2_ pip.), 55.0 (2NCH_2_), 54.6 (2NCH_2_ pip.), 49.6 (NCH_2_), 49.4 (NCH_2_), 47.4 (2NCH_3_), 28.3 (2CH_2_); MALDI-TOF MS m/z [M + H]^+^ Calc for C_40_H_56_N_7_O: 650.455, Found: 650.475.

**2,4-Bis{3-[(3-dimethylaminopropyl)aminomethyl]phenyl}quinoline (1q)**

Yellow oil (97%); ^1^H NMR δ (300 MHz, CDCl_3_) 8.22 (dd, 1H, *J*= 8.10 and 1.20 Hz, H-8), 8.14 (dd, 1H, *J*= 1.50 and 1.50 Hz, H-2′), 8.06 (ddd, 1H, *J*= 7.20, 1.50 and 1.50 Hz, H-6′), 7.87 (dd, 1H, *J*= 8.10 and 1.20 Hz, H-5), 7.83 (s, 1H, H-3), 7.70 (ddd,1H, *J*= 8.10, 6.90 and 1.20 Hz, H-7), 7.51–7.28 (m, 7H, H-6, H-2′′, H-6′′, H-4′, H-4′′, H-5′ and H-5′′), 3.90 (s, 2H, NCH_2_), 3.89 (s, 2H, NCH_2_), 2.72 (t, 2H, *J*= 7.20 Hz, NCH_2_), 2.70 (t, 2H, *J*= 7.20 Hz, NCH_2_), 2.31 (t, 4H, *J*= 7.20 Hz, 2NCH_2_), 2.19 (s, 6H, N(CH_3_)_2_), 2.18 (s, 6H, N(CH_3_)_2_), 1.69 (qt, 4H, *J*= 7.20 Hz, 2CH_2_); 13C NMR δ (75 MHz, CDCl_3_) 158.2 (C-2), 150.6 (C-4), 150.1 (C-8a), 142.4 (C-1′′ and C-3′), 141.1 (C-3′′), 139.8 (C-1′), 131.4 (C-6′), 130.8 (C-6′′), 130.5 (C-2′ and C-4′′), 130.3 (C-2′′), 130.0 (C-4′), 129.5 (C-7 and C-5′′), 128.7 (C-5’), 127.6 (C-8 and C-5), 127.2 (C-4a), 127.0 (C-6), 120.8 (C-3), 59.4 (NCH_2_), 55.5 (NCH_2_), 55.3 (NCH_2_), 49.4 (NCH_2_), 46.9 (NCH_3_), 29.4 (CH_2_); MALDI-TOF MS m/z [M + H]^+^ Calc for C_33_H_44_N_5_: 510.360, Found: 510.391.

**2,4-Bis{3-[(3–(4-methylpiperazin-1-yl)propyl)aminomethyl]phenyl}quinoline (1r)**

Pale-yellow oil (88%); ^1^H NMR δ (300 MHz, CDCl_3_) 8.22 (dd, 1H, *J*= 8.40 and 1.20 Hz, H-8), 8.14 (dd, 1H, *J*= 1.50 and 1.50 Hz, H-2′), 8.06 (ddd, 1H, *J*= 7.20, 1.50 and 1.50 Hz, H-6′), 7.88 (dd, 1H, *J*= 8.40 and 1.20 Hz, H-5), 7.83 (s, 1H, H-3), 7.72 (ddd,1H, *J*= 8.40, 7.20 and 1.20 Hz, H-7), 7.51–7.36 (m, 7H, H-6, H-2′′, H-6′′, H-4′, H-4′′, H-5′ and H-5′′), 3.91 (s, 2H, NCH_2_), 3.90 (s, 2H, NCH_2_), 2.76–2.69 (m, 4H, 2NCH_2_), 2.70 (t, 2H, *J*= 7.20 Hz, NCH_2_), 2.31 (t, 4H, *J*= 7.20 Hz, 2NCH_2_), 2.19 (s, 6H, N(CH_3_)_2_), 2.18 (s, 6H, N(CH_3_)_2_), 2.57–2.28 (m, 20H, 10NCH_2_), 2.22 (s, 3H, NCH_3_), 2.19 (s, 3H, NCH_3_), 1.72 (qt, 4H, *J*= 6.90 Hz, 2CH_2_); 13C NMR δ (75 MHz, CDCl_3_) 158.2 (C-2), 150.6 (C-4), 150.1 (C-8a), 142.4 (C-1′′ and C-3′), 141.1 (C-3′′), 139.8 (C-1′), 131.4 (C-6′), 130.9 (C-6′′), 130.5 (C-2′) 130.4 (C-4′′), 130.3 (C-2′′), 130.0 (C-4′ and C-7), 129.5 (C-5′′), 128.6 (C-5′ and C-8), 127.6 (C-5), 127.3 (C-4a), 127.0 (C-6), 120.8 (C-3), 58.4 (NCH_2_), 56.5 (NCH_2_), 55.4 (NCH_2_), 55.3 (NCH_2_), 54.6 (NCH_2_), 49.8 (NCH_2_), 49.6 (NCH_2_), 47.4 (NCH_3_), 28.3 (CH_2_); MALDI-TOF MS m/z [M + H]^+^ Calc for C_39_H_54_N_7_: 620.444, Found: 620.924.

**2,4-Bis{3-[(3-morpholinopropyl)aminomethyl]phenyl}quinoline (1s)**

Yellow oil (90%); ^1^H NMR δ (300 MHz, CDCl_3_) 8.22 (dd, 1H, *J*= 8.10 and 1.00 Hz, H-8), 8.15 (dd, 1H, *J*= 1.50 and 1.50 Hz, H-2′), 8.05 (ddd, 1H, *J*= 7.50, 1.50 and 1.50 Hz, H-6′), 7.87 (dd, 1H, *J*= 8.10 and 1.00 Hz, H-5), 7.82 (s, 1H, H-3), 7.71 (ddd,1H, *J*= 8.10, 6.90 and 1.00 Hz, H-7), 7.52–7.39 (m, 7H, H-6, H-2′′, H-6′′, H-4′, H-4′′, H-5′ and H-5′′), 3.89 (s, 2H, NCH_2_), 3.88 (s, 2H, NCH_2_), 3.65 (t, 4H, *J*= 4.80 Hz, 2OCH_2_), 3.63 (t, 4H, *J*= 4.80 Hz, 2OCH_2_), 2.75–2.68 (m, 4H, 2NCH_2_), 2.41–2.36 (m, 12H, 6NCH_2_), 1.71 (qt, 4H, *J*= 7.20 Hz, 2CH_2_); 13C NMR δ (75 MHz, CDCl_3_) 158.1 (C-2), 150.6 (C-4), 150.1 (C-8a), 142.3 (C-1′′ and C-3′), 141.1 (C-3′′), 139.8 (C-1′), 131.4 (C-6′), 130.9 (C-6′′), 130.5 (C-2′ and C-4′′), 130.3 (C-2′′), 130.0 (C-4′), 129.5 (C-5′′ and C-7), 128.7 (C-5′), 127.7 (C-5 and C-8), 127.2 (C-4a), 127.0 (C-6), 120.7 (C-3), 68.3 (OCH_2_), 58.8 (NCH_2_), 55.4 (NCH_2_), 55.1 (NCH_2_), 49.5 (NCH_2_), 49.4 (NCH_2_), 27.9 (CH_2_); MALDI-TOF MS m/z [M + H]^+^ Calc for C_37_H_48_N_5_O_2_: 594.381, Found: 594.324.

**2,4-Bis{3-[(2-morpholinoethyl)aminomethyl]phenyl}quinoline (1t)**

Orange oil (98%); ^1^H NMR δ (300 MHz, CDCl_3_) 8.26 (dd, 1H, *J*= 8.40 and 1.20 Hz, H-8), 8.18 (dd, 1H, *J*= 1.50 and 1.50 Hz, H-2′), 8.09 (ddd, 1H, *J*= 7.40, 1.50 and 1.50 Hz, H-6′), 7.91 (dd, 1H, *J*= 8.40 and 1.20 Hz, H-5), 7.85 (s, 1H, H-3), 7.75 (ddd,1H, *J*= 8.40, 6.90 and 1.20 Hz, H-7), 7.54–7.44 (m, 7H, H-6, H-2′′, H-6′′, H-4′, H-4′′, H-5′ and H-5′′), 3.96 (s, 2H, NCH_2_), 3.95 (s, 2H, NCH_2_), 3.68 (t, 4H, *J*= 4.80 Hz, 2OCH_2_), 3.67 (t, 4H, *J*= 4.80 Hz, 2OCH_2_), 2.79 (t, 2H, *J*= 6.60 Hz, NCH_2_), 2.77 (t, 2H, *J*= 6.60 Hz, NCH_2_), 2.54 (t, 2H, *J*= 6.60 Hz, NCH_2_), 2.53 (t, 2H, *J*= 6.60 Hz, NCH_2_), 2.44–2.40 (m, 8H, 6NCH_2_), 2.22 (bs, 2H, 2NH); 13C NMR δ (75 MHz, CDCl_3_) 156.8 (C-2), 149.2 (C-4), 148.8 (C-8a), 140.9 (C-1′′ and C-3′), 139.8 (C-3′′), 138.5 (C-1′), 130.1 (C-6′), 129.6 (C-6′′), 129.2 (C-2′ and C-4′′), 128.9 (C-2′′), 128.6 (C-4′), 128.2 (C-5′′ and C-7), 127.4 (C-5′), 126.3 (C-5 and C-8), 125.9 (C-4a), 125.6 (C-6), 119.4 (C-3), 67.0 (OCH_2_), 58.2 (NCH_2_), 53.7 (NCH_2_), 45.4 (NCH_2_), 45.2 (NCH_2_); MALDI-TOF MS m/z [M + H]^+^ Calc for C_35_H_44_N_5_O_2_: 566.349, Found: 566.339.

**1,3-Bis{4-[(4-dimethylaminobutyl)aminomethyl]phenyl}isoquinoline (2a)**

Yellow oil (88%); ^1^H NMR δ (300 MHz, CDCl_3_) 8.15 (d, 2H, *J*= 8.10 Hz, H-3′′ and H-5′′), 8.10 (d, 1H, *J*= 8.40 Hz, H-8), 8.01 (s, 1H, H-4), 7.86 (d, 1H, *J*= 8.40 Hz, H-5), 7.76 (d, 2H, *J*= 8.10 Hz, H-3′ and H-5′), 7.62 (t, 1H, *J*= 8.40 Hz, H-6), 7.48 (d, 2H, *J*= 8.10 Hz, H-2′′ and H-6′′), 7.45 (t, 1H, *J*= 8.40 Hz, H-7), 7.41 (d, 2H, *J*= 8.10 Hz, H-2′ and H-6′), 3.88 (s, 2H, NCH_2_), 3.83 (s, 2H, NCH_2_), 2.69 (t, 2H, *J*= 6.90 Hz, NCH_2_), 2.64 (t, 2H, *J*= 6.90 Hz, NCH_2_), 2.29–2.22 (m, 4H, 2NCH_2_), 2.20 (s, 6H, N(CH_3_)_2_), 2.18 (s, 6H, N(CH_3_)_2_), 1.55–1.48 (m, 8H, 4CH_2_); 13C NMR δ (75 MHz, CDCl_3_) 161.5 (C-3), 151.3 (C-1), 142.3 (C-4’), 142.0 (C-4′′), 139.9 (C-1′), 139.7 (C-1′′), 139.2 (C-4a), 131.7 (C-2′ and C-6′), 131.3 (C-6), 129.8 (C-2′′ and C-6′′), 129.4 (C-3′ and C-5′), 128.9 (C-7), 128.8 (C5), 128.4 (C-3′′ and C-5′′), 128.1 (C-8), 127.1 (C-8a), 116.7 (C-4), 61.1 (2NCH_2_), 55.1 (NCH_2_), 50.7 (NCH_2_), 50.6 (NCH_2_), 46.8 (2 N(CH_3_)_2_), 29.3 (2CH_2_), 26.9 (2CH_2_); MALDI-TOF MS m/z [M + H]^+^ Calc for C_35_H_48_N_5_: 538.391, Found: 538.389.

**1,3-Bis{4-[(3-dimethylaminopropyl)aminomethyl]phenyl}isoquinoline (2b)**

Yellow oil (83%); ^1^H NMR δ (300 MHz, CDCl_3_) 8.17 (d, 2H, *J*= 8.10 Hz, H-3′′ and H-5′′), 8.13 (d, 1H, *J*= 8.10 Hz, H-8), 8.05 (s, 1H, H-4), 7.91 (d, 1H, *J*= 8.10 Hz, H-5), 7.78 (d, 2H, *J*= 8.10 Hz, H-3′ and H-5′), 7.67 (t, 1H, *J*= 8.10 Hz, H-6), 7.51 (d, 2H, *J*= 8.10 Hz, H-2′′ and H-6′′), 7.47 (t, 1H, *J*= 8.10 Hz, H-7), 7.44 (d, 2H, *J*= 8.10 Hz, H-2′ and H-6′), 3.92 (s, 2H, NCH_2_), 3.86 (s, 2H, NCH_2_), 2.75 (t, 2H, *J*= 6.90 Hz, NCH_2_), 2.70 (t, 2H, *J*= 6.90 Hz, NCH_2_), 2.36 (t, 2H, *J*= 6.90 Hz, NCH_2_), 2.33 (t, 2H, *J*= 6.90 Hz, NCH_2_), 2.24 (s, 6H, N(CH_3_)_2_), 2.22 (s, 6H, N(CH_3_)_2_), 1.79–1.65 (m, 4H, 2CH_2_); 13C NMR δ (75 MHz, CDCl_3_) 161.5 (C-3), 151.4 (C-1), 142.3 (C-4′), 142.1 (C-4′′), 139.9 (C-1′), 139.7 (C-1′′), 139.2 (C-4a), 131.7 (C-2′ and C-6′), 131.4 (C-6), 129.8 (C-2′′ and C-6′′), 129.4 (C-3′ and C-5′), 128.9 (C-7), 128.8 (C5), 128.5 (C-3′′ and C-5′′), 128.2 (C-8), 127.1 (C-8a), 116.8 (C-4), 59.5 (2NCH_2_), 55.2 (2NCH_2_), 49.3 (2NCH_2_), 46.9 (2NCH_2_), 29.4 (2CH_2_); MALDI-TOF MS m/z [M + H]^+^ Calc for C_33_H_44_N_5_: 510.359, Found: 510.360.

**1,3-Bis{4-[(4–(4-methylpiperazin-1-yl)butyl)aminomethyl]phenyl}isoquinoline (2c)**

Yellow oil (65%); ^1^H NMR δ (300 MHz, CDCl_3_) 8.14 (d, 2H, *J*= 8.10 Hz, H-3′′ and H-5′′), 8.09 (dd, 1H, *J*= 8.10 and 1.20 Hz, H-8), 8.00 (s, 1H, H-4), 7.86 (dd, 1H, *J*= 8.10 and 1.20 Hz, H-5), 7.74 (d, 2H, *J*= 8.10 Hz, H-3′ and H-5′), 7.61 (ddd, 1H, *J*= 8.10, 7.90 and 1.20 Hz, H-6), 7.46 (d, 2H, *J*= 8.10 Hz, H-2′′ and H-6′′), 7.45 (ddd, 1H, *J*= 8.10, 7.90 and 1.20 Hz, H-7), 7.39 (d, 2H, *J*= 8.10 Hz, H-2′ and H-6′), 3.87 (s, 2H, NCH_2_), 3.81 (s, 2H, NCH_2_), 2.66 (t, 2H, *J*= 6.80 Hz, NCH_2_), 2.62 (t, 2H, *J*= 6.80 Hz, NCH_2_), 2.45–2.28 (m, 20H, 2NCH_2_ and 8 NCH_2_pip.), 2.24 (s, 3H, NCH_3_), 2.23 (s, 3H, NCH_3_), 1.57–1.48 (m, 8H, 4CH_2_); 13C NMR δ (75 MHz, CDCl_3_) 161.5 (C-3), 151.3 (C-1), 142.3 (C-4′), 142.1 (C-4′′), 139.9 (C-1′), 139.6 (C-1′′), 139.2 (C-4a), 131.6 (C-2′ and C-6′), 131.3 (C-6), 129.8 (C-2′′ and C-6′′), 129.3 (C-3′ and C-5′), 128.8 (C-7), 128.7 (C5), 128.4 (C-3′′ and C-5′′), 128.1 (C-8), 127.1 (C-8a), 116.7 (C-4), 59.9 (NCH_2_), 56.5 (NCH_2_pip.), 55.1 (NCH_2_), 54.6 (NCH_2_pip.), 50.7 (NCH_2_), 50.6 (NCH_2_), 47.4 (NCH_3_), 29.5 (CH_2_), 26.1 (CH_2_); MALDI-TOF MS m/z [M + H]^+^ Calc for C_41_H_58_N_7_: 648.475, Found: 648.473.

**1,3-Bis{4-[(3–(4-methylpiperazin-1-yl)propyl)aminomethyl]phenyl}isoquinoline (2d)**

Yellow oil (87%); ^1^H NMR δ (300 MHz, CDCl_3_) 8.17 (d, 2H, *J*= 8.10 Hz, H-3′′ and H-5′′), 8.12 (d, 1H, *J*= 8.10 Hz, H-8), 8.04 (s, 1H, H-4), 7.91 (d, 1H, *J*= 8.10 Hz, H-5), 7.77 (d, 2H, *J*= 8.10 Hz, H-3′ and H-5′), 7.66 (t, 1H, *J*= 8.10 Hz, H-6), 7.50 (d, 2H, *J*= 8.10 Hz, H-2′′ and H-6′′), 7.48 (t, 1H, *J*= 8.10 Hz, H-7), 7.42 (d, 2H, *J*= 8.10 Hz, H-2′ and H-6′), 3.90 (s, 2H, NCH_2_), 3.85 (s, 2H, NCH_2_), 2.75 (t, 2H, *J*= 6.90 Hz, NCH_2_), 2.69 (t, 2H, *J*= 6.90 Hz, NCH_2_), 2.50–2.37 (m, 20H, 2NCH_2_ and 8 NCH_2_pip.), 2.27 (s, 3H, NCH_3_), 2.26 (s, 3H, NCH_3_), 1.80–1.67 (m, 8H, 4CH_2_); 13C NMR δ (75 MHz, CDCl_3_) 161.6 (C-3), 151.3 (C-1), 142.3 (C-4′), 142.1 (C-4′′), 139.9 (C-1′), 139.7 (C-1′′), 139.2 (C-4a), 131.7 (C-2′ and C-6′), 131.4 (C-6), 129.8 (C-2′′ and C-6′′), 129.3 (C-3′ and C-5′), 128.9 (C-7), 128.8 (C5), 128.4 (C-3′′ and C-5′′), 128.2 (C-8), 127.1 (C-8a), 116.7 (C-4), 58.4 (NCH_2_), 56.5 (NCH2pip.), 55.1 (NCH_2_), 54.7 (NCH_2_pip.), 49.6 (NCH_2_), 49.4 (NCH_2_), 47.4 (NCH_3_), 28.4 (CH_2_); MALDI-TOF MS m/z [M + H]^+^ Calc for C_39_H_54_N_7_: 620.444, Found: 620.441.

**7-Methoxy-1,3-bis{4-[(4-dimethylaminobutyl)aminomethyl]phenyl}isoquinoline (2e)**

Orange oil (85%); ^1^H NMR δ (300 MHz, CDCl_3_) 8.13 (d, 2H, *J*= 8.10 Hz, H-3′′ and H-5′′), 7.97 (s, 1H, H-4), 7.81 (d, 1H,*J*= 9.00 Hz, H-5), 7.79 (d, 2H, *J*= 8.10 Hz, H-3′ and H-5′), 7.51 (d, 2H, *J*= 8.10 Hz, H-2′′ and H-6′′), 7.43 (d, 1H, *J*= 2.40 Hz, H-8), 7.41 (d, 2H, *J*= 8.10 Hz, H-2′ and H-6′), 7.32 (dd, 1H, *J*= 9.00 and 2.40 Hz, H-6), 3.91 (s, 2H, NCH_2_), 3.85 (s, 2H, NCH_2_), 3.81 (s, 3H, CH_3_O), 2.73 (t, 2H, *J*= 6.90 Hz, NCH_2_), 2.67 (t, 2H, *J*= 6.90 Hz, NCH_2_), 2.31–2.24 (m, 4H, 2NCH_2_), 2.22 (s, 6H, N(CH_3_)_2_), 2.20 (s, 6H, N(CH_3_)_2_), 1.59–1.48 (m, 8H, 4CH_2_); 13C NMR δ (75 MHz, CDCl_3_) 159.9 (C-1), 159.6 (C-7), 149.7 (C-3), 142.1 (C-4′), 141.6 (C-4′′), 140.2 (C-1′), 139.8 (C-1′′), 134.8 (C-4a and C-8a), 131.4 (C-2′ and C-6′), 130.4 (C-5), 129.8 (C-2′′ and C-6′′), 129.5 (C-3′ and C-5′), 128.1 (C-3′′ and C-5′′), 124.2 (C-6), 116.7 (C-4), 106.6 (C-8), 61.1 (NCH_2_), 56.8 (OCH_3_), 55.2 (NCH_2_), 55.1 (NCH_2_), 50.8 (NCH_2_), 50.6 (NCH_2_), 46.8 (2 N(CH_3_)_2_), 29.4 (2CH_2_), 27.0 (2CH_2_); MALDI-TOF MS m/z [M + H]^+^ Calc for C_36_H_50_N_5_O: 568.401, Found: 568.435.

**7-Methoxy-1,3-bis{4-[(3-dimethylaminopropyl)aminomethyl]phenyl}isoquinoline (2f)**

Yellow oil (70%); ^1^H NMR δ (300 MHz, CDCl_3_) 8.14 (d, 2H, *J*= 8.30 Hz, H-3′′ and H-5′′), 8.00 (s, 1H, H-4), 7.84 (d, 1H, *J*= 8.90 Hz, H-5), 7.81 (d, 2H, *J*= 8.30 Hz, H-3′ and H-5′), 7.51 (d, 2H, *J*= 8.30 Hz, H-2′′ and H-6′′), 7.44 (d, 1H, *J*= 2.40 Hz, H-8), 7.42 (d, 2H, *J*= 8.30 Hz, H-2′ and H-6′), 7.35(dd, 1H, *J*= 8.90 and 2.40 Hz, H-6), 3.93 (s, 2H, NCH_2_), 3.86 (s, 2H, NCH_2_), 3.84 (s, 3H, CH_3_O), 2.77 (t, 2H, *J*= 6.90 Hz, NCH_2_), 2.70 (t, 2H, *J*= 6.90 Hz, NCH_2_), 2.37 (t, 2H, *J*= 6.90 Hz, NCH_2_), 2.31 (t, 2H, *J*= 6.90 Hz, NCH_2_), 2.25 (s, 6H, N(CH_3_)_2_), 2.23 (s, 6H, N(CH_3_)_2_), 1.80–1.66 (m, 8H, 4CH_2_); 13C NMR δ (75 MHz, CDCl_3_) 159.9 (C-1), 159.6 (C-7), 149.8 (C-3), 142.2 (C-4′), 141.8 (C-4′′), 140.2 (C-1′), 139.8 (C-1′′), 134.8 (C-4a and C-8a), 131.4 (C-2′ and C-6′), 130.3 (C-5), 129.8 (C-2′′ and C-6′′), 129.4 (C-3′ and C-5′), 128.2 (C-3′′ and C-5′′), 124.3 (C-6), 116.7 (C-4), 106.7 (C-8), 59.4 (NCH_2_), 56.8 (OCH_3_), 55.3 (NCH_2_), 49.4 (NCH_2_), 49.2 (NCH_2_), 46.9 (2 N(CH_3_)_2_), 29.4 (2CH_2_); MALDI-TOF MS m/z [M + H]^+^ Calc for C_34_H_46_N_5_O: 540.370, Found: 540.367.

**7-Methoxy-1,3-bis{4-[(4–(4-methylpiperazin-1-yl)butyl)aminomethyl]phenyl}isoquinoline (2g)**

Yellow oil (98%); ^1^H NMR δ (300 MHz, CDCl_3_) 8.10 (d, 2H, *J*= 8.10 Hz, H-3′′ and H-5′′), 7.94 (s, 1H, H-4), 7.78 (d, 1H, *J*= 9.00 Hz, H-5), 7.76 (d, 2H, *J*= 8.10 Hz, H-3′ and H-5′), 7.47 (d, 2H, *J*= 8.10 Hz, H-2′′ and H-6′′), 7.40 (d, 1H, *J*= 2.40 Hz, H-8),7.38 (d, 2H, *J*= 8.10 Hz, H-2′ and H-6′), 7.29 (dd, 1H, *J*= 9.00 and 2.40 Hz, H-6), 3.87 (s, 2H, NCH_2_), 3.81 (s, 2H, NCH_2_), 3.78 (s, 3H, CH_3_O), 2.70 (t, 2H, *J*= 6.60 Hz, NCH_2_), 2.63 (t, 2H, *J*= 6.60 Hz, NCH_2_), 2.45–2.28 (m, 20H, 2NCH_2_ and 8NCH2pip.), 2.24 (s, 3H, NCH_3_), 2.23 (s, 3H, NCH_3_), 1.55–1.45 (m, 8H, 4CH_2_); 13C NMR δ (75 MHz, CDCl_3_) 159.9 (C-1), 159.5 (C-7), 149.7 (C-3), 142.0 (C-4′), 141.5 (C-4′′), 140.2 (C-1′), 139.8 (C-1′′), 134.8 (C-4a and C-8a), 131.4 (C-2′ and C-6′), 130.2 (C-5), 129.8 (C-2′′ and C-6′′), 129.4 (C-3′ and C-5′), 128.2 (C-3′′ and C-5′′), 124.2 (C-6), 116.6 (C-4), 106.6 (C-8), 59.8 (NCH_2_), 56.8 (OCH_3_), 56.4 (NCH_2_), 55.1 (NCH_2_), 55.0 (NCH_2_), 54.5 (NCH2pip.), 50.7 (NCH_2_), 50.5 (NCH_2_), 47.4 (NCH_3_), 29.4 (2CH_2_), 26.1 (2CH_2_); MALDI-TOF MS m/z [M + H]^+^ Calc for C_42_H_60_N_7_O: 678.486, Found: 678.492.

**7-Methoxy-1,3-bis{4-[(3–(4-methylpiperazin-1-yl)propyl)aminomethyl]phenyl}isoquinoline (2h)**

Yellow oil (98%); ^1^H NMR δ (300 MHz, CDCl_3_) 8.13 (d, 2H, *J*= 8.10 Hz, H-3′′ and H-5′′), 7.98 (s, 1H, H-4), 7.82 (d, 1H, *J*= 9.00 Hz, H-5), 7.79 (d, 2H, *J*= 8.10 Hz, H-3′ and H-5′), 7.50 (d, 2H, *J*= 8.10 Hz, H-2′′ and H-6′′), 7.42 (d, 1H, *J*= 2.40 Hz, H-8), 7.41 (d, 2H, *J*= 8.10 Hz, H-2′ and H-6′), 7.33 (dd, 1H, *J*= 9.00 and 2.40 Hz, H-6), 3.91 (s, 2H, NCH_2_), 3.83 (s, 2H, NCH_2_), 3.82 (s, 3H, CH_3_O), 2.76 (t, 2H, *J*= 6.60 Hz, NCH_2_), 2.72 (t, 2H, *J*= 6.60 Hz, NCH_2_), 2.48–2.30 (m, 20H, 2NCH_2_ and 8NCH_2_pip.), 2.28 (s, 3H, NCH_3_), 2.27 (s, 3H, NCH_3_), 1.81–1.67 (m, 8H, 4CH_2_); 13C NMR δ (75 MHz, CDCl_3_) 159.9 (C-1), 159.6 (C-7), 149.8 (C-3), 142.1 (C-4′), 141.6 (C-4′′), 140.2 (C-1′), 139.8 (C-1′′), 134.8 (C-4a and C-8a), 131.4 (C-2′ and C-6′), 130.3 (C-5), 129.8 (C-2′′ and C-6′′), 129.4 (C-3′ and C-5′), 128.2 (C-3′′ and C-5′′), 124.2 (C-6), 116.7 (C-4), 106.7 (C-8), 58.4 (NCH_2_), 56.8 (OCH_3_), 56.5 (NCH_2_pip.), 55.2 (NCH_2_), 55.1 (NCH_2_), 54.6 (NCH_2_pip.), 49.7 (NCH_2_), 49.4 (NCH_2_), 47.4 (NCH_3_), 28.2 (2CH_2_), 28.1 (2CH_2_); MALDI-TOF MS m/z [M + H]^+^ Calc for C_40_H_56_N_7_O: 650.454, Found: 650.451.

**6-Methoxy-1,3-bis{4-[(4-dimethylaminobutyl)aminomethyl]phenyl}isoquinoline (2i)**

Yellow oil (95%); ^1^H NMR δ (300 MHz, CDCl_3_) 8.14 (d, 2H, *J*= 8.40 Hz, H-3′′ and H-5′′), 8.00 (d, 1H, *J*= 9.30 Hz, H-8), 7.94 (s, 1H, H-4), 7.74 (d, 2H, *J*= 8.40 Hz, H-3′ and H-5′), 7.48 (d, 2H, *J*= 8.40 Hz, H-2′′ and H-6′′), 7.42 (d, 2H, *J*= 8.40 Hz, H-2′ and H-6′), 7.15 (d, 1H, *J*= 2.40 Hz, H-5), 7.09 (dd, 1H, *J*= 9.30 and 2.40 Hz, H-7), 3.94 (s, 3H, CH_3_O),) , 3.89 (s, 2H, NCH_2_), 3.84 (s, 2H, NCH_2_),2.70 (t, 2H, *J*= 6.90 Hz, NCH_2_), 2.66 (t, 2H, *J*= 6.90 Hz, NCH_2_), 2.28–2.19 (m, 4H, 2NCH_2_), 2.21 (s, 6H, N(CH_3_)_2_), 2.20 (s, 6H, N(CH_3_)_2_), 1.57–1.51 (m, 8H, 4CH_2_); 13C NMR δ (75 MHz, CDCl_3_) 161.9 (C-3), 161.0 (C-6), 151.9 (C-1), 142.2 (C-4′), 142.0 (C-4′′), 141.2 (C-4a), 140.0 (C-1′), 139.9 (C-1′′), 131.6 (C-2′ and C-6′), 130.7 (C-7), 129.8(C-2′′ and C-6′′), 129.4 (C-3′ and C-5′), 128.5 (C-3′′ and C-5′′), 122.8 (C-8a), 120.9 (C-5), 116.2 (C-4), 106.3 (C-8), 61.1 (2NCH_2_), 56.8 (OCH_3_), 55.1 (2NCH_2_), 50.6 (2NCH_2_), 46.8 (2 N(CH_3_)_2_), 29.3 (2CH_2_), 26.9 (2CH_2_); MALDI-TOF MS m/z [M + H]^+^ Calc for C_36_H_50_N_5_O: 568.401, Found: 568.407.

**6-Methoxy-1,3-bis{4-[(3-dimethylaminopropyl)aminomethyl]phenyl}isoquinoline (2j)**

Pale-yellow oil (96%); ^1^H NMR δ (300 MHz, CDCl_3_) 8.11 (d, 2H, *J*= 8.10 Hz, H-3′′ and H-5′′), 7.96 (d, 1H, *J*= 9.00 Hz, H-8), 7.89 (s, 1H, H-4), 7.71 (d, 2H, *J*= 8.10 Hz, H-3′ and H-5′), 7.45 (d, 2H, *J*= 8.10 Hz, H-2′′ and H-6′′), 7.38 (d, 2H, *J*= 8.40 Hz, H-2′ and H-6′), 7.10 (d, 1H, *J*= 2.40 Hz, H-5), 7.04 (dd, 1H, *J*= 9.00 and 2.40 Hz, H-7), 3.88 (s, 3H, CH_3_O),) , 3.86 (s, 2H, NCH_2_), 3.81 (s, 2H, NCH_2_), 2.70 (t, 2H, *J*= 6.90 Hz, NCH_2_), 2.65 (t, 2H, *J*= 6.90 Hz, NCH_2_), 2.32 (t, 2H, *J*= 6.90 Hz, NCH_2_), 2.29 (t, 2H, *J*= 6.90 Hz, NCH_2_), 2.20 (s, 6H, N(CH_3_)_2_), 2.18 (s, 6H, N(CH_3_)_2_), 1.71–1.63 (m, 4H, 2CH_2_); 13C NMR δ (75 MHz, CDCl_3_) 161.9 (C-3), 160.9 (C-6), 151.9 (C-1), 142.1 (C-4′), 141.9 (C-4′′), 141.2 (C-4a), 140.0 (C-1′), 139.8 (C-1′′), 131.5 (C-2′ and C-6′), 130.6 (C-7), 129.7 (C-2′′ and C-6′′), 129.3 (C-3′ and C-5′), 128.4 (C-3′′ and C-5′′), 122.8 (C-8a), 119.9 (C-5), 116.2 (C-4), 106.2 (C-8), 59.4 (2NCH_2_), 56.7 (OCH_3_), 55.1 (2NCH_2_), 49.1 (2NCH_2_), 46.9 (2 N(CH_3_)_2_), 29.4 (2CH_2_); MALDI-TOF MS m/z [M + H]^+^ Calc for C_34_H_46_N_5_O: 540.370, Found: 540.368.

**6-Methoxy-1,3-bis{4-[(4–(4-methylpiperazin-1-yl)butyl)aminomethyl]phenyl}isoquinoline (2k)**

Yellow oil (97%); ^1^H NMR δ (300 MHz, CDCl_3_) 8.11 (d, 2H, *J*= 8.10 Hz, H-3′′ and H-5′′), 7.97 (d, 1H, *J*= 9.30 Hz, H-8), 7.91 (s, 1H, H-4), 7.71 (d, 2H, *J*= 8.10 Hz, H-3′ and H-5′), 7.44 (d, 2H, *J*= 8.10 Hz, H-2′′ and H-6′′), 7.38 (d, 2H, *J*= 8.10 Hz, H-2′ and H-6′), 7.12 (d, 1H, *J*= 2.40 Hz, H-5), 7.06 (dd, 1H, *J*= 9.30 and 2.40 Hz, H-7), 3.91 (s, 3H, CH_3_O),) , 3.86 (s, 2H, NCH_2_), 3.81 (s, 2H, NCH_2_), 2.67–2.62 (m, 4H, 2NCH_2_), 2.41–2.30 (m, 20H, 2NCH_2_ and 8NCH2pip.), 2.24 (s, 3H, NCH_3_), 2.23 (s, 3H, NCH_3_), 1.53–1.48 (m, 8H, 4CH_2_); 13C NMR δ (75 MHz, CDCl_3_) 161.9 (C-3), 161.0 (C-6), 151.9 (C-1), 142.3 (C-4′), 142.1 (C-4′′), 141.2 (C-4a), 140.0 (C-1′), 139.8 (C-1′′), 131.6 (C-2′ and C-6′), 130.7 (C-7), 129.7 (C-2′′ and C-6′′), 129.3 (C-3′ and C-5′), 128.4 (C-3′′ and C-5′′), 122.8 (C-8a), 120.9 (C-5), 116.2 (C-4), 106.2 (C-8), 59.9 (NCH_2_), 56.8 (OCH_3_), 55.2 (NCH_2_), 54.6 (NCH_2_pip.), 50.7 (NCH_2_), 50.6 (NCH_2_), 47.4 (NCH_3_), 29.5 (2CH_2_), 26.1 (2CH_2_); MALDI-TOF MS m/z [M + H]^+^ Calc for C_42_H_60_N_7_O: 678.486, Found: 678.485.

**6-Methoxy-1,3-bis{4-[(3–(4-methylpiperazin-1-ylpropyl)aminomethyl]phenyl}isoquinoline (2l)**

Yellow oil (59%); ^1^H NMR δ (300 MHz, CDCl_3_) 8.14 (d, 2H, *J*= 8.20 Hz, H-3′′ and H-5′′), 8.00 (d, 1H, *J*= 9.20 Hz, H-8), 7.95 (s, 1H, H-4), 7.74 (d, 2H, *J*= 8.20 Hz, H-3′ and H-5′), 7.48 (d, 2H, *J*= 8.20 Hz, H-2′′ and H-6′′), 7.42 (d, 2H, *J*= 8.20 Hz, H-2′ and H-6′), 7.16 (d, 1H, *J*= 2.50 Hz, H-5), 7.10 (dd, 1H, *J*= 9.20 and 2.50 Hz, H-7), 3.96 (s, 3H, CH_3_O),) , 3.89 (s, 2H, NCH_2_), 3.84 (s, 2H, NCH_2_), 2.73 (t, 2H, *J*= 6.90 Hz, NCH_2_), 2.69 (t, 2H, *J*= 6.90 Hz, NCH_2_), 2.47 (bs, 2H, 2NH), 2.46–2.38 (m, 16H, 8NCH_2_pip.), 2.44 (t, 2H, *J*= 6.90 Hz, NCH_2_), 2.41 (t, 2H, *J*= 6.90 Hz, NCH_2_), 2.27 (s, 3H, NCH_3_), 2.26 (s, 3H, NCH_3_), 1.79–1.67 (m, 4H, 2CH_2_); 13C NMR δ (75 MHz, CDCl_3_) 162.0 (C-3), 161.0 (C-6), 152.0 (C-1), 142.2 (C-4′), 142.1 (C-4′′), 141.2 (C-4a), 140.0 (C-1′), 139.8 (C-1′′), 131.6 (C-2′ and C-6′), 130.7 (C-7), 129.7 (C-2′′ and C-6′′), 129.3 (C-3′ and C-5′), 128.4 (C-3′′ and C-5′′), 122.8 (C-8a), 120.9 (C-5), 116.2 (C-4), 106.2 (C-8), 58.4 (NCH_2_), 56.8 (OCH_3_), 56.5 (NCH_2_pip.), 55.1 (NCH_2_), 54.6 (NCH_2_), 49.5 (NCH_2_), 47.4 (NCH_3_), 28.3 (2CH_2_); MALDI-TOF MS m/z [M + H]^+^ Calc for C_40_H_56_N_7_O: 650.454, Found: 650.451.

**2,4-Bis{4-[(4-dimethylaminobutyl)aminomethyl]phenyl}quinazoline (3a)**

Yellow oil (83%); ^1^H NMR δ (300 MHz, CDCl_3_) 8.69 (d, 2H, *J*= 8.10 Hz, H-2′ and H-6′), 8.17 (dd, 1H, *J*= 8.40 and 1.20 Hz, H-8), 8.14 (dd, 1H, *J*= 8.40 and 1.20 Hz, H-5), 7.91 (ddd, 1H, *J*= 8.40, 7.00 and 1.20 Hz, H-7), 7.89 (d, 2H, *J*= 8.10 Hz, H-2′′ and H-6′′), 7.63 (d, 2H, *J*= 8.10 Hz, H-3′ and H-5′), 7.57 (ddd, 1H, *J*= 8.40, 7.00 and 1.20 Hz, H-6), 7.55 (d, 2H, *J*= 8.10 Hz, H-3′′ and H-5′′), 3.99 (s, 2H, NCH_2_), 3.96 (s, 2H, NCH_2_), 2.79 (t, 2H, *J*= 6.90 Hz, NCH_2_), 2.77 (t, 2H, *J*= 6.90 Hz, NCH_2_), 2.37–2.32 (m, 4H, 2NCH_2_), 2.26 (s, 6H, N(CH_3_)_2_), 2.19 (s, 6H, N(CH_3_)_2_), 1.76–1.52 (m, 8H, 4CH_2_). 13C NMR δ (75 MHz, CDCl_3_) 169.5 (C-2), 161.5 (C-4), 153.3 (C-8a), 144.2 (C-4′′), 143.9 (C-4′), 138.4 (C-1′′), 137.7 (C-1′), 134.8 (C-7), 131.7 (C-3′′and C-5′′), 130.4 (C-8), 130.1 (C-3', C-5',C-2'', C-6'', C-2' and C-6'), 128.4 (C-5), 128.2 (C-6), 123.0 (C-4a), 61.0 (NCH_2_), 55.1 (NCH_2_), 50.7 (NCH_2_), 50.6(NCH_2_), 46.8 (2 N(CH_3_)_2_), 29.3 (2CH_2_), 26.8 (2CH_2_); MALDI-TOF MS *m/z* [M + H]^+^ Calc for C_34_H_47_N_6_: 539.386, Found: 539.245.

**2,4-Bis{4-[(3-dimethylaminopropyl)aminomethyl]phenyl}quinazoline (3b)**

Yellow oil (97%); IR ν_max_ (KBr)/cm^−1^ 3290 (NH), 1610 (C = N); ^1^H NMR δ (300 MHz, CDCl_3_) 8.64 (d, 2H, *J*= 8.10 Hz, H-2′ and H-6′), 8.13 (d, 2H, *J*= 9.00 Hz, H-8 and H-5), 7.90–7.84 (m, 3H, H-7, H-2′′ and H-6′′), 7.57–7.46 (m, 5H, H-3′, H-5′, H-6, H-3′′ and H-5′′), 3.93 (s, 2H, NCH_2_), 3.87 (s, 2H, NCH_2_), 2.75 (t, 2H, *J*= 6.90 Hz, NCH_2_), 2.70 (t, 2H, *J*= 6.90 Hz, NCH_2_), 2.36 (t, 2H, *J*= 6.90 Hz, NCH_2_), 2.33 (t, 2H, *J*= 6.90 Hz, NCH_2_), 2.24 (s, 6H, N(CH_3_)_2_), 2.22 (s, 6H, N(CH_3_)_2_), 1.79–1.70 (m, 4H, 2CH_2_). 13C NMR δ (75 MHz, CDCl_3_) 169.5 (C-2), 161.5 (C-4), 153.4 (C-8a), 144.3 (C-4′′), 144.0 (C-4′), 138.4 (C-1′′), 137.7 (C-1′), 134.9 (C-7), 131.7 (C-3′′ and C-5′′), 130.5 (C-8), 130.1 (C-3′, C-5′), 129.6 (C-2′′, C-6′′, C-2′ and C-6′), 128.4 (C-5), 128.2 (C-6), 123.0 (C-4a), 59.5 (NCH_2_), 55.2 (NCH_2_), 49.4 (NCH_2_), 49.2 (NCH_2_), 47.00 (2N(CH_3_)_2_), 29.4 (2CH_2_); MALDI-TOF MS *m/z* [M + H]^+^ Calc for C_32_H_43_N_6_: 511.355, Found: 511.400.

**2,4-Bis{4-[(2-dimethylaminoethyl)aminomethyl]phenyl}quinazoline (3c)**

Yellow oil (85%); ^1^H NMR δ (300 MHz, CDCl_3_) 8.64 (d, 2H, *J*= 8.20 Hz, H-2′ and H-6′), 8.14–8.10 (m, 2H, H-8 and H-5), 7.88–7.83 (m, 3H, H-7, H-2′′ and H-6′′), 7.56 (d, 2H, *J*= 8.20 Hz, H-3′ and H-5′), 7.49–7.45 (m, 3H, H-6, H-3′′ and H-5′′), 3.95 (s, 2H, NCH_2_), 3.90 (s, 2H, NCH_2_), 2.77 (t, 2H, *J*= 6.40 Hz, NCH_2_), 2.71 (t, 2H, *J*= 6.40 Hz, NCH_2_), 2.47 (t, 2H, *J*= 6.40 Hz, NCH_2_), 2.44 (t, 2H, *J*= 6.40 Hz, NCH_2_), 2.24 (s, 6H, N(CH_3_)_2_), 2.19 (s, 6H, N(CH_3_)_2_).13C NMR δ (75 MHz, CDCl_3_) 169.5 (C-2), 161.5 (C-4), 153.4 (C-8a), 144.4 (C-4′′), 144.0 (C-4′), 138.3 (C-1′′), 137.7 (C-1'), 134.8 (C-7), 131.7 (C-3′′ and C-5′′), 130.4(C-8), 130.1 (C-3′, C-5′), 129.7 (C-2′′ and C-6′′), 129.6 (C-2′ and C-6′), 128.4 (C-5), 128.2 (C-6), 123.0 (C-4a), 60.5 (NCH_2_), 55.2 (NCH_2_), 48.2 (NCH_2_), 49.2 (NCH_2_), 47.8 (NCH_2_), 47.0 (N(CH_3_)_2_),46.9 (N(CH_3_)_2_); MALDI-TOF MS *m/z* [M + H]^+^ Calc for C_30_H_39_N_6_: 483.324, Found: 483.397.

**2,4-Bis{4-[(4–(4-methylpiperazin-1-yl)butyl)aminomethyl]phenyl}quinazoline (3d)**

Yellow oil (73%); ^1^H NMR δ (300 MHz, CDCl_3_) 8.63 (d, 2H, *J*= 8.40 Hz, H-2′ and H-6′), 8.15–8.10 (m, 2H, H-8 and H-5), 7.87 (ddd, 1H, *J*= 8.10, 7.20 and 1.50 Hz, H-7), 7.84 (d, 2H, *J*= 8.40 Hz, H-2′′ and H-6′′), 7.54 (d, 2H, *J*= 8.40 Hz, H-3′ and H-5′), 7.52 (ddd, 1H, *J*= 8.10, 7.20 and 1.50 Hz, H-6), 7.45 (d, 2H, *J*= 8.40 Hz, H-3′′ and H-5′′), 3.91 (s, 2H, NCH_2_), 3.87 (s, 2H, NCH_2_), 2.71–2.55 (m, 4H, 2NCH_2_), 2.46–2.34 (m, 20H, 2NCH_2_ and 8NCH_2_pip.), 2.27 (s, 3H, NCH_3_), 2.26 (s, 3H, NCH_3_), 1.57–1.50 (m, 8H, 4CH_2_). 13C NMR δ (75 MHz, CDCl_3_) 169.5 (C-2), 161.5 (C-4), 153.3 (C-8a), 144.3 (C-4′′), 143.9 (C-4'), 138.4 (C-1′′), 137.7 (C-1′), 134.9 (C-7), 131.7 (C-3′′ and C-5′′), 130.5 (C-8), 130.1 (C-3' and C-5'), 129.6 (C-2′′, C-6′′, C-2′ and C-6′), 128.7 (C-5), 128.3 (C-6), 123.0 (C-4a), 59.8 (NCH_2_), 56.5 (NCH_2_), 55.1 (NCH_2_), 54.6 (NCH_2_), 50.7 (NCH_2_), 50.6 (NCH_2_), 47.4 (2NCH_3_), 29.4 (2CH_2_), 26.1 (2CH_2_); MALDI-TOF MS *m/z* [M + H]^+^ Calc for C_40_H_57_N_8_: 649.471, Found: 649.291.

**2,4-Bis{4-[(3–(4-methylpiperazin-1-yl)propyl)aminomethyl]phenyl}quinazoline (3e)**

Yellow oil (70%); ^1^H NMR δ (300 MHz, CDCl_3_) 8.62 (d, 2H, *J*= 8.25 Hz, H-2′ and H-6′), 8.13–8.09 (m, 2H, H-8 and H-5), 7.87 (ddd, 1H, *J*= 8.10, 6.90 and 1.20 Hz, H-7), 7.83 (d, 2H, *J*= 8.25 Hz, H-2′′ and H-6′′), 7.53 (d, 2H, *J*= 8.25 Hz, H-3' and H-5′), 7.51 (ddd, 1H, *J*= 8.10, 6.90 and 1.20 Hz, H-6), 7.45 (d, 2H, *J*= 8.25 Hz, H-3′′ and H-5′′), 3.90 (s, 2H, NCH_2_), 3.86 (s, 2H, NCH_2_), 2.73 (t, 2H, *J*= 6.90 Hz, NCH_2_), 2.68 (t, 2H, *J*= 6.90 Hz, NCH_2_), 2.46–2.35 (m, 20H, 2NCH_2_ and 8NCH_2_pip.), 2.26 (s, 3H, NCH_3_), 2.25 (s, 3H, NCH_3_), 1.77–1.68 (m, 4H, 2CH_2_). 13C NMR δ (75 MHz, CDCl_3_) 169.5 (C-2), 161.5 (C-4), 153.3 (C-8a), 144.2 (C-4′′), 143.9 (C-4′), 138.4 (C-1′′), 137.7 (C-1′), 134.9 (C-7), 131.7 (C-3′′ and C-5′′), 130.4 (C-8), 130.1 (C-3′ and C-5′), 129.6 (C-2′′, C-6′′, C-2′ and C-6′), 128.6 (C-5), 128.2 (C-6), 123.0 (C-4a), 58.4 (NCH_2_), 56.5 (NCH_2_), 55.1 (NCH_2_), 54.6 (NCH_2_), 49.6 (NCH_2_), 49.5(NCH_2_), 47.4 (2NCH_3_), 28.2 (2CH_2_); MALDI-TOF MS m/z [M + H]^+^ Calc for C_38_H_53_N_8_: 621.439, Found: 621.626.

**2,4-Bis{4-[(3-morpholinopropyl)aminomethyl]phenyl}quinazoline (3f)**

Yellow oil (95%); ^1^H NMR δ (300 MHz, CDCl_3_) 8.66 (d, 2H, *J*= 8.10 Hz, H-3′′ and H-5′′), 8.14 (dd, 1H, *J*= 8.10 and 1.20 Hz, H-8), 8.11 (dd, 1H, *J*= 8.10 and 1.20 Hz, H-5), 7.91 (ddd,1H, *J*= 8.10, 7.20 and 1.20 Hz, H-7), 7.86 (d, 2H, *J*= 8.10 Hz, H-3′ and H-5′), 7.59 (d, 2H, *J*= 8.10 Hz, H-2′′ and H-6′′), 7.54 (ddd,1H, *J*= 8.10, 7.20 and 1.20 Hz, H-6), 7.51 (d, 2H, *J*= 8.10 Hz, H-2' and H-6'), 3.97 (s, 2H, NCH_2_), 3.94 (s, 2H, NCH_2_), 3.68 (t, 4H, *J*= 4.50 Hz, 2OCH_2_), 3.68 (t, 4H, *J*= 4.50 Hz, 2OCH_2_), 2.81 (t, 2H, *J*= 6.60 Hz, NCH_2_), 2.80 (t, 2H, *J*= 6.60 Hz, NCH_2_), 2.49–2.40 (m, 12H, 6NCH_2_), 1.85–1.75 (m, 4H, 2CH_2_). 13C NMR δ (75 MHz, CDCl_3_) 169.4 (C-2), 161.2 (C-4), 153.3 (C-8a), 142.6 (C-4′′), 142.0 (C-4′), 138.9 (C-1′′), 138.0 (C-1'), 135.0 (C-7), 131.8 (C-3′′ and C-5′′), 130.5 (C-8), 130.3 (C-3′ and C-5′), 130.0 (C-2′′ and C-6′′), 129.8 (C-2'′ and C-6′), 128.4 (C-5), 128.3 (C-6), 123.0 (C-4a), 68.3 (OCH_2_), 58.8 (NCH_2_), 58.7 (NCH_2_), 55.1 (NCH_2_), 54.7 (NCH_2_), 54.5 (NCH_2_), 49.2 (NCH_2_), 27.4 (CH_2_), 26.8 (CH_2_); MALDI-TOF MS m/z [M + H]^+^ Calc for C_36_H_47_N_6_O_2_: 595.376, Found: 595.326.

**2,4-Bis{4-[(2-morpholinoethyl)aminomethyl]phenyl}quinazoline (3g)**

Yellow oil (78%); ^1^H NMR δ (300 MHz, CDCl_3_) 8.65 (d, 2H, *J*= 8.10 Hz, H-3′′ and H-5′′), 8.14 (dd, 1H, *J*= 8.40 and 1.50 Hz, H-8), 8.12 (dd, 1H, *J*= 8.40 and 1.50 Hz, H-5), 7.87 (ddd,1H, *J*= 8.40, 7.20 and 1.50 Hz, H-7), 7.86 (d, 2H, *J*= 8.10 Hz, H-3′ and H-5′), 7.58–7.46 (m, 5H, H-2′′, H-6′′,H-6, H-2′ and H-6′), 3.95 (s, 2H, NCH_2_), 3.91 (s, 2H, NCH_2_), 3.74–3.68 (m, 8H, 4OCH_2_), 2.79 (t, 2H, *J*= 6.30 Hz, NCH_2_), 2.73 (t, 2H, *J*= 6.30 Hz, NCH_2_), 2.57 (t, 2H, *J*= 6.30 Hz, NCH_2_), 2.51 (t, 2H, *J*= 6.30 Hz, NCH_2_), 2.47–2.38 (m, 8H, 4NCH_2_), 2.20 (bs, 2H, 2NH). 13C NMR δ (75 MHz, CDCl_3_) 169.5 (C-2), 161.5 (C-4), 153.4 (C-8a), 144.3 (C-4′′), 143.9 (C-4′), 138.4 (C-1′′), 137.7 (C-1′), 134.9 (C-7), 131.7 (C-3′′ and C-5′′), 130.5 (C-8), 130.1 (C-3′ and C-5′), 129.6 (C-2′′ and C-6′′), 129.5 (C-2′ and C-6′), 128.4 (C-5), 128.3 (C-6), 123.0 (C-4a), 68.4 (OCH_2_), 59.6 (NCH_2_), 55.1 (NCH_2_), 55.0 (NCH_2_), 46.8 (NCH_2_), 46.5 (NCH_2_); MALDI-TOF MS m/z [M + H]^+^ Calc for C_34_H_43_N_6_O_2_: 567.345, Found: 567.326.

**6-Methoxy-2,4-bis{4-[(4-dimethylaminobutyl)aminomethyl]phenyl}quinazoline (3h)**

Yellow oil (96%); ^1^H NMR δ (300 MHz, CDCl_3_) 8.58 (d, 2H, *J*= 8.10 Hz, H-2′′ and H-6′′), 8.00 (d, 1H, *J*= 9.20 Hz, H-8), 7.84 (d, 2H, *J*= 8.10 Hz, H-2′ and H-6′), 7.53 (d, 2H, *J*= 8.10 Hz, H-3′′ and H-5′′), 7.49 (dd, 1H, *J*= 9.20 and 2.80 Hz, H-7), 7.44 (d, 2H, *J*= 8.10 Hz, H-3′ and H-5′), 7.36 (d, 1H, *J*= 2.80 Hz, H-5), 3.90 (s, 2H, NCH_2_), 3.86 (s, 2H, NCH_2_), 3.81 (s, 3H, CH_3_O), 2.71 (t, 2H, *J*= 7.00 Hz, NCH_2_), 2.65 (t, 2H, *J*= 7.00 Hz, NCH_2_), 2.26 (t, 2H, *J*= 7.00 Hz, NCH_2_), 2.24 (t, 2H, *J*= 7.00 Hz, NCH_2_), 2.20 (s, 6H, N(CH_3_)_2_), 2.17 (s, 6H, N(CH_3_)_2_), 1.56–1.48 (m, 8H, 4CH_2_); 13C NMR δ (75 MHz, CDCl_3_) 167.6 (C-6), 159.7 (C-2), 159.4 (C-4), 149.5 (C-8a), 143.3 (C-4′), 143.1 (C-4′′), 138.7 (C-1′), 138.1 (C-1′′), 131.9 (C-8), 131.3 (C-3′ and C-5′), 129.8 (C-3′′ and C-5′′, C-2′ and C-6′, C-2′′ and C-6′′), 127.5 (C-7), 123.7 (C-4a), 105.7 (C-5), 60.9 (NCH_2_), 60.8 (NCH_2_), 57.0 (CH_3_O), 54.9 (NCH_2_), 54.8 (NCH_2_), 50.7 (NCH_2_), 50.4 (NCH_2_), 46.7 (N(CH_3_)_2_), 46.5 (N(CH_3_)_2_), 29.1 (2CH_2_), 26.9 (2CH_2_); MALDI-TOF MS m/z [M + H^+^] Calc for C_35_H_49_N_6_O: 569.397, Found: 569.332.

**6-Methoxy-2,4-bis{4-[(3-dimethylaminopropyl)aminomethyl]phenyl}quinazoline (3i)**

Yellow oil (97%); ^1^H NMR δ (300 MHz, CDCl_3_) 8.55 (d, 2H, *J*= 8.25 Hz, H-2′′ and H-6′′), 7.96 (d, 1H, *J*= 9.30 Hz, H-8), 7.80 (d, 2H, *J*= 8.25 Hz, H-2′ and H-6′), 7.48 (d, 2H, *J*= 8.25 Hz, H-3′′ and H-5′′), 7.43 (dd, 1H, *J*= 9.30 and 2.80 Hz, H-7), 7.40 (d, 2H, *J*= 8.25 Hz, H-3′ and H-5′), 7.31 (d, 1H, *J*= 2.80 Hz, H-5), 3.85 (s, 2H, NCH_2_), 3.81 (s, 2H, NCH_2_), 3.75 (s, 3H, CH_3_O), 2.69 (t, 2H, *J*= 7.10 Hz, NCH_2_), 2.64 (t, 2H, *J*= 7.10 Hz, NCH_2_), 2.30 (t, 2H, *J*= 7.10 Hz, NCH_2_), 2.26 (t, 2H, *J*= 7.10 Hz, NCH_2_), 2.18 (s, 6H, N(CH_3_)_2_), 2.16 (s, 6H, N(CH_3_)_2_), 1.72–1.59 (m, 4H, 2CH_2_); 13C NMR δ (75 MHz, CDCl_3_) 167.6 (C-6), 159.8 (C-2), 159.2 (C-4), 149.4 (C-8a), 143.9 (C-4′), 143.8 (C-4′′), 138.4 (C-1′), 137.9 (C-1′′), 131.9 (C-8), 131.3 (C-3′ and C-5′), 129.7 (C-3′′ and C-5′′), 129.5 (C-2′ and C-6′, C-2′′ and C-6′′), 127.4 (C-7), 123.6 (C-4a), 105.7 (C-5), 59.4 (2NCH_2_), 56.9 (CH_3_O), 55.1 (2NCH_2_), 49.4 (NCH_2_), 49.2 (NCH_2_), 46.9 (2N(CH_3_)_2_), 29.4 (2CH_2_); MALDI-TOF MS m/z [M + H]^+^ Calc for C_33_H_45_N_6_O: 541.365, Found: 541.376.

**6-Methoxy-2,4-bis{4-[(2-dimethylaminoethyl)aminomethyl]phenyl}quinazoline (3j)**

Yellow oil (97%); ^1^H NMR δ (300 MHz, CDCl_3_) 8.56 (d, 2H, *J*= 8.15 Hz, H-2′′ and H-6′′), 7.97 (d, 1H, *J*= 9.00 Hz, H-8), 7.82 (d, 2H, *J*= 8.15 Hz, H-2′ and H-6'), 7.51 (d, 2H, *J*= 8.15 Hz, H-3′′ and H-5′′), 7.44 (dd, 1H, *J*= 9.00 and 2.60 Hz, H-7), 7.42 (d, 2H, *J*= 8.15 Hz, H-3′ and H-5′), 7.32 (d, 1H, *J*= 2.60 Hz, H-5), 3.89 (s, 2H, NCH_2_), 3.84 (s, 2H, NCH_2_), 3.76 (s, 3H, CH_3_O), 2.73 (t, 2H, *J*= 7.10 Hz, NCH_2_), 2.66 (t, 2H, *J*= 6.00 Hz, NCH_2_), 2.43 (t, 2H, *J*= 6.00 Hz, NCH_2_), 2.39 (t, 2H, *J*= 6.00 Hz, NCH_2_), 2.19 (s, 6H, N(CH_3_)_2_), 2.15 (s, 6H, N(CH_3_)_2_); 13C NMR δ (75 MHz, CDCl_3_) 167.7 (C-6), 159.8 (C-2), 159.3 (C-4), 149.4 (C-8a), 143.9 (C-4′), 143.7 (C-4′′), 138.4 (C-1′), 137.9 (C-1′′), 131.9 (C-8), 131.3 (C-3′ and C-5′), 129.7 (C-3′′ and C-5′′, C-2′ and C-6′), 129.6 (C-2′′ and C-6′′), 127.5 (C-7), 123.7 (C-4a), 105.6 (C-5), 60.4 (2NCH_2_), 56.9 (CH_3_O), 55.2 (2NCH_2_), 48.2 (NCH_2_), 47.8 (NCH_2_), 46.9 (2 N(CH_3_)_2_); MALDI-TOF MS m/z [M + H]^+^ Calc for C_31_H_41_N_6_O: 513.334, Found: 513.316.

**6-Methoxy-2,4-bis{4-[(3–(4-methylpiperazin-1-yl)propyl)aminomethyl]phenyl}quinazoline (3k)**

Yellow oil (97%); ^1^H NMR δ (300 MHz, CDCl_3_) 8.54 (d, 2H, *J*= 8.30 Hz, H-2′′ and H-6′′), 7.97 (d, 1H, *J*= 9.20 Hz, H-8), 7.81 (d, 2H, *J*= 8.30 Hz, H-2′ and H-6′), 7.49 (d, 2H, *J*= 8.30 Hz, H-3′′ and H-5′′), 7.47 (dd, 1H, *J*= 9.20 and 2.80 Hz, H-7), 7.39 (d, 2H, *J*= 8.30 Hz, H-3′ and H-5′), 7.33 (d, 1H, *J*= 2.80 Hz, H-5), 3.86 (s, 2H, NCH_2_), 3.80 (s, 2H, NCH_2_), 3.77 (s, 3H, CH_3_O), 2.70 (t, 2H, *J*= 6.75 Hz, NCH_2_), 2.63 (t, 2H, *J*= 6.75 Hz, NCH_2_), 2.50–2.30 (m, 20H, 2NCH_2_ and 8NCH_2_ pip.), 2.22 (s, 3H, NCH_3_), 2.21 (s, 3H, NCH_3_), 1.75–1.61 (m, 4H, 2CH_2_); 13C NMR δ (75 MHz, CDCl_3_) 167.6 (C-6), 159.8 (C-2), 159.3 (C-4), 149.5 (C-8a), 143.9 (C-4′), 143.8 (C-4′′), 138.4 (C-1'), 137.9 (C-1′′), 131.9 (C-8), 131.3 (C-3′ and C-5′), 129.7 (C-3′′ and C-5′′, C-2′ and C-6′), 129.5 (C-2′′ and C-6′′), 127.4 (C-7), 123.7 (C-4a), 105.7 (C-5), 58.4 (2NCH_2_), 56.9 (CH_3_O), 56.5 (2NCH_2_ pip.), 55.1 (2NCH_2_), 54.6 (2NCH_2_ pip.), 49.7 (NCH_2_), 49.4 (NCH_2_), 47.4 (2NCH_3_), 28.4 (2CH_2_); MALDI-TOF MS m/z [M + H]^+^ Calc for C_39_H_55_N_8_O: 651.450, Found: 651.394.

**6-Methoxy-2,4-bis{4-[(2–(4-methylpiperazin-1-yl)ethyl)aminomethyl]phenyl}quinazoline (3l)**

Yellow oil (96%); ^1^H NMR δ (300 MHz, CDCl_3_) 8.59 (d, 2H, *J*= 8.20 Hz, H-2′′ and H-6′′), 8.04 (d, 1H, *J*= 9.20 Hz, H-8), 7.87 (d, 2H, *J*= 8.20 Hz, H-2′ and H-6′), 7.55 (d, 2H, *J*= 8.30 Hz, H-3′′ and H-5′′), 7.52 (dd, 1H, *J*= 9.20 and 2.80 Hz, H-7), 7.44 (d, 2H, *J*= 8.20 Hz, H-3' and H-5'), 7.39 (d, 1H, *J*= 2.80 Hz, H-5), 3.94 (s, 2H, NCH_2_), 3.88 (s, 2H, NCH_2_), 3.84 (s, 3H, CH_3_O), 2.79 (t, 2H, *J*= 6.00 Hz, NCH_2_), 2.71 (t, 2H, *J*= 6.00 Hz, NCH_2_), 2.58–2.44 (m, 20H, 2NCH_2_ and 8NCH_2_ pip.), 2.28 (s, 3H, NCH_3_), 2.27 (s, 3H, NCH_3_); 13C NMR δ (75 MHz, CDCl_3_) 167.7 (C-6), 159.8 (C-2), 159.3 (C-4), 149.5 (C-8a), 143.7 (C-4′, C-4′′), 138.5 (C-1′), 138.0 (C-1′′), 132.0 (C-8), 131.3 (C-3′ and C-5′), 129.7 (C-3′′ and C-5′′, C-2′ and C-6′, C-2′′ and C-6′′), 127.5 (C-7), 123.7 (C-4a), 105.7 (C-5), 59.1 (2NCH_2_), 57.0 (CH_3_O), 56.5 (2NCH_2_ pip.), 55.1 (NCH_2_), 54.8 (NCH_2_), 54.5 (2NCH_2_ pip.), 47.4 (2NCH_2_), 47.2 (NCH_2_), 46.8 (NCH_3_); MALDI-TOF MS m/z [M + H]^+^ Calc for C_37_H_53_N_8_O: 625.434, Found: 625.593.

**7-Methoxy-2,4-bis{4-[(4-dimethylaminobutyl)aminomethyl]phenyl}quinazoline (3 m)**

Pale yellow oil (89%); ^1^H NMR δ (300 MHz, CDCl_3_) 8.61 (d, 2H, *J*= 7.95 Hz, H-2′′ and H-6′′), 7.99 (d, 1H, *J*= 9.15 Hz, H-5), 7.82 (d, 2H, *J*= 7.95 Hz, H-2′ and H-6′), 7.53 (d, 2H, *J*= 7.95 Hz, H-3′′ and H-5′′), 7.46 (d, 2H, *J*= 7.95 Hz, H-3′ and H-5′), 7.41 (d, 1H, *J*= 2.10 Hz, H-8), 7.13 (dd, 1H, *J*= 9.15 and 2.10 Hz, H-6), 3.99 (s, 3H, CH_3_O), 3.91 (s, 2H, NCH_2_), 3.87 (s, 2H, NCH_2_), 2.70 (t, 2H, *J*= 6.60 Hz, NCH_2_), 2.66 (t, 2H, *J*= 6.60 Hz, NCH_2_), 2.30–2.25 (m, 4H, 2NCH_2_), 2.21 (s, 6H, N(CH_3_)_2_), 2.19 (s, 6H, N(CH_3_)_2_), 1.57–1.52 (m, 8H, 4CH_2_); 13C NMR δ (75 MHz, CDCl_3_) 168.3 (C-7), 165.1 (C-2), 162.1 (C-4), 155.8 (C-8a), 143.7 (C-4′, C-4′′), 138.7 (C-1′), 137.9 (C-1′′), 131.6 (C-3′ and C-5′), 130.1 (C-3′′ and C-5′′), 129.7 (C-5, C-2' and C-6', C-2′′ and C-6′′), 121.4 (C-6), 118.4 (C-4a), 108.0 (C-8), 60.9 (2NCH_2_), 57.1 (CH_3_O), 55.0 (2NCH_2_), 50.6 (NCH_2_), 50.1 (NCH_2_), 46.7 (2 N(CH_3_)_2_), 29.7 (2CH_2_), 26.9 (2CH_2_); MALDI-TOF MS m/z [M + H]^+^ Calc for C_35_H_49_N_6_O: 569.397, Found: 569.455.

**7-Methoxy-2,4-bis{4-[(3-dimethylaminopropyl)aminomethyl]phenyl}quinazoline (3n)**

Yellow oil (96%); ^1^H NMR δ (300 MHz, CDCl_3_) 8.59 (d, 2H, *J*= 8.20 Hz, H-2'' and H-6''), 7.98 (d, 1H, *J*= 9.20 Hz, H-5), 7.80 (d, 2H, *J*= 8.20 Hz, H-2′ and H-6'), 7.51 (d, 2H, *J*= 8.20 Hz, H-3′′ and H-5′′), 7.44 (d, 2H, *J*= 8.20 Hz, H-3′ and H-5′), 7.40 (d, 1H, *J*= 2.50 Hz, H-8), 7.11 (dd, 1H, *J*= 9.20 and 2.50 Hz, H-6), 3.97 (s, 3H, CH_3_O), 3.89 (s, 2H, NCH_2_), 3.85 (s, 2H, NCH_2_), 2.71 (t, 2H, *J*= 7.05 Hz, NCH_2_), 2.67 (t, 2H, *J*= 7.05 Hz, NCH_2_), 2.33 (t, 2H, *J*= 7.05 Hz, NCH_2_), 2.30 (t, 2H, *J*= 7.05 Hz, NCH_2_), 2.22 (s, 6H, N(CH_3_)_2_), 2.20 (s, 6H, N(CH_3_)_2_), 1.76–1.63 (m, 4H, 2CH_2_); 13C NMR δ (75 MHz, CDCl_3_) 168.3 (C-7), 165.0 (C-2), 162.1 (C-4), 155.8 (C-8a), 144.2 (C-4′), 143.8 (C-4′′), 138.5 (C-1′), 137.8 (C-1′′), 131.6 (C-3′ and C-5′), 130.0 (C-3′′ and C-5′′), 129.7 (C-5), 129.5 (C-2′ and C-6′, C-2′′ and C-6′′), 121.4 (C-6), 118.4 (C-4a), 107.8 (C-8), 59.4 (NCH_2_), 59.3 (NCH_2_), 57.1 (CH_3_O), 55.2 (NCH_2_), 55.1 (NCH_2_), 49.3 (NCH_2_), 49.2 (NCH_2_), 46.9 (N(CH_3_)_2_), 46.8 (N(CH_3_)_2_), 29.4 (CH_2_), 29.3 (CH_2_); MALDI-TOF MS m/z [M + H]^+^ Calc for C_33_H_45_N_6_O: 541.36, Found: 541.476.

**7-Methoxy-2,4-bis{4-[(2-dimethylaminoethyl)aminomethyl]phenyl}quinazoline (3o)**

Yellow oil (97%); ^1^H NMR δ (300 MHz, CDCl_3_) 8.60 (d, 2H, *J*= 8.25 Hz, H-2'' and H-6''), 7.98 (d, 1H, *J*= 9.20 Hz, H-5), 7.79 (d, 2H, *J*= 8.25 Hz, H-2' and H-6'), 7.51 (d, 2H, *J*= 8.25 Hz, H-3'' and H-5''), 7.45 (d, 2H, *J*= 8.25 Hz, H-3' and H-5'), 7.38 (d, 1H, *J*= 2.50 Hz, H-8), 7.10 (dd, 1H, *J*= 9.20 and 2.50 Hz, H-6), 3.96 (s, 3H, CH_3_O), 3.90 (s, 2H, NCH_2_), 3.87 (s, 2H, NCH_2_), 2.72 (t, 2H, *J*= 6.10 Hz, NCH_2_), 2.68 (t, 2H, *J*= 6.10 Hz, NCH_2_), 2.44 (t, 2H, *J*= 6.10 Hz, NCH_2_), 2.41 (t, 2H, *J*= 6.10 Hz, NCH_2_), 2.20 (s, 6H, N(CH_3_)_2_), 2.17 (s, 6H, N(CH_3_)_2_); 13C NMR δ (75 MHz, CDCl_3_) 168.3 (C-7), 165.0 (C-2), 162.1 (C-4), 155.8 (C-8a), 144.3 (C-4′), 143.8 (C-4′′), 138.4 (C-1′), 137.8 (C-1′′), 131.5 (C-3′ and C-5′), 130.0 (C-3′′ and C-5′′), 129.6 (C-5, C-2′ and C-6′, C-2′′ and C-6′′), 121.3 (C-6), 118.4 (C-4a), 108.0 (C-8), 60.5 (2NCH_2_), 57.1 (CH_3_O), 55.2 (2NCH_2_), 48.1 (NCH_2_), 47.7 (NCH_2_), 46.9 (2 N(CH_3_)_2_); MALDI-TOF MS m/z [M + H]^+^ Calc for C_31_H_41_N_6_O: 513.334, Found: 513.456.

**7-Methoxy-2,4-bis{4-[(4–(4-methylpiperazin-1-yl)butyl)aminomethyl]phenyl}quinazoline (3p)**

Yellow oil (97%); ^1^H NMR δ (300 MHz, CDCl_3_) 8.52 (d, 2H, *J*= 8.30 Hz, H-2′′ and H-6′′), 7.89 (d, 1H, *J*= 9.30 Hz, H-5), 7.72 (d, 2H, *J*= 8.30 Hz, H-2′ and H-6′), 7.44 (d, 2H, *J*= 8.30 Hz, H-3′′ and H-5′′), 7.38 (d, 2H, *J*= 8.30 Hz, H-3′ and H-5′), 7.30 (d, 1H, *J*= 2.30 Hz, H-8), 7.02 (dd, 1H, *J*= 9.30 and 2.30 Hz, H-6), 3.88 (s, 3H, CH_3_O), 3.81 (s, 2H, NCH_2_), 3.78 (s, 2H, NCH_2_), 2.60 (t, 2H, *J*= 6.60 Hz, NCH_2_), 2.57 (t, 2H, *J*= 6.60 Hz, NCH_2_), 2.40–2,21 (m, 20H, 2NCH_2_ and 8NCH_2_ pip.), 2.17 (s, 3H, NCH_3_), 2.16 (s, 3H, NCH_3_), 1.48–1.44 (m, 8H, 4CH_2_); 13C NMR δ (75 MHz, CDCl_3_) 168.1 (C-7), 164.9 (C-2), 161.8 (C-4), 155.7 (C-8a), 144.5 (C-4′), 143.2 (C-4′′), 138.5 (C-1′), 137.8 (C-1′′), 131.5 (C-3′ and C-5′), 130.0 (C-3′′ and C-5′′), 129.6 (C-5, C-2′ and C-6′, C-2′′ and C-6′′), 121.3 (C-6), 118.2 (C-4a), 107.9 (C-8), 59.7 (2NCH_2_), 57.0 (CH_3_O), 56.4 (2NCH_2_ pip.), 54.8 (2NCH_2_), 54.5 (2NCH_2_ pip.), 50.5 (NCH_2_), 50.4 (NCH_2_), 47.3 (2NCH_3_), 29.2 (2CH_2_), 26.0 (2CH_2_); MALDI-TOF MS m/z [M + H]^+^ Calc for C_41_H_59_N_8_O: 679.481, Found: 679.506.

**7-Methoxy-2,4-bis{4-[(3–(4-methylpiperazin-1-yl)propyl)aminomethyl]phenyl}quinazoline (3q)**

Yellow oil (97%); ^1^H NMR δ (300 MHz, CDCl_3_) 8.55 (d, 2H, *J*= 8.20 Hz, H-2′′ and H-6′′), 7.92 (d, 1H, *J*= 9.10 Hz, H-5), 7.75 (d, 2H, *J*= 8.20 Hz, H-2′ and H-6′), 7.46 (d, 2H, *J*= 8.20 Hz, H-3′′ and H-5′′), 7.39 (d, 2H, *J*= 8.20 Hz, H-3′ and H-5′), 7.34 (d, 1H, *J*= 2.50 Hz, H-8), 7.05 (dd, 1H, *J*= 9.10 and 2.50 Hz, H-6), 3.92 (s, 3H, CH_3_O), 3.84 (s, 2H, NCH_2_), 3.80 (s, 2H, NCH_2_), 2.67 (t, 2H, *J*= 7.05 Hz, NCH_2_), 2.63 (t, 2H, *J*= 7.05 Hz, NCH_2_), 2.38–2.34 (m, 20H, 2NCH_2_ and 8NCH_2_ pip.), 2.21 (s, 3H, NCH_3_), 2.20 (s, 3H, NCH_3_), 1.71–1.63 (m, 4H, 2CH_2_); 13C NMR δ (75 MHz, CDCl_3_) 168.3 (C-7), 165.0 (C-2), 162.2 (C-4), 155.8 (C-8a), 144.3 (C-4′), 143.9 (C-4′′), 138.4 (C-1′), 137.8 (C-1′′), 131.5 (C-3′ and C-5′), 130.0 (C-3′′ and C-5′′), 129.7 (C-5), 129.4 (C-2′ and C-6′, C-2′′ and C-6′′), 121.3 (C-6), 118.3 (C-4a), 107.9 (C-8), 58.4 (NCH_2_), 58.3 (NCH_2_), 57.1 (CH_3_O), 56.5 (2NCH_2_ pip.), 55.1 (NCH_2_), 55.0 (NCH_2_), 54.6 (2NCH_2_ pip.), 49.5 (NCH_2_), 49.4 (NCH_2_), 47.4 (2NCH_3_), 28.4 (CH_2_), 28.3 (CH_2_); MALDI-TOF MS m/z [M + H]^+^ Calc for C_39_H_55_N_8_O: 651.450, Found: 651.654.

**7-Methoxy-2,4-bis{4-[(2–(4-methylpiperazin-1-yl)ethyl)aminomethyl]phenyl}quinazoline (3r)**

Yellow oil (89%); ^1^H NMR δ (300 MHz, CDCl_3_) 8.62 (d, 2H, *J*= 8.40 Hz, H-2′′ and H-6′′), 7.99 (d, 1H, *J*= 9.30 Hz, H-5), 7.83 (d, 2H, *J*= 8.40 Hz, H-2′ and H-6′), 7.53 (d, 2H, *J*= 8.40 Hz, H-3′′and H-5′′), 7.46 (d, 2H, *J*= 8.40 Hz, H-3′ and H-5′), 7.43 (d, 1H, *J*= 2.70 Hz, H-8), 7.14 (dd, 1H, *J*= 9.30 and 2.70 Hz, H-6), 4.01 (s, 3H, CH_3_O), 3.93 (s, 2H, NCH_2_), 3.90 (s, 2H, NCH_2_), 2.77 (t, 2H, *J*= 6.90 Hz, NCH_2_), 2.72 (t, 2H, *J*= 6.90 Hz, NCH_2_), 2.56–2,41 (m, 20H, 2NCH_2_ and 8NCH_2_ pip.), 2.29 (s, 3H, NCH_3_), 2.27 (s, 3H, NCH_3_); 13C NMR δ (75 MHz, CDCl_3_) 168.4 (C-7), 165.1 (C-2), 162.1 (C-4), 155.8 (C-8a), 144.3 (C-4′), 143.7 (C-4′′), 138.5 (C-1′), 137.9 (C-1′′), 131.6 (C-3′ and C-5′), 130.1 (C-3′′ and C-5′′), 129.7 (C-5), 129.6 (C-2′ and C-6′, C-2′′and C-6′′), 121.4 (C-6), 118.4 (C-4a), 108.0 (C-8), 59.1 (2NCH_2_), 57.1 (CH_3_O), 56.6 (2NCH_2_ pip.), 55.1 (2NCH_2_), 54.5 (2NCH_2_ pip.), 47.4 (2NCH_3_), 47.2 (NCH_2_), 46.9 (NCH_2_); MALDI-TOF MS m/z [M + H]^+^ Calc for C_37_H_51_N_8_O: 623.418, Found: 623.325.

**2,4-Bis{3-[(3-dimethylaminopropyl)aminomethyl]phenyl}quinazoline (3 s)**

Yellow oil (60%); ^1^H NMR δ (300 MHz, CDCl_3_) 8.21 (dd, 1H, *J*= 8.20 and 1.20 Hz, H-8), 8.17–8.13 (m, 2H, H-6′ and H-2′), 7.91 (dd, 1H, *J*= 8.20 and 1.20 Hz, H-5), 7.80 (dd, 1H, *J*= 1.40 and 1.40 Hz, H-2′′), 7.72 (ddd, 1H, *J*= 8.20, 7.00 and 1.20 Hz, H-7), 7.55–7.43 (m, 6H, H-6, H-6′′, H-4′, H-4′′, H-5′ and H-5′′), 3.90 (s, 2H, NCH_2_), 3.87 (s, 2H, NCH_2_), 2.73–2.64 (m, 4H, 2NCH_2_), 2.45–2.32 (m, 4H, 2NCH_2_), 2.28 (s, 6H, N(CH_3_)_2_), 2.27 (s, 6H, N(CH_3_)_2_), 1.61–1.52 (m, 4H, 2CH_2_); 13C NMR δ (75 MHz, CDCl_3_) 158.1 (C-2), 150.3 (C-4), 150.2 (C-8a), 143.1 (C-3′′), 142.2 (C-3′), 139.7 (C-1′′), 138.4 (C-1′), 131.4 (C-6′), 131.0 (C-6′′), 130.9 (C-2′ and C-2′′), 129.9 (C-4′ and C-4′′), 129.7 (C-5′ and C-5′′), 129.0 (C-8), 127.6 (C-7), 127.1 (C-4a), 127.0 (C-5), 120.6 (C-6), 59.9 (NCH_2_), 56.5 (NCH_2_), 55.1 (NCH_2_), 54.6 (NCH_2_), 50.8 (NCH_2_), 50.6 (NCH_2_), 47.4 (NCH_3_), 29.5 (CH_2_), 26.1 (CH_2_); MALDI-TOF MS m/z [M + H]^+^ Calc for C_32_H_43_N_6_: 511.355, Found: 511.755.

**2,4-Bis{3-[(3–(4-methylpiperazin-1-yl)propyl)aminomethyl]phenyl}quinazoline(3t)**

Pale-yellow oil (88%); ^1^H NMR δ (300 MHz, CDCl_3_) 8.56–8.52 (m, 2H, H-6′ and H-2′), 8.10 (dd, 1H, *J*= 8.40 and 1.20 Hz, H-8), 8.06 (dd, 1H, *J*= 8.40 and 1.20 Hz, H-5), 7.84 (ddd, 1H, *J*= 8.40, 7.20 and 1.20 Hz, H-7), 7.81 (dd, 1H, *J*= 1.40 and 1.40 Hz, H-2′′), 7.73–7.69 (m, 1H, H-6′′), 7.52–7.43 (m, 5H, H-6, H-4′, H-4′′, H-5′ and H-5′′), 3.90 (s, 2H, NCH_2_), 3.88 (s, 2H, NCH_2_), 2.70 (t, 2H, *J*= 6.60 Hz, NCH_2_), 2.68 (t, 2H, *J*= 6.60 Hz, NCH_2_), 2.19 (s, 3H, NCH_3_), 2.17 (s, 3H, NCH_3_), 1.72–1.66 (m, 4H, 2CH_2_); 13C NMR δ (75 MHz, CDCl_3_) 169.8 (C-2), 161.5 (C-4), 153.2 (C-8a), 142.4 (C-3′′), 142.0 (C-3′), 139.7 (C-1′′), 139.0 (C-1′), 134.9 (C-8), 131.7 (C-6′), 131.0 (C-6′′ and C-7), 130.4(C-2′ and C-2′′), 130.0 (C-4′ and C-4′′), 129.6 (C-5′′), 128.8 (C-5′), 128.4 (C-5 and C-6), 123.1 (C-4a), 58.4 (NCH_2_), 56.5 (NCH_2_), 55.4 (NCH_2_), 55.2 (NCH_2_), 54.6 (NCH_2_), 49.6 (NCH_2_), 47.3 (NCH_3_), 28.3 (CH_2_); MALDI-TOF MS m/z [M + H]^+^ Calc for C_38_H_53_N_8_: 621.439, Found: 621.478.

**2,4-Bis{3-[(3-morpholinopropyl)aminomethyl]phenyl}quinazoline (3 u)**

Yellow oil (82%); ^1^H NMR δ (300 MHz, CDCl_3_) 8.58 (dd, 1H, *J*= 1.50 and 1.50 Hz, H-2′), 8.56 (ddd, 1H, *J*= 7.80, 1.50 and 1.50 Hz, H-6′), 8.13 (dd, 1H, *J*= 8.70 and 1.10 Hz, H-8), 8.08 (dd, 1H, *J*= 8.70 and 1.10 Hz, H-5), 7.87 (ddd, 1H, *J*= 8.70, 7.20 and 1.10 Hz, H-7), 7.81 (dd, 1H, *J*= 1.50 and 1.50 Hz, H-2′′), 7.76–7.71 (m, 1H, H-6′′), 7.55–7.45 (m, 5H, H-6, H-4′, H-4′′, H-5′ and H-5′′), 3.93 (s, 2H, NCH_2_), 3.91 (s, 2H, NCH_2_), 3.69 (t, 4H, *J*= 4.80 Hz, 2OCH_2_), 3.64 (t, 4H, *J*= 4.80 Hz, 2OCH_2_), 2.75–2.66 (m, 4H, 2NCH_2_), 2.42–2.31 (m, 12H, 2NCH_2_ and 4NCH_2_morph.), 1.71 (qt, 4H, *J*= 6.90 Hz, 2CH_2_); 13C NMR δ (75 MHz, CDCl_3_) 169.8 (C-2), 161.6 (C-4), 153.3 (C-8a), 142.4 (C-3′′), 142.1 (C-3′), 139.7 (C-1′′), 139.1 (C-1′), 135.0 (C-8), 131.7 (C-6′), 131.1 (C-6′′), 131.0(C-7), 130.5 (C-2′′), 130.2 (C-2′), 130.0 (C-4′′), 129.9 (C-4′), 129.6 (C-5′′), 128.8 (C-5′), 128.4 (C-5 and C-6), 123.1 (C-4a), 63.3 (OCH_2_), 58.8 (NCH_2_), 58.7 (NCH_2_), 55.5 (NCH_2_), 55.3 (NCH_2_), 55.1 (NCH_2_), 49.4 (NCH_2_), 28.1 (CH_2_), 28.0 (CH_2_); MALDI-TOF MS m/z [M + H]^+^ Calc for C_36_H_47_N_6_O_2_: 595.376, Found: 595.398.

**2,4-Bis{3-[(2-morpholinoethyl)aminomethyl]phenyl}quinazoline (3v)**

Yellow oil (66%); ^1^H NMR δ (300 MHz, CDCl_3_) 8.60–8.56 (m, 2H, H-2′ and H-6′), 8.14 (dd, 1H, *J*= 8.40 and 1.20 Hz, H-8), 8.08 (dd, 1H, *J*= 8.40 and 1.20 Hz, H-5), 7.88 (ddd, 1H, *J*= 8.40, 7.20 and 1.20 Hz, H-7), 7.82 (dd, 1H, *J*= 1.40 and 1.40 Hz, H-2′′), 7.76–7.72 (m, 1H, H-6′′), 7.56–7.46 (m, 5H, H-6, H-4′, H-4′′, H-5′ and H-5′′), 3.96 (s, 2H, NCH_2_), 3.94 (s, 2H, NCH_2_), 3.67–3.62 (m, 8H, 4OCH_2_), 2.79–2.71 (m, 4H, 2NCH_2_), 2.53–2.36 (m, 12H, 2NCH_2_ and 4NCH_2_morph.); 13C NMR δ (75 MHz, CDCl_3_) 169.8 (C-2), 161.5 (C-4), 153.3 (C-8a), 142.4 (C-3′′), 142.1 (C-3′), 139.7 (C-1′′), 139.1 (C-1′), 135.0 (C-8), 131.8 (C-6′), 131.0 (C-6′′), 130.5 (C-7), 130.1 (C-2′ and C-2′′), 130.0 (C-4′ and C-4′′), 129.6 (C-5′′), 128.9 (C-5′), 128.4 (C-5 and C-6), 123.1 (C-4a), 68.3 (OCH_2_), 59.6 (NCH_2_), 55.1 (NCH_2_), 46.8 (NCH_2_), 46.5 (NCH_2_); MALDI-TOF MS m/z [M + H]^+^ Calc for C_34_H_43_N_6_O_2_: 567.345, Found: 567.377.

### General procedure for 2,4-bis[(substituted-aminomethyl)phenyl]quinolines (1a–t ⋅ m(COOH)_2_), 1,3-bis[(substituted-aminomethyl)phenyl]isoquinolines (2a–l ⋅ m(COOH)_2_), and 2,4-bis[(substituted-aminomethyl)phenyl]quinazolines oxalate salts (3a–v ⋅ m(COOH)_2_)

To a solution of compounds **1–3** (0.3 mmol) in isopropanol (11 ml) was added oxalic acid (2.4 mmol, 8 eq.). The reaction mixture was heated under reflux for 30 min. The precipitate was filtered, washed with isopropanol then with diethyl ether, and dried under reduced pressure to give the oxalate salts of **1–3**.

### *In vitro* antiplasmodial activity

The *in vitro* antiplasmodial activities were tested over concentrations ranging from 39 to 40 µM against culture-adapted *P. falciparum* reference strains 3D7 and W2. The former strain is susceptible to CQ but displays a decreased susceptibility to MQ; the latter is considered resistant to CQ. The parasites were cultivated in RPMI medium (Sigma-Aldrich, Lyon, France) supplemented with 0.5% Albumax I (Life Technologies Corporation, Paisley, UK), hypoxanthine (Sigma-Aldrich), and gentamicin (Sigma-Aldrich) with human erythrocytes and were incubated at 37 °C in a candle jar, as described previously^43^ .The *P. falciparum* drug susceptibility test was carried out in 96-well flat-bottom sterile plates in a final volume of 250 µl. After 48 h incubation period with the drugs, quantities of DNA in treated and control cultures of parasites in human erythrocytes were quantified using the SYBR Green I (Sigma-Aldrich) fluorescence-based method^44^^,^^45^. Briefly, after incubation, plates were frozen at −20 °C until use. Plates were then thawed for 2 h at room temperature, and 100 µl of each homogenised culture was transferred to a well of a 96-well flat-bottom sterile black plate (Nunc, Inc., Rochester, NY) that contained 100 µl of the SYBR Green I lysis buffer (2xSYBR Green, 20 mM Tris base pH 7.5, 5 mM EDTA, 0.008% w/v saponin, 0.08% w/v Triton X-100). Negative controls treated with solvent (typically DMSO or H_2_O), and positive controls (CQ and MQ) were added to each set of experiments. Plates were incubated for 1 h at room temperature and then read on a fluorescence plate reader (Tecan, Grödig, Austria) using excitation and emission wavelengths of 485 and 535 nm, respectively. IC_50_ values were calculated by non-linear regression analysis of data from dose-response curves, using TableCurve 2D version 5.0 software (Systat Software, San Jose, CA). IC_50_ values are reported as means calculated from three independent experiments^46^.

### *In vitro* antileishmanial activity

*L. donovani* (MHOM/IN/00/DEVI) used in this study was provided by the CNR *Leishmania* (Montpellier, France). The effects of the tested compounds on the growth of *L. donovani* (MHOM/IN/00/DEVI) promastigotes were assessed by MTT assay^47^. Briefly, promastigotes in log-phase in Schneider’s medium supplemented with 20% foetal calf serum (FCS), 2 mM L-glutamine and antibiotics (100 U/ml penicillin and 100 µg/ml streptomycin), were incubated at an average density of 10^6^ parasites/ml in sterile 96-well plates with various concentrations of compounds dissolved in DMSO (final concentration less than 0.5% v/v), in duplicate. Appropriate controls treated with DMSO and amphotericin B (reference drug purchased from Sigma-Aldrich) were added to each set of experiments. After 72 h incubation period at 27 °C, parasite metabolic activity was determined. Each well was microscopically examined for precipitate formation. To each well was added 20 µl of 5 mg/ml MTT [3–(4,5-dimethylthiazol-2-yl)-2,5-diphenyltetrazolium bromide)] solution followed by 4 h incubation time. The enzyme reaction was stopped by addition of 100 µl of 50% isopropanol/10% sodium dodecyl sulfate^48^. Plates were vigorously shaken (300 rpm) for 10 min, and the absorbance was measured at 570 nm in a BIO-TEK ELx808 Absorbance Microplate Reader. The IC_50_ was defined as the concentration of drug required to inhibit by 50% of the metabolic activity of *L. donovani* promastigotes compared to the control. IC_50_ of the parasite’s growth (half maximal inhibitory concentration or IC_50_ values) were then calculated from the obtained experimental results using a previously described regression programme^46^.

### *In vitro* antitrypanosomal activity

The effects of the tested compounds on the growth of *T. brucei brucei* were assessed using an Alamar Blue® assay described by Räz et al.^49^ *T. brucei brucei* AnTat 1.9 (IMTA, Antwerpen, Belgium) was cultured in MEM with Earle’s salts, supplemented according to the protocol of Baltz et al.^50^ with the following modifications: 0.5 mM mercaptoethanol (Sigma Aldrich), 1.5 mM L-cysteine (Sigma Aldrich), 0.05 mM bathocuproine sulphate (Sigma Aldrich), and 20% heat-inactivated horse serum (Gibco, France) at 37 °C and 5% CO_2_. Samples were incubated at an average density of 2000 parasites/well in sterile 96-wells plates (Fisher, France) with various concentrations of compounds dissolved in 0.9% NaCl. All doses were tested in duplicate. Appropriate controls treated with solvents 0.9% NaCl or DMSO or with suramin, pentamidine, eflornithine, and fexinidazole (reference drugs purchased from Sigma Aldrich and Fluorochem, UK) were added to each set of experiments. After 69 h incubation period at 37 °C, 10 µl of the viability marker Alamar Blue (Fisher) was added to each well, and the plates were incubated for 5 h. The plates were read in a PerkinElmer ENSPIRE (Germany) microplate reader using an excitation wavelength of 530 nm and an emission wavelength of 590 nm. The IC_50_ was defined as the concentration of drug necessary to inhibit by 50% the activity of *T. brucei brucei* compared to the control. IC_50_ values were calculated using a nonlinear regression analysis of dose-response curves performed using GraphPad Prism software (La Jolla, CA). IC_50_ values were calculated from three independent experiments.

### Cytotoxicity evaluation

A cytotoxicity evaluation was performed using the method reported by Mosmann^48^ with slight modifications to determine the cytotoxic concentrations 50% (CC_50_) and using doxorubicin as a cytotoxic reference compound. These assays were performed in human HepG2 cells purchased from ATCC (ref HB-8065). These cells are a commonly used human hepatocarcinoma-derived cell line that has characteristics similar to those of primary hepatocytes. These cells express many hepatocyte-specific metabolic enzymes, thus enabling the cytotoxicity of tested product metabolites to be evaluated. Briefly, cells in 100 µL of complete RPMI medium, [RPMI supplemented with 10% FCS, 1% L-glutamine (200 mM), penicillin (100 U/ml), and streptomycin (100 µg/ml)] were inoculated at 37 °C into each well of 96-well plates in a humidified chamber in 6% CO_2_. After 24 h, 100 µl of medium with test compound at various concentrations dissolved in DMSO (final concentration less than 0.5% v/v) were added, and the plates were incubated for 72 h at 37 °C. Duplicate assays were performed for each sample. Each well was microscopically examined for precipitate formation before the medium was aspirated from the wells. After aspiration, 100 µl of MTT solution (0.5 mg/ml in medium without FCS) were then added to each well. Cells were incubated for 2 h at 37 °C. The MTT solution was removed, and DMSO (100 µl) was added to dissolve the resulting blue formazan crystals. Plates were shaken vigorously (300 rpm) for 5 min. The absorbance was measured at 570 nm with 630 nm as reference wavelength in a BIO-TEK ELx808 Absorbance Microplate Reader. DMSO was used as blank and doxorubicin (Sigma Aldrich) as positive control. Cell viability was calculated as percentage of control (cells incubated without compound). The CC_50_ was determined from the dose-response curve using the TableCurve 2D version 5.0 software (Systat Software, San Jose, CA)

### FRET melting experiments

The best bioactive compounds (**1b–c**, **1e–g**, **1s–t**, **2b**, **2e–f**, **2i–j**, **3b**, **3f–j**, **3m,** and **3v**) have been selected for the subsequent FRET melting experiments. These were performed with dual-labelled oligonucleotides mimicking the *Plasmodium* telomeric sequences FPf1T [FAM-5′(GGGTTTA)_3_-GGG3′-TAMRA] and FPf8T [FAM-5′ (GGGTTCA)_3_GGG3′-TAMRA], the *Trypanosoma* 9 and 11 chromosomic sequence FTrypBT (also named FEBR1T) [FAM-5′GGGCAGGGGGTGATGGGGAGGAGCCAGGG3′-TAMRA], the human telomeric sequence F21T [FAM-(GGGTTA)_3_-GGG3′-TAMRA], and the human duplex sequence FdxT [FAM5′-TATAGCTATA-hexaethyleneglycol-TATAGCTATA3′-TAMRA]^51^^,^^52^. The oligonucleotides were pre-folded in 10 mM lithium cacodylate buffer (pH 7.2), with 10 mM KCl and 90 mM LiCl (K^+^condition). The FAM emissions were recorded at 516 nm using a 492-nm excitation wavelength in the absence and presence of a single compound as a function of temperature (25–95 °C) in 96-well microplates by using a Stratagene MX3000P real-time PCR device at a rate of 1 °C·min^−1^. Data were normalised between 0 and 1, and the required temperature for half-denaturation of oligonucleotides corresponding to the emission value of 0.5 was taken as the T_m_. Each experiment was performed in duplicate with 0.2 µM of labelled oligonucleotide and 2 µM of compound under K^+^ condition. For each compound, three independent experiments were carried out.

### Data analysis

The 20 compounds (**1b–c**, **1e–g**, **1s–t**, **2b**, **2e–f**, **2i–j**, **3b**, **3f–j**, **3m,** and **3v**), previously selected for FRET assays due to their IC_50_ best results, have been then submitted to a statistical multivariate analysis in order to check consistent compound types. In particular, a Hierarchical Ascendant Classification (HAC) was performed on the first three principal components (accounting for the 97% of the total variance) of a Principal Component Analysis based on the FPf1T, FPf8T, F21T, and IC_50_ (against the *P. falciparum* 3D7 strain) variables. As well, the same analysis was carried out on FtryBT, F21T,l and IC_50_ (against *T. brucei brucei* strain) variables. To this end, a Ward’s minimum variance clustering based on Euclidean distances was performed, and the number of retained clusters (Q) was chosen according to the growth of inertia, selecting Q in order to maximise the difference in inertia between (Q − 1, Q) and (Q, Q + 1). This analysis was chosen according to the normal data distribution evaluated with the Shapiro-Wilk normality test. All the analyses were performed within the R 3.0.1 programming environment using functions from the “FactoMineR,” and “lmtest” packages^53–55^.

## Results and discussion

### Chemistry

**Scheme 1.**
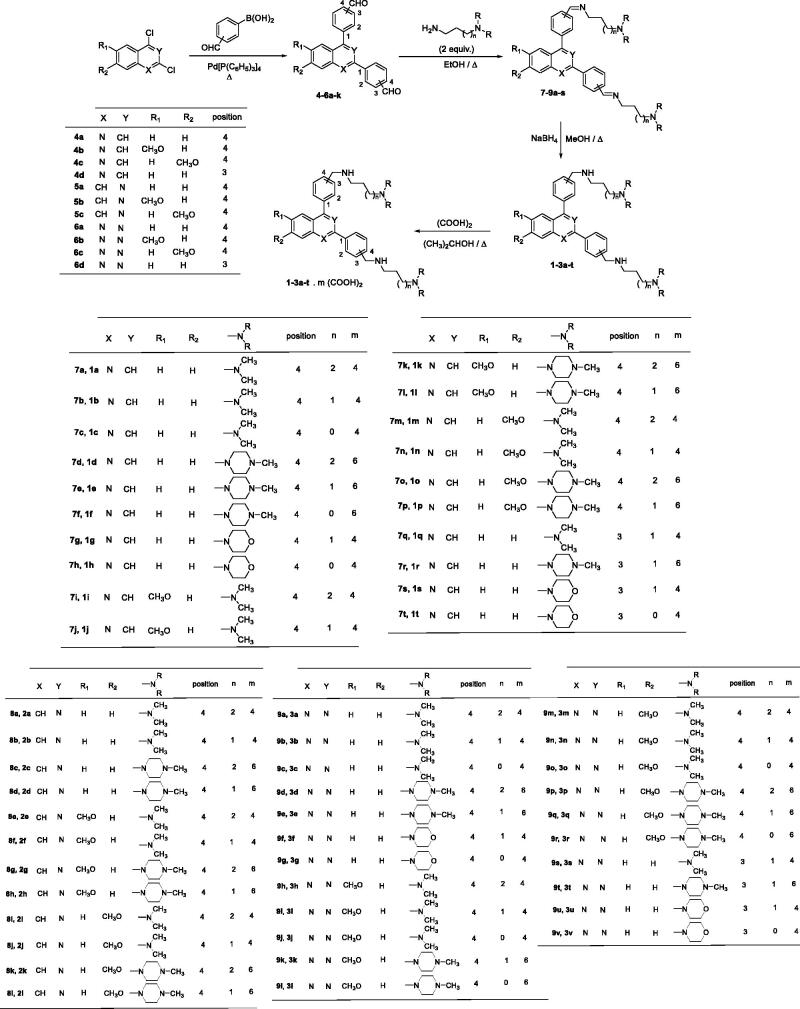
Synthesis of 2,4-bis[(substituted-aminomethyl)phenyl]quinoline, 1,3-bis[(substituted-aminomethyl)phenyl]isoquinoline and 2,4-bis[(substituted-aminomethyl)phenyl]quinazoline derivatives **1–3**.

Novel 2,4-bis[(substituted-aminomethyl)phenyl]quinoline, 1,3-bis[(substituted-aminomethyl)phenyl]isoquinoline and 2,4-bis[(substituted-aminomethyl)phenyl]quinazoline derivatives **1–3** were prepared starting from the commercially substituted 2,4-dichloroquinolines, 1,3-dichloroisoquinolines, or 1,4-dichloroquinazolines (Scheme 1). The intermediate *bis*-(formylphenyl)-quinolines, -isoquinolines, or -quinazolines **4–6** were synthesised by a double-Suzuki-Miyaura cross-coupling reaction of dichloro-derivatives with 3-, or 4-formylphenylboronic acids in the presence of Pd(PPh_3_)_4_ as a catalyst and in the presence of sodium carbonate^35^^,^^56–58^. Condensation of primary amines with these latter dialdehydes **4–6** afforded the di-imines **7–9**, which were immediately reduced into the 2,4-bis[(substituted-aminomethyl)phenyl]quinolines **1a–t**, 1,3-bis[(substituted-aminomethyl)phenyl]isoquinolines **2a–l** and 2,4-bis[(substituted-aminomethyl)phenyl]quinazolines **3a–v** using sodium borohydride as reductive agent in refluxing methanol as previously described by our team^35^. All compounds were extensively characterised (Supplementary Material).

All quinolines, isoquinolines and quinazolines **1–3** were then converted into ammonium oxalate salts by treatment with oxalic acid in refluxing isopropanol. These oxalate salts were found less hygroscopic than the hydrochloride ones and also soluble in water. Table 1 summarises the physical properties of the new synthesised **1–3** oxalates.

**Table 1.** Physical properties of amines **1a–t**, **2a–l,** and **3a–v**.

Compound		Salt^a^	Mp (°C)^b^	% Yield^c^
**1a**	White crystals	4 (COOH)_2_	258–260	72
**1b**	White crystals	4 (COOH)_2_	244–246	75
**1c**	Beige crystals	4 (COOH)_2_	229–231	68
**1d**	White crystals	6 (COOH)_2_	225–227	94
**1e**	White crystals	6 (COOH)_2_	249–251	90
**1f**	White crystals	6 (COOH)_2_	240–242	82
**1g**	Beige crystals	4 (COOH)_2_	202–204	70
**1h**	White crystals	4 (COOH)_2_	235–237	92
**1i**	Yellow crystals	4 (COOH)_2_	227–229	79
**1j**	Pale-yellow crystals	4 (COOH)_2_	224–226	73
**1k**	Pale-yellow crystals	6 (COOH)_2_	235–237	89
**1l**	Yellow crystals	6 (COOH)_2_	223–225	76
**1m**	Pale-yellow crystals	4 (COOH)_2_	208–210	66
**1n**	Pale-yellow crystals	4 (COOH)_2_	237–239	69
**1o**	Pale-yellow crystals	6 (COOH)_2_	237–239	68
**1p**	Pale-yellow crystals	6 (COOH)_2_	245–247	73
**1q**	Beige crystals	4 (COOH)_2_	243–245	80
**1r**	White crystals	6 (COOH)_2_	237–239	78
**1s**	White crystals	4 (COOH)_2_	221–223	76
**1t**	Beige crystals	4 (COOH)_2_	225–227	86
**2a**	Beige crystals	4 (COOH)_2_	184–186	69
**2b**	Yellow crystals	4 (COOH)_2_	186–188	76
**2c**	White crystals	6 (COOH)_2_	181–183	80
**2d**	Pale-yellow crystals	6 (COOH)_2_	217–219	77
**2e**	Yellow crystals	4 (COOH)_2_	157–159	76
**2f**	Yellow crystals	4 (COOH)_2_	178–180	85
**2g**	Pale-yellow crystals	6 (COOH)_2_	172–174	75
**2h**	Yellow crystals	6 (COOH)_2_	195–197	53
**2i**	Pale-yellow crystals	4 (COOH)_2_	135–137	70
**2j**	White crystals	4 (COOH)_2_	171–173	86
**2k**	White crystals	6 (COOH)_2_	216–218	61
**2l**	Beige crystals	6 (COOH)_2_	226–228	66
**3a**	Beige crystals	4 (COOH)_2_	153–155	71
**3b**	White crystals	4 (COOH)_2_	203–205	73
**3c**	Pale-yellow crystals	4 (COOH)_2_	203–205	72
**3d**	White crystals	6 (COOH)_2_	223–225	86
**3e**	Beige crystals	6 (COOH)_2_	229–231	92
**3f**	Beige crystals	4 (COOH)_2_	183–185	80
**3g**	Beige crystals	4 (COOH)_2_	195–197	79
**3h**	Pale-yellow crystals	4 (COOH)_2_	156–158	66
**3i**	Pale-yellow crystals	4 (COOH)_2_	243–245	68
**3j**	Pale-yellow crystals	4 (COOH)_2_	233–235	72
**3k**	Pale-yellow crystals	6 (COOH)_2_	227–229	76
**3l**	Pale-yellow crystals	6 (COOH)_2_	229–231	49
**3m**	Yellow crystals	4 (COOH)_2_	216–218	30
**3n**	White crystals	4 (COOH)_2_	223-225	75
**3o**	Pale-yellow crystals	4 (COOH)_2_	234–236	71
**3p**	Pale-yellow crystals	6 (COOH)_2_	222–224	72
**3q**	Pale-yellow crystals	6 (COOH)_2_	229–231	75
**3r**	Yellow-orange crystals	6 (COOH)_2_	238–240	57
**3s**	White crystals	4 (COOH)_2_	>260	91
**3t**	White crystals	6 (COOH)_2_	252–254	86
**3u**	White crystals	4 (COOH)_2_	186–188	80
**3v**	Beige crystals	4 (COOH)_2_	234–236	74

^a^The stoichiometry and composition of the salts were determined by elemental analyses and obtained values were within ±0.4% of the theoretical values.

^b^Crystallisation solvent: 2-PrOH–H_2_O.

^c^The yields only included the conversions into the ammonium oxalates.

### *In vitro* antimalarial activity

All the new quinoline, isoquinoline, and quinazoline derivatives **1–3** were evaluated for their antimalarial activity *in vitro* by incubation with *P. falciparum* CQ-resistant strain W2 (IC_50_ CQ = 0.40 µM, IC_50_ MQ = 0.016 µM) and the strain 3D7, which is CQ sensitive and which has decreased sensitivity to MQ (IC_50_ CQ = 0.11 µM, MQ = 0.06 µM). As shown in Table 2, these new derivatives **1–3** showed IC_50_ values between 0.13 and 11.50 µM against W2 and between 0.032 and 10.43 µM against 3D7 *P. falciparum* strains. These biological results could be analysed concerning the position on the phenyl ring of these polyamino side chains, the nature of the terminal amine, and also the influence of the length of the carbon chain between the two amino functions of the side-chain.

**Table 2.** *In vitro* sensitivity of *P. falciparum*, *L. donovani* and *T. brucei brucei* strains to compounds **1a–t**, **2a–l** and **3a–v** and cytotoxicity of these compounds in HepG2 cells.

Compound	*P. falciparum strains*IC_50_ values (μM)^a^		*L. donovani*IC_50_ values (μM)^b^	*Trypanosoma brucei brucei* IC_50_ values (μM )^c^	Cytotoxicity to HepG2 cells CC_50_ values (μM)^d^
	W2	3D7		*Trypanos Antat 1.9*	
Chloroquine^e^	0.40 ± 0.04	0.11 ± 0.01	n.d.^h^	n.d.^h^	30
Mefloquine^e^	0.016 ± 0.002	0.06 ± 0.003	n.d.^h^	n.d.^h^	n.d.^h^
Pentamidine^f^	n.d.^h^	n.d.^h^	5.5 ± 0.8	0.0002 ± 0.00006	2.3 ± 0.5
Amphotericin B^f^	n.d.^h^	n.d.^h^	0.1 ± 0.04	n.d.^h^	8.8 ± 0.6
Suramine^g^	n.d.^h^	n.d.^h^	n.d.^h^	0.03 ± 0.003	n.d.^h^
Fexinidazole^g^	n.d.^h^	n.d.^h^	n.d.^h^	0.59 ± 0.039	n.d.^h^
Eflornithine^g^	n.d.^h^	n.d.^h^	n.d.^h^	15.19 ± 0.64	n.d.^h^
Doxorubicin	n.d.^h^	n.d.^h^	n.d.^h^	n.d.^h^	0.06 ± 0.02
**1a**	n.d.^h^	4.08 ± 0.488	≥10^i^	0.84 ± 0.033	5.91 ± 1.5
**1b**	0.88 ± 0.14	0.47 ± 0.06	≥10^i^	0.82 ± 0.022	4.09 ± 0.2
**1c**	0.34 ± 0.037	0.032 ± 0.004	≥10^i^	1.48 ± 0.085	3.10 ± 0.9
**1d**	n.d.^h^	0.69 ± 0.070	≥10^i^	1.22 ± 0.121	7.71±.4
**1e**	1.29 ± 0.063	0.47 ± 0.06	≥10^i^	1.78 ± 0.089	11.33 ± 0.5
**1f**	0.13 ± 0.024	2.42 ± 0.27	≥10^i^	0.99 ± 0.132	4.09 ± 0.3
**1g**	n.d.^h^	0.099 ± 0.007	≥10^i^	0.38 ± 0.045	1.47 ± 0.3
**1h**	n.d.^h^	1.34 ± 0.173	≥10^i^	1.59 ± 0.071	4.50 ± 0.4
**1i**	7.92 ± 0.64	3.09 ± 0.38	≥10^i^	0.67 ± 0.091	3.58 ± 0.3
**1j**	4.21 ± 0.31	2.65 ± 0.18	≥10^i^	0.62 ± 0.070	2.50 ± 0.2
**1k**	4.10 ± 0.54	2.59 ± 0.20	≥10^i^	0.98 ± 0.118	6.16 ± 0.4
**1l**	4.51 ± 0.71	1.80 ± 0.20	≥10^i^	1.54 ± 0.164	1.02 ± 0.1
**1m**	>40	8.98 ± 0.36	≥10^i^	1.08 ± 0.068	5.40 ± 0.3
**1n**	1.69 ± 0.24	3.18 ± 0.36	≥10^i^	1.20 ± 0.296	0.48 ± 0.1
**1o**	2.26 ± 0.48	2.41 ± 0.08	≥10^i^	1.12 ± 0.118	3.32 ± 0.5
**1p**	2.73 ± 0.76	4.34 ± 0.50	≥10^i^	1.01 ± 0.135	6.44 ± 0.6
**1q**	n.d.^h^	1.44 ± 0.128	≥10^i^	1.30 ± 0.101	6.74 ± 0.7
**1r**	n.d.^h^	1.95 ± 0.774	≥10^i^	1.36 ± 0.194	4.97 ± 0.3
**1s**	n.d.^h^	0.21 ± 0.040	≥10^i^	0.73 ± 0.264	2.63 ± 0.5
**1t**	n.d.^h^	0.23 ± 0.021	≥10^i^	0.46 ± 0.120	3.93 ± 1.1
**2a**	6.23 ± 0.80	1.74 ± 0.36	≥10^i^	0.64 ± 0.163	8.31 ± 1.0
**2b**	3.02 ± 0.25	1.82 ± 0.31	≥10^i^	0.35 ± 0.049	3.47 ± 0.3
**2c**	4.42 ± 0.56	1.96 ± 0.58	≥10^i^	1.10 ± 0.318	4.98 ± 0.5
**2d**	5.46 ± 0.44	5.77 ± 0.67	≥10^i^	1.28 ± 0.122	14.85 ± 1.1
**2e**	6.06 ± 0.64	4.41 ± 0.51	≥10^i^	0.55 ± 0.122	9.57 ± 1.0
**2f**	4.76 ± 0.66	1.47 ± 0.24	≥10^i^	0.52 ± 0.072	2.12 ± 0.2
**2g**	7.61 ± 0.94	3.27 ± 0.31	≥10^i^	1.00 ± 0.261	10.16 ± 0.9
**2h**	1.28 ± 0.34	1.81 ± 0.19	≥10^i^	0.81 ± 0.255	7.18 ± 0.6
**2i**	2.40 ± 0.49	8.46 ± 0.66	≥10^i^	0.64 ± 0.323	10.76 ± 0.8
**2j**	5.09 ± 0.76	7.21 ± 0.59	≥10^i^	0.59 ± 0.131	2.16 ± 0.2
**2k**	5.96 ± 0.54	8.91 ± 0.87	≥10^i^	0.99 ± 0.122	6.48 ± 0.6
**2l**	9.60 ± 0.99	9.66 ± 1.20	≥10^i^	1.34 ± 0.088	2.01 ± 0.2
**3a**	n.d.^h^	2.15 ± 0.106	≥10^i^	0.90 ± 0.054	4.23 ± 0.4
**3b**	n.d.^h^	0.62 ± 0.077	≥10^i^	1.18 ± 0.057	4.01 ± 0.8
**3c**	n.d.^h^	1.06 ± 0.103	≥10^i^	1.48 ± 0.178	15.87 ± 1.7
**3d**	n.d.^h^	2.35 ± 0.488	≥10^i^	1.86 ± 0.135	28.72 ± 1.9
**3e**	n.d.^h^	1.52 ± 0.172	≥10^i^	2.39 ± 0.129	15.87 ± 1.7
**3f**	n.d.^h^	0.42 ± 0.083	≥10^i^	0.81 ± 0.063	4.52 ± 0.4
**3g**	n.d.^h^	0.15 ± 0.027	≥10^i^	1.27 ± 0.632	4.08 ± 0.6
**3h**	3.68 ± 0.50	3.84 ± 0.38	≥10^i^	0.27 ± 0.035	11.67 ± 1.2
**3i**	4.34 ± 0.74	4.05 ± 0.95	≥10^i^	0.44 ± 0.084	1.93 ± 0.1
**3j**	1.23 ± 0.15	0.56 ± 0.02	≥10^i^	1.04 ± 0.085	3.12 ± 0.2
**3k**	4.84 ± 0.58	3.12 ± 0.23	≥10^i^	0.94 ± 0.089	22.62 ± 1.1
**3l**	1.34 ± 0.29	1.81 ± 0.25	≥10^i^	0.59 ± 0.057	6.53 ± 0.6
**3m**	4.22 ± 0.55	1.18 ± 0.43	≥10^i^	0.75 ± 0.060	25.13 ± 1.2
**3n**	n.d.^h^	2.52 ± 0.237	≥10^i^	1.05 ± 0.058	2.71 ± 0.3
**3o**	0.99 ± 0.09	0.61 ± 0.03	≥10^i^	0.75 ± 0.121	1.14 ± 0.4
**3p**	5.08 ± 0.48	6.56 ± 1.44	≥10^i^	1.02 ± 0.194	17.35 ± 1.2
**3q**	11.50 ± 1.05	10.43 ± 0.58	≥10^i^	1.71 ± 0.145	1.27 ± 0.5
**3r**	4.75 ± 0.31	4.34 ± 0.33	≥10^i^	1.07 ± 0.164	3.90 ± 0.9
**3s**	n.d.^h^	1.13 ± 0.107	≥10^i^	2.07 ± 0.196	1.14 ± 0.4
**3t**	n.d.^h^	2.24 ± 0.435	≥10^i^	2.05 ± 0.535	29.57 ± 2.2
**3u**	n.d.^h^	0.43 ± 0.142	≥10^i^	0.72 ± 0.070	2.64 ± 0.2
**3v**	n.d.^h^	0.36 ± 0.126	≥10^i^	1.37 ± 0.658	6.46 ± 1.0

^a^Values were measured against chloroquine-resistant and mefloquine-sensitive strain W2 and the chloroquine-sensitive and mefloquine-resistant strain 3D7.

^b^IC50 values were measured against the promastigotes of Leishmania donovani strain. The IC50 (µM) values correspond to the mean ± standard deviations from three independent experiments.

^c^IC50 values were measured against the slender bloodstream trypomastigotes of *Trypanosoma brucei brucei* AnTat 1.9 strain. The IC50 (µM) values correspond to the means ± standard deviations from 3 independent experiments with each concentration tested in duplicate in all experiments.

^d^CC50 values were measured against HepG2 cells. The CC50 (µM) values correspond to the means ± standard deviations from three independent experiments.

^e^Chloroquine and mefloquine were used as antiplasmodial compounds of reference.

^f^Pentamidine and amphotericin B were used as antileishmanial compounds of reference.

^g^Suramine, pentamidine, fexinidazole and eflornithine were used as antitrypanosomal compounds of reference.

^h^n.d.: not done.

^i^No activity noted at the highest concentration tested.

Against the *P. falciparum* CQ-sensitive strain 3D7, compound **1c** bearing dimethylaminoethylamino side chains at position 4 of each of the benzyl moieties was found to be the most active derivative with an IC_50_ of 0.032 µM. In the non-substituted quinoline series, derivative **1c** bearing dimethylaminoethylamino side chains at position 4 of the benzyl rings displayed better activity than the analogues substituted with dimethylaminobutylamino or dimethylaminopropylamino side chains (IC_50_=0.0.32 µM for **1c** *versus* IC_50_=0.47 and 4.08 µM for **1b** and **1a**, respectively). In comparison, when we replaced the dimethylamino function by a methylpiperazine moiety (compounds **1d–f**), the quinoline **1f** bearing a C_2_ side chains showed a decrease in antimalarial activity; i.e. IC_50_=0.69 µM for **1d** and 0.47 µM for **1e** *versus* 2.42 µM for **1f**). In addition, the quinolines **1g–h** which are disubstituted with C_3_ or C_2_ morpholino chains on position 4 of its benzyl moieties, exhibited similar behaviour in the biological activity; i.e. morpholinopropylamino compound **1g** was found more active (up to 13.5 times) than its morpholinoethylamino analogue **1h** with IC_50_ of 0.099 *versus* 1.34 µM. The same observations could be done against the *P. falciparum* CQ-resistant strain W2 with the biological results obtained for these quinolines **1a–h**.

The introduction of a methoxy group substituted in position 6 or 7 of the quinoline ring (compounds **1i–p**) did not increase the antimalarial activity but seemed to decrease it when compared to the non-substituted quinoline (**1a–h** and **1q–t**). In these subseries in which the quinoline moiety was substituted by a methoxy, the biological activity was found more interesting with a substituted-aminopropylamino side chain (derivatives **1j**, **1l,** and **1n**) compared to a substituted-aminobutylamino side chain (derivatives **1i**, **1n**, and **1 m**), excepted for compounds **1o** *versus* **1p** (IC_50_=2.41 µM for **1o** *versus* IC_50_=4.34 µM for **1p**). Quinoline derivatives with polyamino side chains at position 3 of the benzyl moieties (compounds **1q–t**) were significantly less active than quinolines **1a**–**h** substituted in the position 4 of the benzyl rings, with the exception of compound **1t** which showed a better activity (IC_50_=0.23 µM) than its counterpart bearing the morpholinoethylamino side chains at position 4 (compound **1 h**) for which the IC_50_ was found to be 1.34 µM.

Against the 3D7 strain, the non-substituted isoquinolines **2a–d** were generally the most active compounds in these series, except for **2d** (IC_50_=5.77 µM) when compared to **2h** (IC_50_=1.81 µM), and also for **2b** (IC_50_=1.82 µM) *versus* **2f** (IC_50_=1.47 µM). Thus, introduction of a methoxy substituent on the isoquinoline heterocyclic skeleton was not beneficial, meanly in position 6 (compounds **2i–l**) for which the IC_50_ were found between 7.21 and 9.66 µM. Moreover, quinolines **1** generally exhibited better antimalarial activities against the *P. falciparum* 3D7 strain than their isoquinolines analogues **2**, with the exception of compound **2a** which showed better antimalarial activity than **1a** (IC_50_=4.08 µM for **1a** *versus* IC_50_=1.74 µM for **2a**). Against the *P. falciparum* CQ-resistant strain W2, compound **2h** bearing methylpiperazinylpropylamino side chains at position 4 of each of the benzyl moieties was found to be the most active with an IC_50_ of 1.28 µM.

Among the newly substituted quinazolines **3a–r**, all derivatives bearing aminoethylamino side chains at position 4 of the benzyl rings generally displayed better activities against 3D7 and W2 strains than their analogues substituted with aminopropylamino or aminobutylamino side chains (for example: IC_50_=0.56 µM for **3j** *versus* IC_50_=3.84 and 4.05 µM against 3D7 strain for **3h** and **3i**, respectively; IC_50_=1.23 µM for **3j** *versus* IC_50_=3.68 and 4.34 µM against W2 strain for **3h** and **3i**, respectively). However, the aminopropylamino substituted compound **3b** was found more active than its analogue aminoethylamino quinazoline **3c** against the 3D7 strain (IC_50_=0.62 µM for **3b** *versus* IC_50_=1.06 for **3c**). The substitution of the amino-alkylamino side chains at position 3 of the benzyl nucleus in these quinazoline series was not found detrimental in comparison with the substitution in position 4 against both of the *P. falciparum* strains.

### *In vitro* antileishmanial activity against promastigote forms

In order to better understand the biological profile of our new heterocycles **1–3**, some complementary antiparasitic analyses were also performed. Notably, *P. falciparum* belongs to the coccidian protozoan parasite family. Therefore, *in vitro* activity against flagellate protozoan parasite *L. donovani* was evaluated (Table 2). The reference drugs amphotericin B and pentamidine had IC_50_ values of 0.10 and 5.50 µM, respectively, against *L. donovani*. Unfortunately, none of our novel compounds showed any antileishmanial activity *in vitro* (all IC_50_ values ≥ 10 µM).

### *In vitro* activity against Trypanosoma brucei brucei

These newly synthesised nitrogen heterocyclic derivatives **1–3** were evaluated against *T. brucei brucei*. Pentamidine, suramine, fexinidazole, and eflornithine were also used here as reference compounds. The screening data are presented in Table 2. All quinolines, isoquinolines, and quinazolines **1–3** were active against *T. brucei brucei* with IC_50_ values ranging from 0.27 to 2.39 µM; and most of them showed an antiparasitic activity around the µM value. Among them the quinazoline **3e** was found as the less active compound (IC_50 _=2.39 µM), while the best activity against the *T. brucei brucei* strain was observed with 6-methoxy substituted quinazoline **3h** with an IC_50_ of 0.27 µM. Surprisingly, as all these new tested nitrogen heterocycles **1–3** showed similar range of antiparasitic activities against *T. brucei brucei*, no relevant structure-activity relationships (SAR) could be deduced among these new derivatives. However, the quinoline **1g** which is disubstituted with C_3_ morpholinopropyl chains on position 4 of its benzyl moieties, exhibited better antitrypanosomal activity than its C_2_ analogue (compound **1h**), and was found four-time more active with IC_50_ of 0.38 *versus* 1.59 µM. In addition, the structural analogous **1a** and **1b**, bearing, respectively (dimethylaminobutyl)aminomethyl and (dimethylaminopropyl)aminomethyl side chains at position 4 of their phenyls, were found slightly more active than the (dimethylaminoethyl)aminomethyl compound **1c** (IC_50 _=0.84, 0.82, and 1.48 µM for **1a**, **1b,** and **1c**, respectively). In comparison, when we replaced the dimethylamino function by a methylpiperazine moiety (compounds **1d–f**), quinoline **1f** bearing a C_2_ side chains at position 4 of the phenyls showed an increase in the antitrypanosomal activity; i.e. IC_50_=1.22 µM for **1d** and 1.78 µM for **1e** *versus* 0.99 µM for **1f**). In the isoquinoline series (compounds **2**), derivatives **2a** and **2b** (IC_50_ of 0.64 and 0.35 µM) disubstituted with a C_4_ or C_3_ dimethylaminoalkyl chains on position 4 of the benzyl moieties, exhibited better antitrypanosomal activity than their methylpiperazinealkyl C_4_ or C_3_ analogues (compounds **2c** and **2d**) for which IC_50_ were noticed at 1.10 and 1.28 µM, respectively. Interestingly, a similar behaviour was also observed with their 6- and 7-methoxy substituted isoquinoline analogous (compounds **2e–h** and **2i–l**, respectively) for which the substitution of the dimethylamino terminal amine on the polyaminoalkyl side chains by a methylpiperazine moiety led to a decrease in the antitrypanosomal activity (IC_50_=0.52–0.55 µM for **2e–f** *versus* 0.81–1.00 µM for **2g–h**, and IC_50_=0.59–0.64 µM for **2i–j** *versus* 0.99–1.34 µM for **2k–l**). The influence of the length of the carbon chain in the polyaminoalkyl side-chain for the 6-methoxy substituted quinazolines **3h–j** seems also to be detrimental: a shorter alkyl chain (C_4_ to C_2_) led to a decreased antitrypanosomal activity (IC_50 _=0.27 µM for **3h** *versus* 1.04 and 0.44 µM for **3j** and **3i**, respectively).

### Cytotoxicity and selectivity index

In order to assess selectivity of action, the cytotoxicities of these new synthesised antiparasitic heterocyclic compounds **1–3** were evaluated *in vitro* in the human cell line HepG2, which is a commonly used human-derived hepatocarcinoma cell line. These cells express many hepatocyte-specific metabolic enzymes. The aim of this assay using HepG2 cells was to evaluate the impact of metabolic activation of the tested compounds on cell viability. The cytotoxic concentrations 50% (CC_50_) were determined, and selectivity indexes (SIs), defined as the ratios of cytotoxic to antiparasitic activities (SI = CC_50_/IC_50_) were calculated. The results of cytotoxicity assays and the associated SI values are presented in Table 3. Most of these “quinoline-like” derivatives that were found active against the different parasites showed significant cytotoxicity against the HepG2 cells with CC_50_ values ranging from 0.48 to 29.57 µM. Concerning the W2 strain, the calculated SIs were between 0.11 and 31.46. For the CQ sensitive strain 3D7, the SIs were noticed from 0.12 to 96.88. Analyses of SI values led us to identify the quinoline compound **1c** as a promising compound with a SI of 96.88 for the 3D7 strain. In addition, the quinazoline **3g** also had interesting SI value of 27.20 for the CQ sensitive strain 3D7. Against the *T. brucei brucei* strain, quinazolines **3h** and **3m** had SIs of 43.22 and 33.51, respectively. These SI values could indicate that these new nitrogen heterocyclic derivatives warrant further investigation into their potential use as antiparasitic drugs.

**Table 3.** Selectivity indexes of compounds **1–3**.

	Selectivity index^a^		
Compound	HepG2/W2	HepG2/3D7	HepG2/*Tryp.*
Chloroquine	75	272	n.d.^b^
Pentamidine	n.d.^b^	n.d.^b^	11500
**1a**	n.d.^b^	1.45	7.03
**1b**	0.21	8.70	5.00
**1c**	9.11	96.88	2.09
**1d**	n.d.^b^	11.17	6.32
**1e**	8.78	24.10	6.36
**1f**	31.46	1.69	4.13
**1g**	n.d.^b^	14.85	3.87
**1h**	n.d.^b^	3.36	2.83
**1i**	0.45	1.16	5.34
**1j**	0.59	0.94	4.03
**1k**	1.50	2.38	6.28
**1l**	0.23	0.57	0.66
**1m**	0.13	0.60	5.00
**1n**	0.28	0.15	0.40
**1o**	1.47	1.38	2.96
**1p**	2.36	1.48	6.37
**1q**	n.d.^b^	4.68	5.18
**1r**	n.d.^b^	2.55	3.65
**1s**	n.d.^b^	12.52	3.60
**1t**	n.d.^b^	17.09	8.54
**2a**	1.33	4.78	12.98
**2b**	1.15	1.90	9.91
**2c**	1.13	2.54	4.53
**2d**	2.72	2.57	11.60
**2e**	1.58	2.17	17.4
**2f**	0.45	1.44	4.07
**2g**	1.33	3.11	10.16
**2h**	5.61	3.97	8.86
**2i**	4.48	1.27	16.81
**2j**	0.42	0.30	3.66
**2k**	1.09	0.73	6.54
**2l**	0.21	0.21	1.50
**3a**	n.d.^b^	1.96	4.70
**3b**	n.d.^b^	6.47	3.40
**3c**	n.d.^b^	14.97	10.72
**3d**	n.d.^b^	12.22	15.44
**3e**	n.d.^b^	10.44	6.64
**3f**	n.d.^b^	10.76	5.58
**3g**	n.d.^b^	27.20	3.21
**3h**	3.17	3.04	43.22
**3i**	0.44	0.48	7.15
**3j**	2.54	0.77	7.09
**3k**	4.67	7.25	24.06
**3l**	4.87	3.61	11.06
**3m**	5.95	21.30	33.51
**3n**	n.d.^b^	1.08	2.58
**3o**	1.15	1.87	1.52
**3p**	3.41	2.64	17.01
**3q**	0.11	0.12	0.74
**3r**	0.82	0.90	3.64
**3s**	n.d.^b^	1.01	0.55
**3t**	n.d.^b^	13.20	14.42
**3u**	n.d.^b^	6.14	3.67
**3v**	n.d.^b^	17.94	4.72

^a^SI was defined as the ratio between the CC_50_ value on the HepG2 cells and the IC_50_ value against the *P. falciparum* W2 or 3D7 or *Trypanosoma brucei brucei* strains.

^b^ n.d.: not done.

### FRET-melting experiments

As the telomeres of the parasites *P. falciparum* and *Trypanosoma* could be potential targets of this kind of nitrogen heterocyclic compounds, we have also investigated stabilisation of the *P. falciparum* telomeric or *T. brucei brucei* chromosomic G-quadruplexes by our best bioactive compounds **1–3** through a FRET melting assays. We used a FRET melting assay to determine the degree to which the new quinoline, isoquinoline and quinazoline derivatives stabilise the G-quadruplexes formed by oligonucleotides with *P. falciparum* or *T. brucei brucei* as well as human telomeric sequences. For this purpose, we used two fluorescently labelled *P. falciparum* telomeric and one *T. brucei brucei* chromosomic sequences (FPf1T, FPf8T, and FtrypBT) and one human telomeric sequence (F21T).

To probe the G4 selectivity of our selected ligands **1–3** over duplex DNA, a FRET melting assay was performed using a duplex control sequence, FdxT. For comparison, we evaluated reference G4 ligand PhenDC3 and the antimalarial reference drugs CQ and MQ. To enable comparison of selectivities, we calculated the difference (ΔT_m_) between the T_m_ of the G-quadruplex formed by FPf1T, FPf8T, FtrypBT (FEBR1T), F21T, or FdxT in the presence or absence of each selected compound. These ΔT_m_ values are presented in Table 4. For these selected compounds, the ΔT_m_ values ranged from 0.1 to 27.3 °C at 2 µM ligand concentration. The best ligands which stabilise all the four G-quadruplexes sequences were compounds **2e–f**, **3h,** and **3m** (Table 4). 7-Methoxy-1,3-bis{4-[(4-dimethylaminobutyl)aminomethyl]phenyl}isoquinoline **2e** strongly stabilised all the four G-quadruplex sequences with ΔT_m_ values ranged from 22.2 to 27.3 °C. These nitrogen heterocyclic compounds **2e–f**, **3h,** and **3m** which exhibited a strong stabilisation profile were all substituted by a methoxy function on the heterocyclic moiety and dimethylaminobutylamino or dimethylaminopropylamino side chains at position 4 of the benzyl rings. Among the tested compounds, derivatives **2e–f** and **3h** displayed a better stabilisation profile for both the *P. falciparum* telomeric sequences than the human telomeric G-quadruplex. Moreover, it could be noticed that all the selected quinoline derivatives **1** scarcely stabilised the G-quadruplex, whereas all the isoquinolines **2** strongly stabilised the protozoal and human G-quadruplex. In addition, the quinazoline ligands **3** showed low to moderate ranges of stabilisation on the different telomeric G-quadruplex. These last results could also confirm the importance of the position of the nitrogen atom in the isoquinoline and quinazoline heterocycles. Concerning data noticed for the stabilisation of the *T. brucei brucei* non-telomeric G-quadruplexes (FtrypBT), the results showed the same profile as those obtained for the *P. falciparum* telomeric sequences (FPf1T, FPf8T) with slightly lower ΔT_m_ values. FRET assays showed there was no selectivity to duplex DNA sequence.

**Table 4.** FRET-melting values for the selected compounds **1–3**with FPf1T, FPf8T, FtryBT, F21T, and FdxT in K^+^ conditions at 2 μM.

	ΔT_m_ (°C)^a^	ΔT_m_ (°C)^a^	ΔT_m_ (°C)^a^	ΔT_m_ (°C)^a^	ΔT_m_ (°C)^a^
Compound	FPf1T	FPf8T	FtrypBT	F21T	FdxT
PhenDC3	24.6 ± 0.1	24.7 ± 0.2	19.2 ± 0.2	26.3 ± 0.1	0.1 ± 0.2
**CQ**	1.9 ± 0.1	2.4 ± 1.2	n.d.^b^	2.4 ± 1.1	n.d.^b^
**MF**	3.1 ± 0.5	6.6 ± 2.3	n.d.^b^	2.6 ± 0.5	n.d.^b^
**1b**	8.4 ± 1.0	9.9 ± 0.1	7.8 ± 1.2	12.7 ± 0.6	0.0 ± 0.7
**1c**	2.3 ± 0.7	2.7 ± 0.2	2.6 ± 0.4	2.8 ± 1.3	−0.5 ± 0.4
**1e**	9.4 ± 0.6	9.2 ± 0.5	7.4 ± 0.1	11.3 ± 1.1	0.1 ± 0.1
**1f**	10.7 ± 1.0	11.0 ± 0.5	9.4 ± 0.6	9.5 ± 2.9	−0.1 ± 0.2
**1g**	3.7 ± 0.3	3.7 ± 0.6	2.0 ± 0.2	7.1 ± 2.7	−1.8 ± 0.4
**1s**	3.0 ± 0.4	2.8 ± 0.5	1.8 ± 0.7	2.5 ± 0.1	−1.5 ± 0.4
**1t**	0.9 ± 0.2	0.6 ± 0.7	0.6 ± 0.4	0.3 ± 0.3	−1.6 ± 0.4
**2b**	13.6 ± 1.3	13.0 ± 1.1	11.7 ± 0.6	12.7 ± 0.3	0.3 ± 0.2
**2e**	27.3 ± 1.1	26.6 ± 0.2	24.0 ± 0.3	22.2 ± 0.1	4.1 ± 0.3
**2f**	20.3 ± 1.1	18.5 ± 0.5	16.9 ± 1.1	15.8 ± 0.1	0.8 ± 0.3
**2i**	12.8 ± 1.3	12.9 ± 1.0	8.4 ± 0.3	23.2 ± 0.6	0.9 ± 0.1
**2j**	15.3 ± 0.7	15.0 ± 0.6	12.2 ± 1.1	13.9 ± 0.1	0.5 ± 0.3
**3b**	11.6 ± 1.1	11.2 ± 0.8	10.0 ± 0.4	13.2 ± 0.3	0.1 ± 0.1
**3f**	3.3 ± 0.3	2.9 ± 0.3	2.8 ± 0.1	3.1 ± 0.5	−0.2 ± 0.2
**3g**	1.4 ± 0.1	1.2 ± 0.4	1.0 ± 0.5	4.2 ± 2.1	0.3 ± 0.2
**3h**	19.8 ± 0.6	18.8 ± 0.7	15.7 ± 0.3	16.4 ± 1.3	2.0 ± 0.2
**3i**	16.0 ± 0.3	15.6 ± 0.3	12.7 ± 0.7	12.6 ± 0.0	0.3 ± 0.3
**3j**	3.4 ± 0.4	3.1 ± 0.4	4.4 ± 0.5	7.2 ± 0.4	−0.4 ± 0.2
**3m**	18.3 ± 0.7	17.8 ± 0.5	13.8 ± 0.5	19.4 ± 0.4	1.7 ± 0.3
**3v**	0.5 ± 0.3	0.5 ± 0.4	0.6 ± 0.1	0.1 ± 0.1	0.0 ± 0.0

^a^ΔT_m_ of FPf1T, FPf8T, FtryBT, F21T and FdxT (0.2 μM) were recorded in 10 mM lithium cacodylate (pH 7.2), 10 mM KCl, 90 mM LiCl. PhenDC3 was tested at 0.5 μM, whereas CQ and MF at 1 μM. Error margins correspond to SD of three replicates.

^b^n.d.: not determined.

### Classification of bioactive ligands

The selected compounds **1–3** were classified through HAC in two clusters (Figure 2(A)), defining consistent compound types in relation to *P. falciparum* variables (FPf1T, FPf8T, F21T, and IC_50_ against the 3D7 strain). Both clusters included ten compounds and were clearly separated. The red cluster was distinguished by an increase in F21T, FPf1T, FPf8T, and 3D7 values, defining a compound class mainly characterised by a high affinity for *P. falciparum* and human telomeric G-quadruplexes and with a scarce antimalarial activity. On the other hand, the blue cluster was characterised by a decrease in FRET-melting and IC_50_ values, mainly defining a compound class with lower affinity for the employed G-quadruplex sequences and higher antimalarial activity. Consequently, these results suggest that the ability of our compounds to target *P. falciparum* telomeric G-quadruplex sequences is not a desirable property for antimalarial activity.

These selected bioactive compounds were classified in two consistent clusters also in relation to *T. brucei brucei* variables (FtryBT, F21T, and IC_50_ against *T. brucei brucei* strain) (Figure 2(B)). In sharp contrast with *P. falciparum* (Figure 2(A)), the red cluster was characterised by the most active molecules showing also a higher selectivity for the tested G-quadruplex sequences contrarily to the blue ones. The latter classification highlights a positive correlation between the anti-trypanosomal activity and the ability of these compounds to stabilise telomeric G-quadruplexes.

## Conclusions

In this report, we described the design, the synthesis, the antiprotozoal activities, and the *in vitro* cytotoxicity towards human cells of a novel series of 2,4-bis[(substituted-aminomethyl)phenyl]quinoline, 1,3-bis[(substituted-aminomethyl)phenyl]isoquinoline and 2,4-bis[(substituted-aminomethyl)phenyl]quinazoline derivatives. These new “quinoline-like” derivatives were tested for their *in vitro* antiparasitic activity towards the CQ-sensitive 3D7 and CQ-resistant W2 *P. falciparum* strains, the promastigote form of *L. donovani*, and a *T. brucei brucei* strain. Among these new synthesised nitrogen heterocyclic molecules, a few of them were identified as potential *in vitro* antiplasmodial leads with IC_50_ ranging from 0.032 to 0.23 µM on the W2 and 3D7 strains of *P. falciparum*. The 2,4-bis[(substituted-aminomethyl)phenyl]quinoline **1c** was identified as the most potent antimalarial candidate with a ratio of cytotoxic to antiparasitic activities of 97 against the *P. falciparum* CQ-sensitive strain 3D7. In general, the quinoline and quinazoline derivatives **1** and **3** were found more active against both *Plasmodium* strains than their isoquinoline analogues **2**. Moreover, introduction of a methoxy substituent on the heterocyclic moieties generally did not led to an increase of the antimalarial activity. Unfortunately, none of our compounds showed activity against the promastigote forms of *L. donovani*. Moreover, the antiprotozoal activity spectrum of our new synthesised derivatives using a *T. brucei brucei* strain revealed IC_50_ values ranging from 0.27 to 2.39 µM, which warrant further investigations. The 2,4-bis[(substituted-aminomethyl)phenyl]quinazoline **3 h** was also identified as the most potent trypanosomal candidate with SI of 43 on *Trypanosoma brucei brucei* strain. In addition, the *in vitro* cytotoxicity of these new heterocyclic compounds was assessed on the human HepG2 cell line. Structure-activity relationships of these new synthetic compounds are here also discussed, as well as their relative ability of targeting *P. falciparum* or *Trypanosoma* telomeres as an hypothetical mechanism of action. Thus, as the telomeres of the parasites could constitute interesting targets, we have also investigated the possibility of targeting *Plasmodium* telomeres or *Trypanosoma* chromosomes by stabilising the *Plasmodium* or *Trypanosoma* G-quadruplexes sequences through FRET melting assays with our best bioactive compounds. Concerning the stabilisation of the parasitic G-quadruplex, the isoquinoline derivatives **2** seem to better stabilise the protozoal and human G-4 structures in comparison with their quinoline and quinazoline homologues **1** and **3**.

## Supplementary Material

Supplemental Material
