# Supplementary material for: Design, synthesis, and antiprotozoal evaluation of new 2,4-bis[(substituted-aminomethyl)phenyl]quinoline, 1,3-bis[(substituted-aminomethyl)phenyl]isoquinoline and 2,4-bis[(substituted-aminomethyl)phenyl]quinazoline derivatives
Source: J Enzyme Inhib Med Chem. 2020 Jan 3;35(1):432–59. doi: 10.1080/14756366.2019.1706502 (PMC6968685; doi:10.1080/14756366.2019.1706502)
Supplement: Supplemental Material [file IENZ_A_1706502_SM3794.pdf]

# Design, synthesis, and antiprotozoal evaluation of new 2,4-bis[(substituted-aminomethyl)phenyl]quinoline, 1,3-bis[(substituted-aminomethyl)phenyl]isoquinoline and 2,4-bis[(substituted-aminomethyl)phenyl]quinazoline derivatives

Jean Guillon[1], Anita Cohen[2], Clotilde Boudot[3], Alessandra Valle[1], Vittoria Milano[1], Rabindra Nath Das[1], Aurore Guédin[1], Stéphane Moreau[1], Luisa Ronga[4], Solène Savrimoutou[1], Maxime Demourgues[1], Elodie Reviriego[1], Sandra Rubio[1], Sandie Ferriez[1], Patrice Agnamey[5], Cécile Pauc[5], Serge Moukha[6], Pascale Dozolme[6], Sophie Da Nascimento[5], Pierre Laumaillé[5], Anne Bouchut[5], Nadine Azas[2], Jean-Louis Mergny[1,7,8], Catherine Mullié[5], Pascal Sonnet[5], Bertrand Courtioux[3]

[1]Université de Bordeaux, INSERM U1212, UMR CNRS 5320, ARNA Laboratory, UFR des Sciences Pharmaceutiques, Bordeaux, France ; [2]Aix-Marseille Univ., IRD, AP-HM, SSA, VITROME, Marseille, France ; [3]INSERM U1094, Tropical Neuroepidemiology, Université de Limoges, Institute of Neuroepidemiology and Tropical Neurology, Limoges, France ; [4]Université de Pau, PREM UMR5254 - UPPA/CNRS, Technopole Hélioparc, Pau, France ; [5]Université de Picardie Jules Verne, UFR de Pharmacie, AGIR (Agents Infectieux, Résistance et chimiothérapie), EA 4294, Amiens, France ; [6]Université de Bordeaux, Laboratoire de Toxicologie et d'Hygiène Appliquée - INRA, UFR des Sciences Pharmaceutiques, Bordeaux, France ; [7]Institut Curie, Université Paris-Saclay, CNRS-UMR 9187, INSERM U1196, F-91405 Orsay, France ; [8]Institute of Biophysics of the CAS, v.v.i., Královopolská 135, 612 65 Brno, Czech Republic

**CONTACT** Jean Guillon, jean.guillon@u-bordeaux.fr, Université de Bordeaux, INSERM U1212, UMR CNRS 5320, ARNA Laboratory, UFR des Sciences Pharmaceutiques, 146, rue Léo Saignat, 33076 Bordeaux, France

### $^{1}$H NMR and $^{13}$C NMR spectra of 2,4-bis[(substituted-aminomethyl)phenyl]quinoline, 1,3-bis[(substituted-aminomethyl)phenyl]isoquinoline and 2,4-bis[(substituted-aminomethyl)phenyl]quinazoline derivatives 1-3 and some representative compounds 4-6.

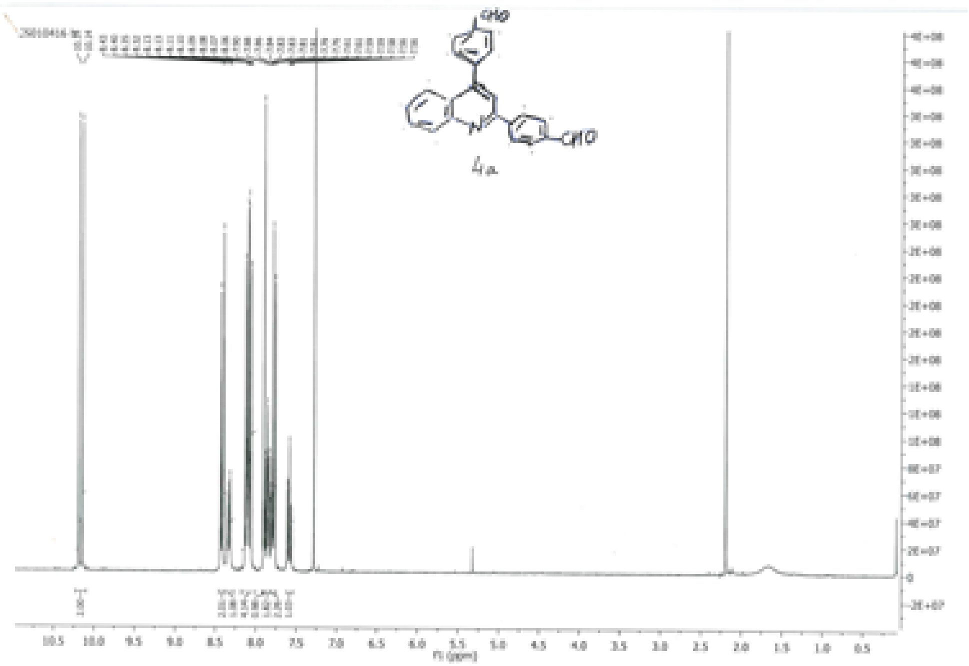


**Fig. S1.** $^{1}$H NMR spectrum of **4a**

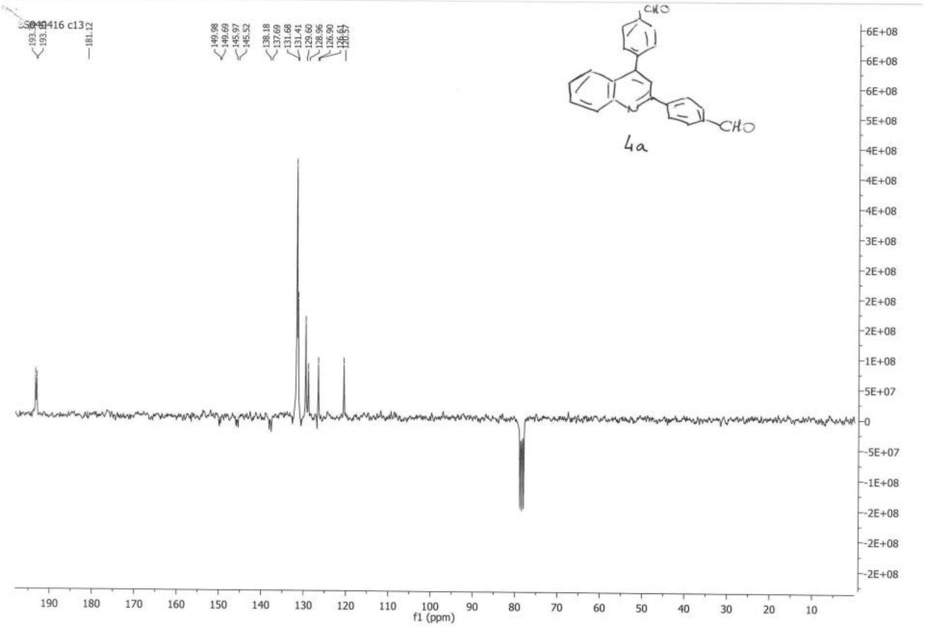


**Fig. S2.** $^{13}$C NMR spectrum of **4a**

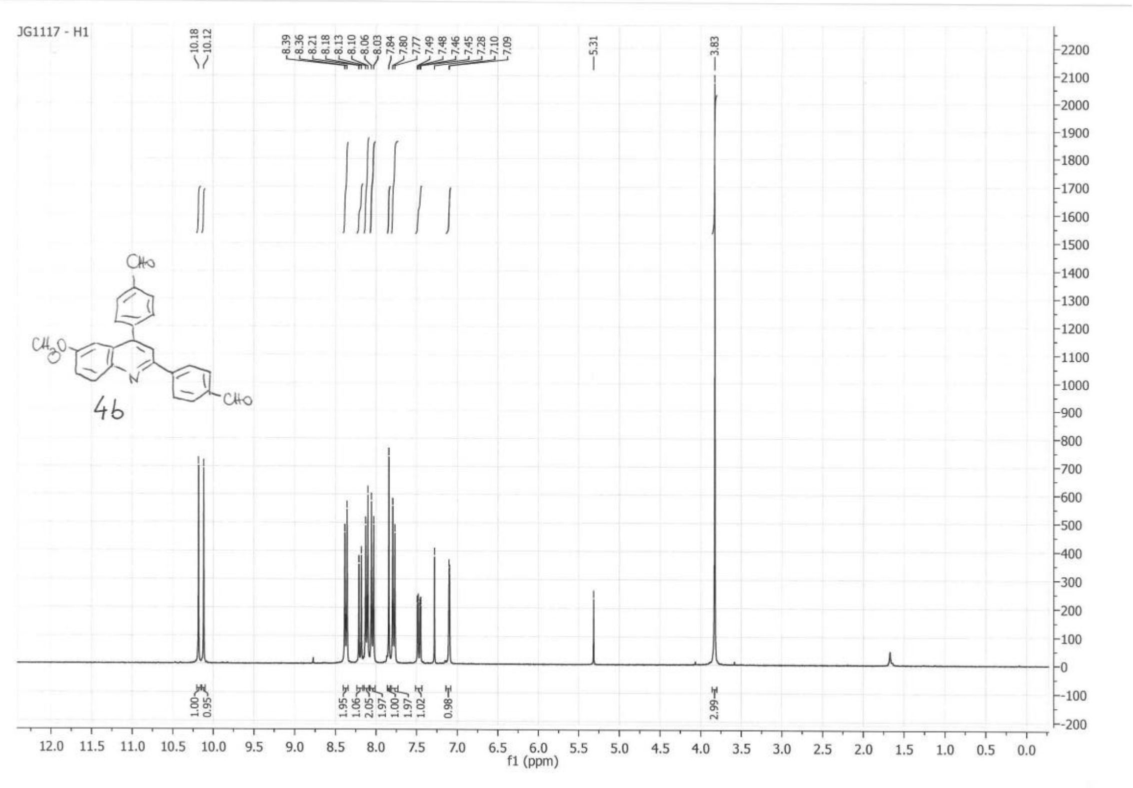

**Fig. S3.** $^{1}$H NMR spectrum of **4b**

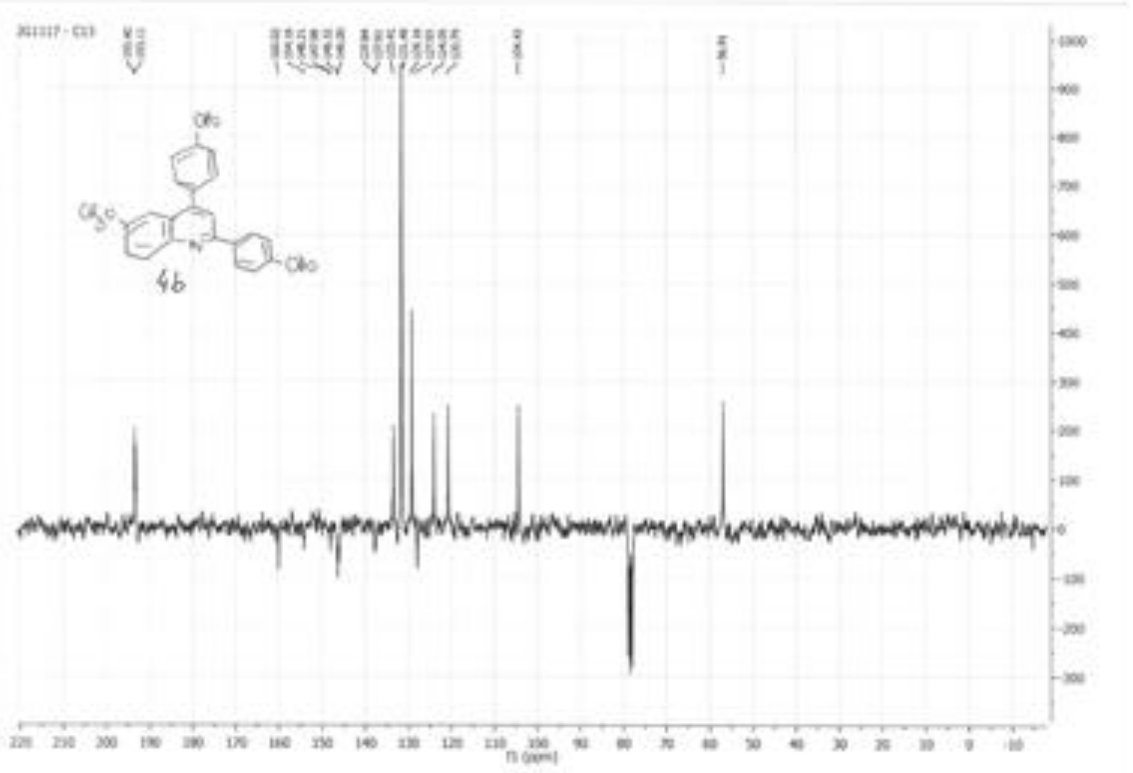

**Fig. S4.** $^{13}$C NMR spectrum of **4b**

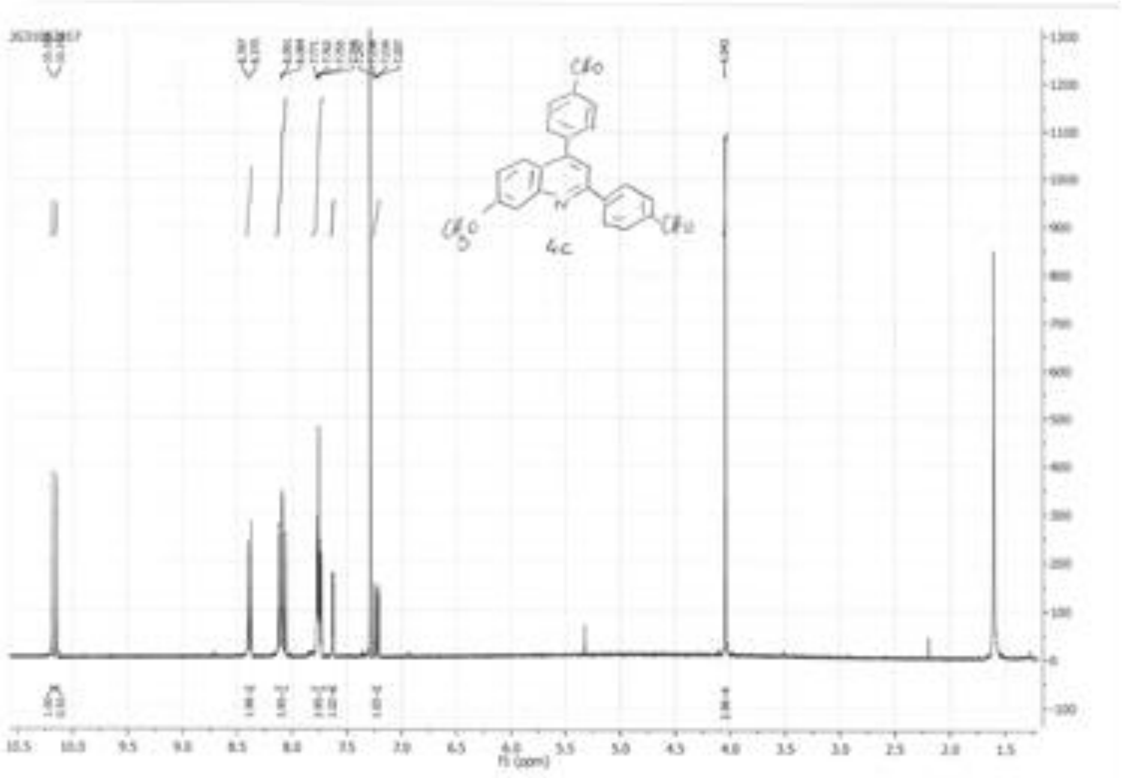

**Fig. S5.** $^{1}$H NMR spectrum of **4c**

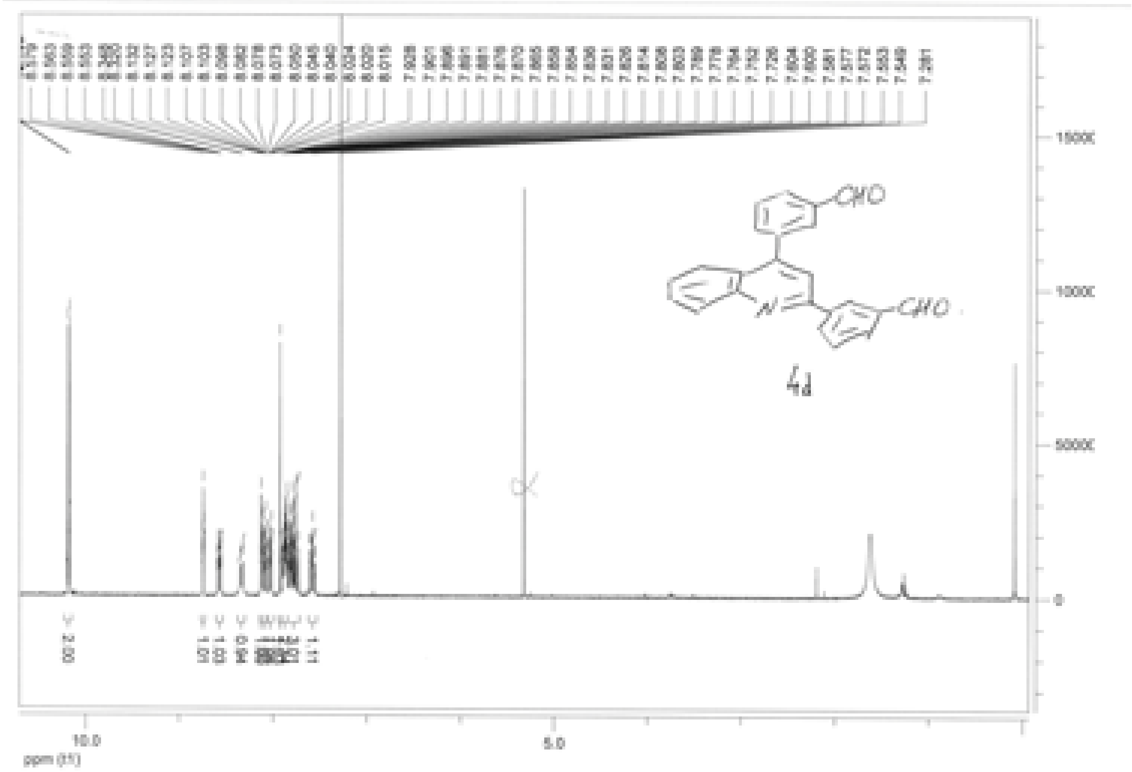

**Fig. S6.** $^1$H NMR spectrum of **4d**

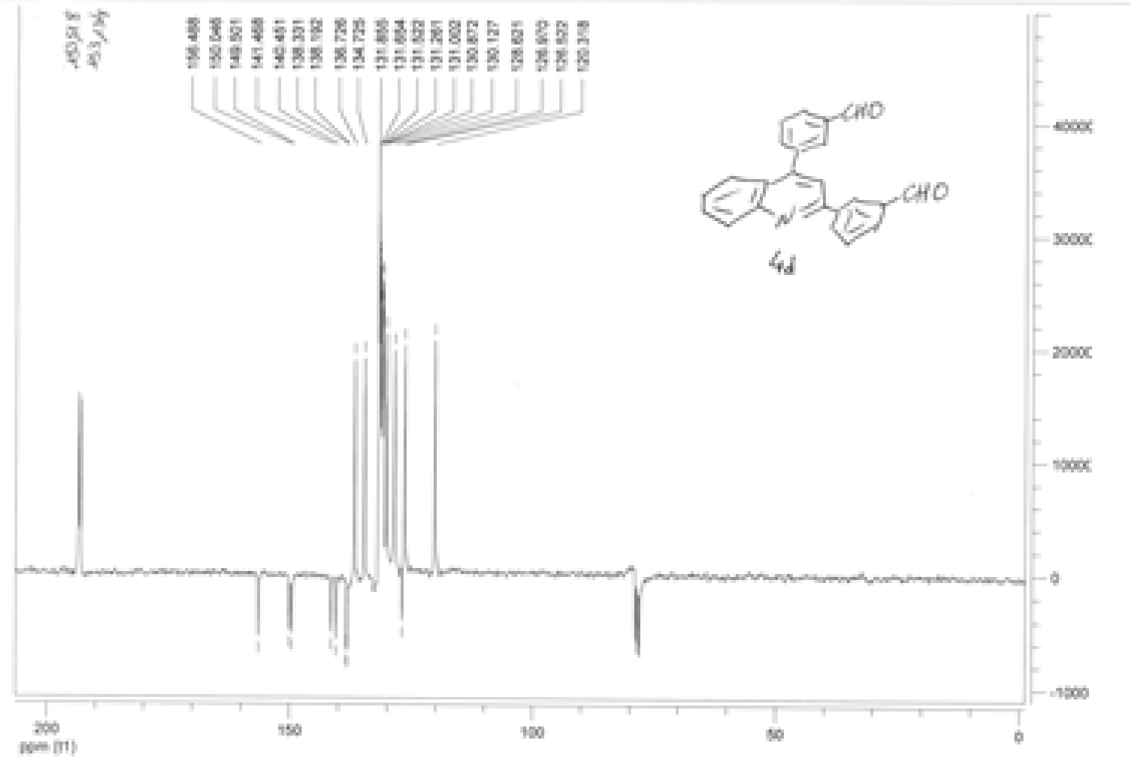

**Fig. S7.** $^{13}$C NMR spectrum of **4d**

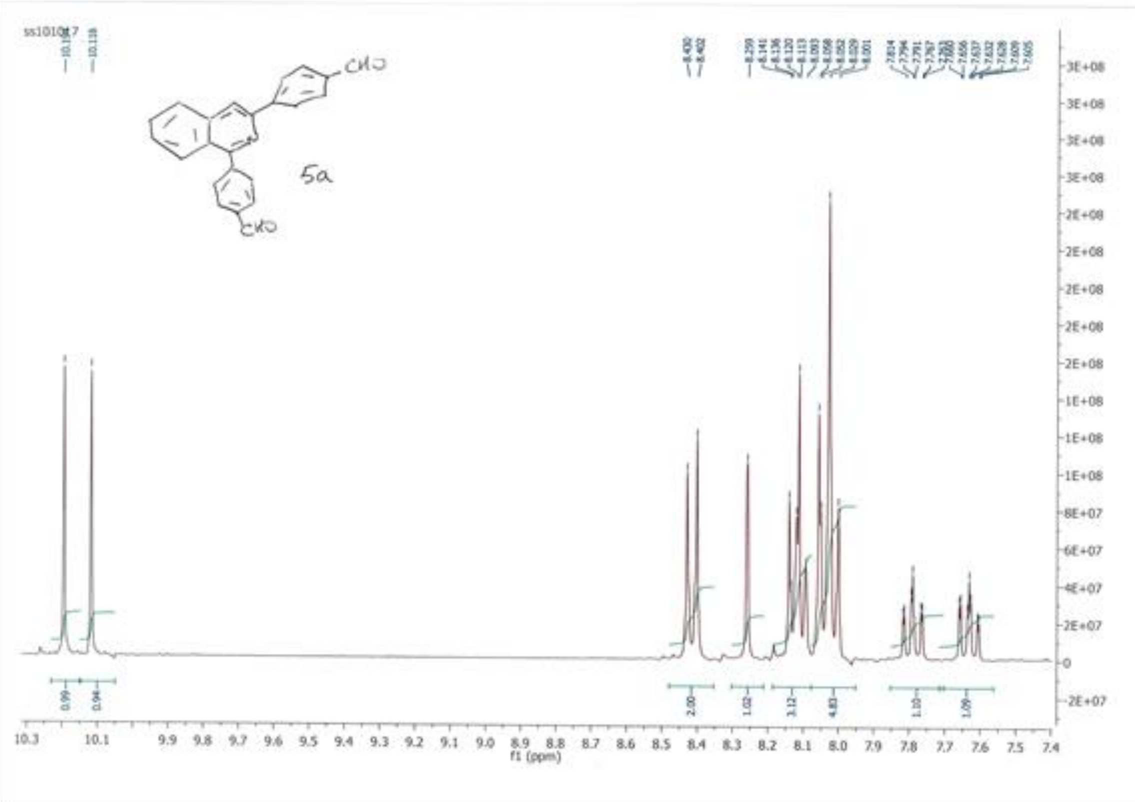

**Fig. S8**. $^{1}$H NMR spectrum of **5a**

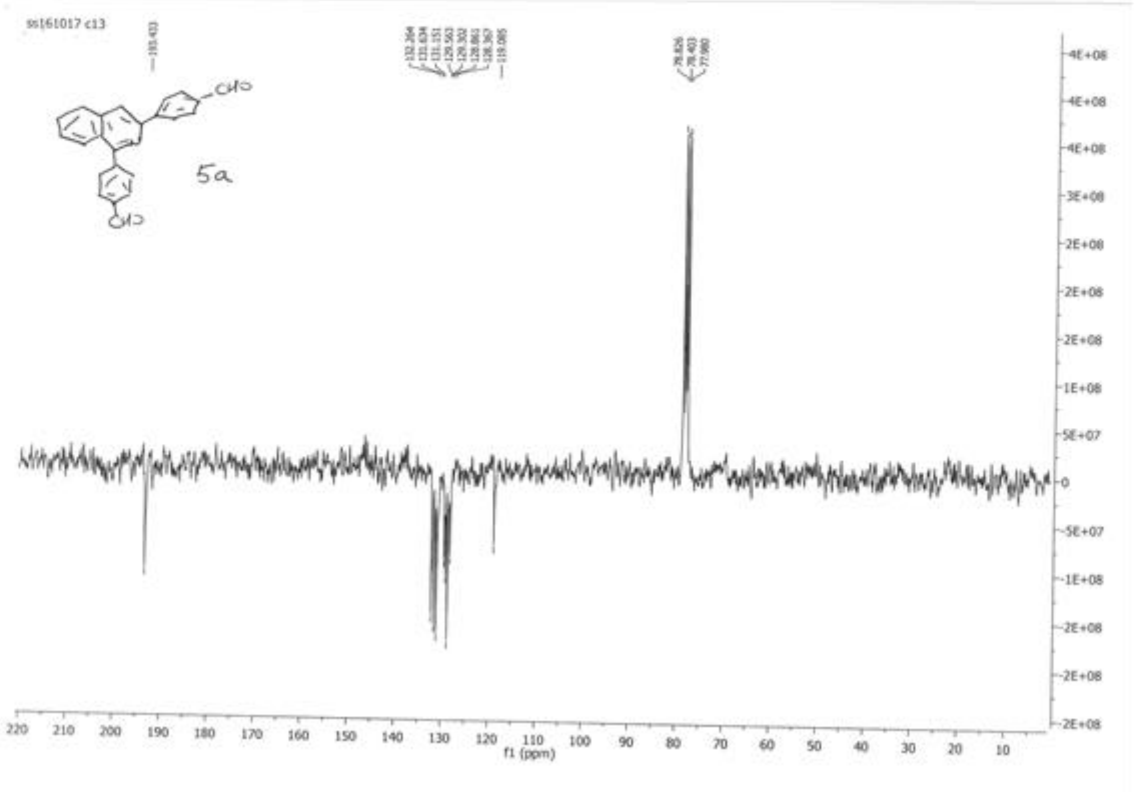

**Fig. S9.** $^{13}$C NMR spectrum of **5a**

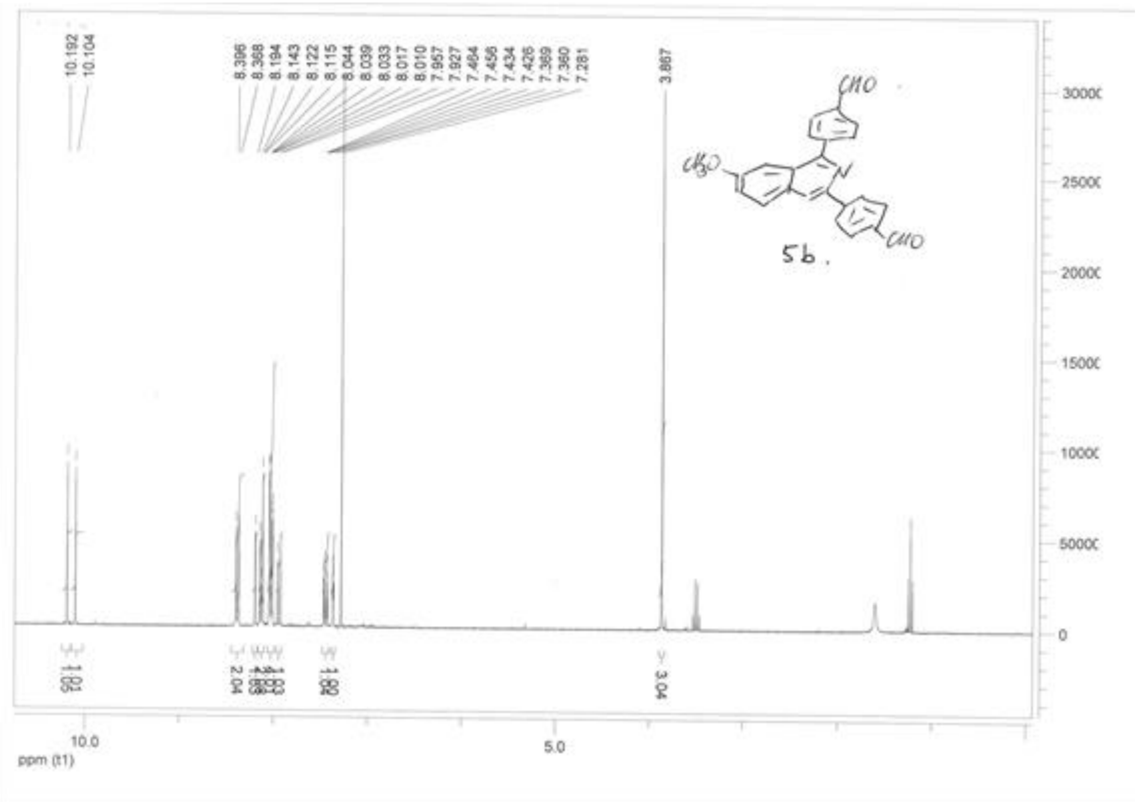

**Fig. S10.** $^{1}$H NMR spectrum of **5b**

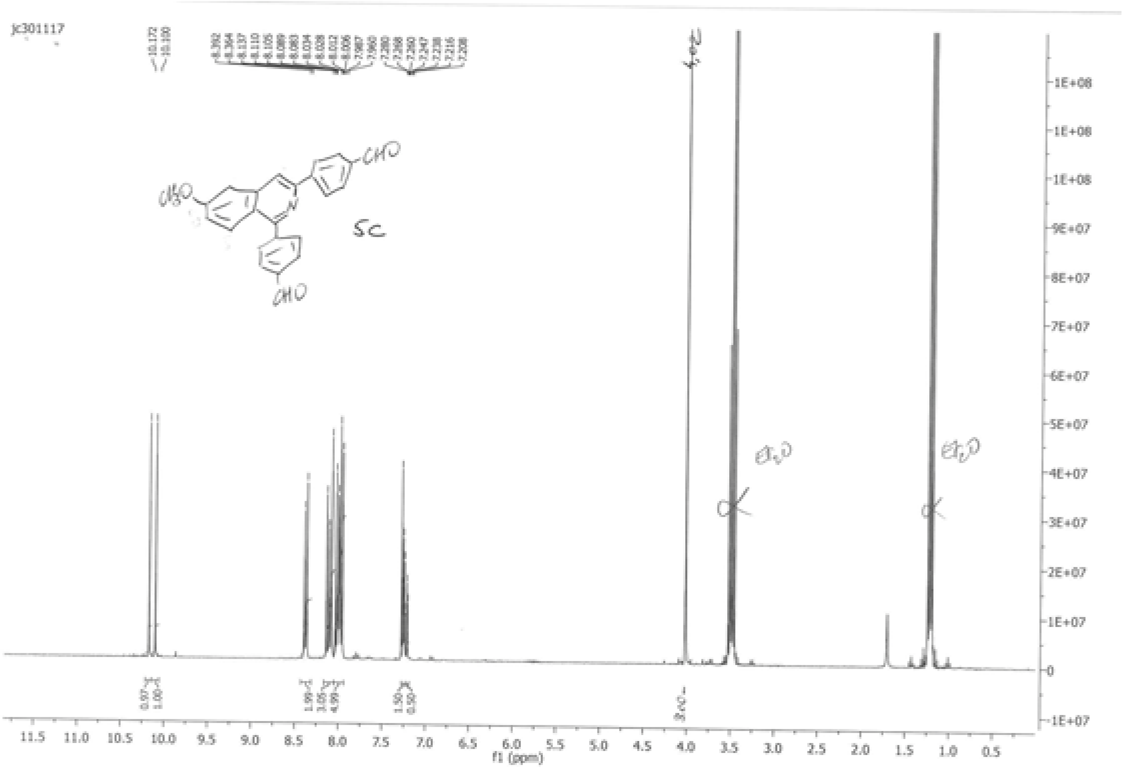

**Fig. S11.** $^{1}$H NMR spectrum of **5c**

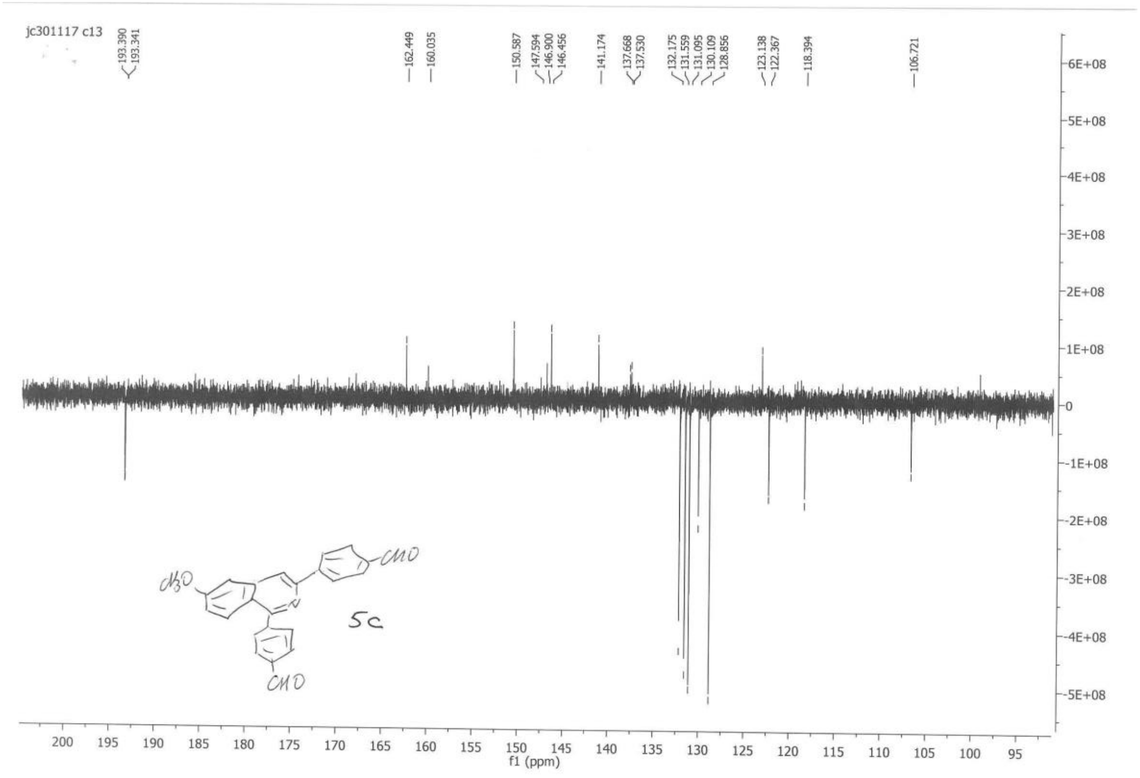

**Fig. S12.** $^{13}$C NMR spectrum of **5c**

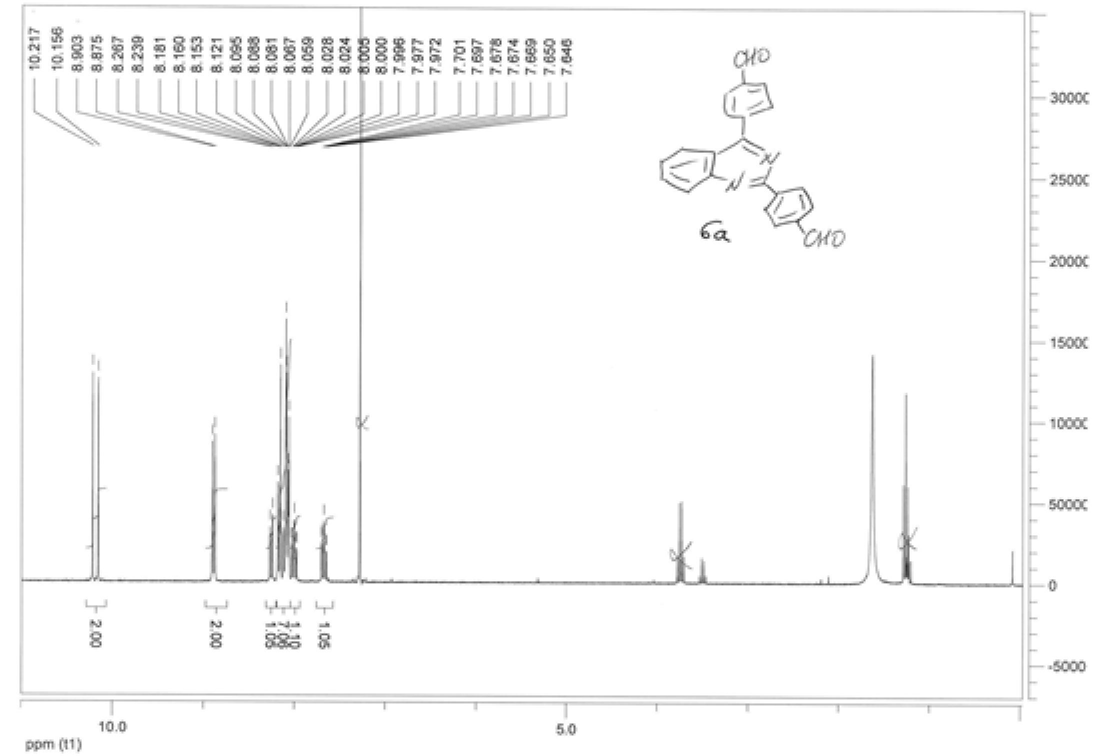

**Fig. S13.** $^{1}$H NMR spectrum of **6a**

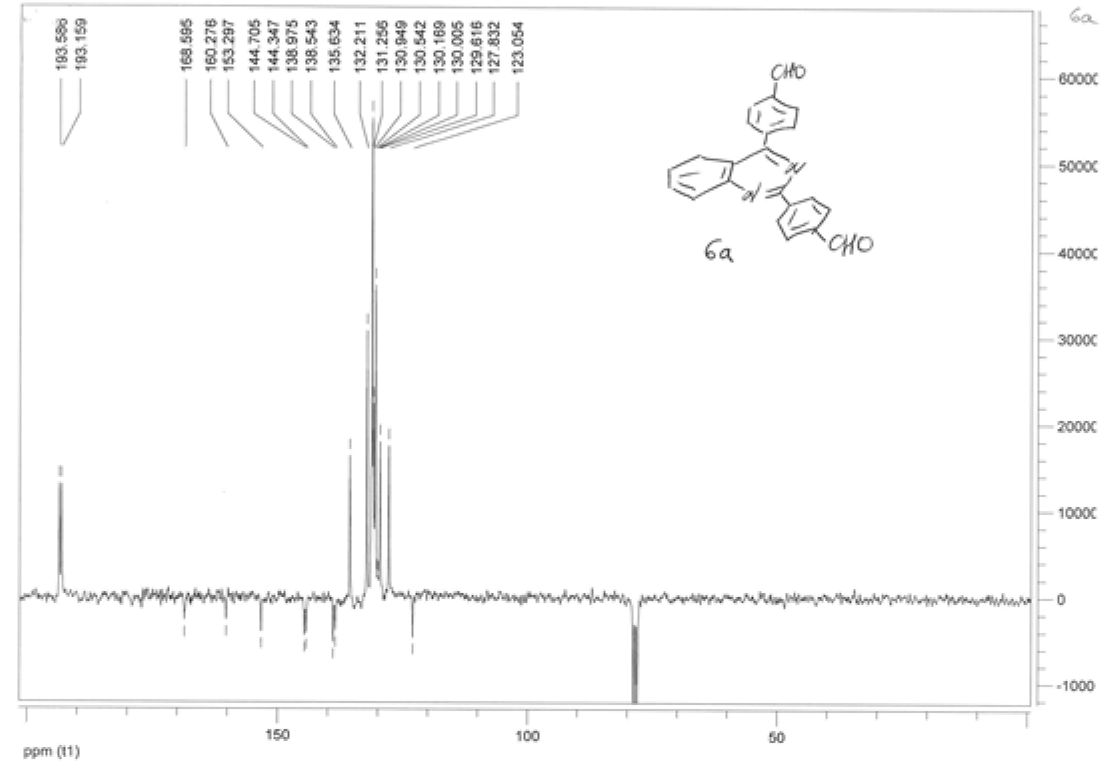

**Fig. S14.** $^{13}$C NMR spectrum of **6a**

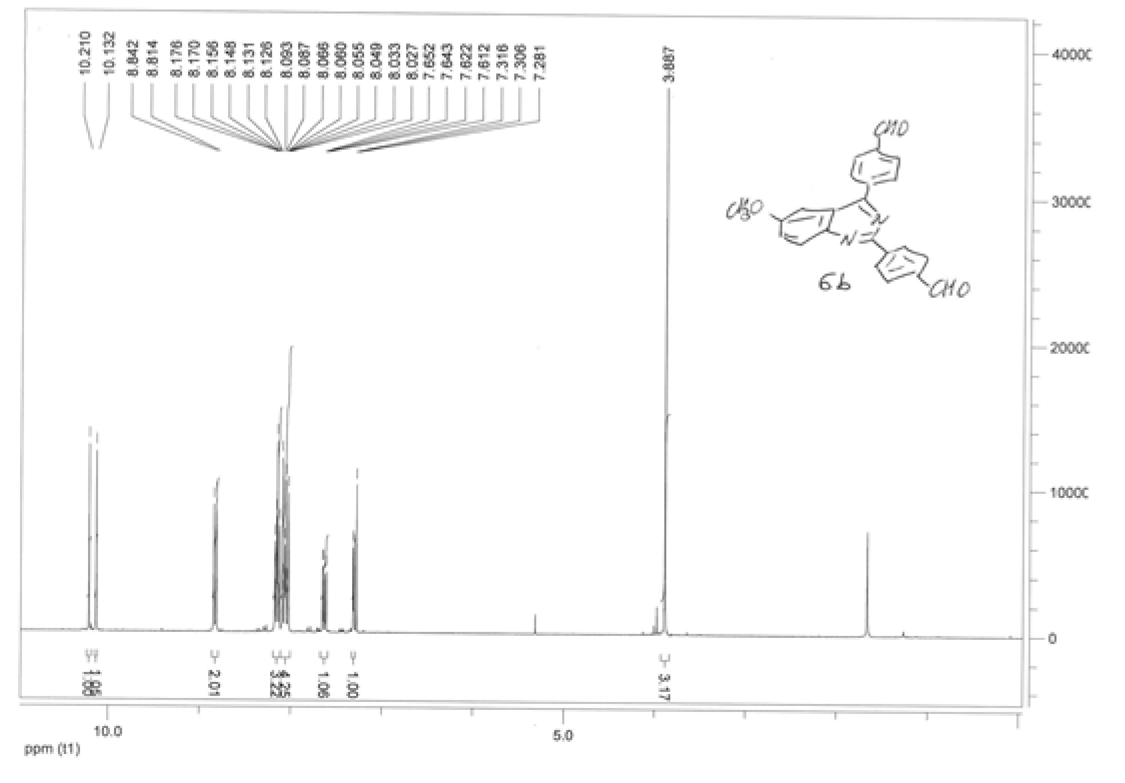

**Fig. S15.** $^{1}$H NMR spectrum of **6b**

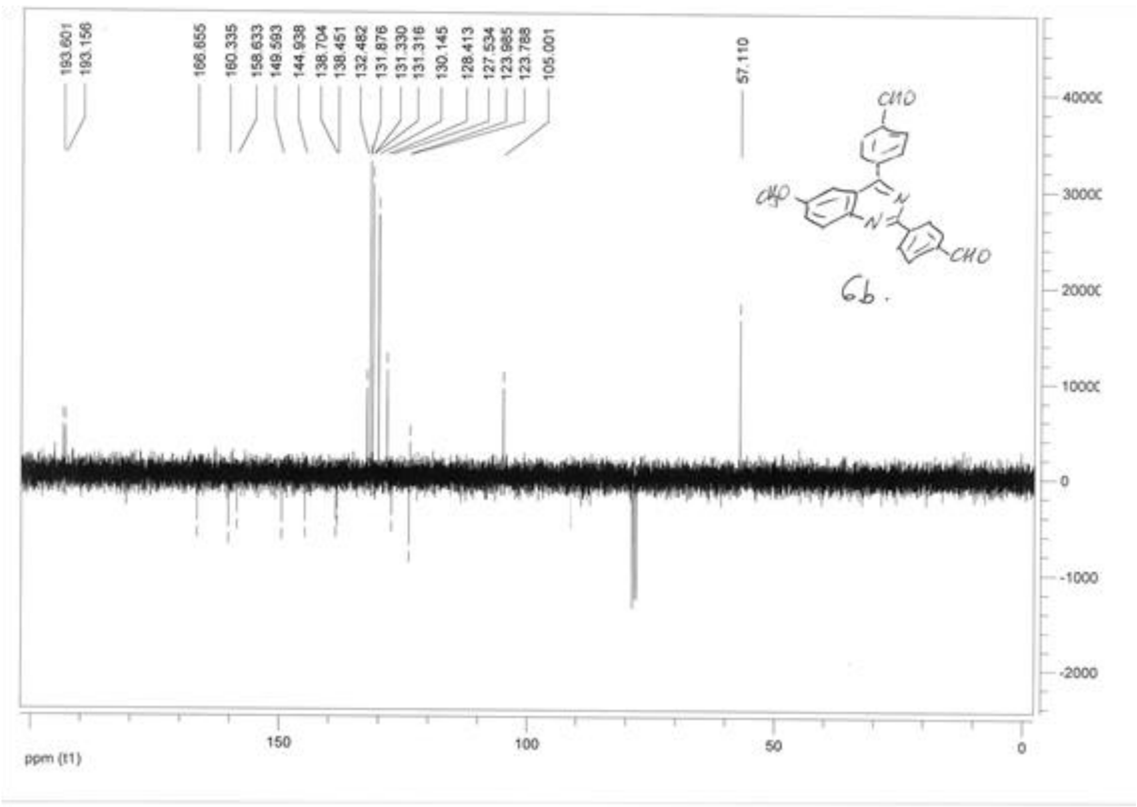

**Fig. S16.** $^{13}$C NMR spectrum of **6b**

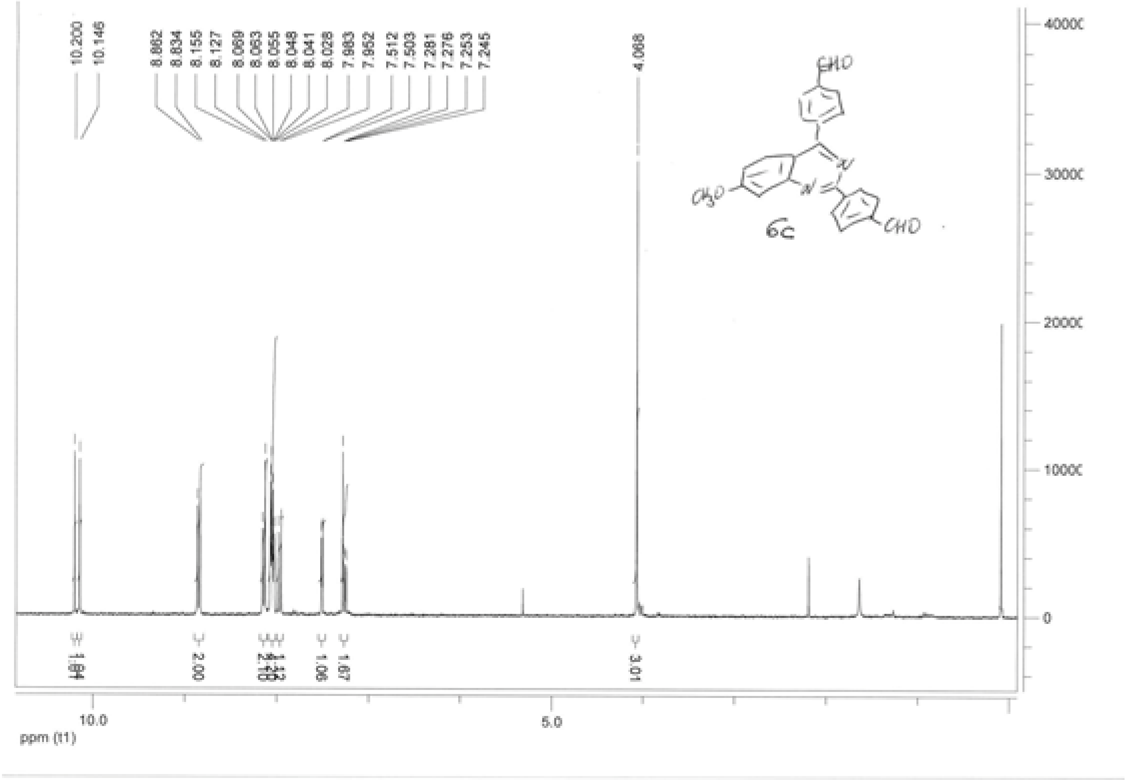


**Fig. S17.** $^{1}$H NMR spectrum of **6c**

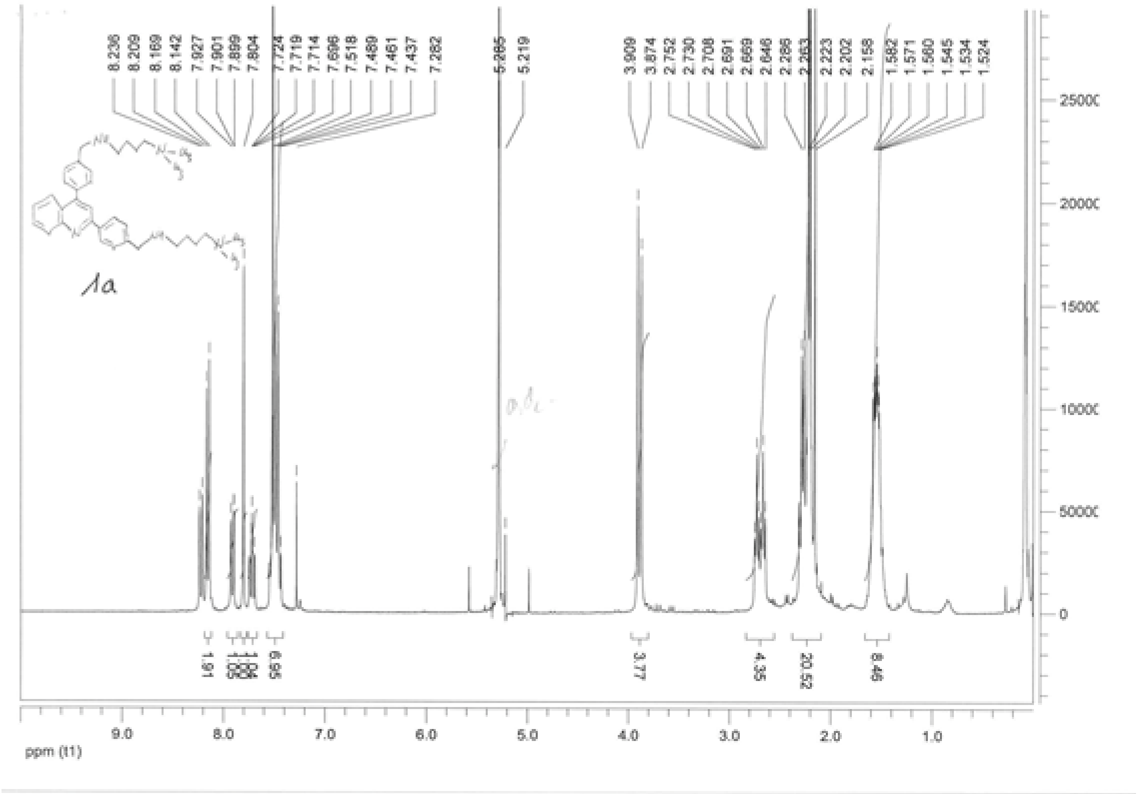

**Fig. S18.** $^{1}$H NMR spectrum of **1a**

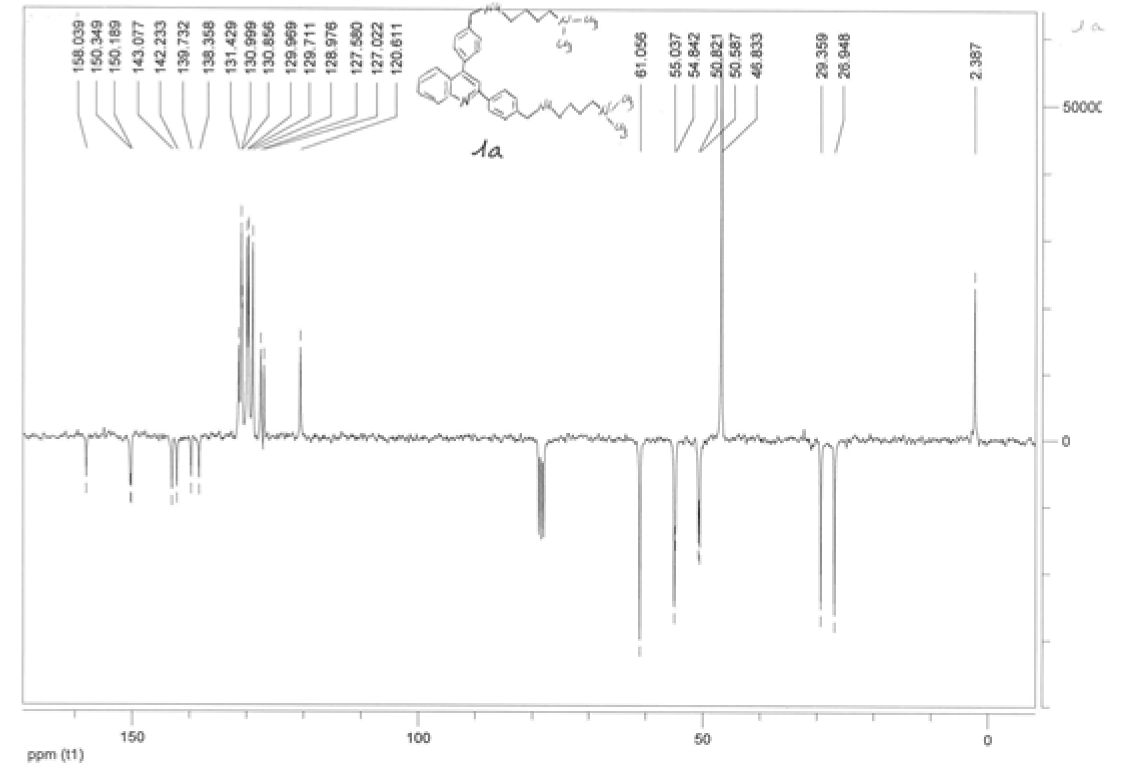

**Fig. S19.** $^{13}$C NMR spectrum of **1a**

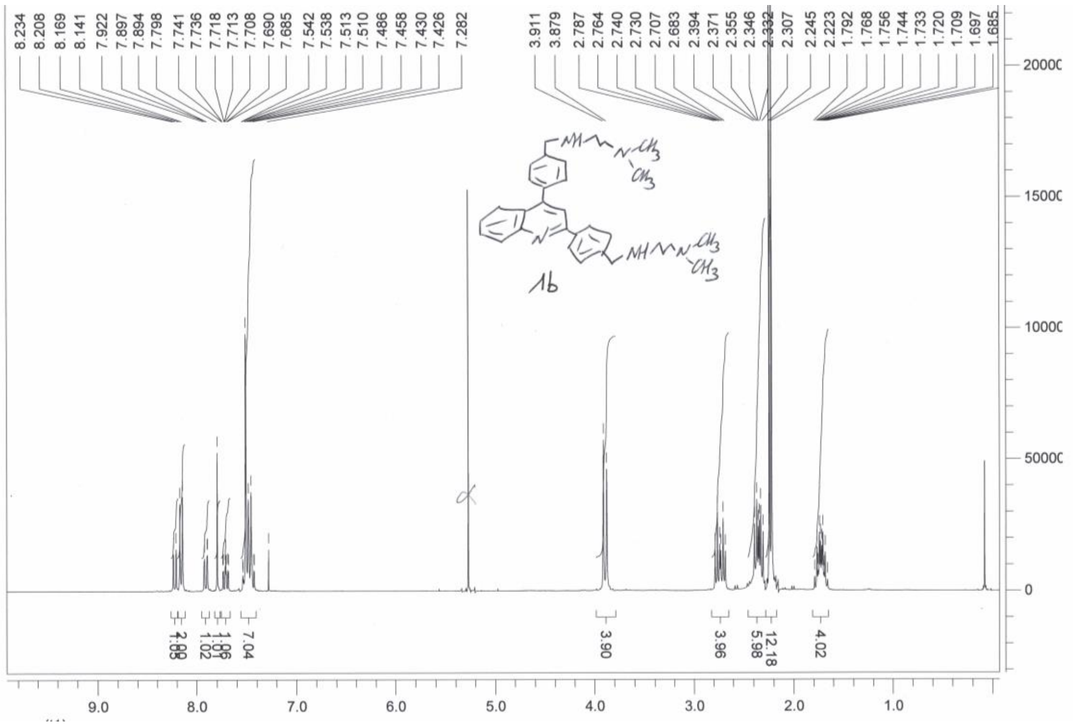


**Fig. S20.** $^{1}$H NMR spectrum of **1b**

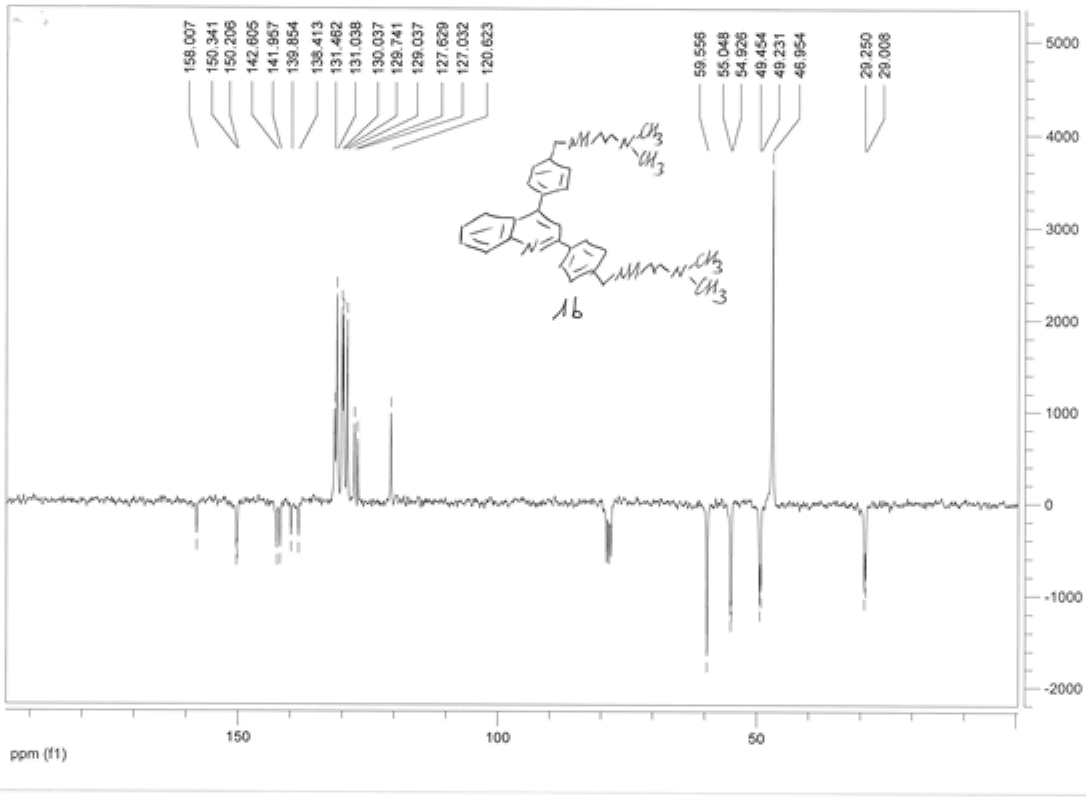


**Fig. S21.** $^{13}$C NMR spectrum of **1b**

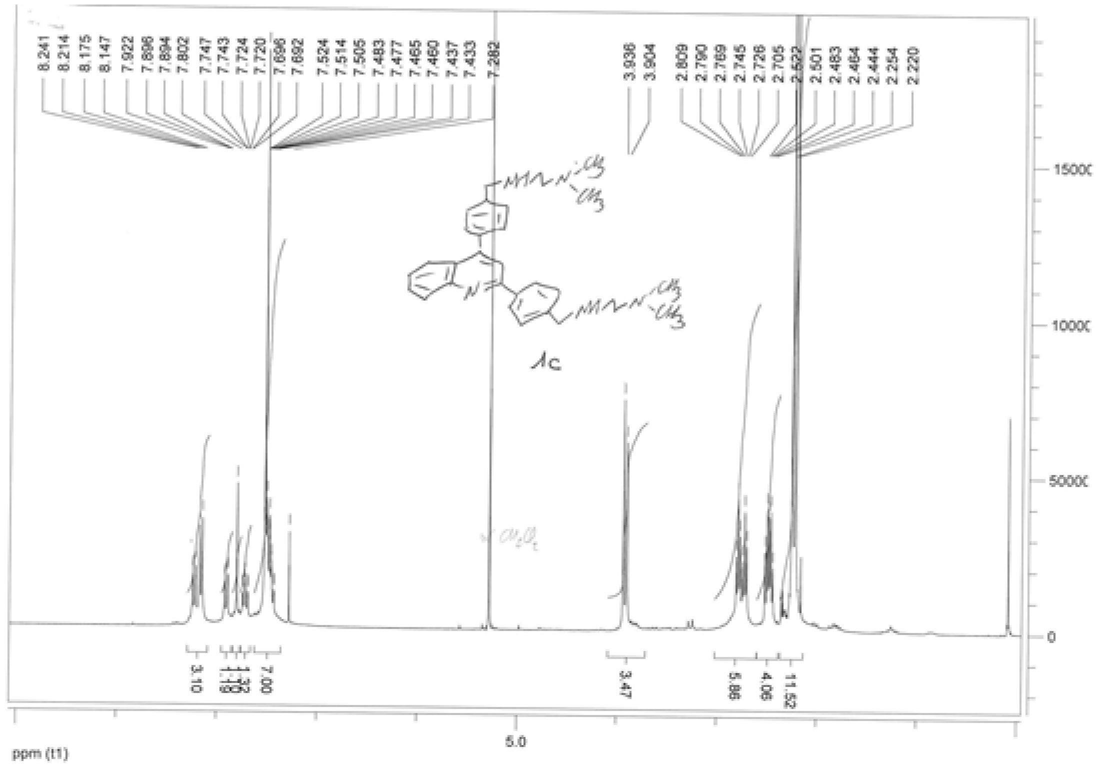

**Fig. S22.** $^1$H NMR spectrum of **1c**

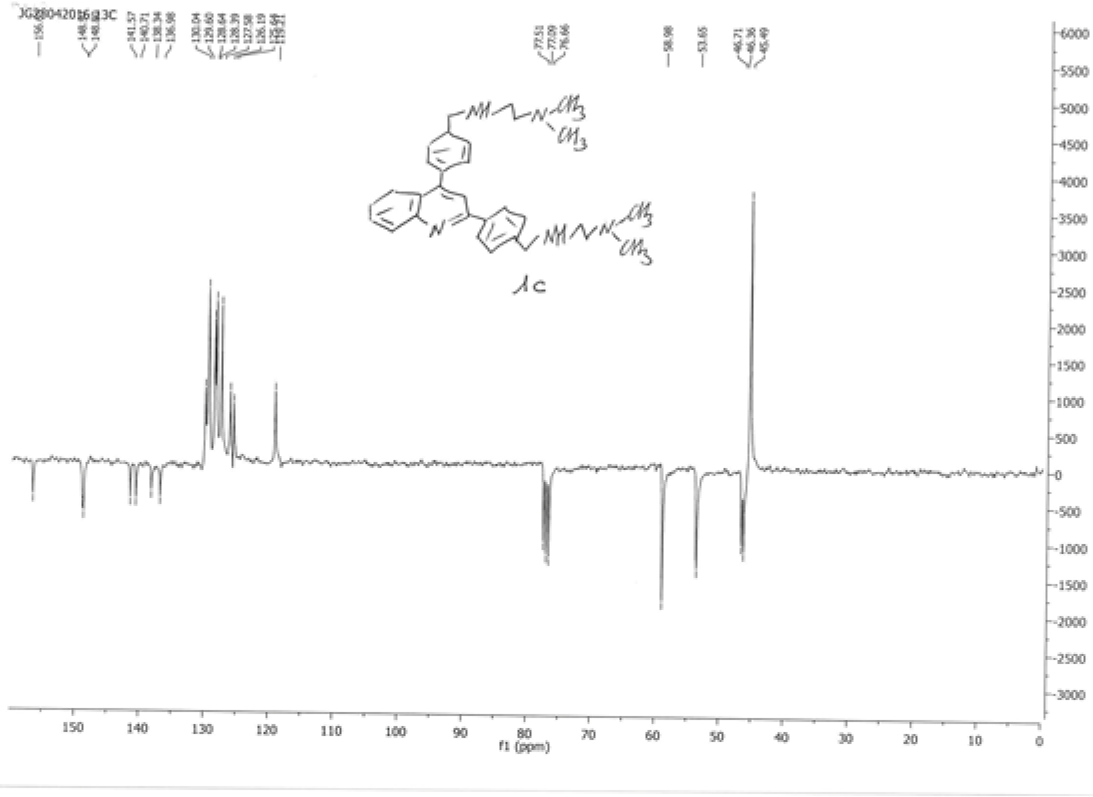

**Fig. S23.** $^{13}$C NMR spectrum of **1c**

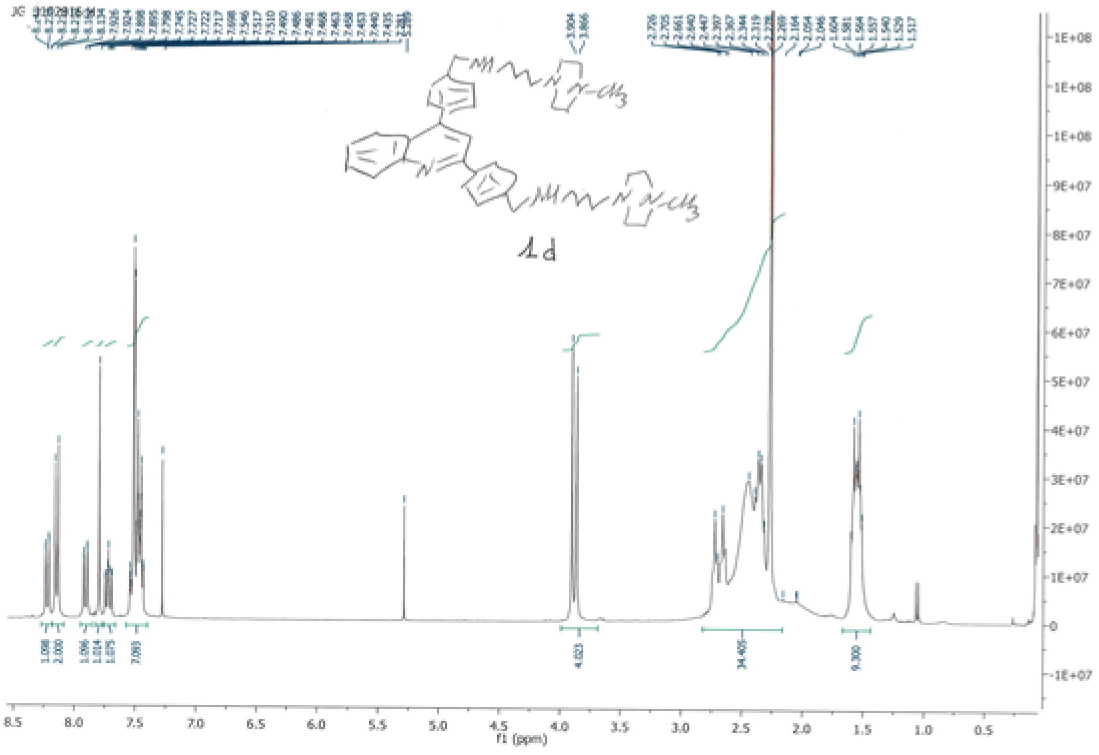

**Fig. S24.** $^{1}$H NMR spectrum of **1d**

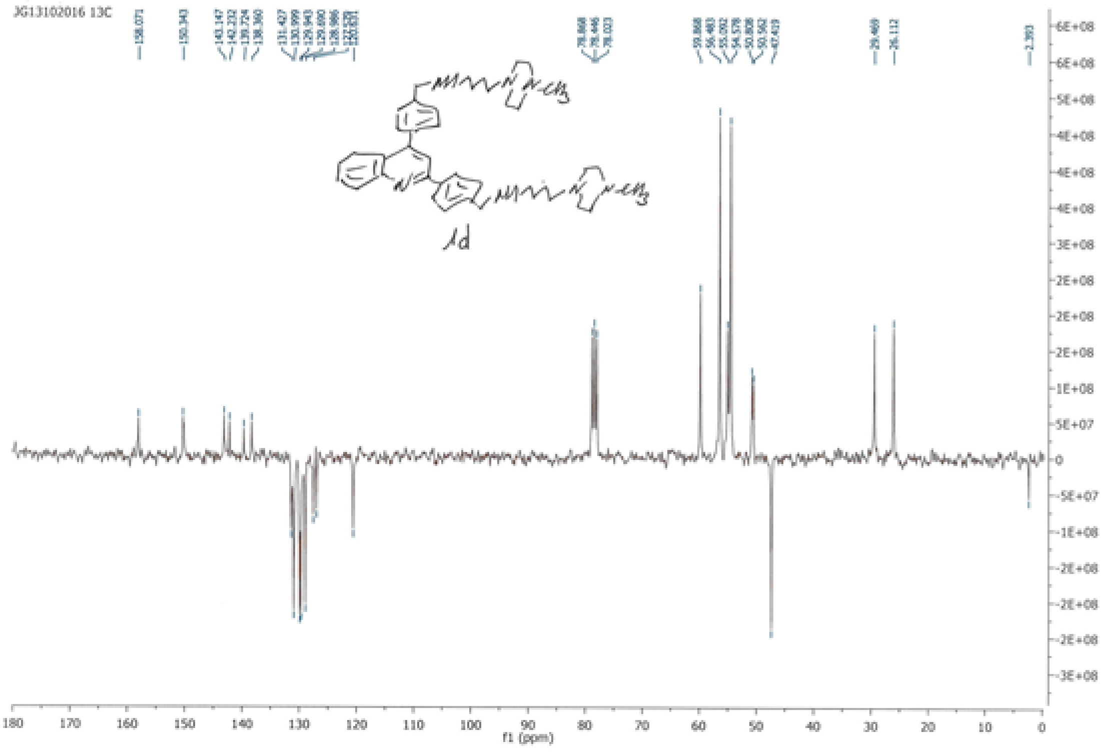

**Fig. S25.** $^{13}$C NMR spectrum of **1d**

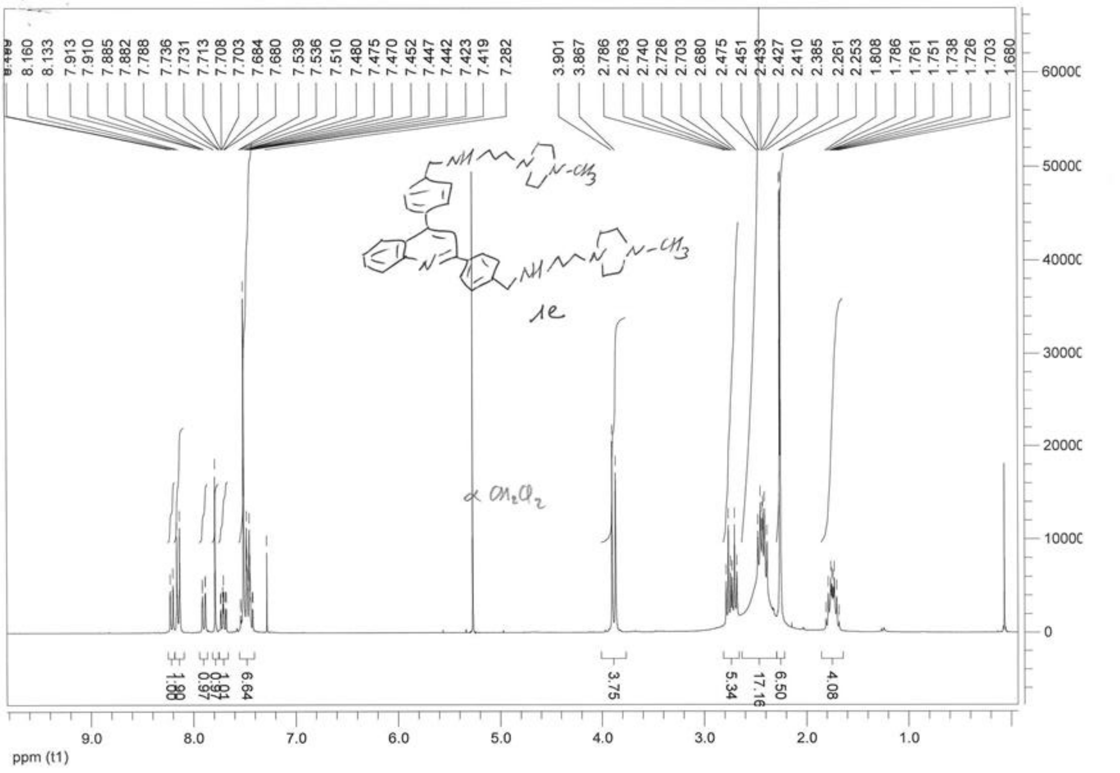

**Fig. S26.** $^{1}$H NMR spectrum of **1e**

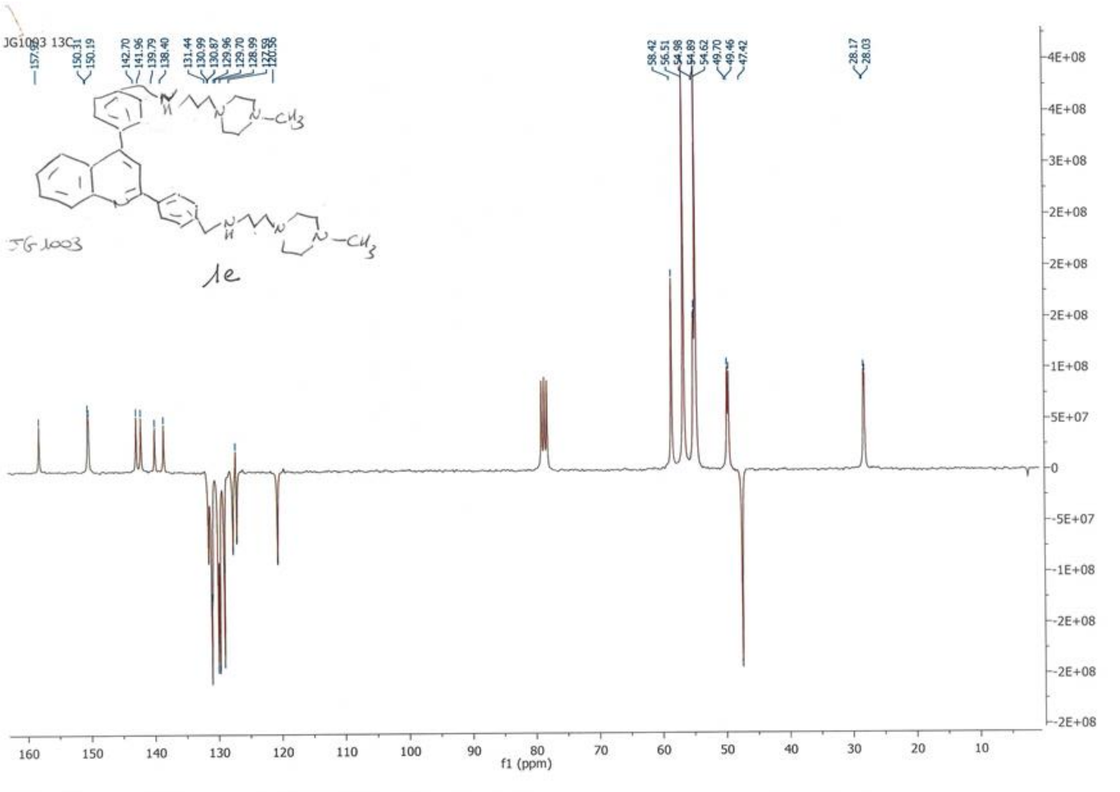

**Fig. S27.** $^{13}$C NMR spectrum of **1e**

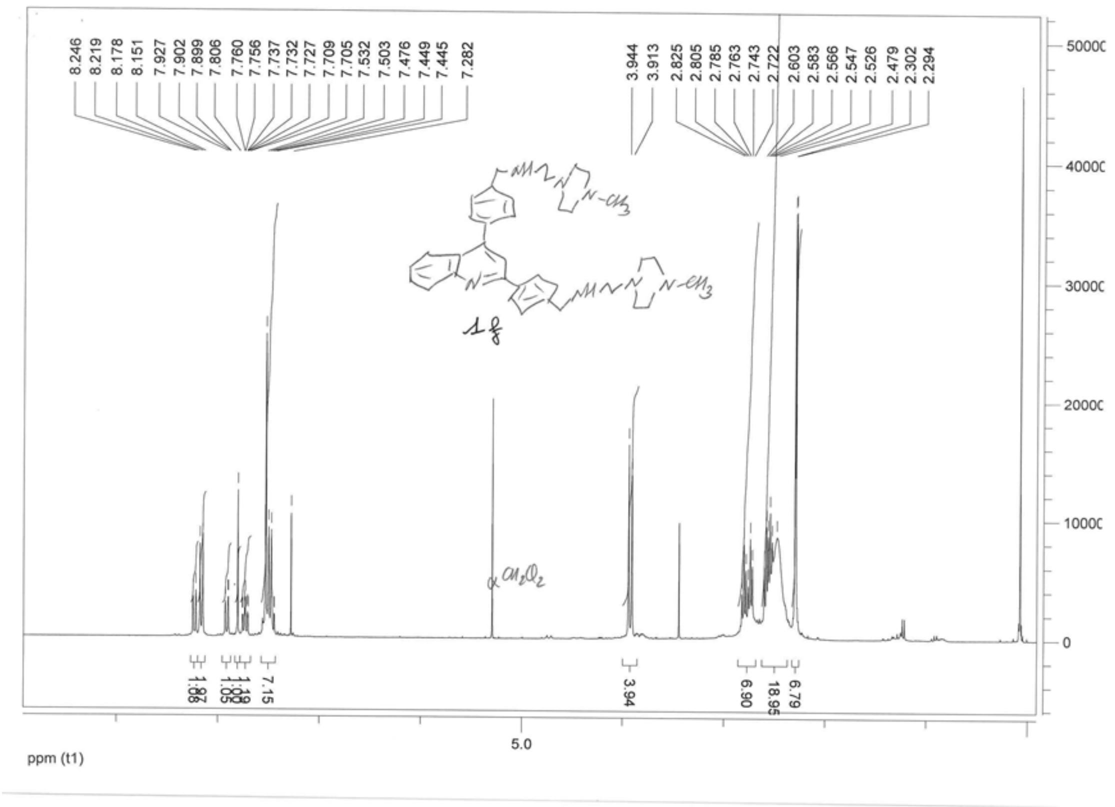

**Fig. S28.** $^{1}$H NMR spectrum of **1f**

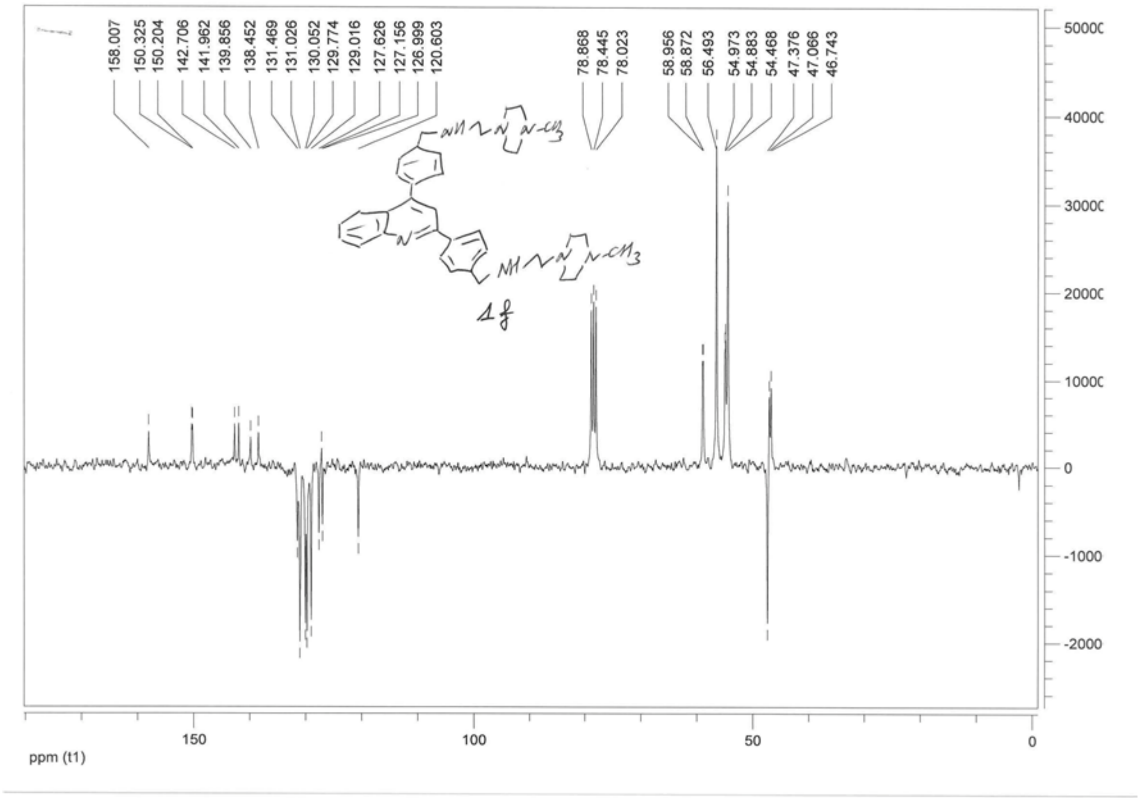

**Fig. S29.** $^{13}$C NMR spectrum of **1f**

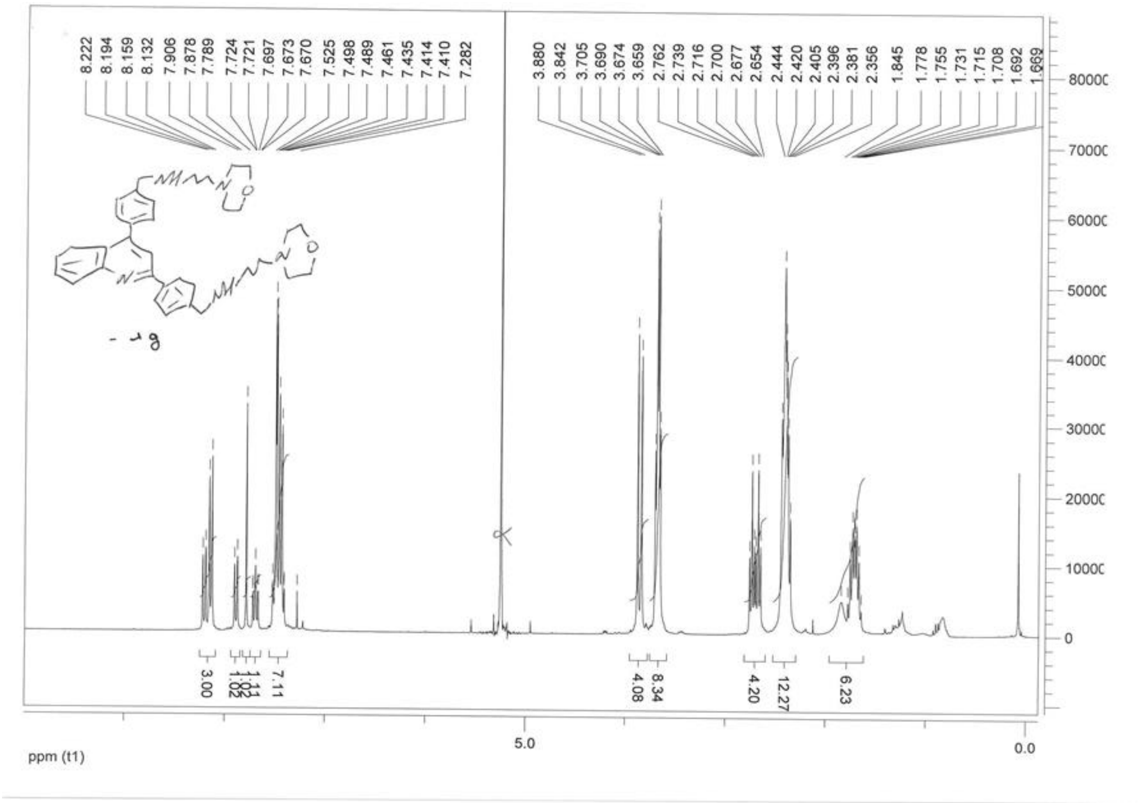

**Fig. S30.** $^{1}$H NMR spectrum of **1g**

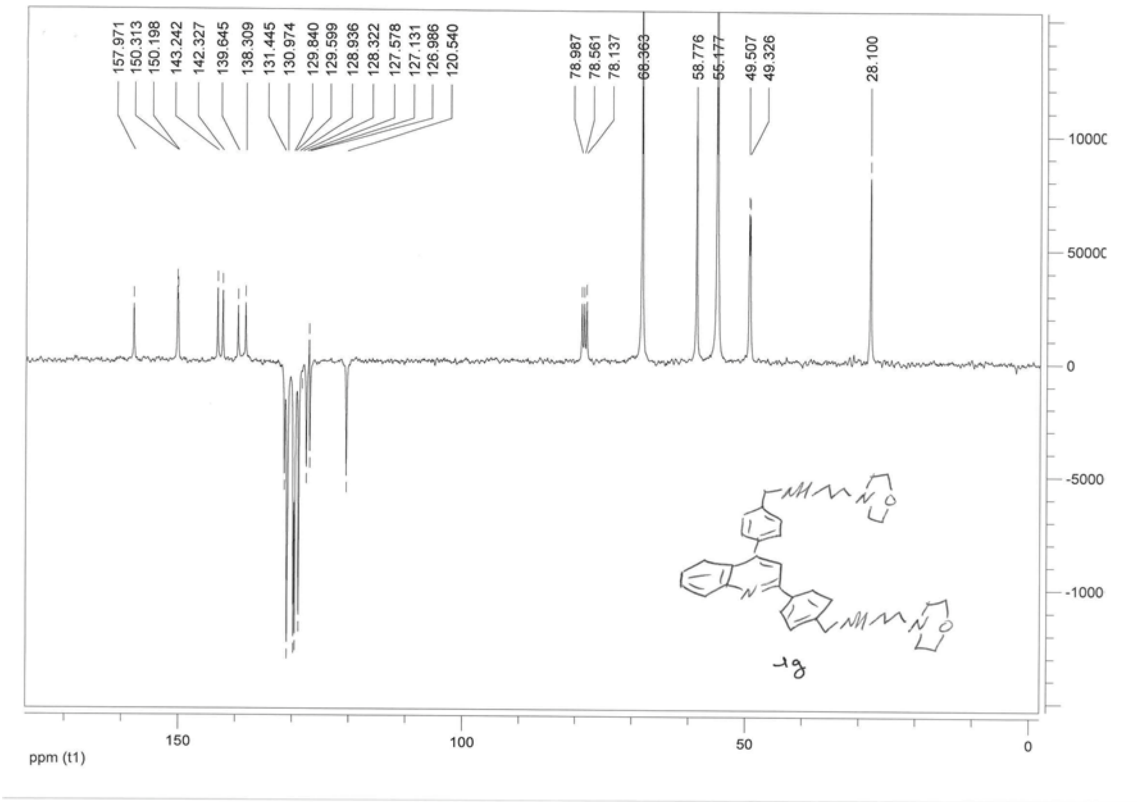

**Fig. S31.** $^{13}$C NMR spectrum of **1g**

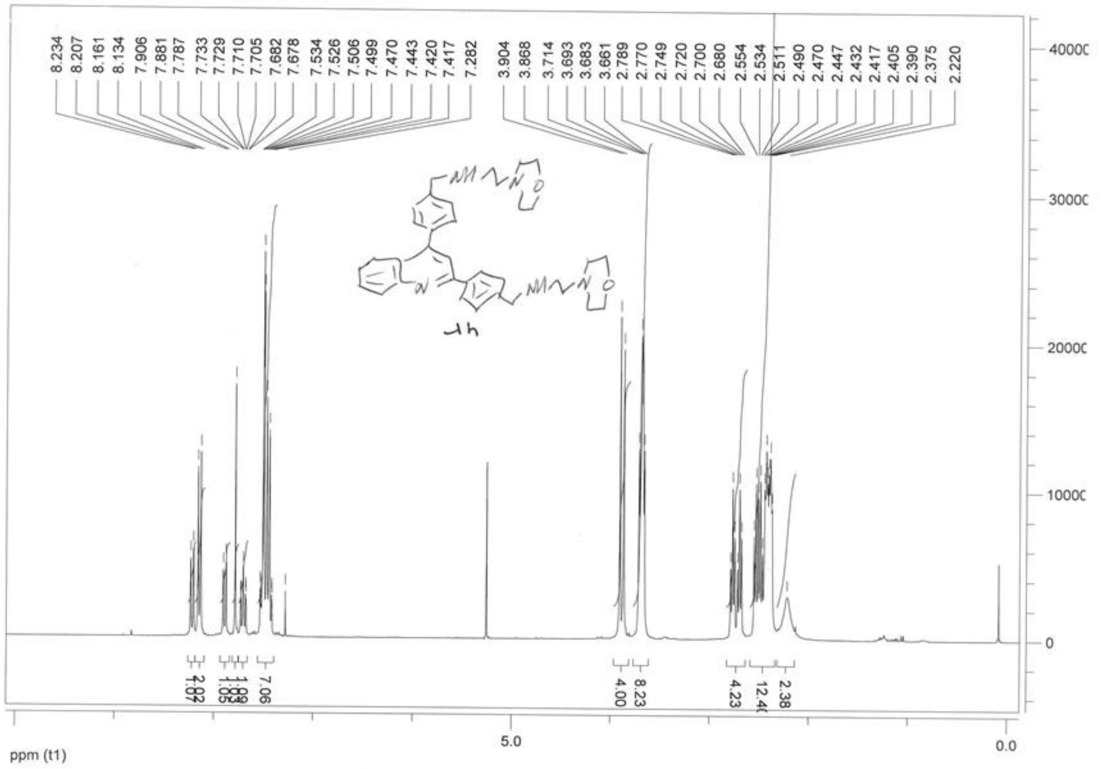

**Fig. S32.** $^{1}$H NMR spectrum of **1h**

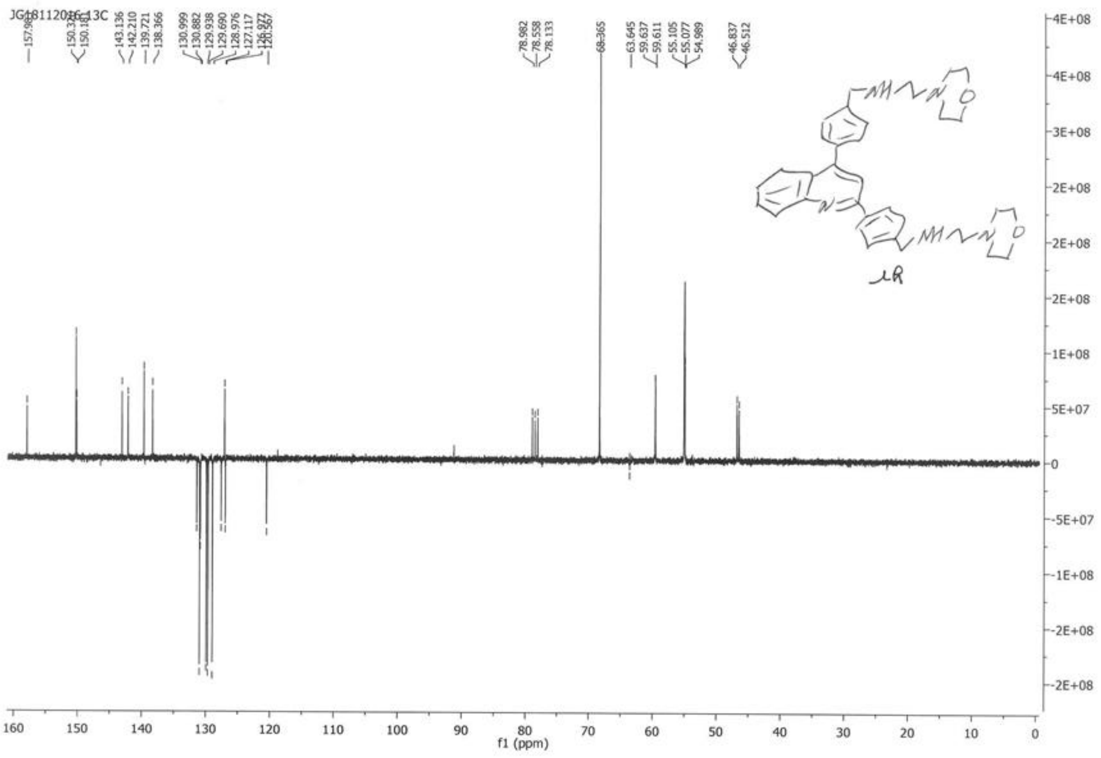

**Fig. S32.** $^{13}$C NMR spectrum of **1h**

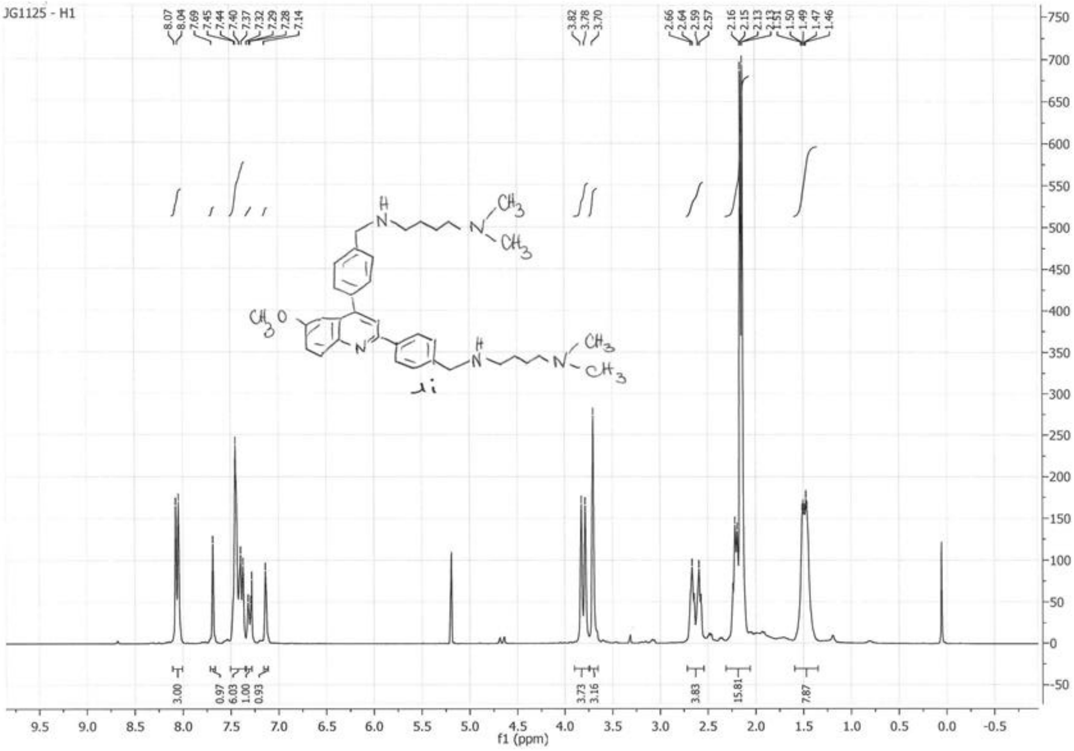

**Fig. S33.** $^{1}$H NMR spectrum of **1i**

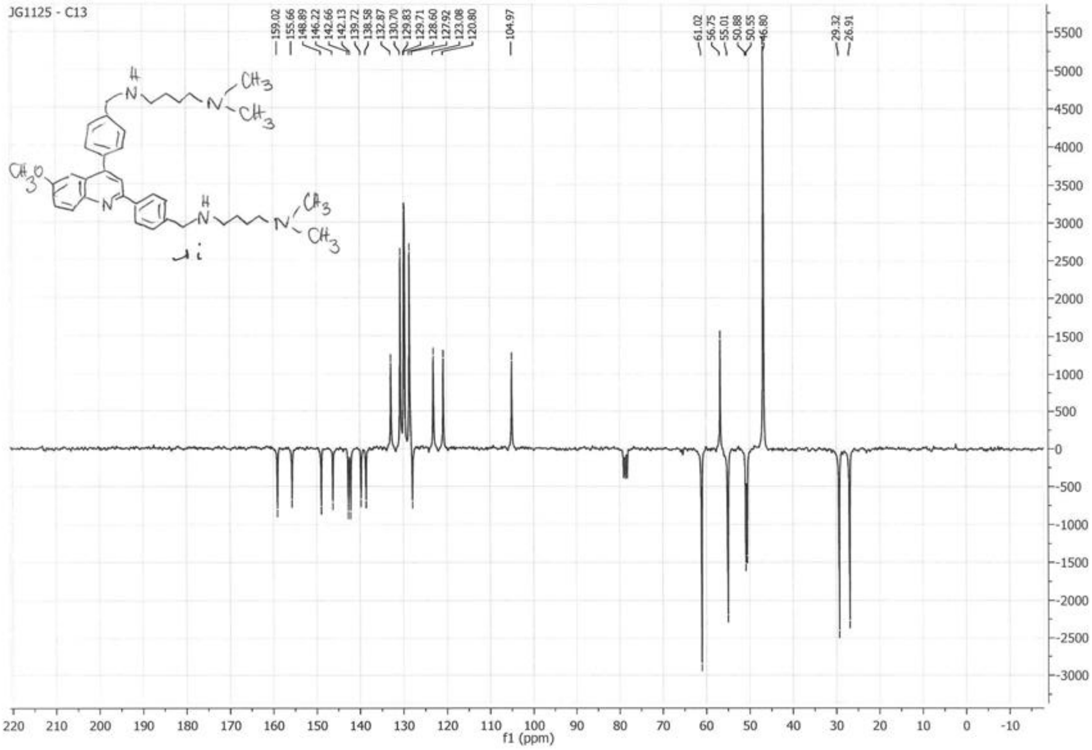

**Fig. S34.** $^{13}$C NMR spectrum of **1i**

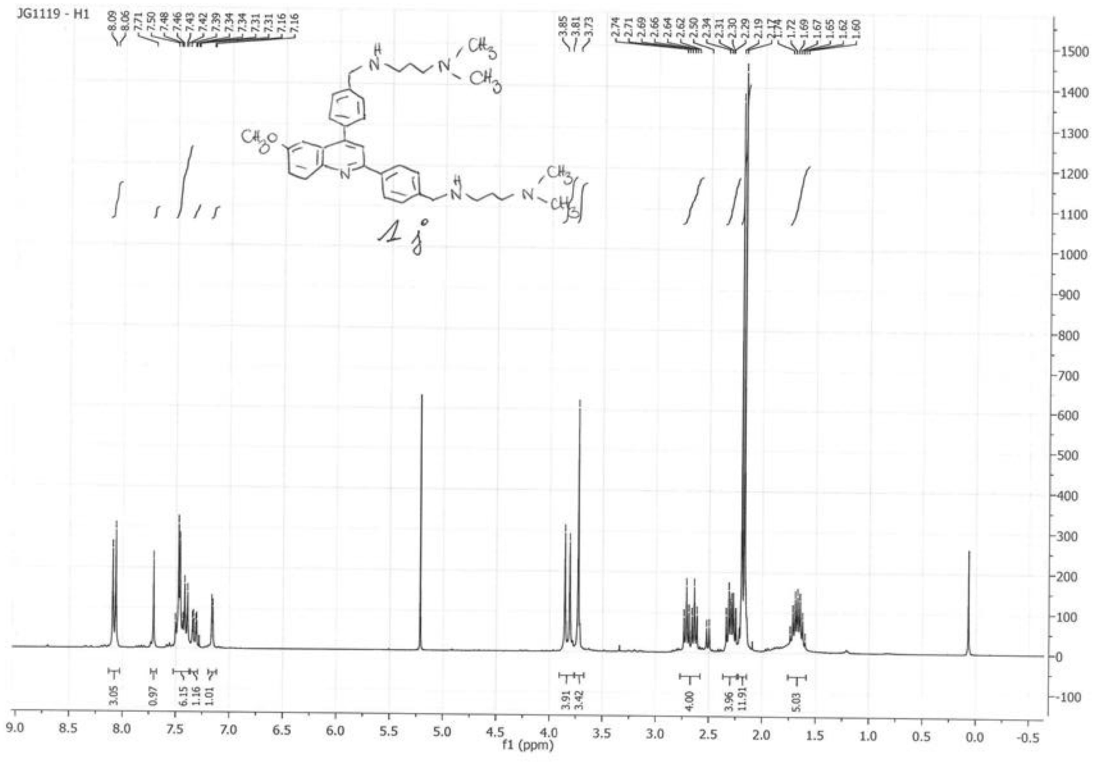

**Fig. S35.** $^{1}H$ NMR spectrum of **1j**

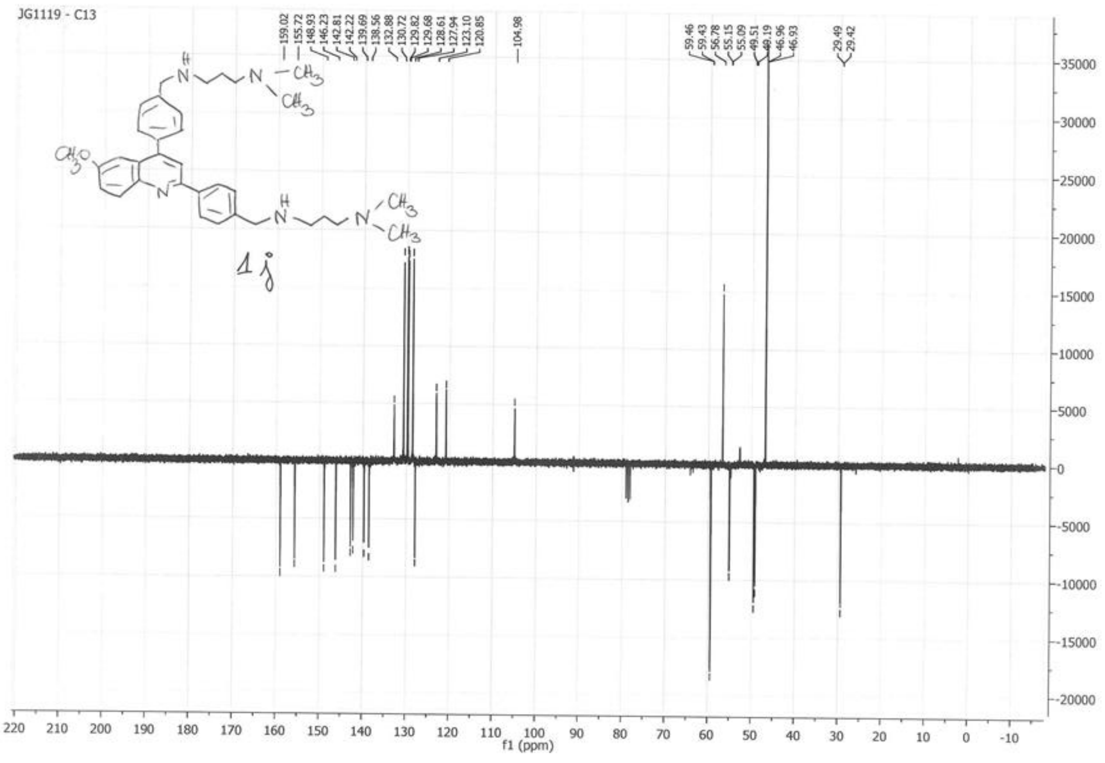

**Fig. 36.** $^{13}C$ NMR spectrum of **1j**

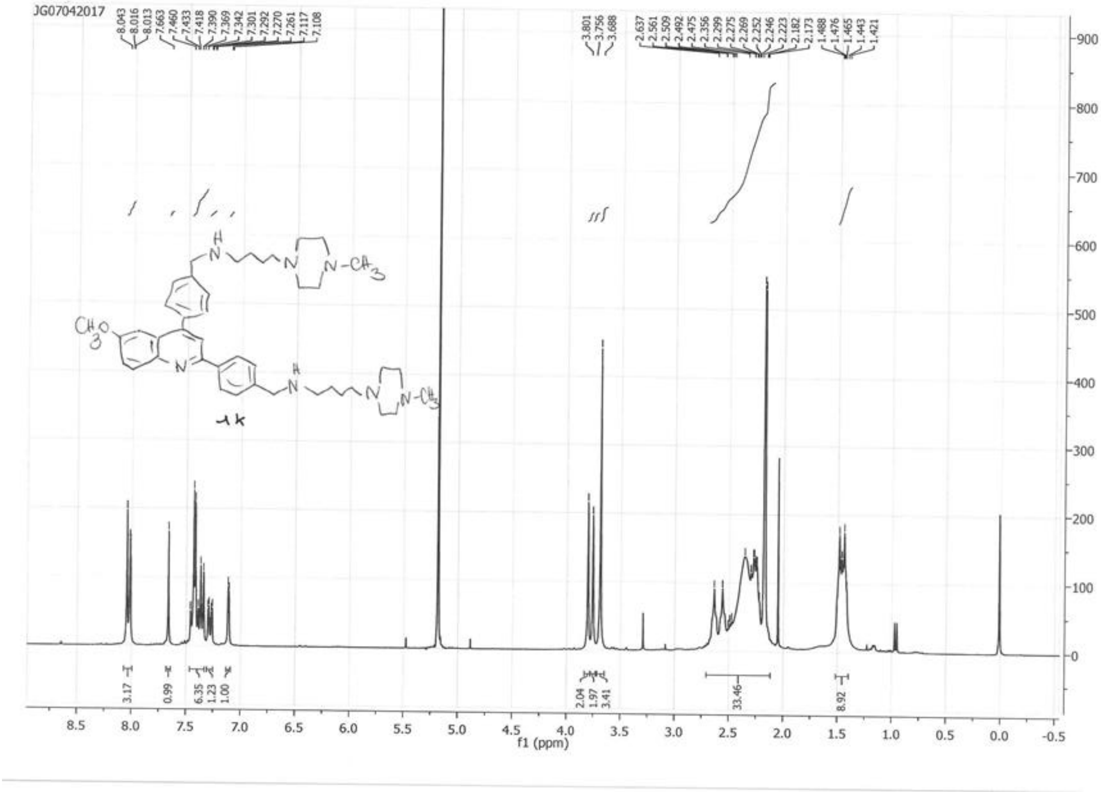

**Fig. S37.** $^{1}$H NMR spectrum of **1k**

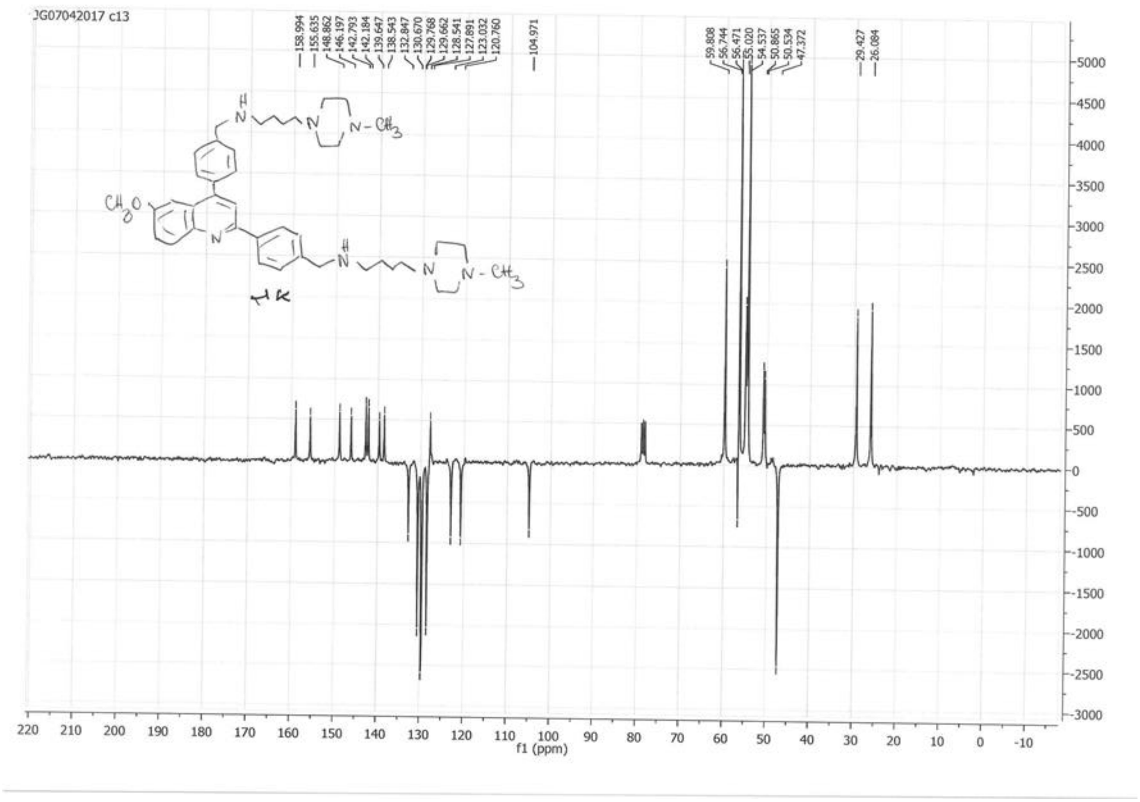

**Fig. S38.** $^{13}$C NMR spectrum of **1k**

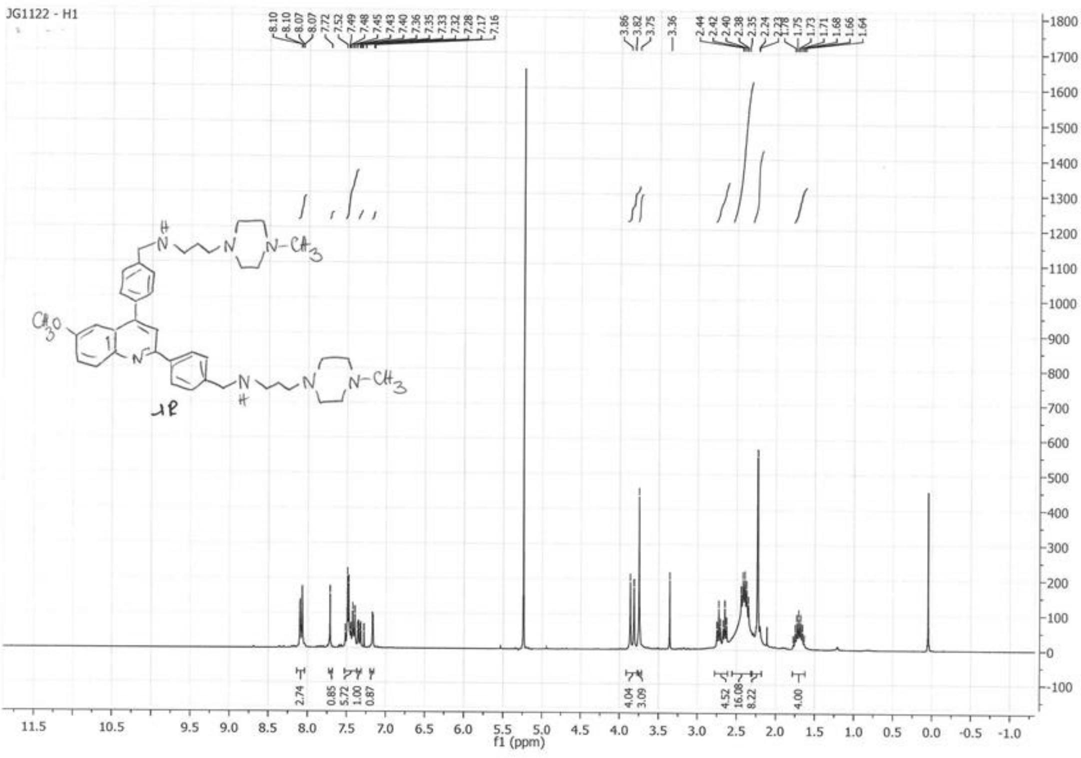

**Fig. S39.** $^{1}$H NMR spectrum of **1l**

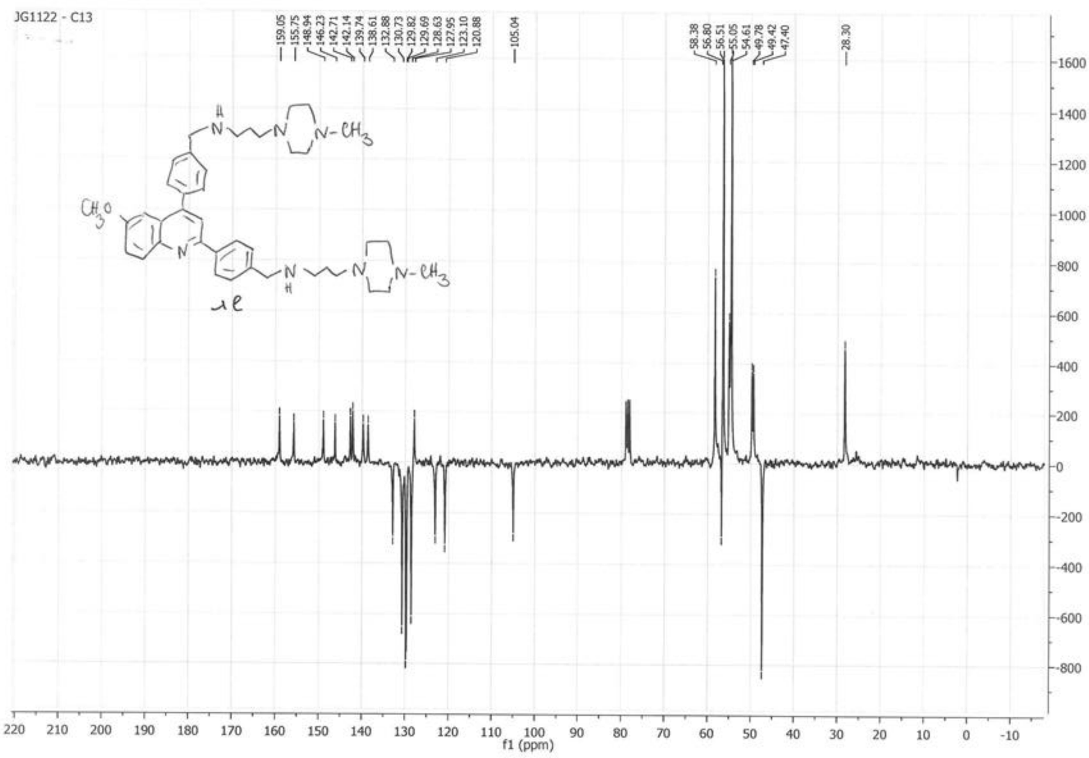

**Fig. S40.** $^{13}$C NMR spectrum of **1l**

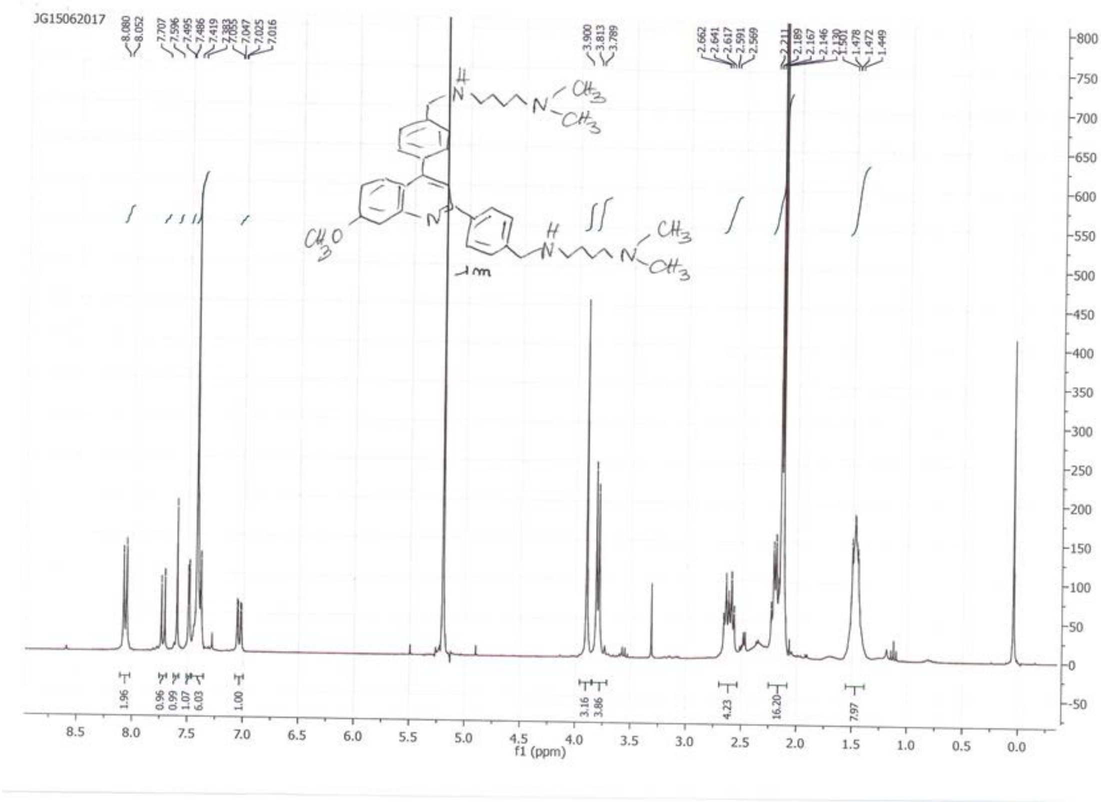

**Fig. S41.** $^1$H NMR spectrum of **1m**

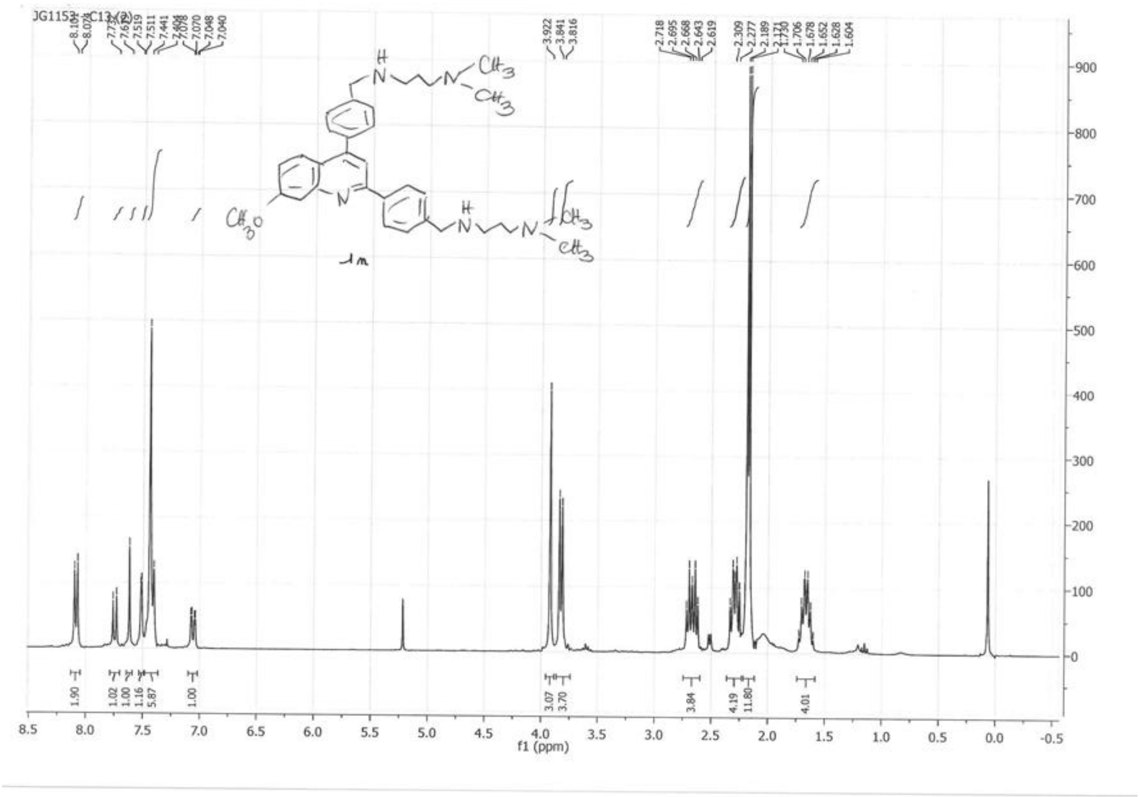

**Fig. S42.** $^{1}$H NMR spectrum of **1n**

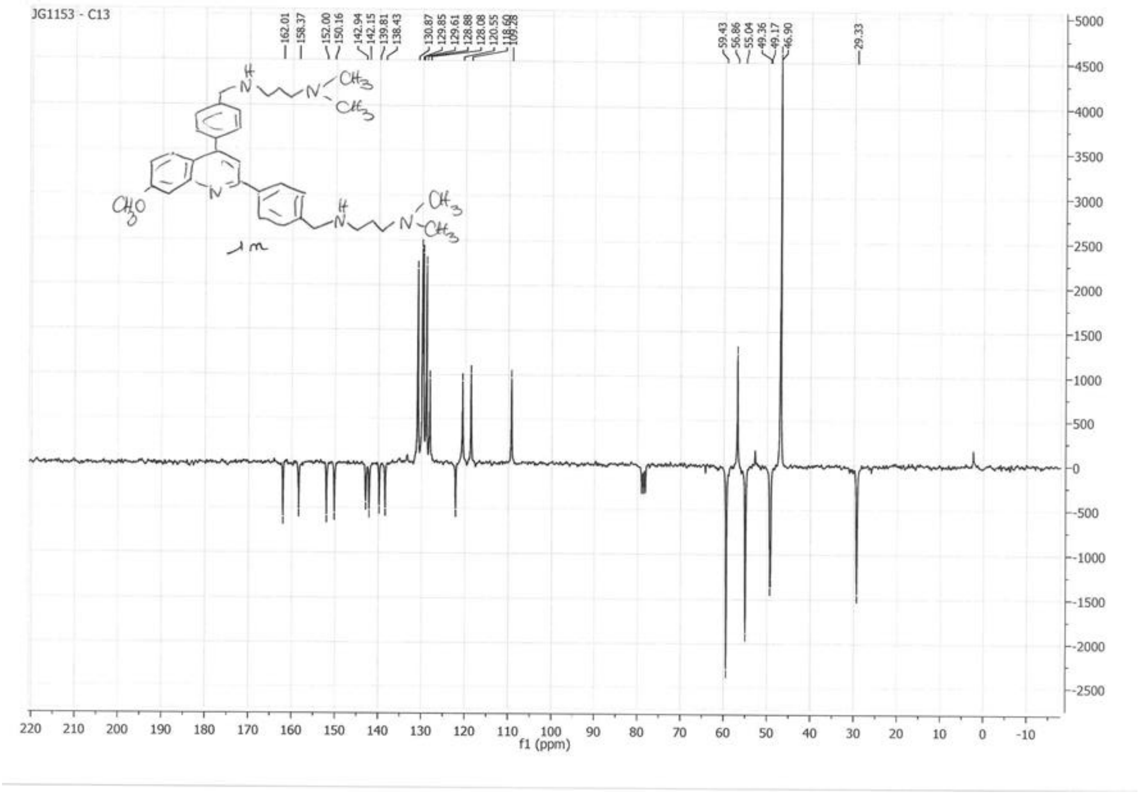

**Fig. S43.** $^{13}$C NMR spectrum of **1n**

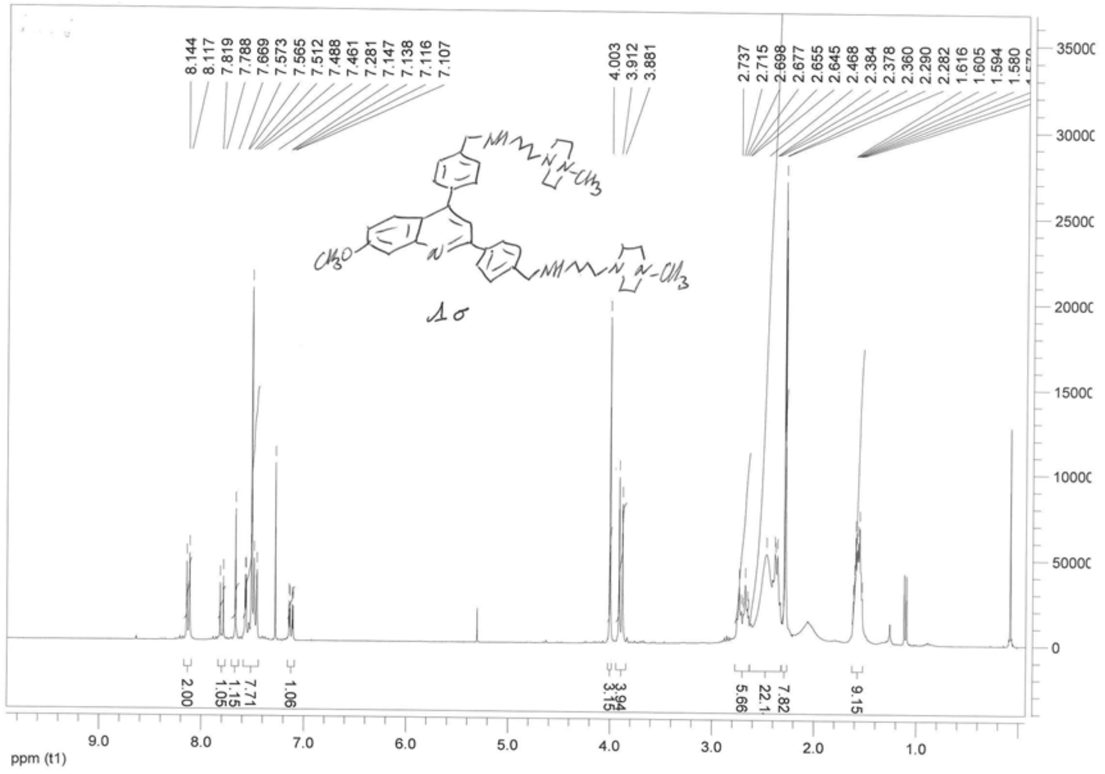

**Fig. S44.** $^{1}H$ NMR spectrum of **1o**

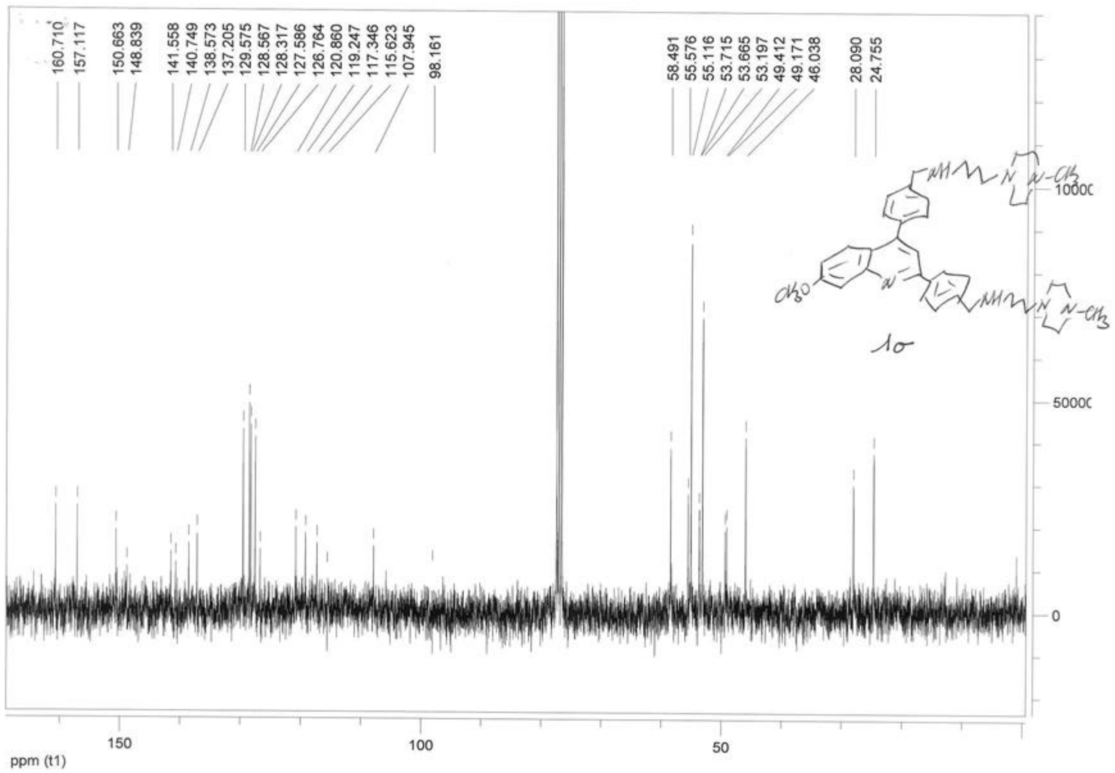

**Fig. S45.** $^{13}C$ NMR spectrum of **1o**

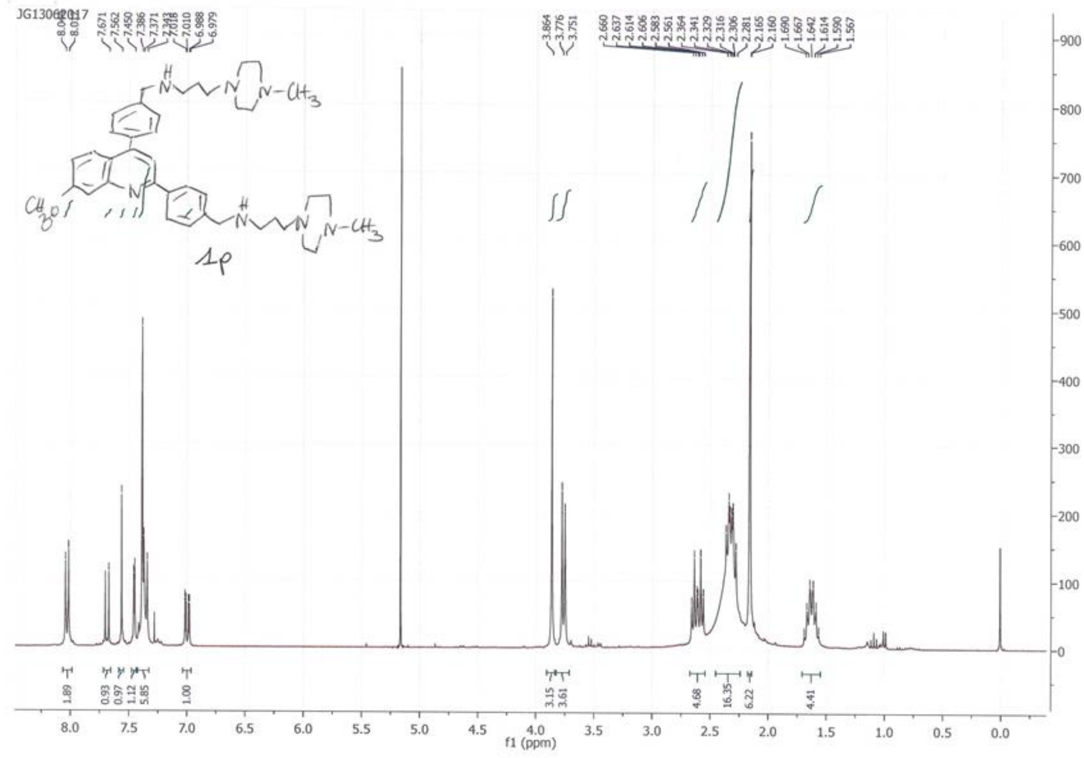

**Fig.S46.** $^{1}$H NMR spectrum of **1p**

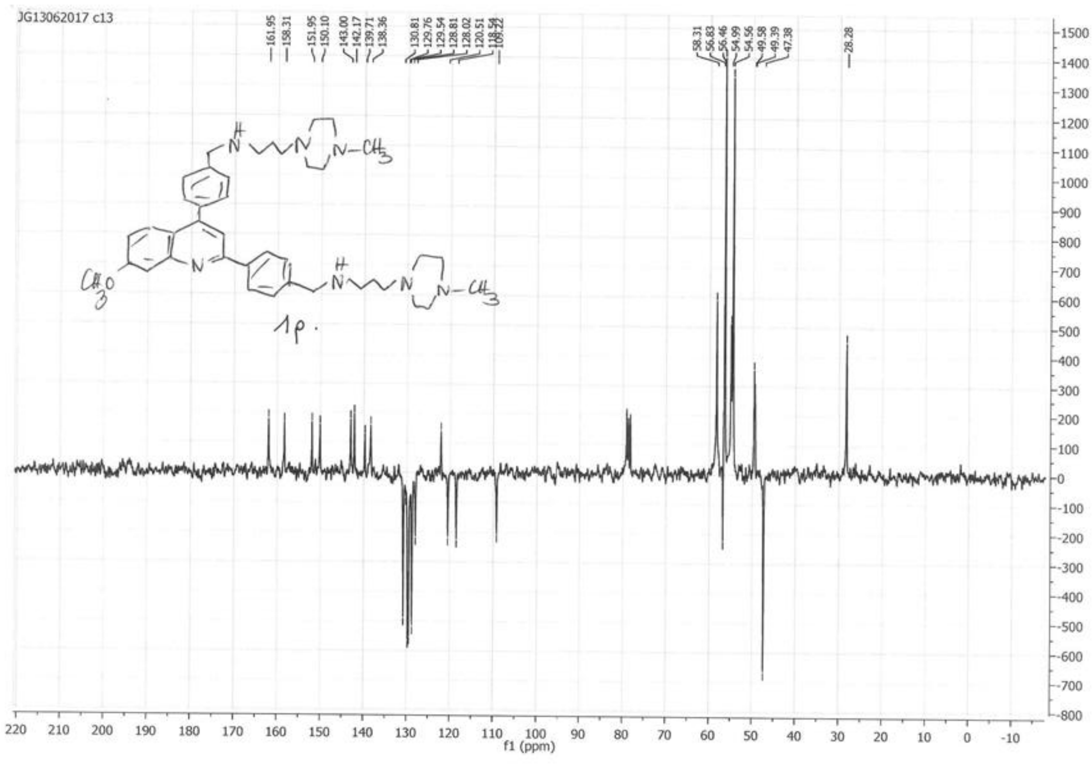

**Fig.S47.** $^{13}$C NMR spectrum of **1p**

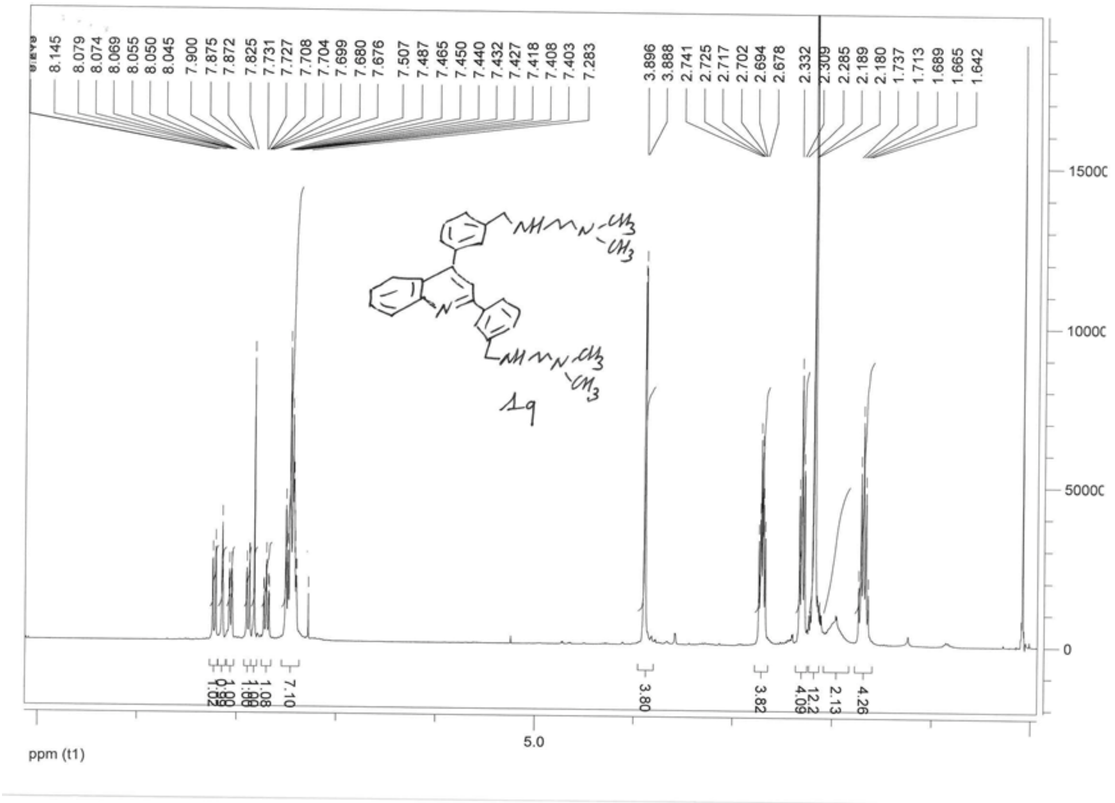

**Fig.S48.** $^{1}$H NMR spectrum of **1q**

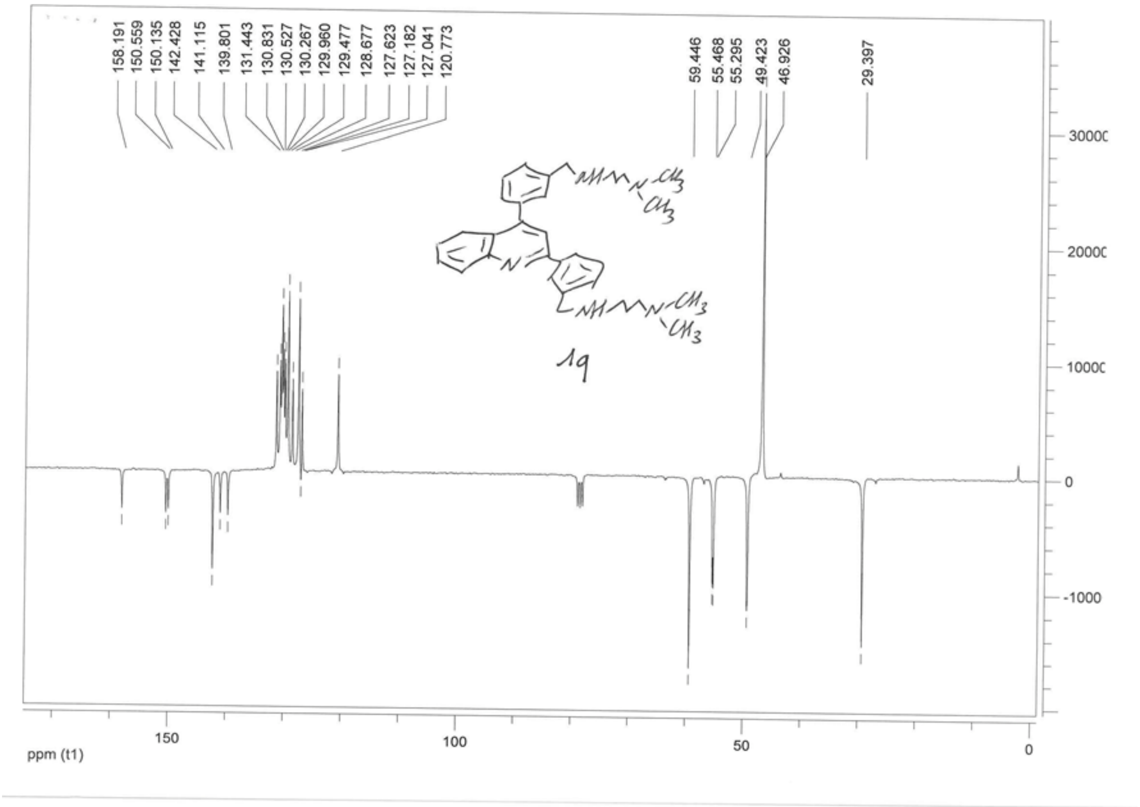

**Fig.S49.** $^{13}$C NMR spectrum of **1q**

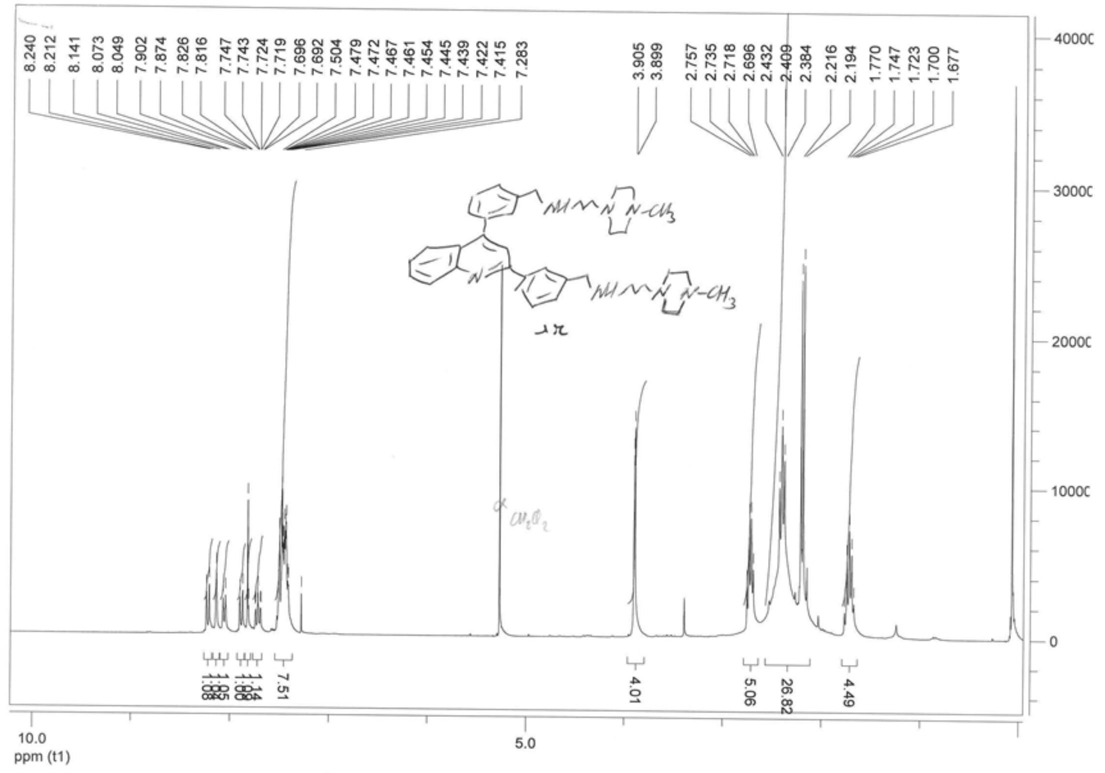

**Fig.S50.** $^{1}$H NMR spectrum of **1r**

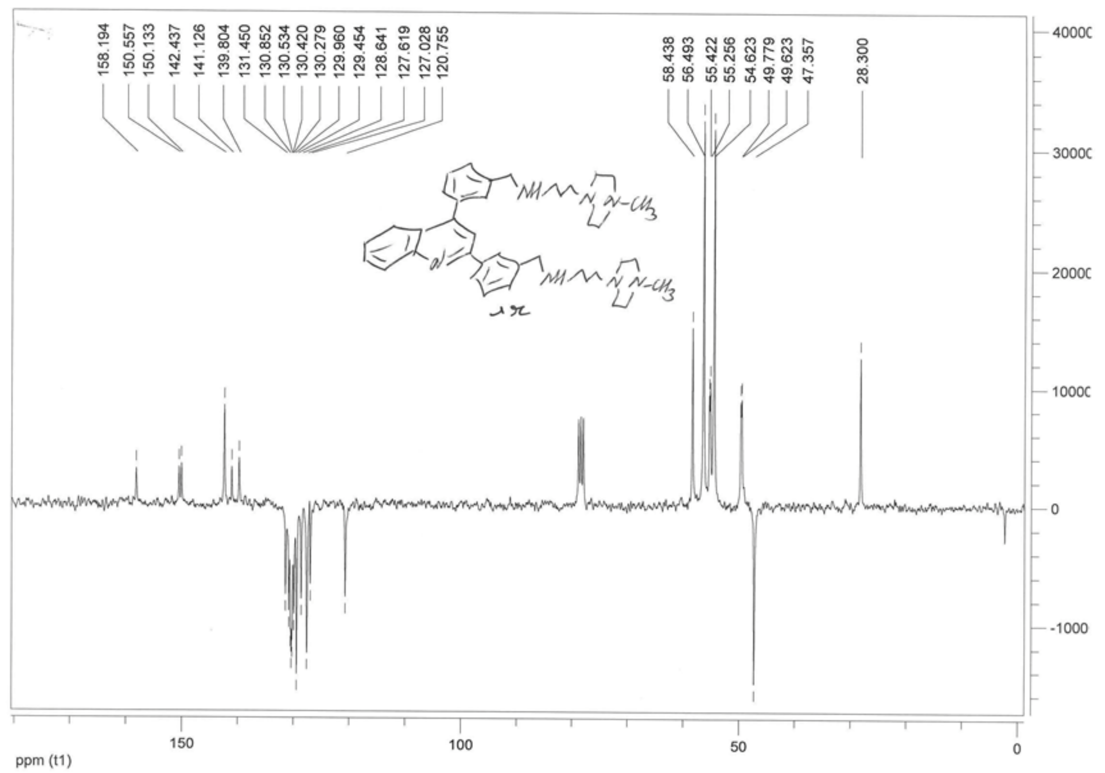

**Fig.S51.** $^{13}$C NMR spectrum of **1r**

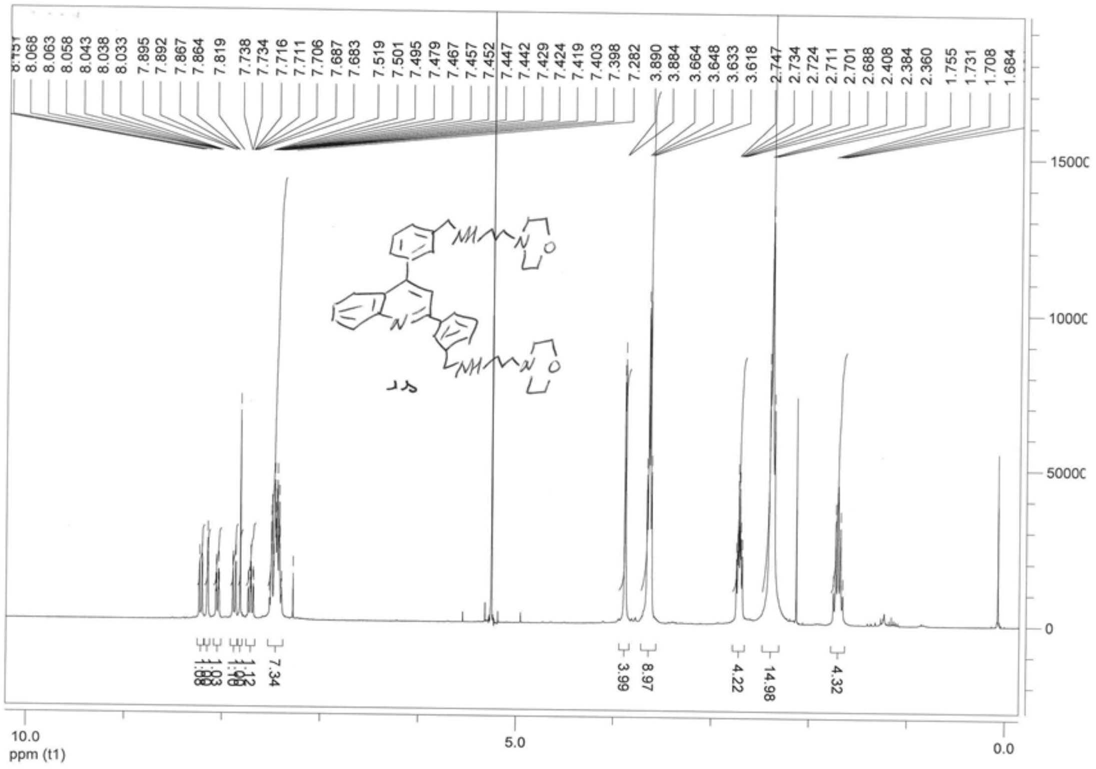

**Fig.S52.** $^{1}$H NMR spectrum of **1s**

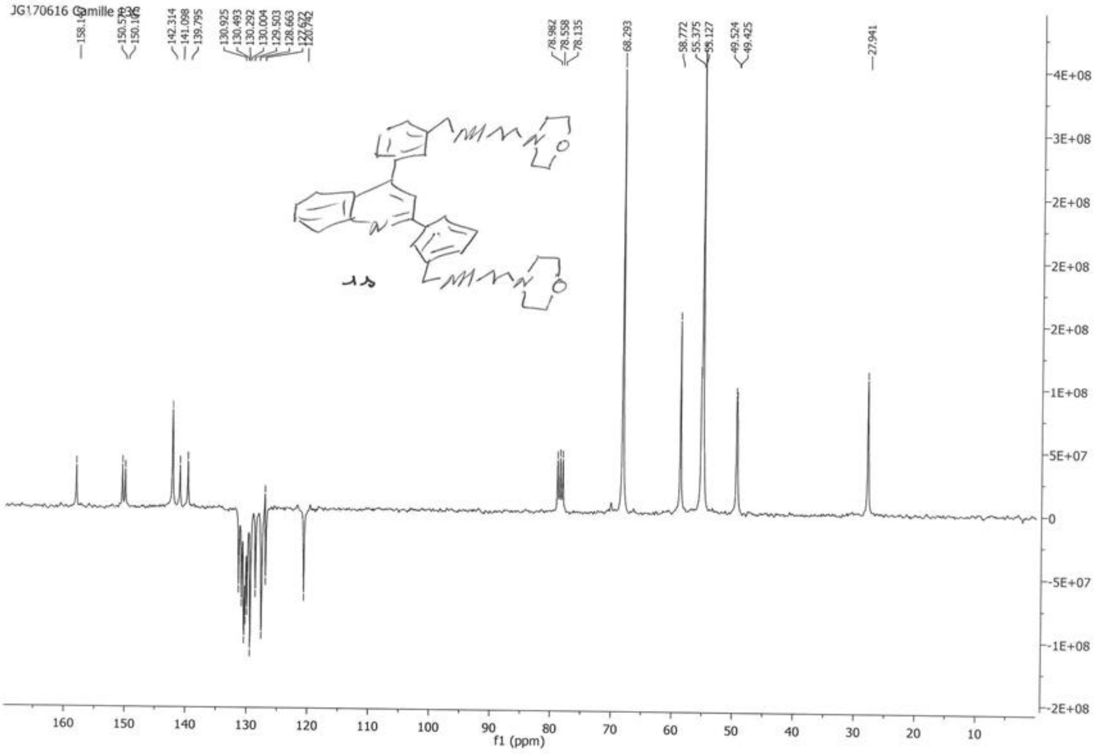

**Fig.S53.** $^{13}$C NMR spectrum of **1s**

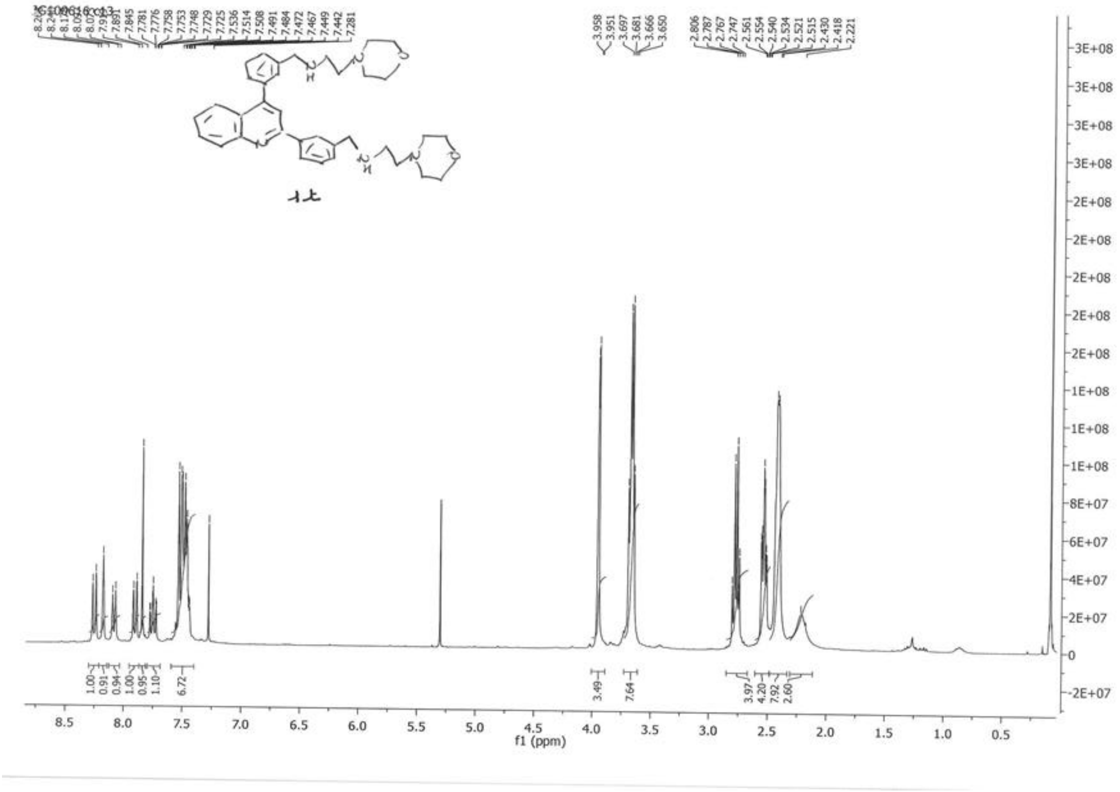

**Fig.S54.** $^{1}$H NMR spectrum of **1t**

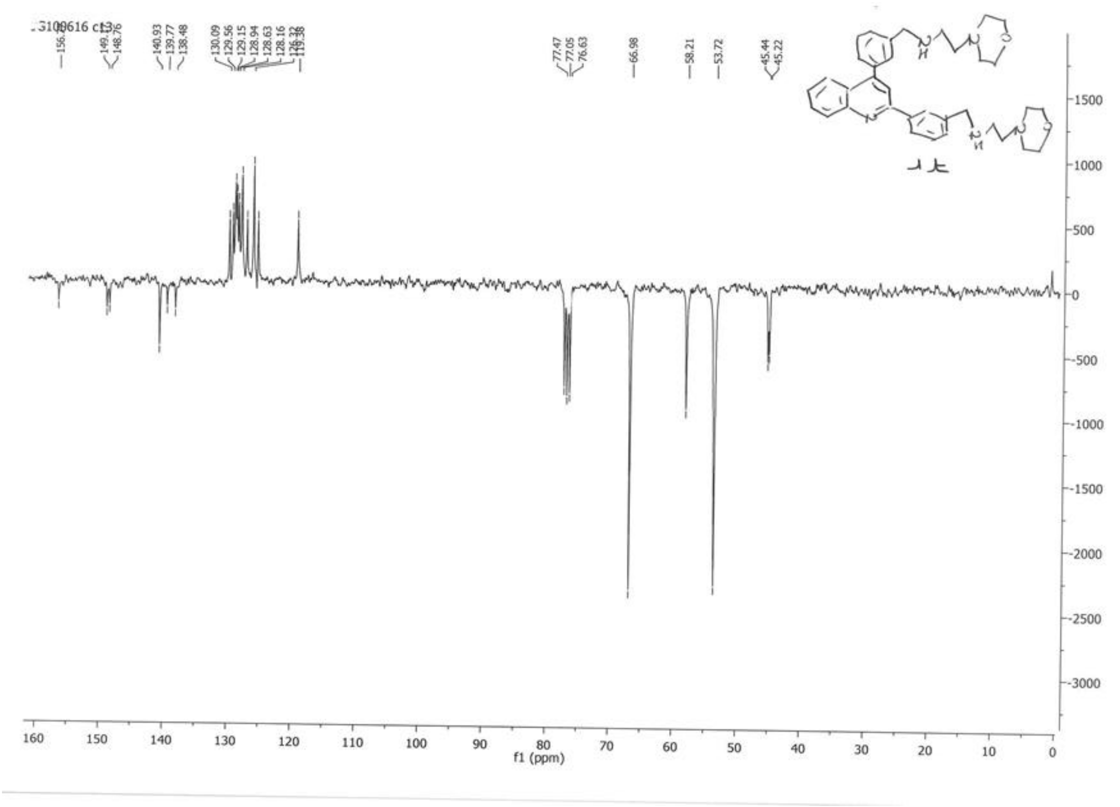

**Fig.S55.** $^{13}$C NMR spectrum of **1t**

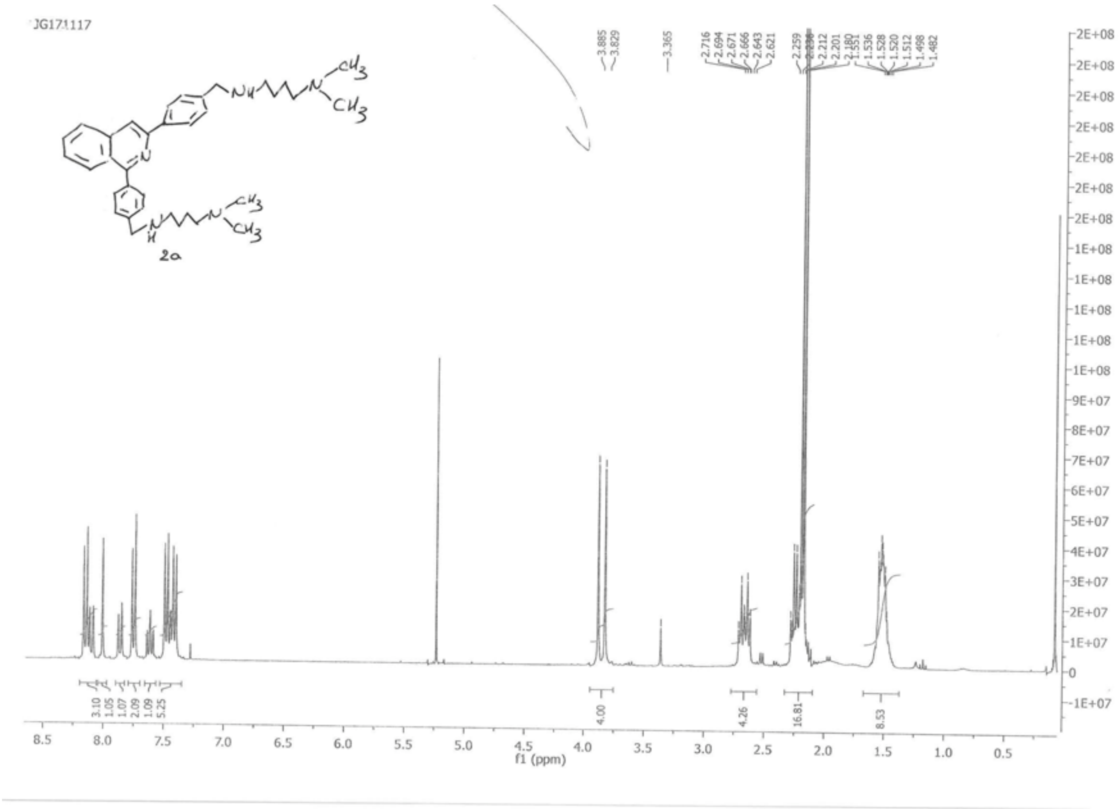

**Fig.S56.** $^{1}$H NMR spectrum of **2a**

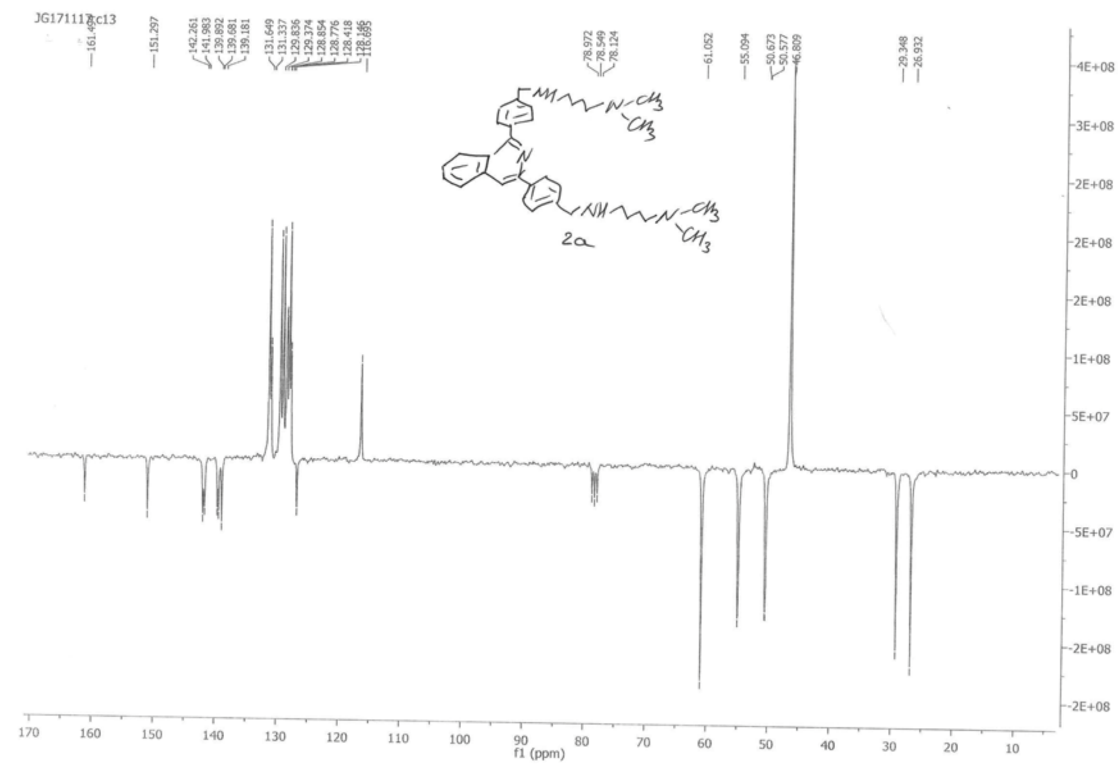

**Fig.S57.** $^{13}$C NMR spectrum of **2a**

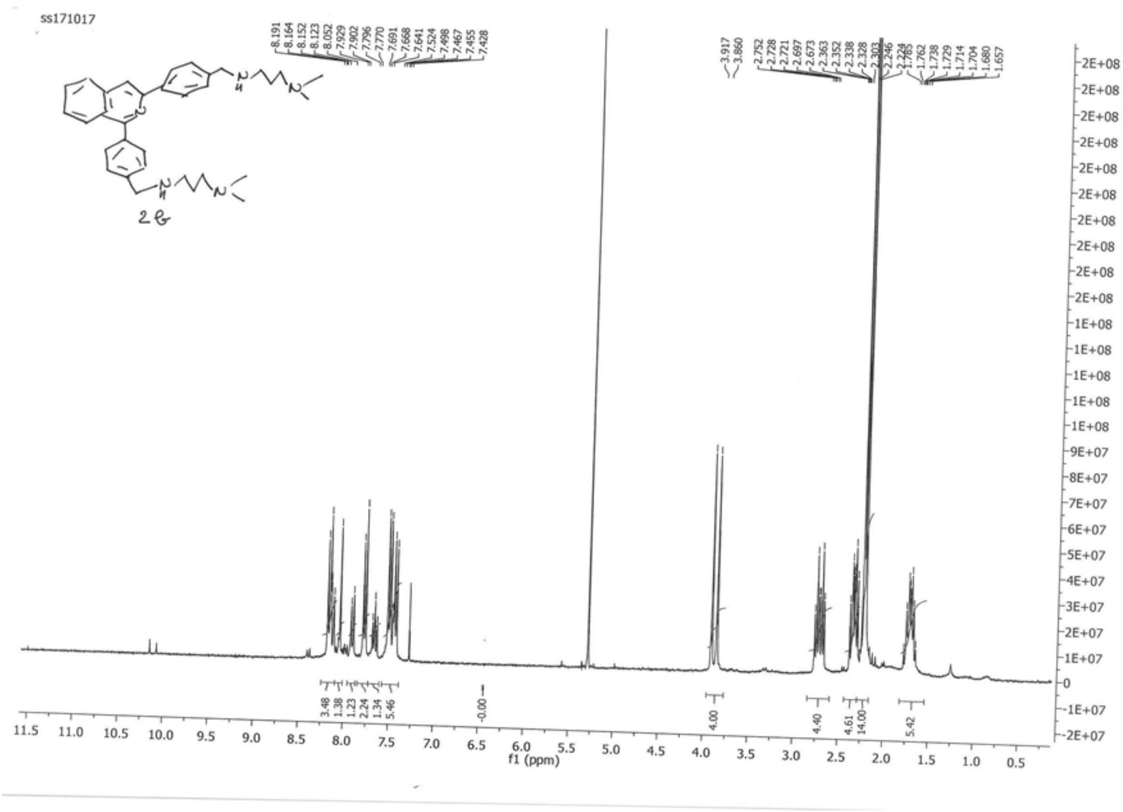

**Fig.S58.** $^{1}$H NMR spectrum of **2b**

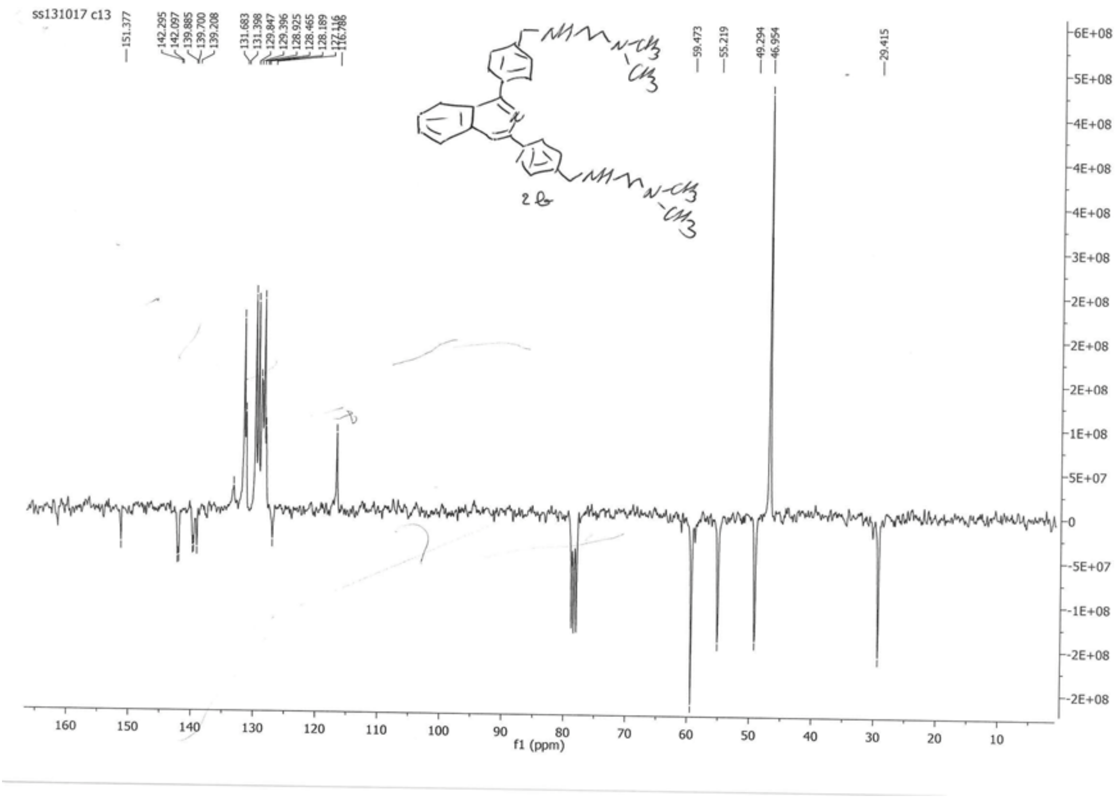

**Fig.S59.** $^{13}$C NMR spectrum of **2b**

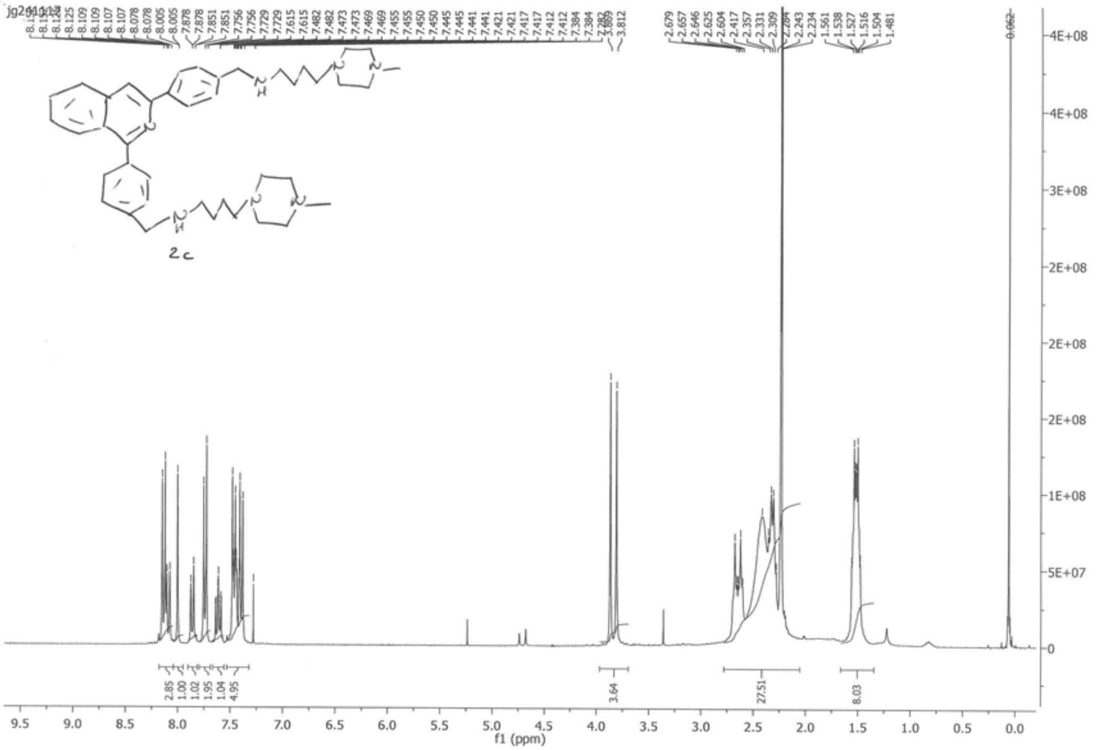

**Fig.S60.** $^{1}$H NMR spectrum of **2c**

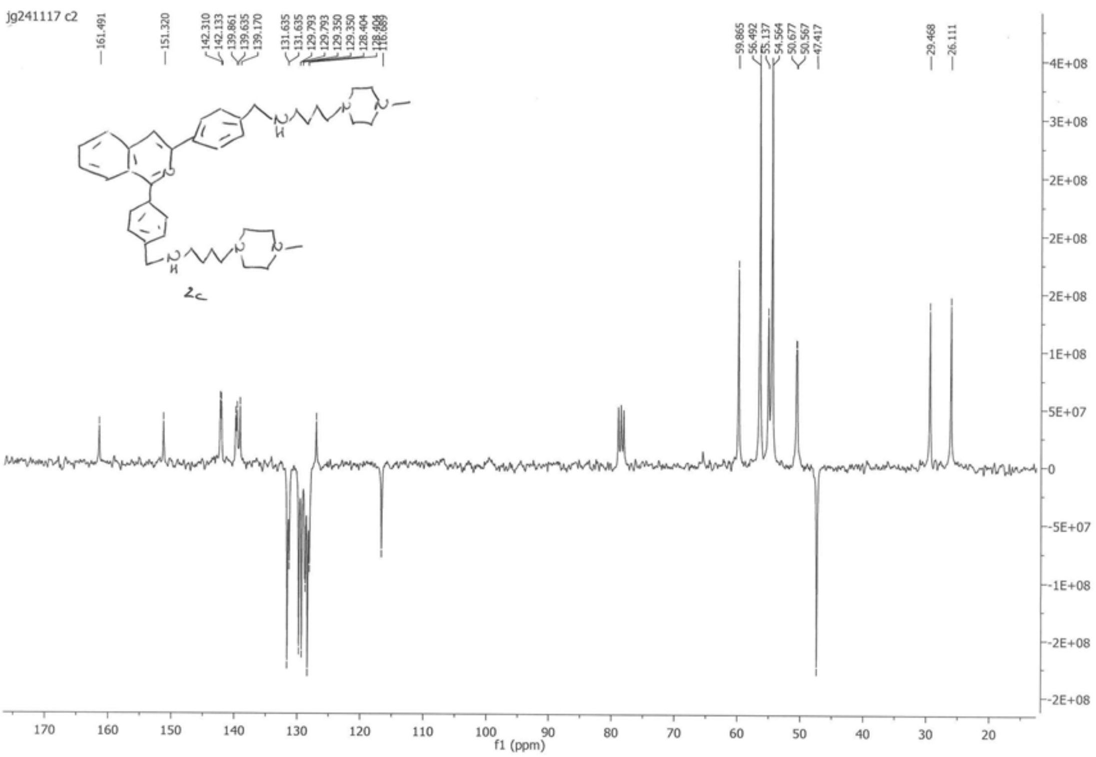

**Fig.S61.** $^{13}$C NMR spectrum of **2c**

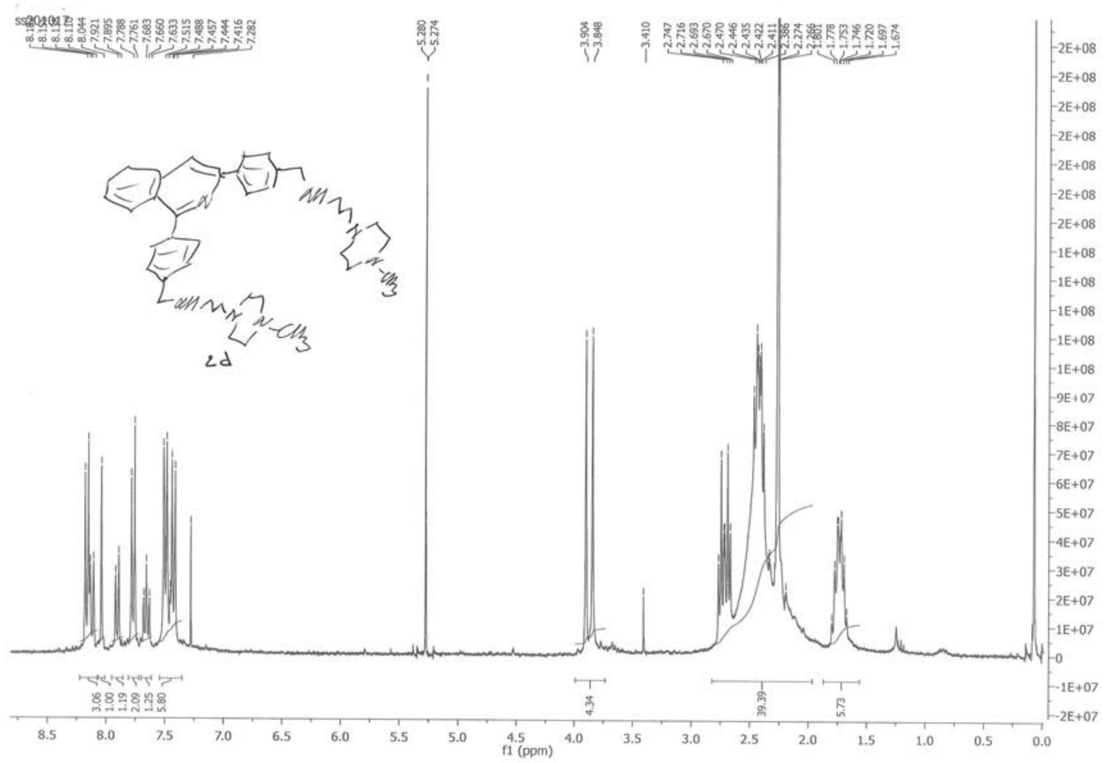

**Fig.S62.** $^{1}$H NMR spectrum of **2d**

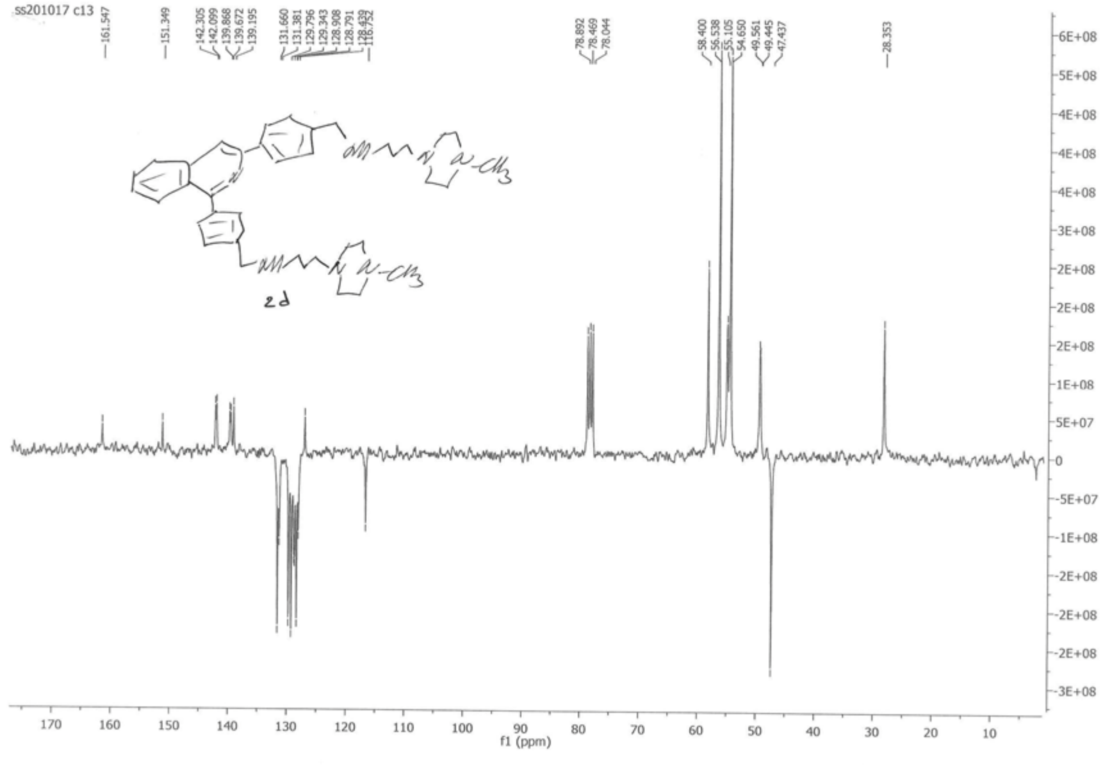

**Fig.S63.** $^{13}$C NMR spectrum of **2d**

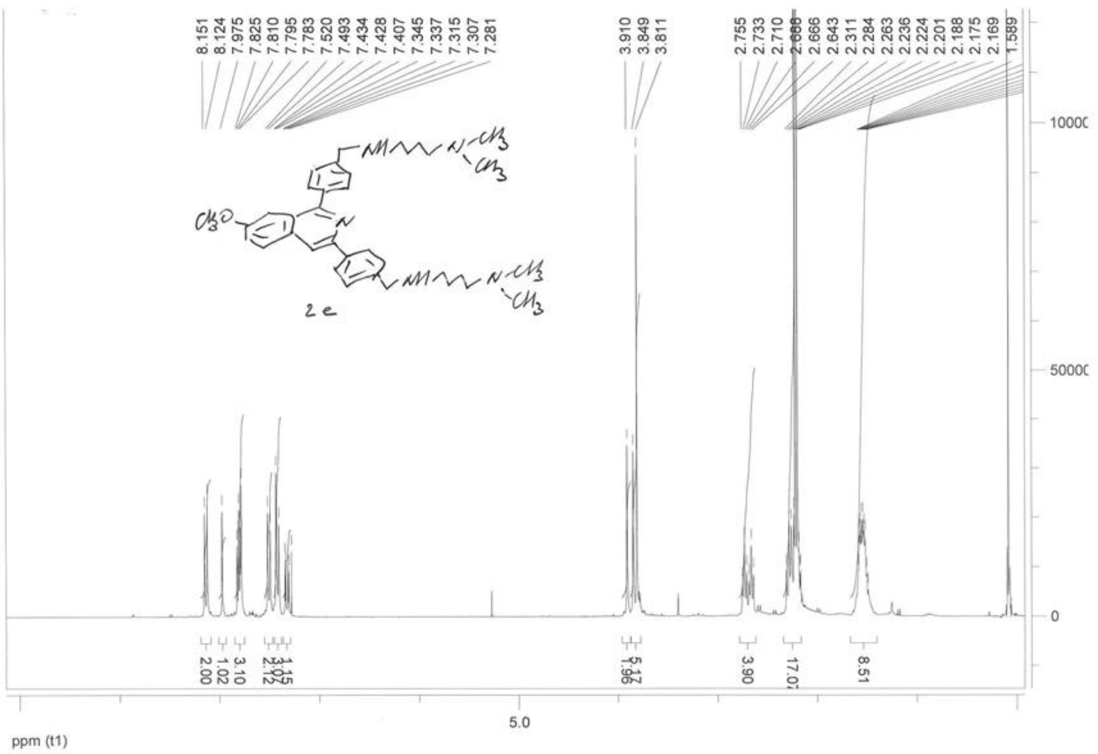


**Fig.S64.** $^{1}$H NMR spectrum of **2e**

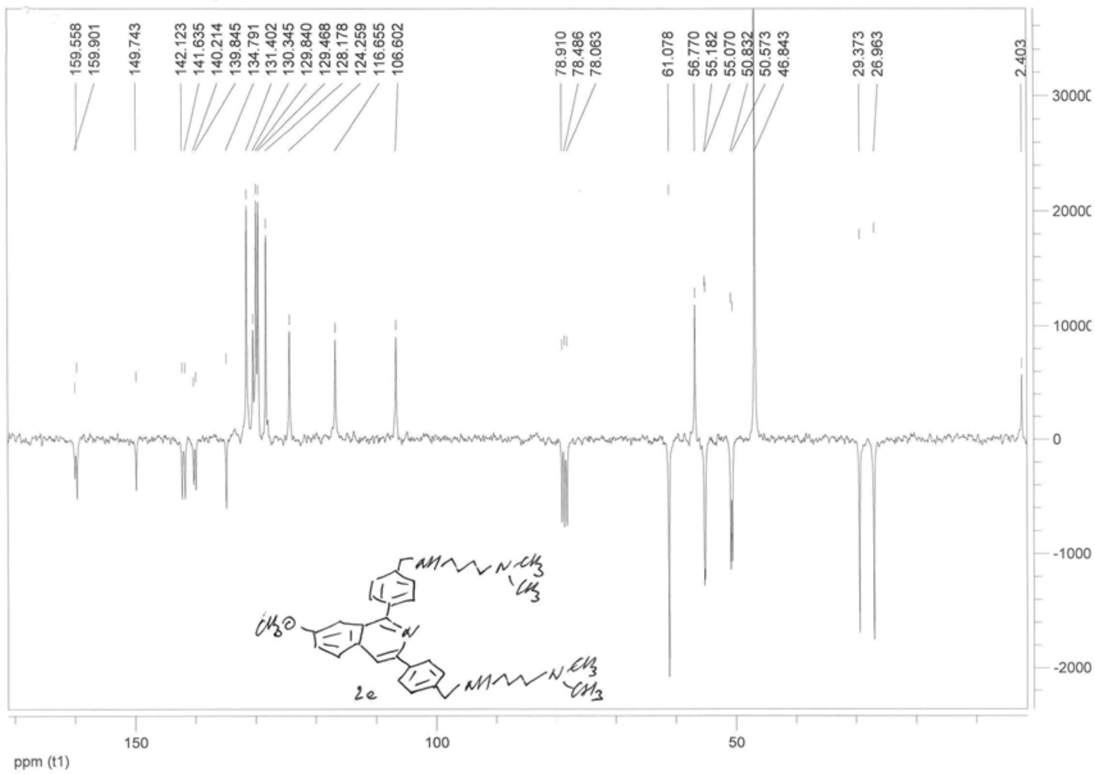


**Fig.S65.** $^{13}$C NMR spectrum of **2e**

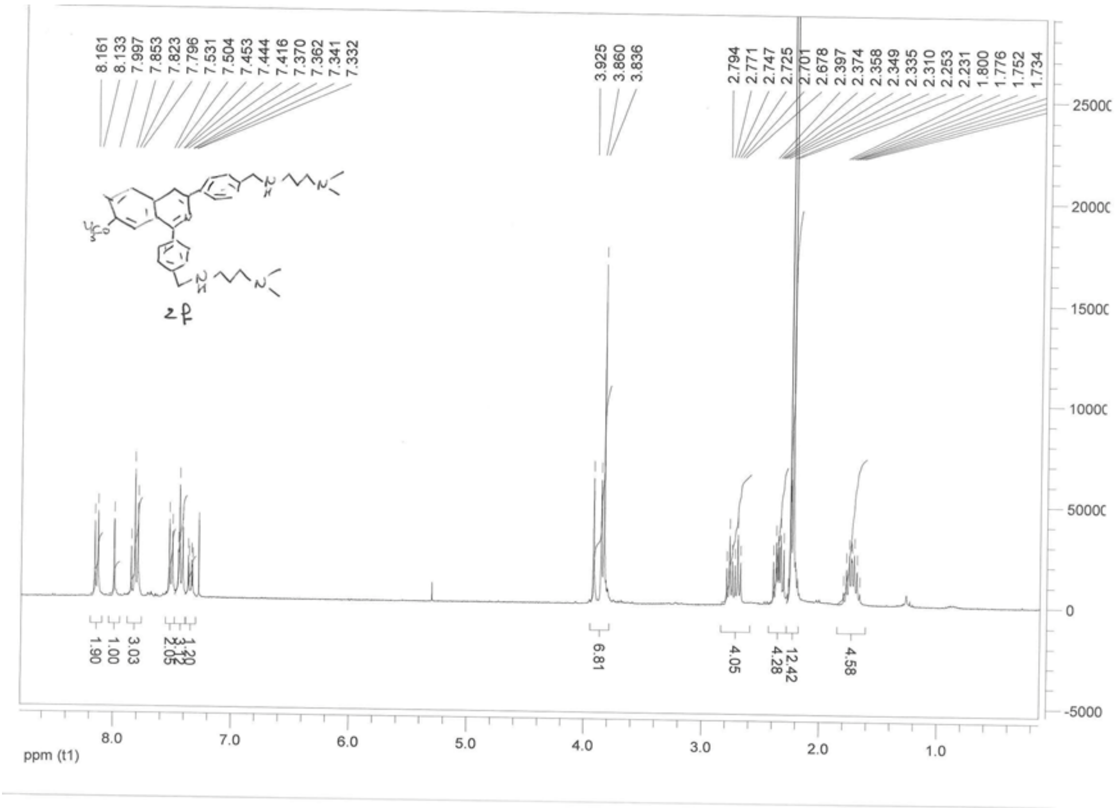

**Fig.S66.** $^{1}$H NMR spectrum of **2f**

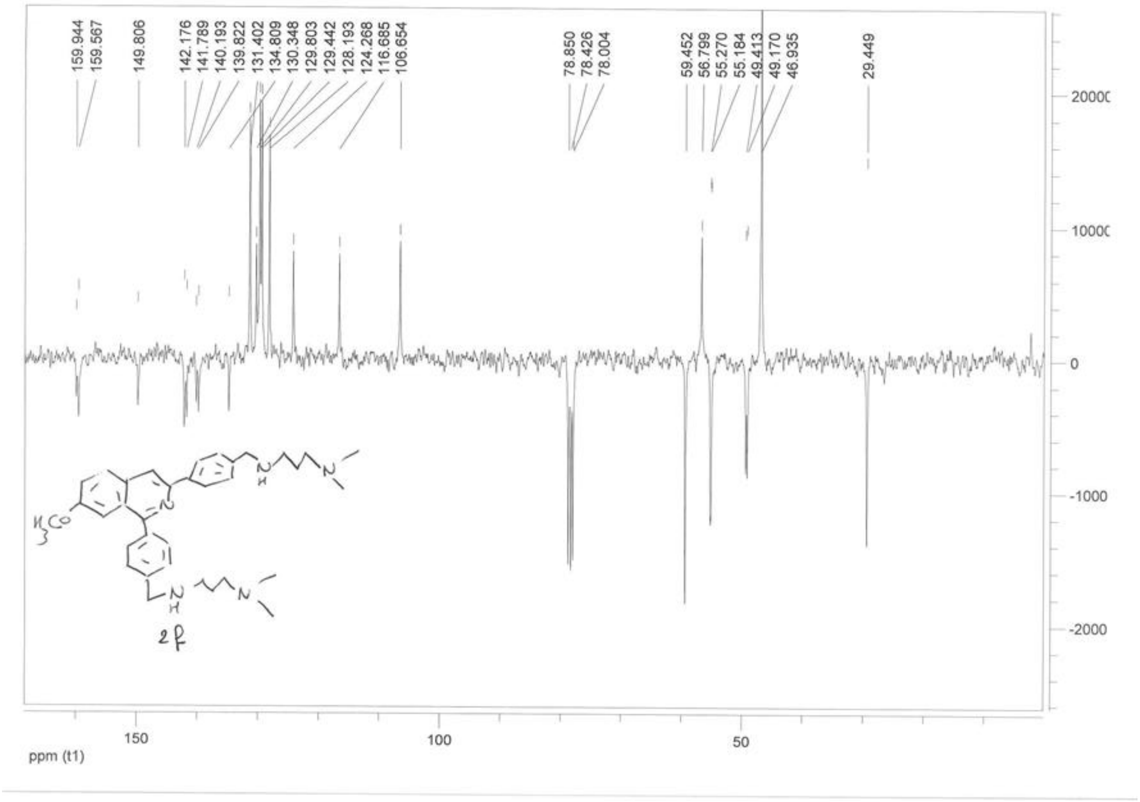

**Fig.S67.** $^{13}$C NMR spectrum of **2f**

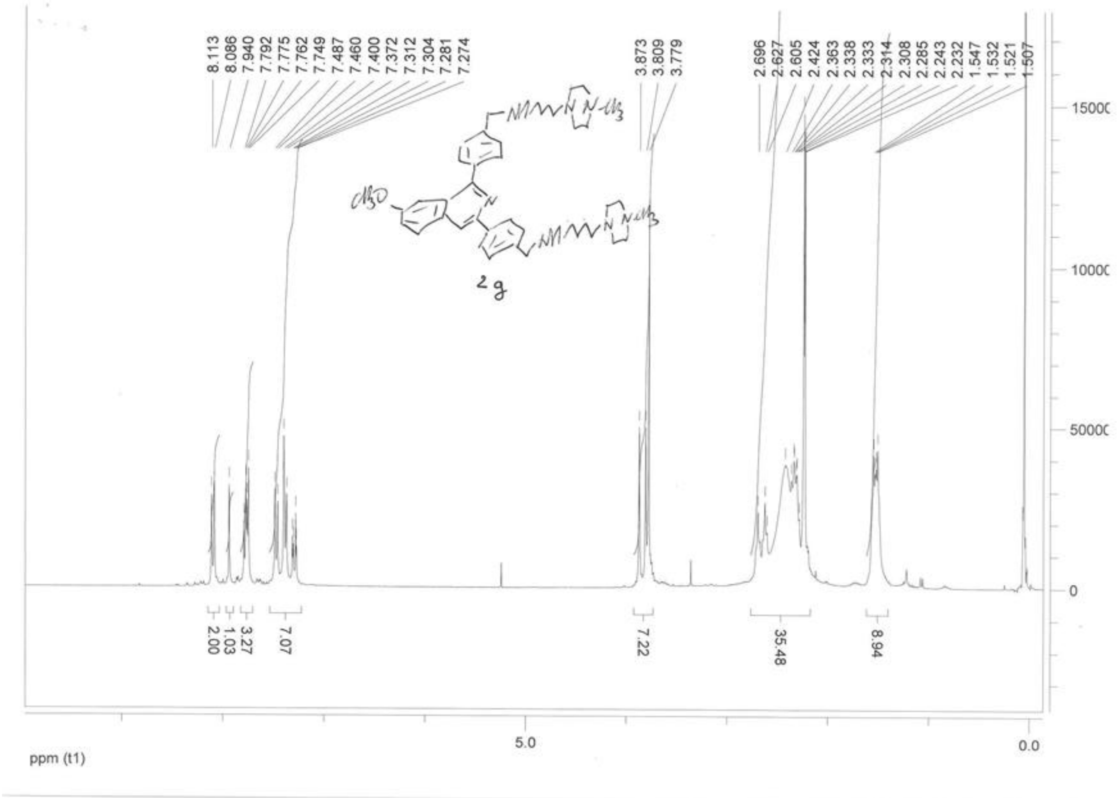

**Fig.S68.** $^{1}$H NMR spectrum of **2g**

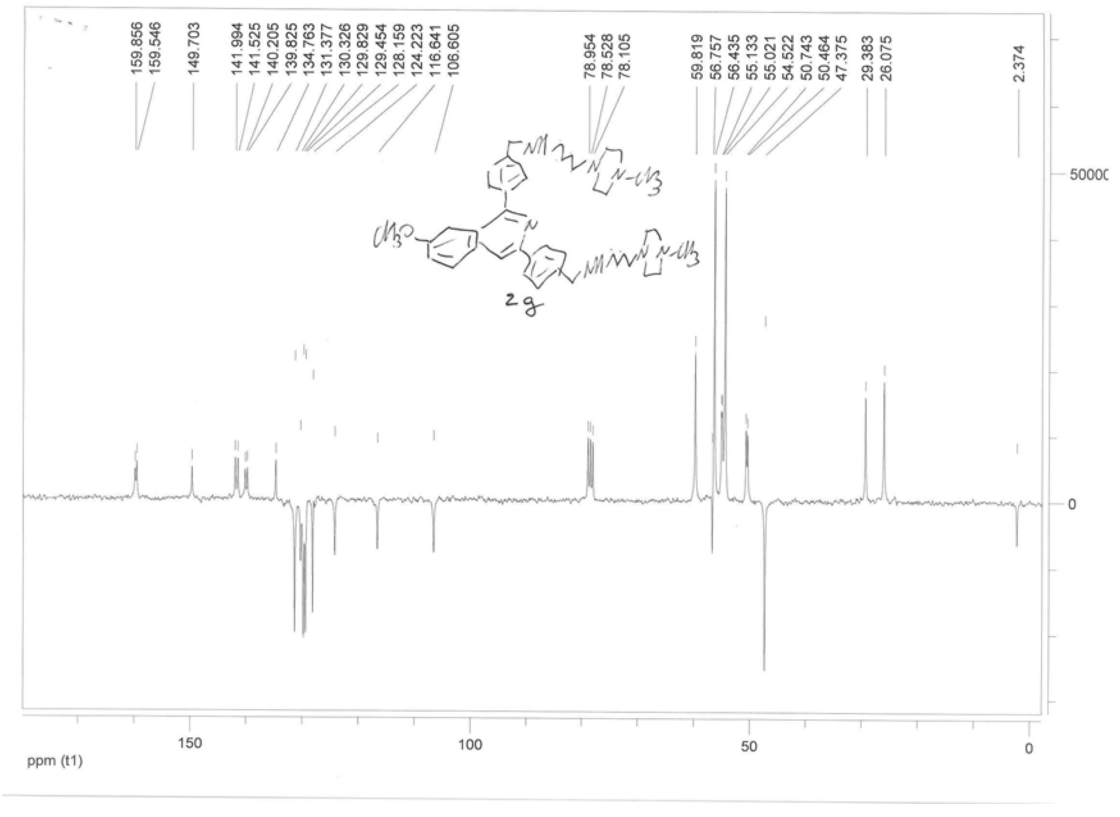

**Fig.S69.** $^{13}$C NMR spectrum of **2g**

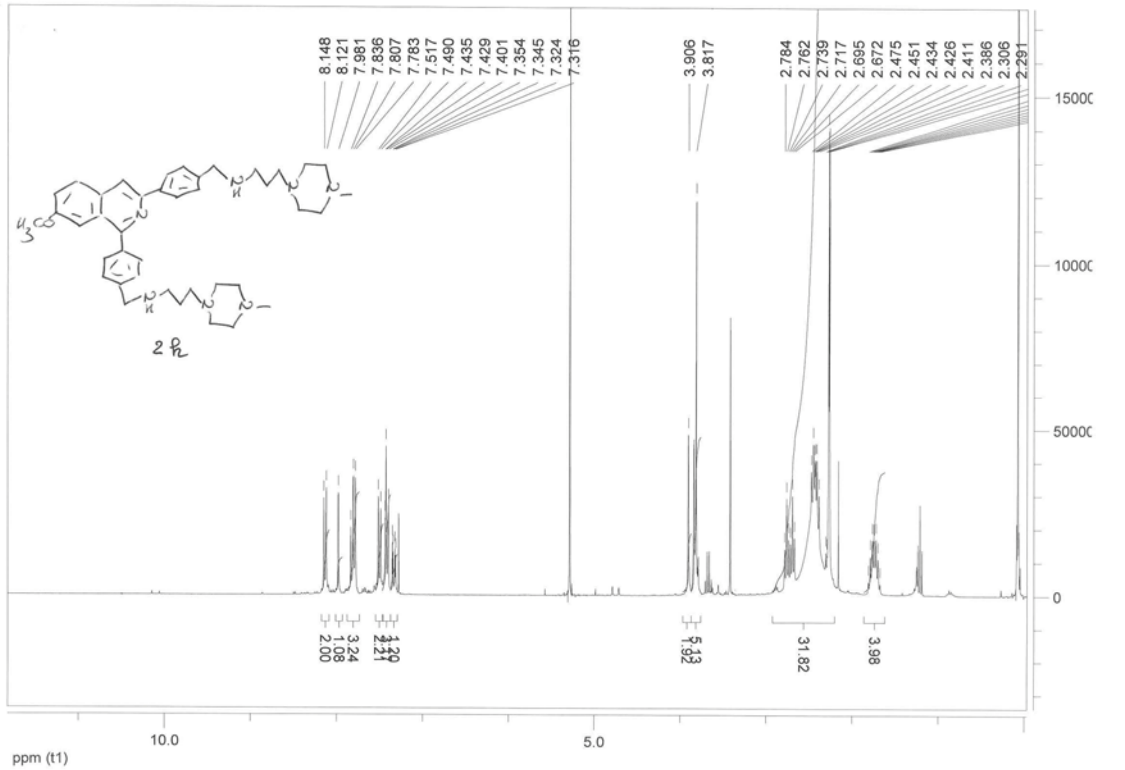

**Fig.S70.** $^{1}$H NMR spectrum of **2h**

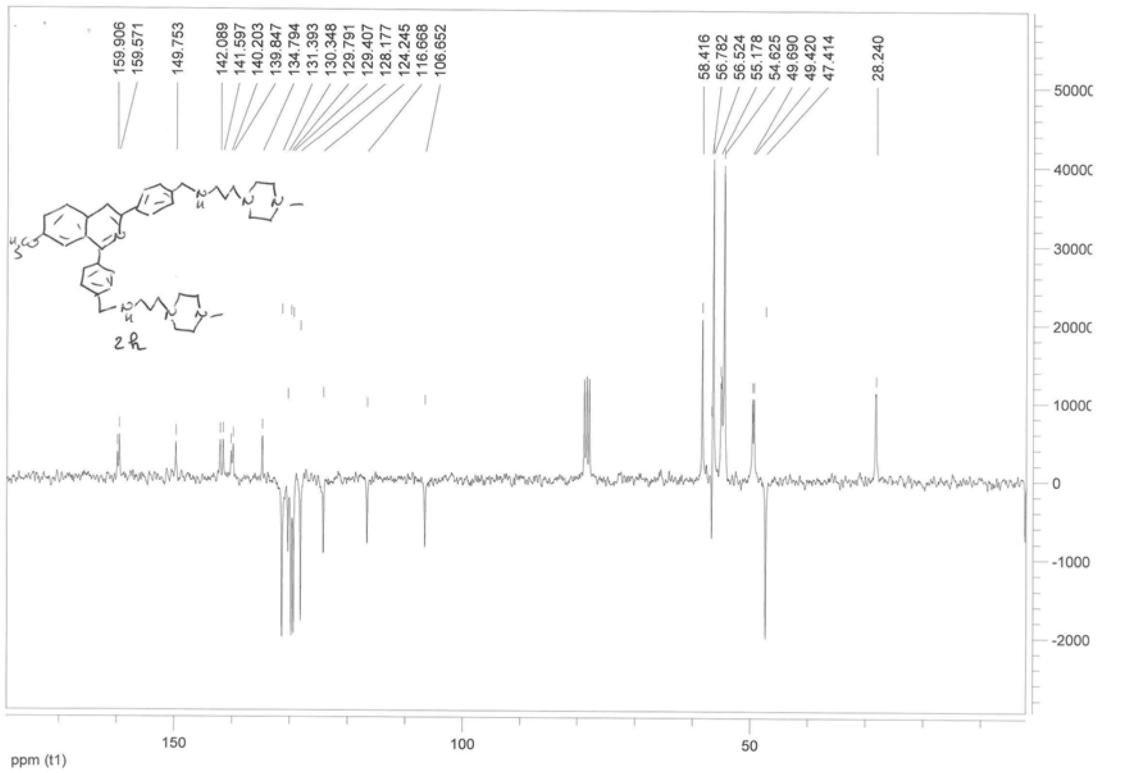

**Fig.S71.** $^{13}$C NMR spectrum of **2h**

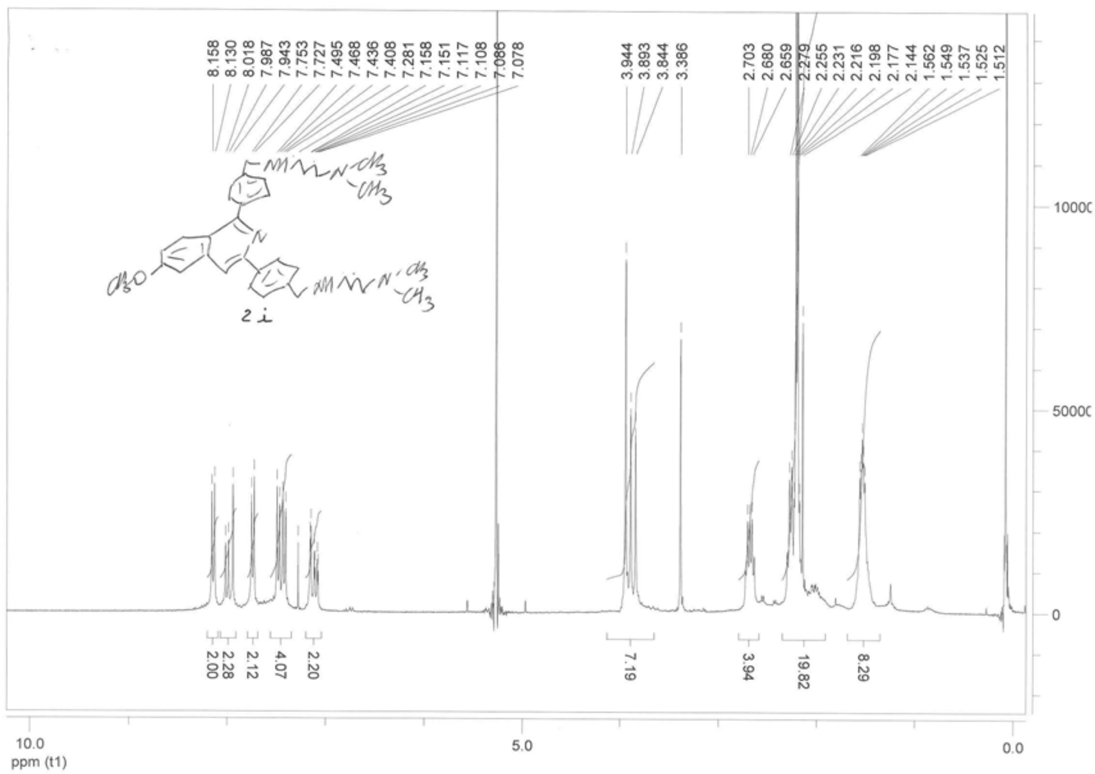


**Fig.S72.** $^{1}$H NMR spectrum of **2i**

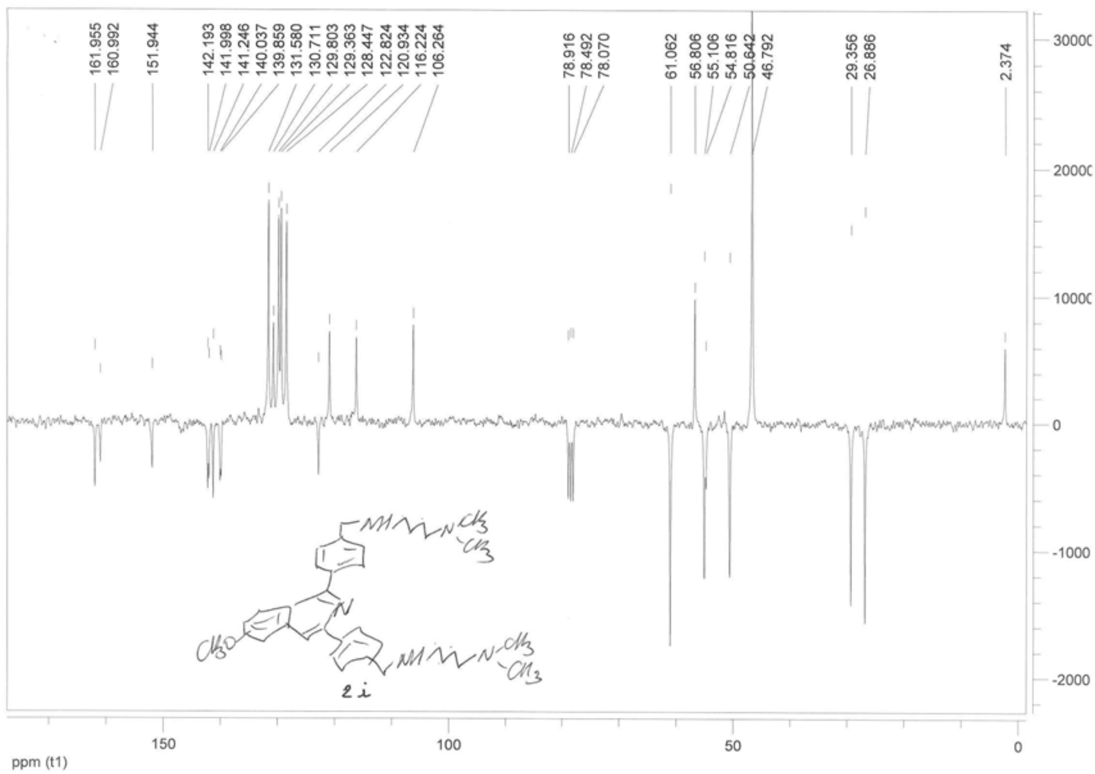


**Fig.S73.** $^{13}$C NMR spectrum of **2i**

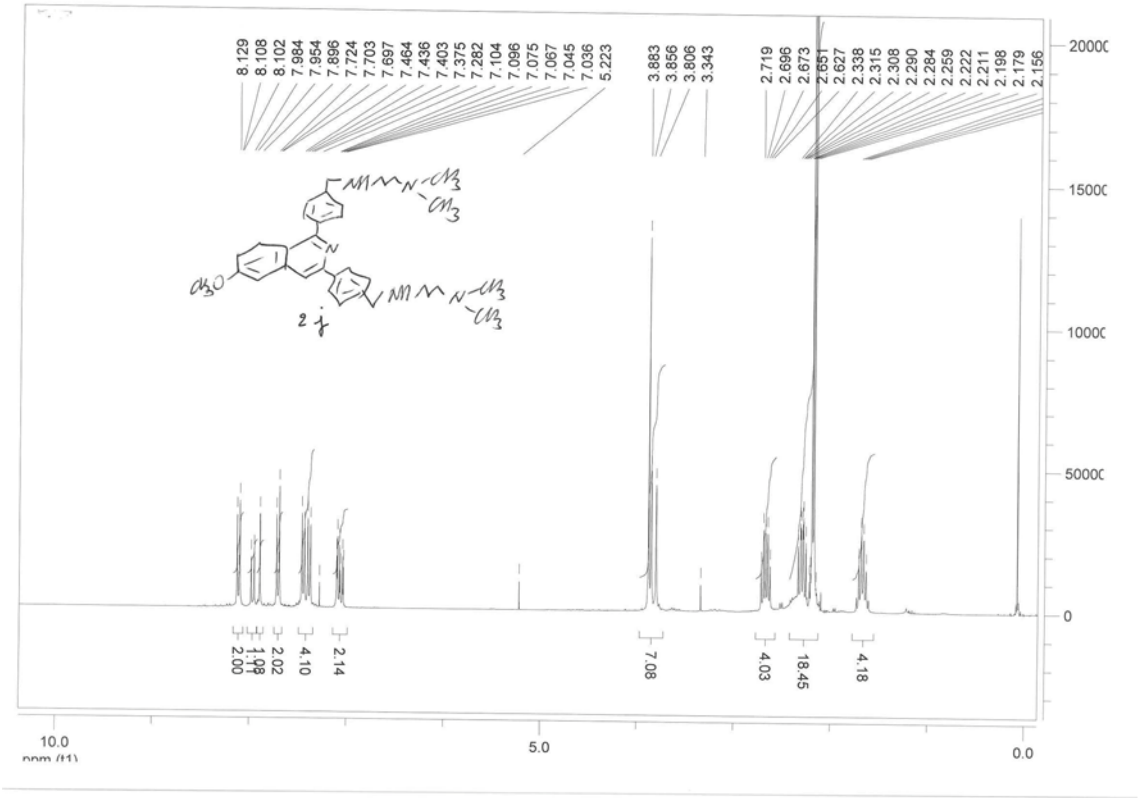

**Fig.S74.** $^{1}$H NMR spectrum of **2j**

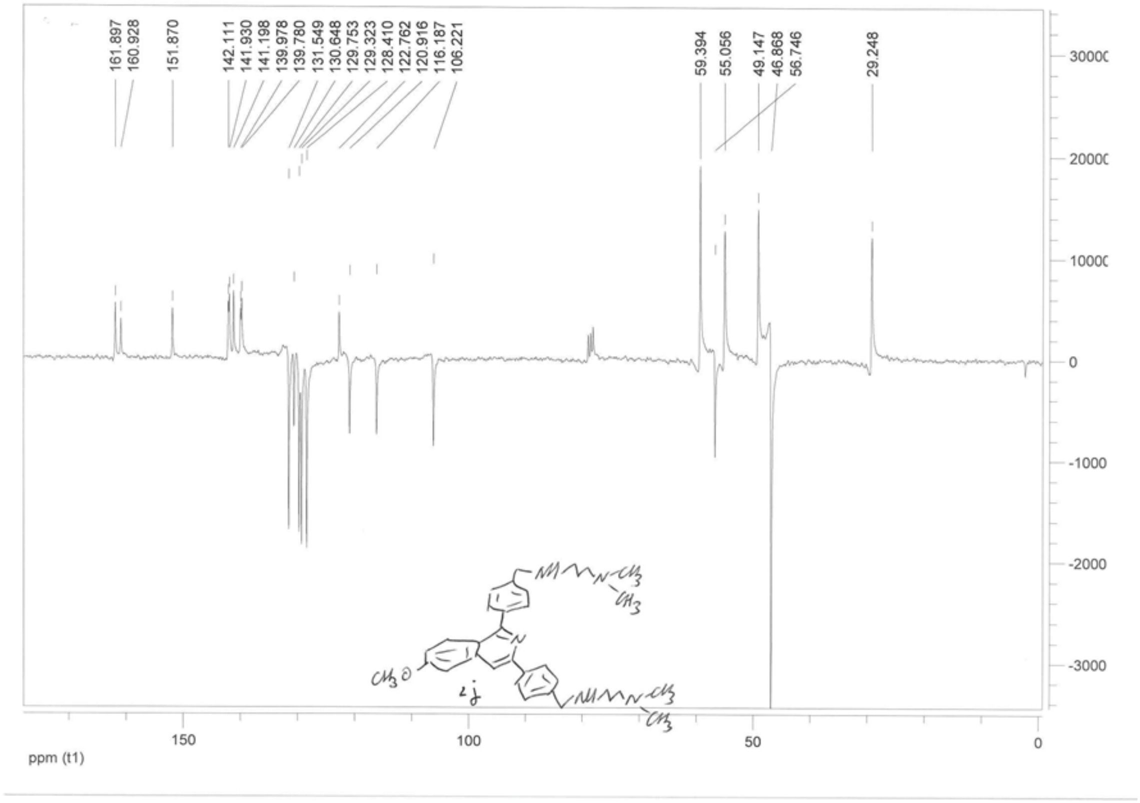

**Fig.S75.** $^{13}$C NMR spectrum of **2j**

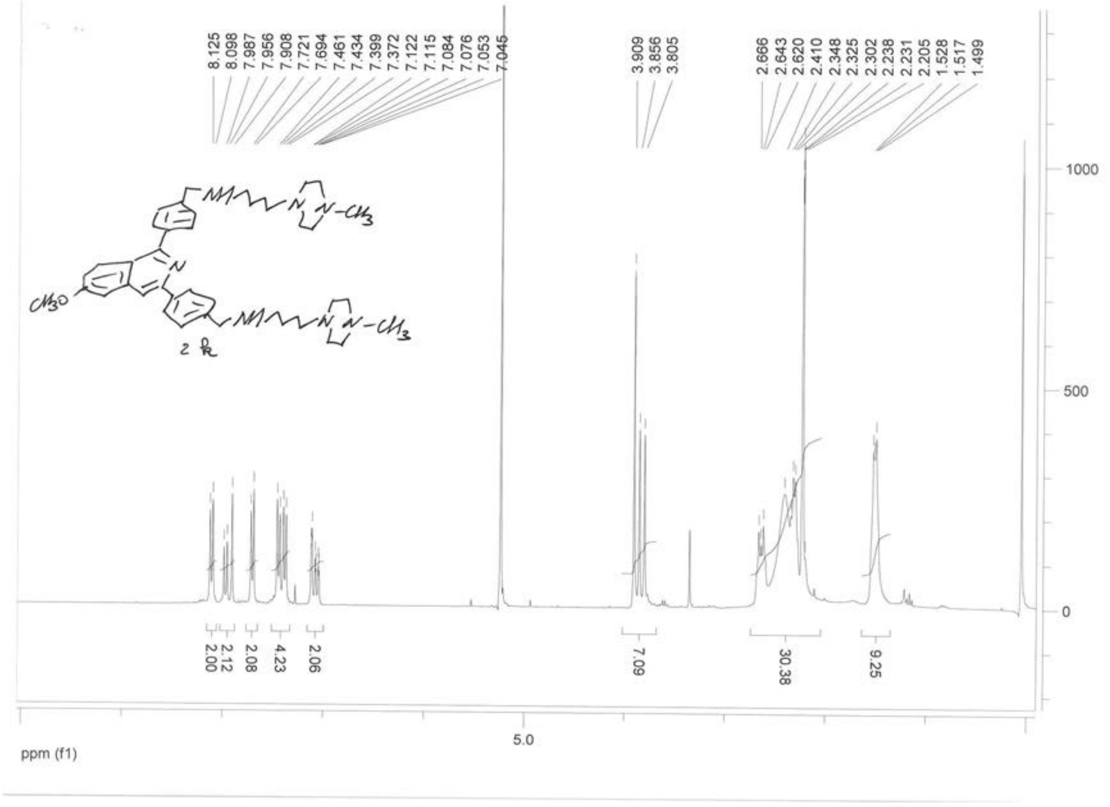


**Fig.S76.** $^{1}$H NMR spectrum of **2k**

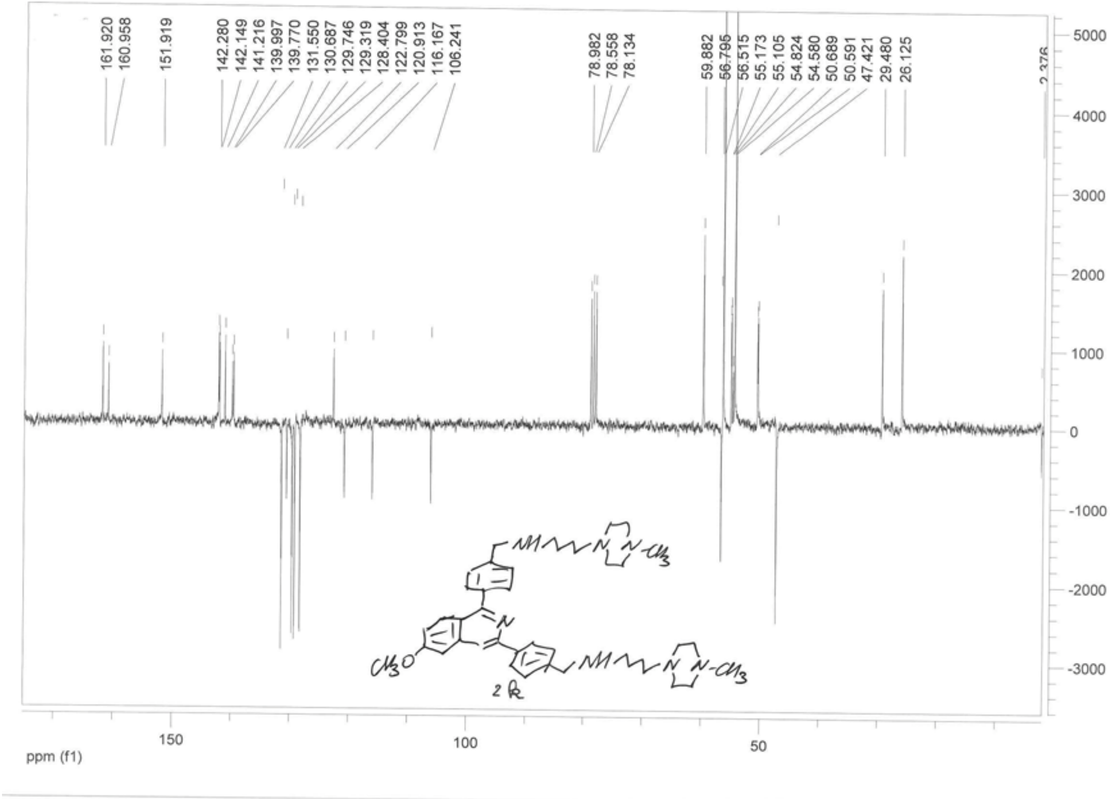


**Fig.S77.** $^{13}$C NMR spectrum of **2k**

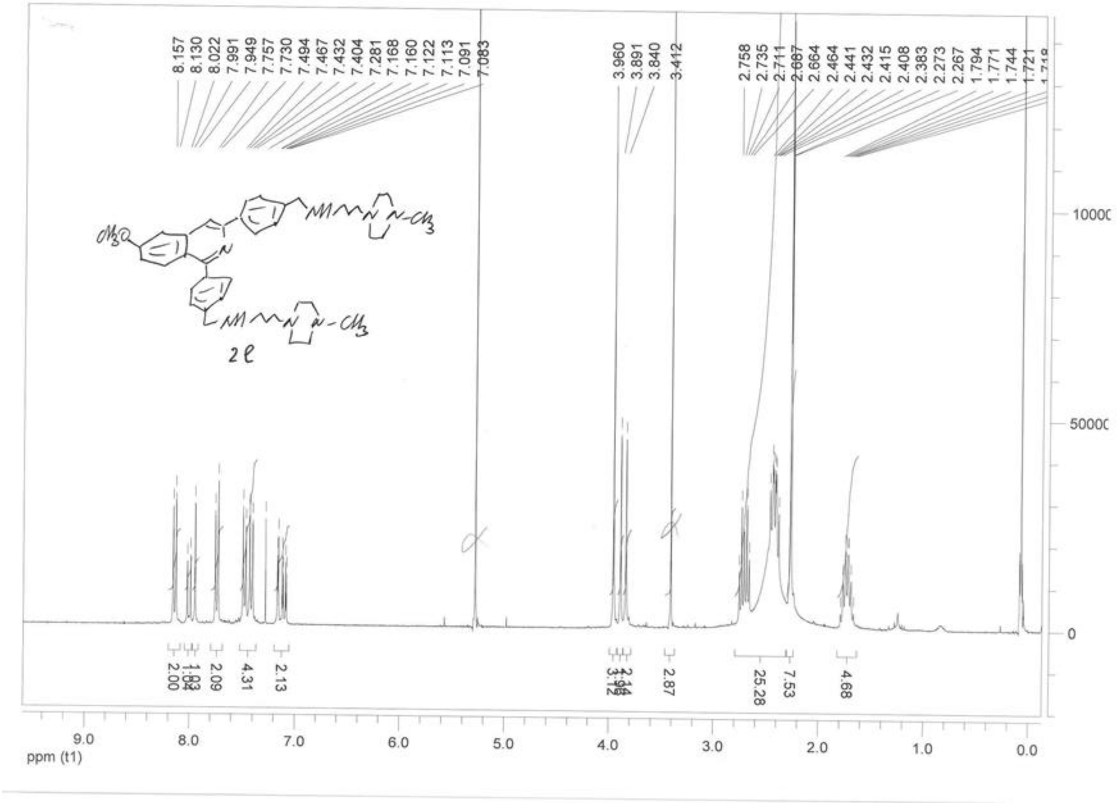

**Fig.S78.** $^{1}$H NMR spectrum of **2l**

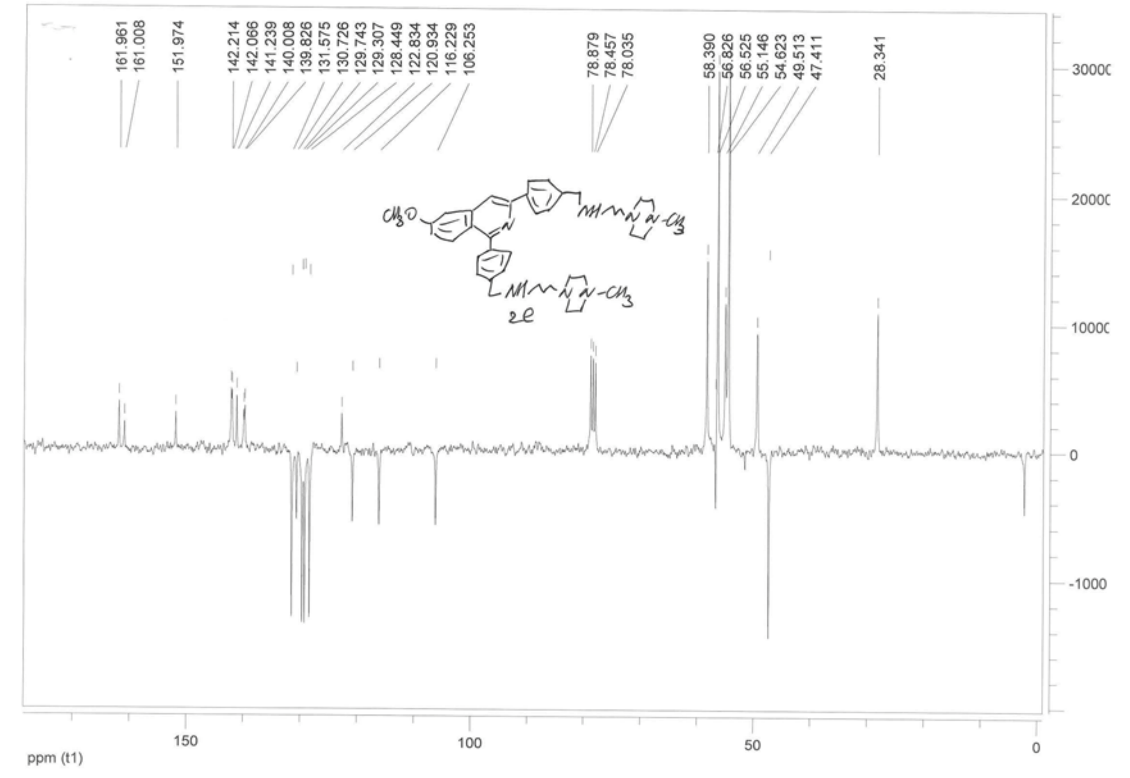

**Fig.S79.** $^{13}$C NMR spectrum of **2l**

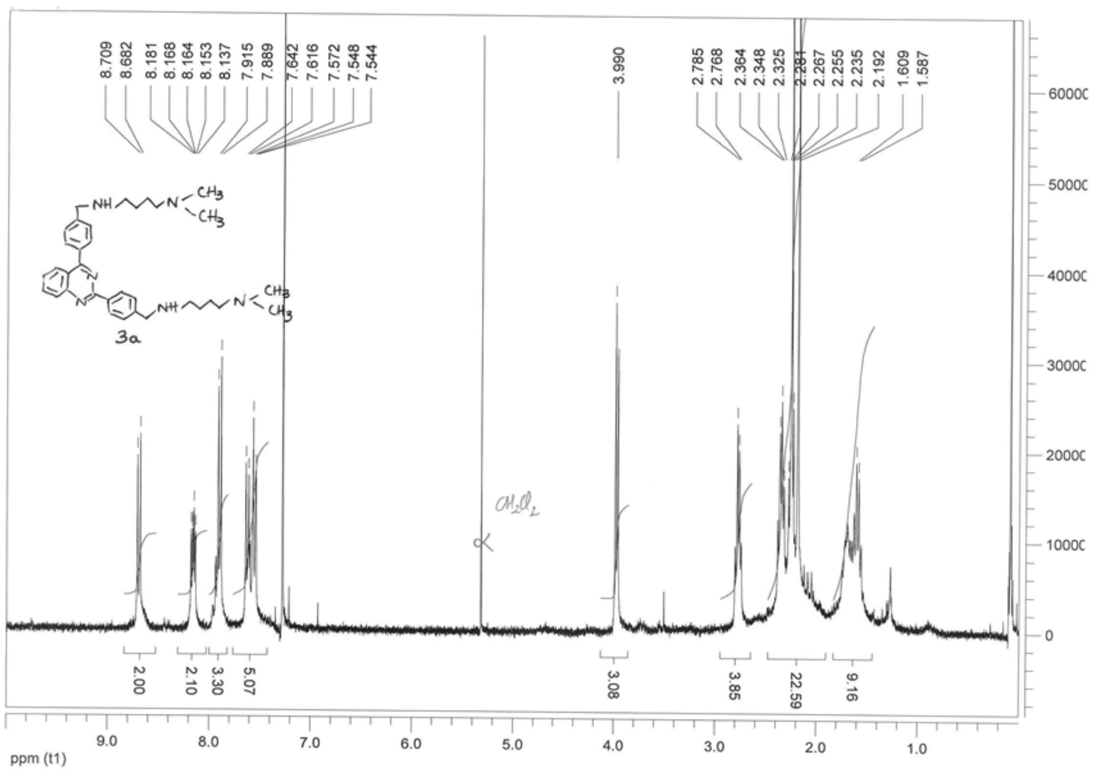


**Fig.S80**. $^{1}$H NMR spectrum of **3a**

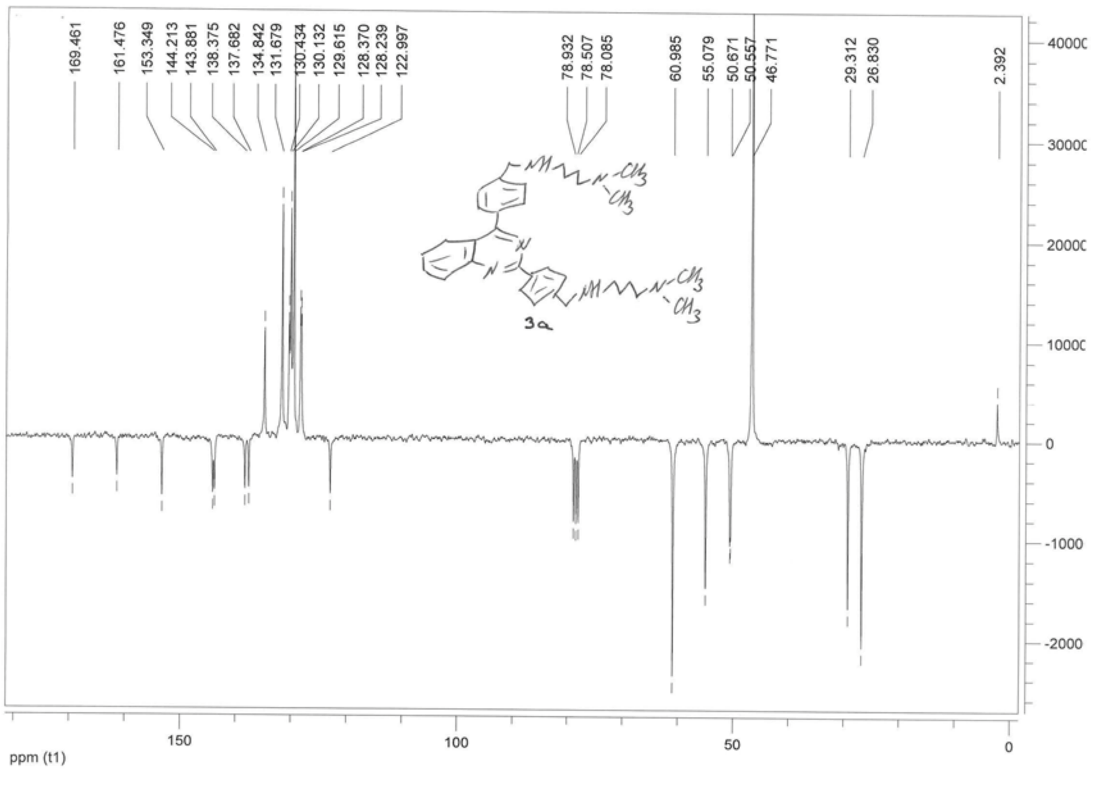


**Fig.S81.** $^{13}$C NMR spectrum of **3a**

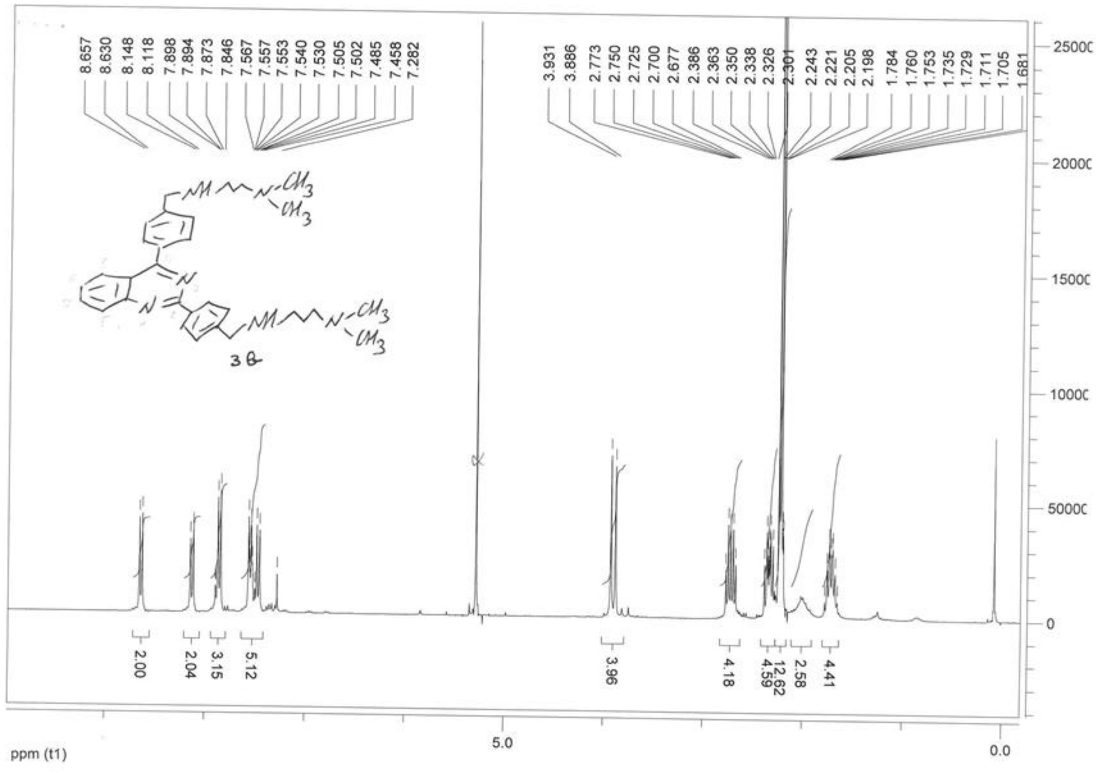

**Fig.S82**. $^1$H NMR spectrum of **3b**

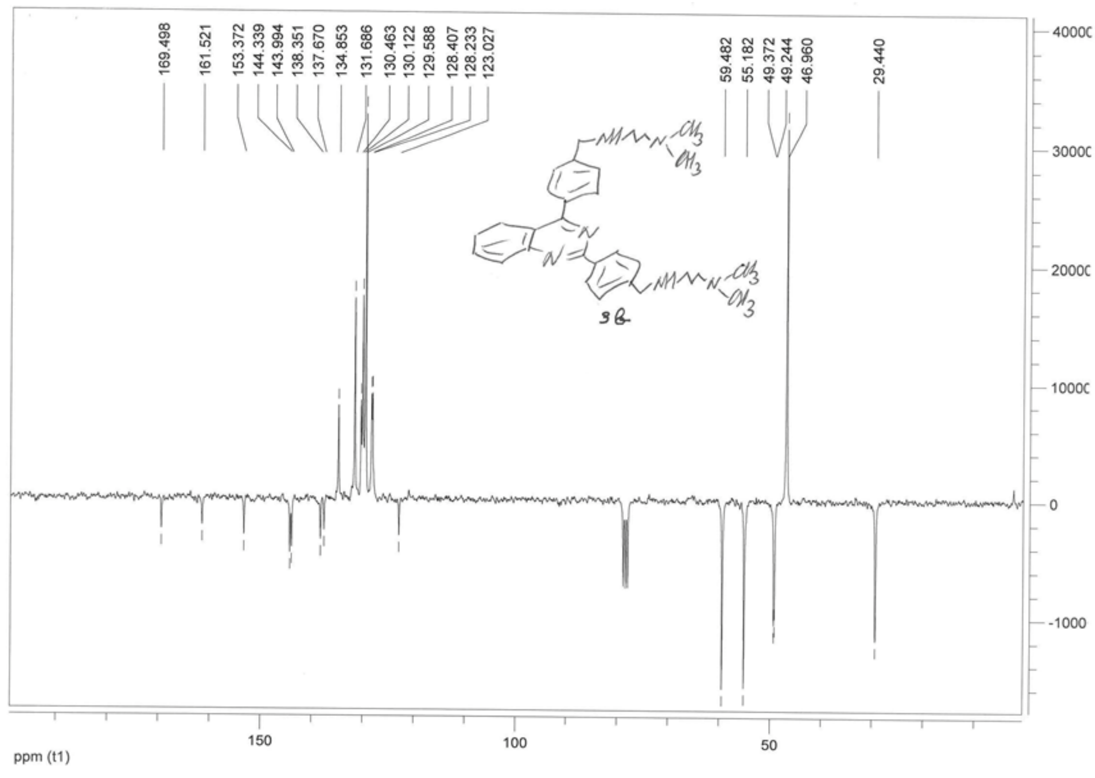

**Fig.S83.** $^{13}$C NMR spectrum of **3b**

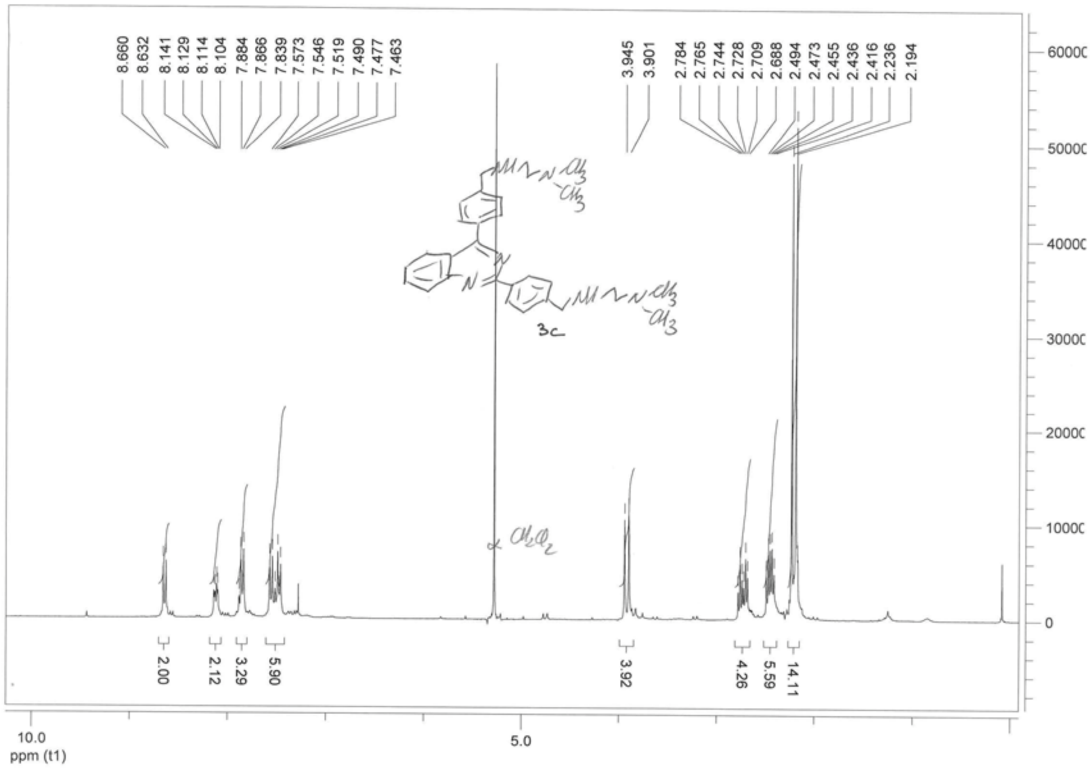

**Fig.S84.** $^{1}$H NMR spectrum of **3c**

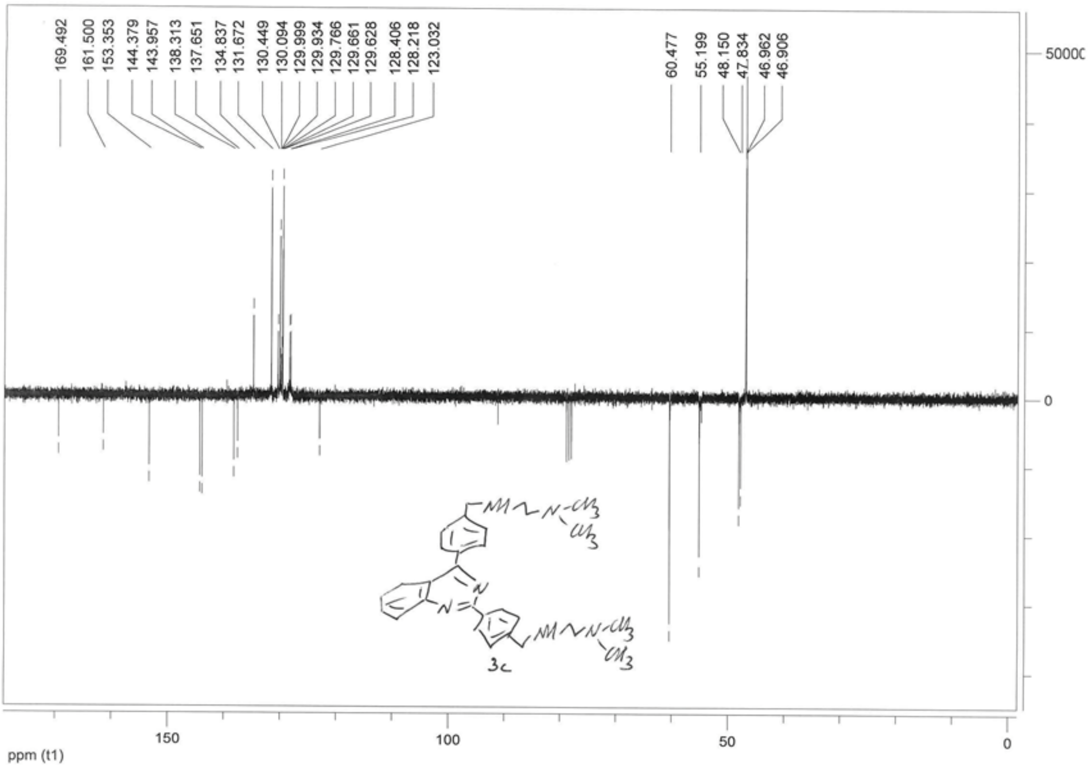

**Fig.S85.** $^{13}$C NMR spectrum of **3c**

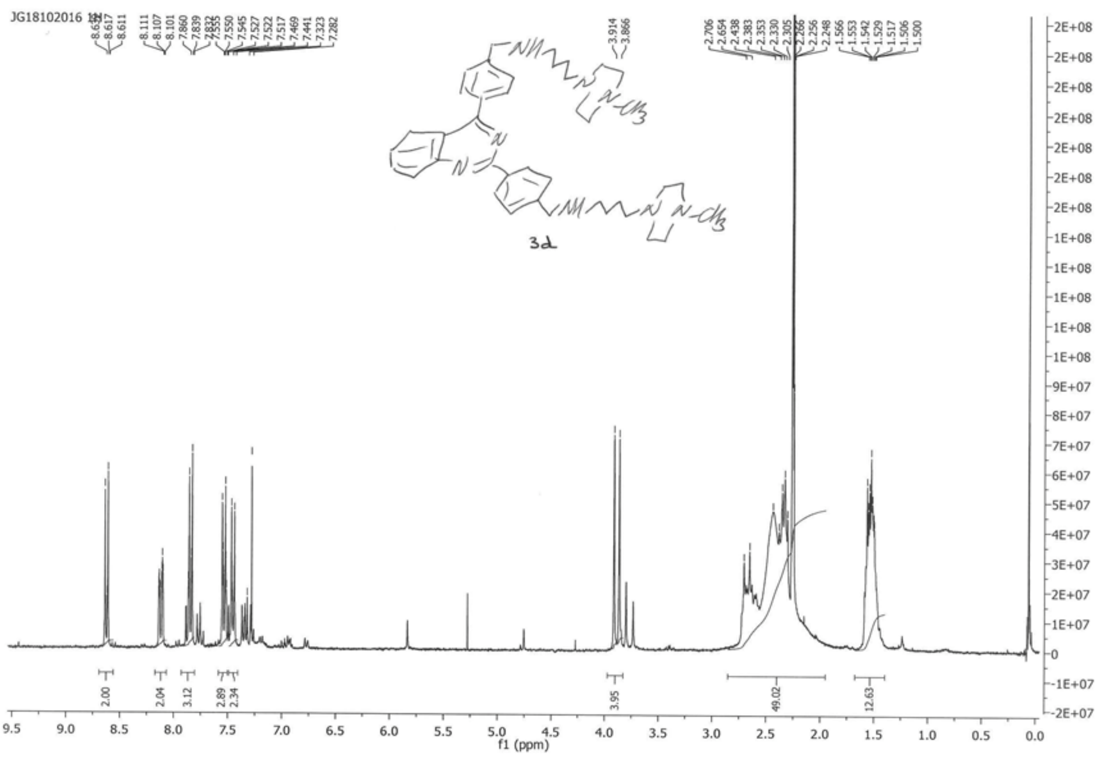

**Fig.S85.** $^{1}$H NMR spectrum of **3d**

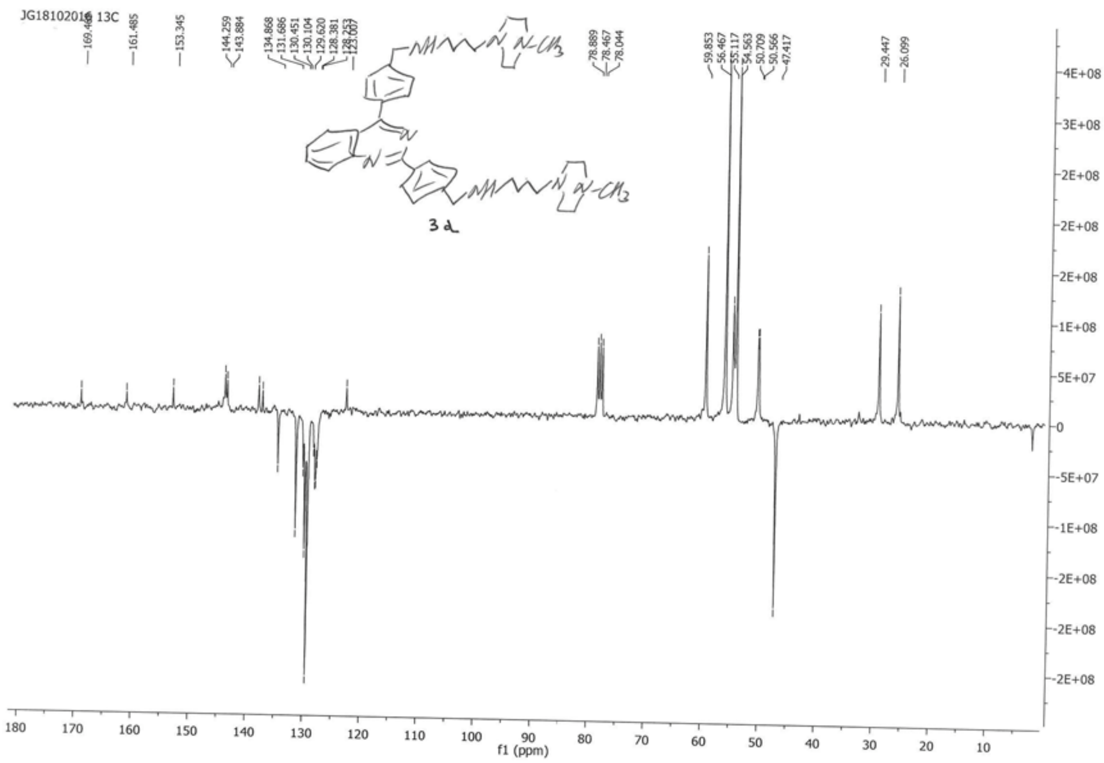

**Fig.S86.** $^{13}$C NMR spectrum of **3d**

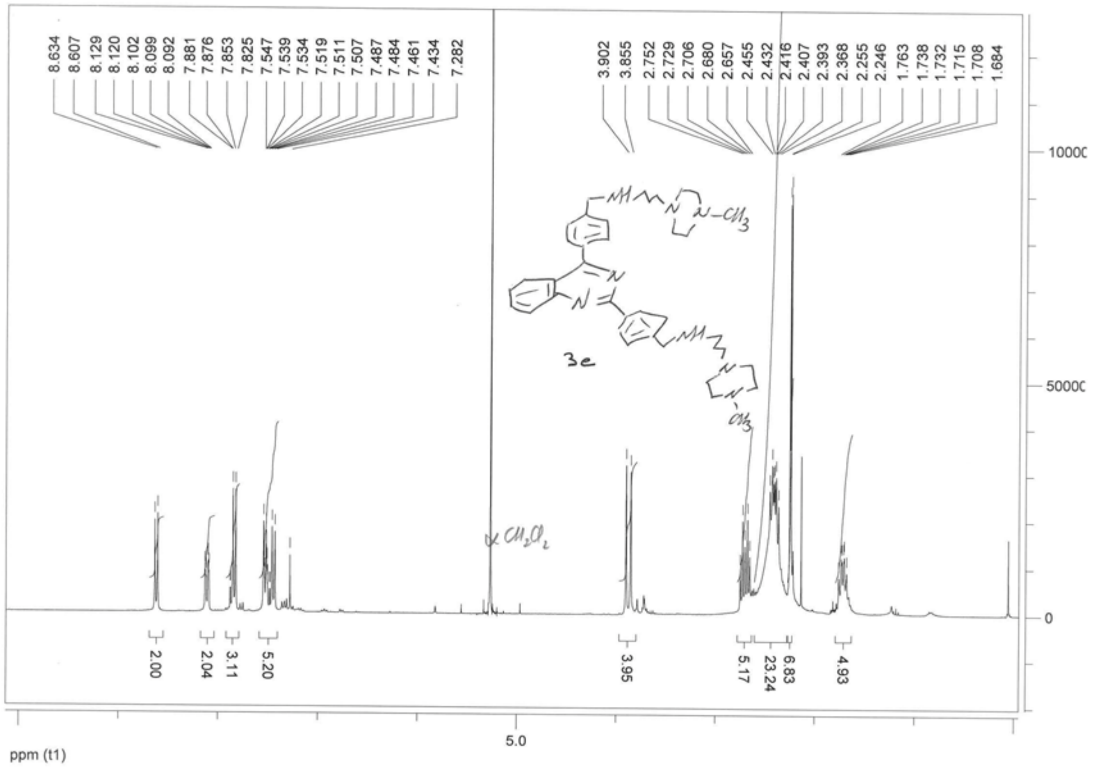

**Fig.S87**. $^{1}$H NMR spectrum of **3e**

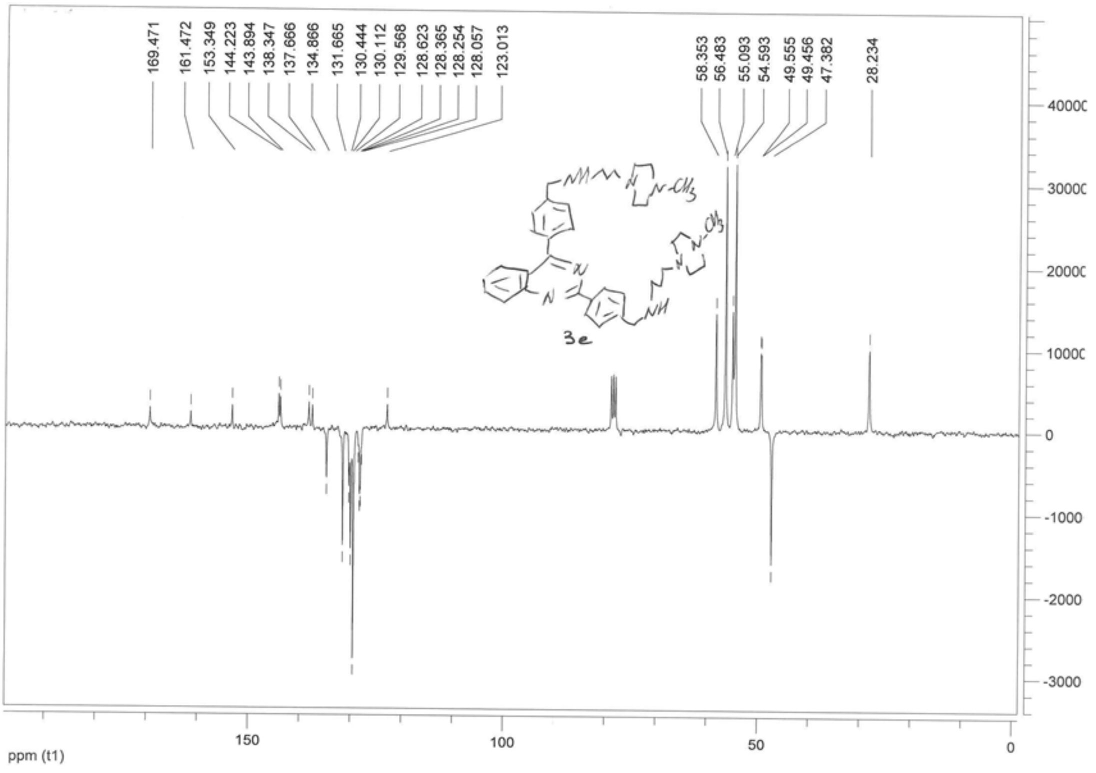

**Fig.S88.** $^{13}$C NMR spectrum of **3e**

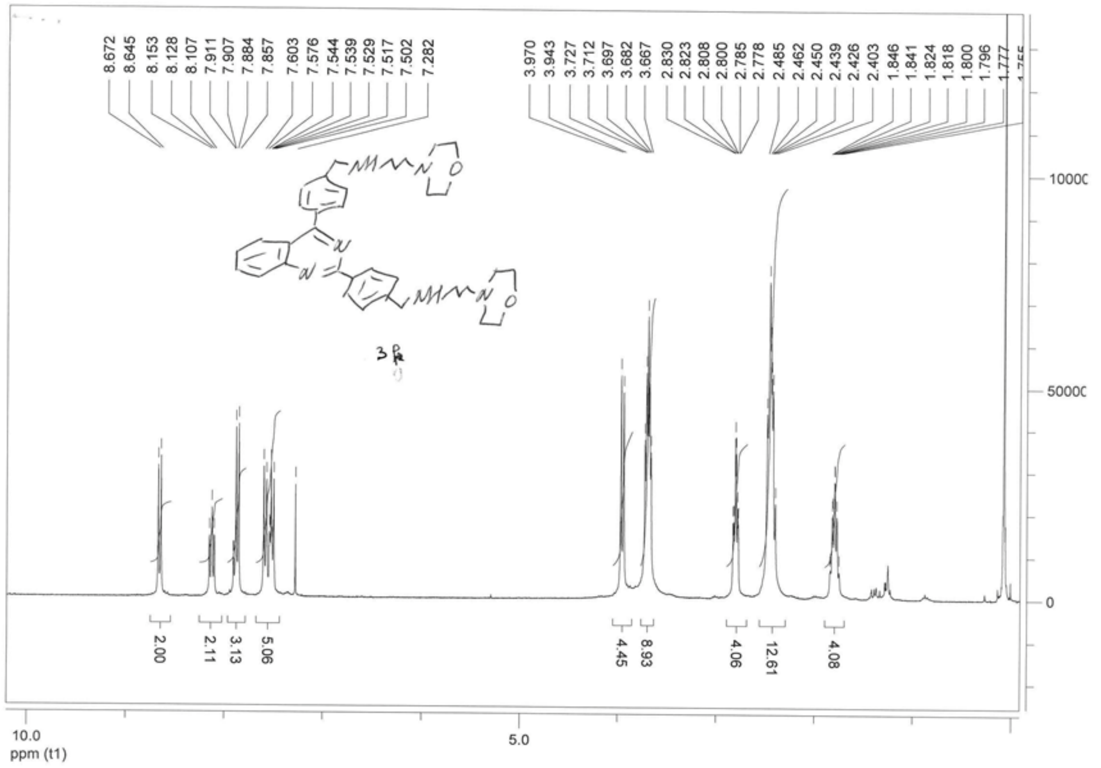


**Fig.S89.** $^{1}$H NMR spectrum of **3f**

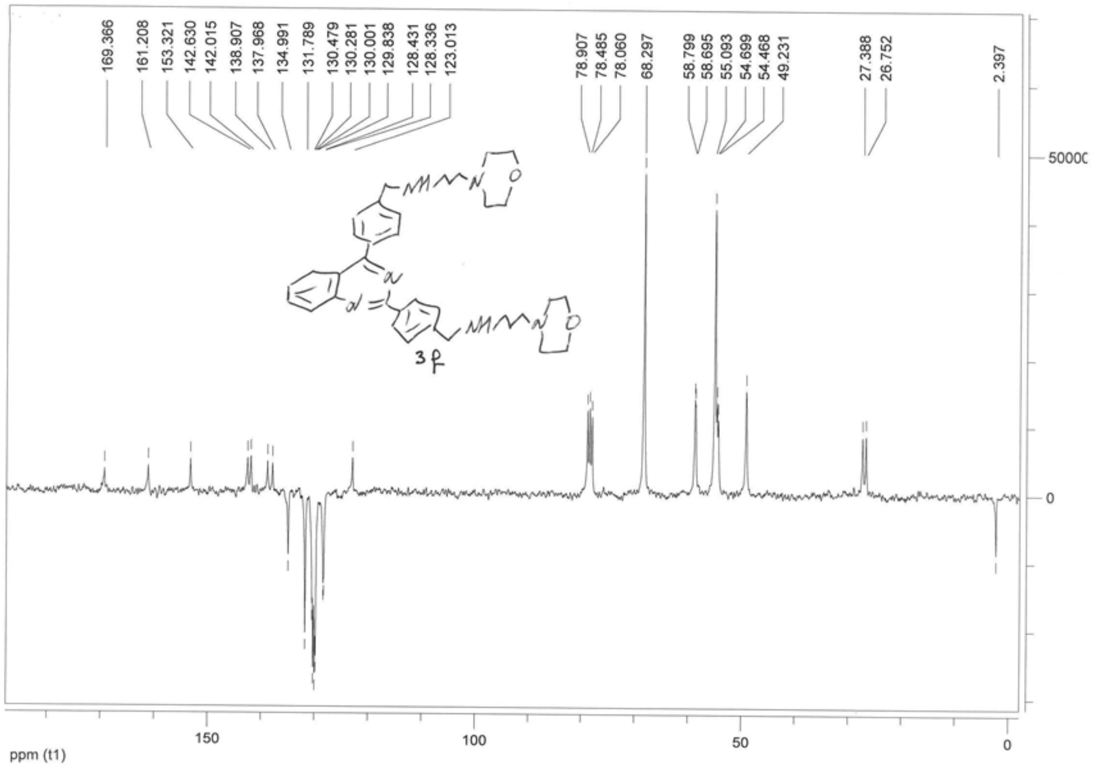


**Fig.S90.** $^{13}$C NMR spectrum of **3f**

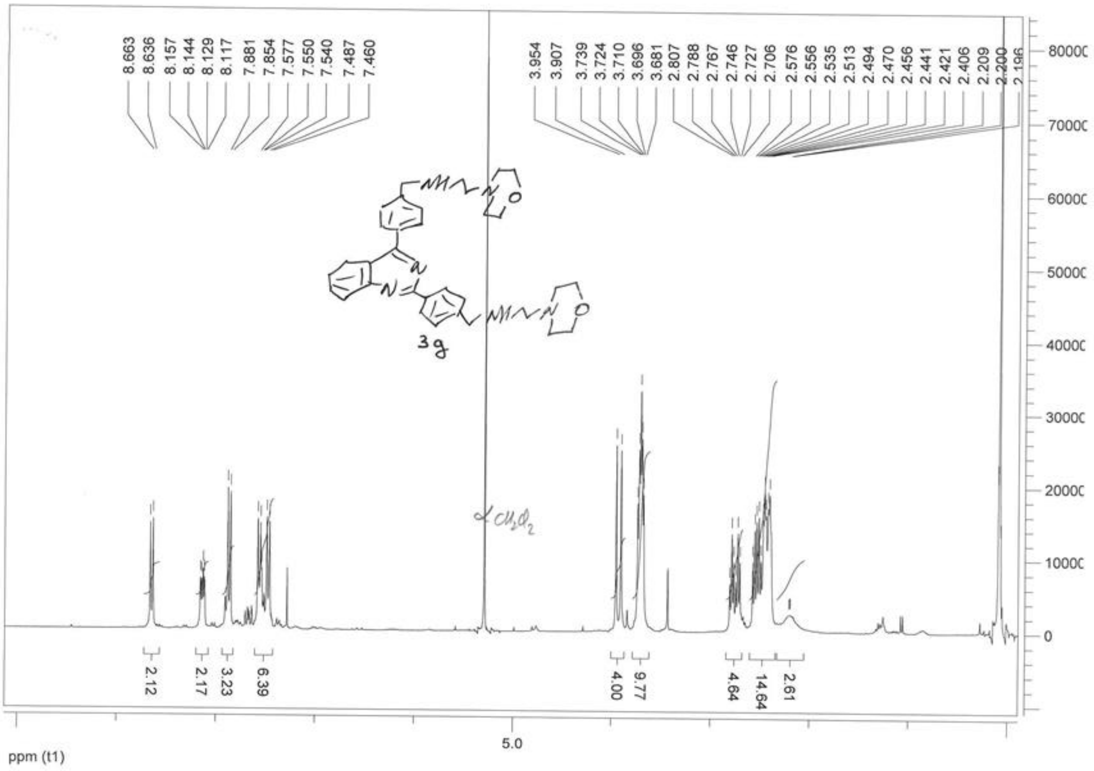

**Fig.S91**. $^{1}$H NMR spectrum of **3g**

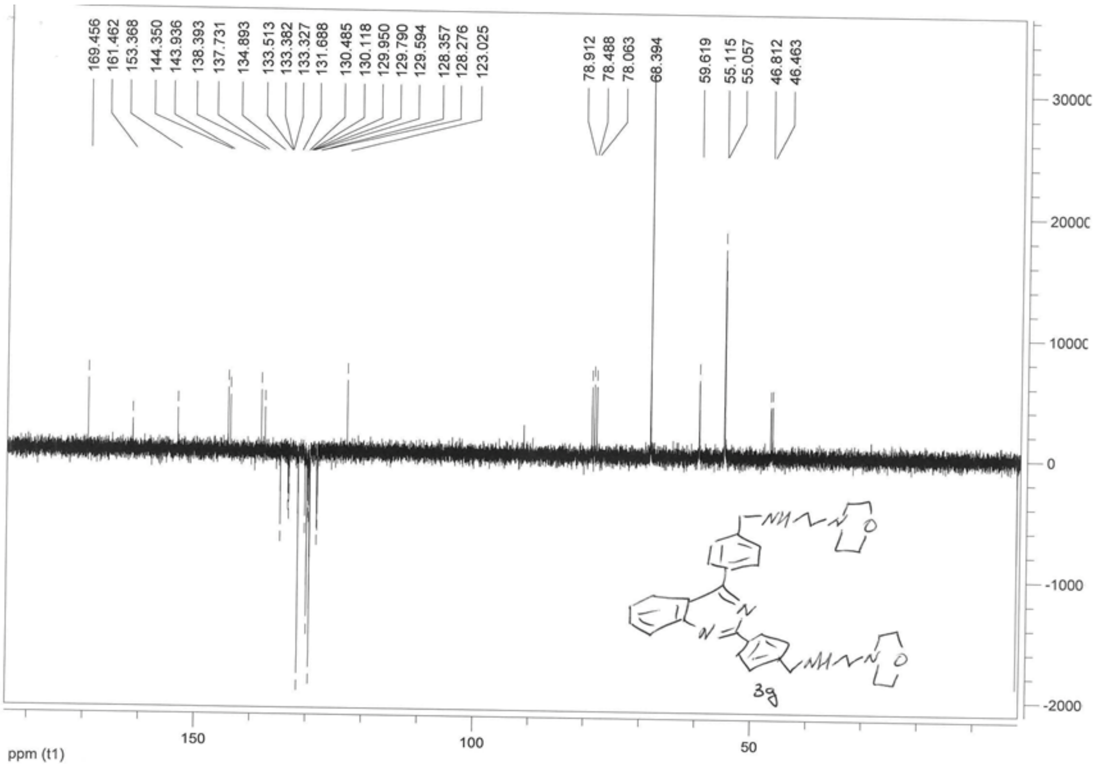

**Fig.S92.** $^{13}$C NMR spectrum of **3g**

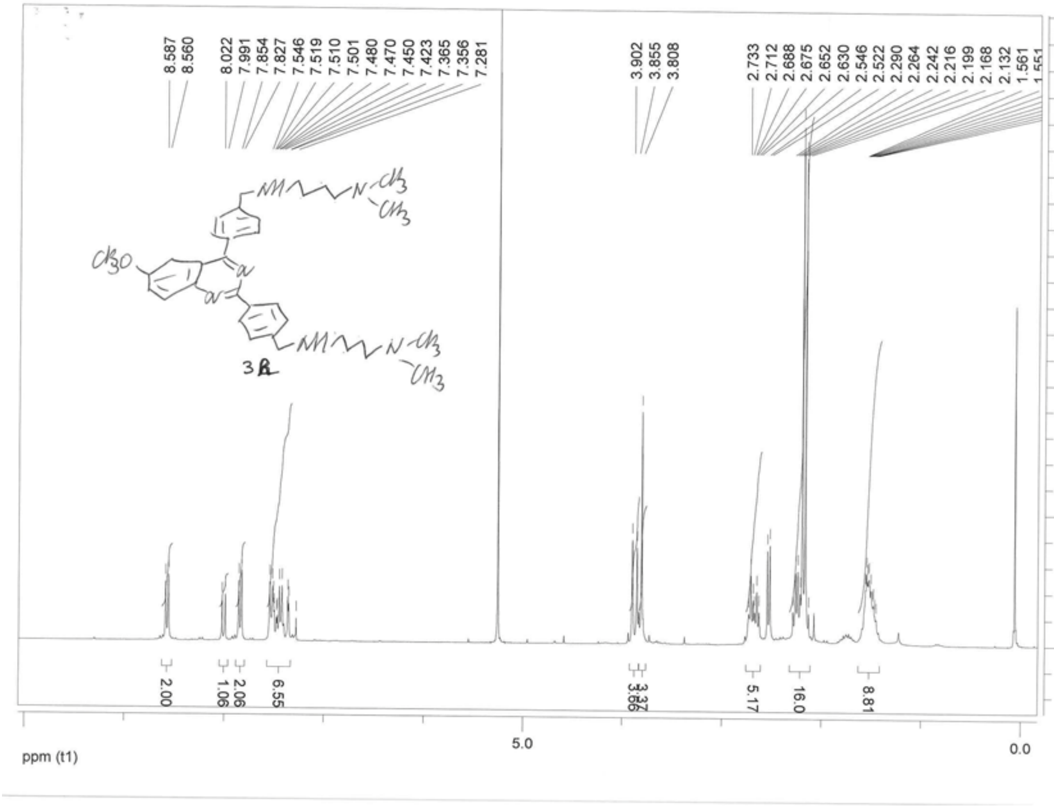

**Fig.S93**. $^{1}$H NMR spectrum of **3h**

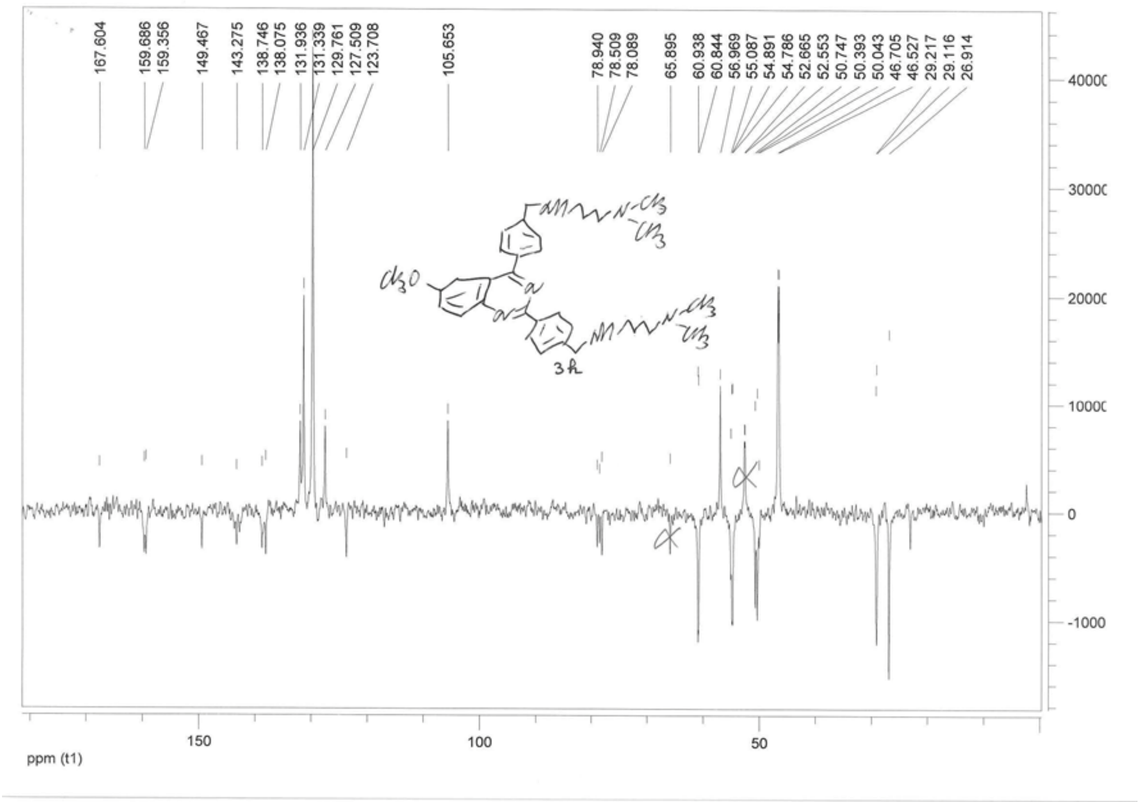

**Fig.S94.** $^{13}$C NMR spectrum of **3h**

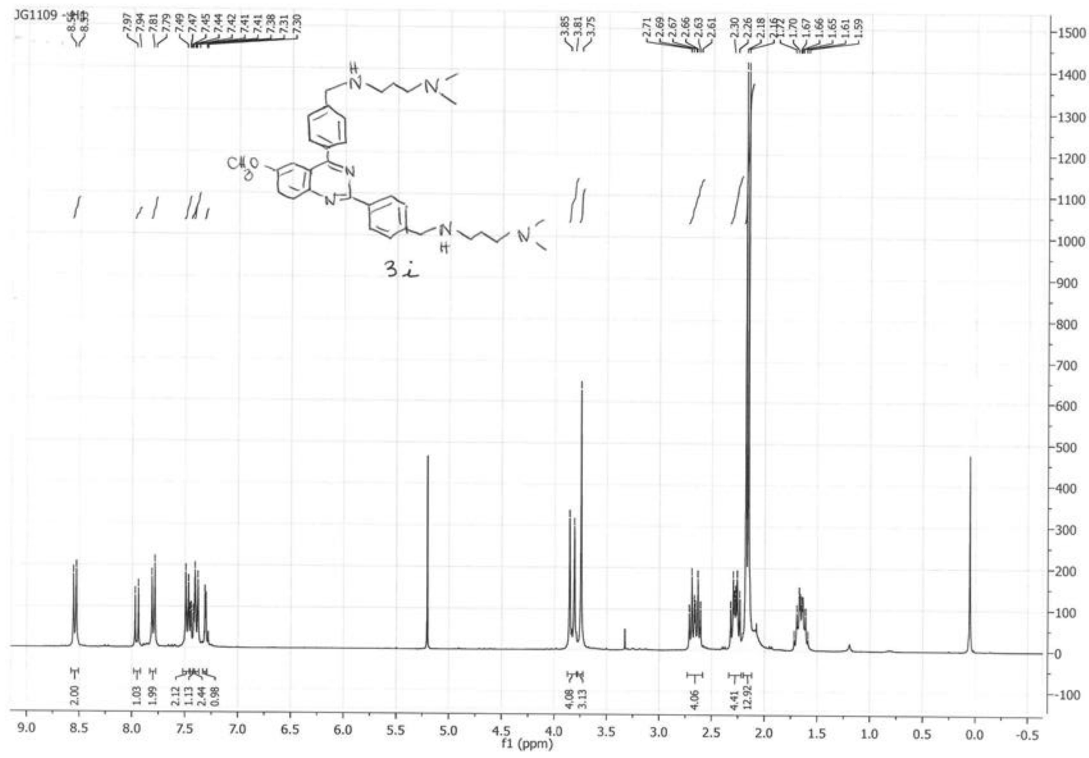

**Fig.S95.** $^{1}$H NMR spectrum of **3i**

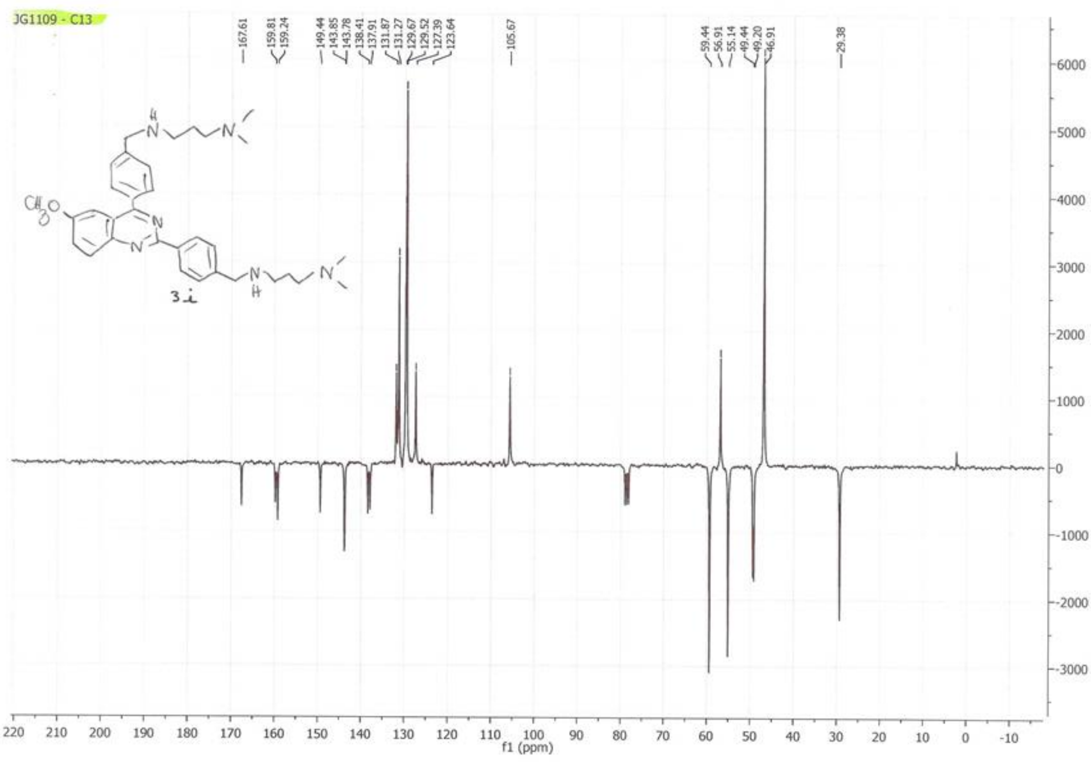

**Fig.S96.** $^{13}$C NMR spectrum of **3i**

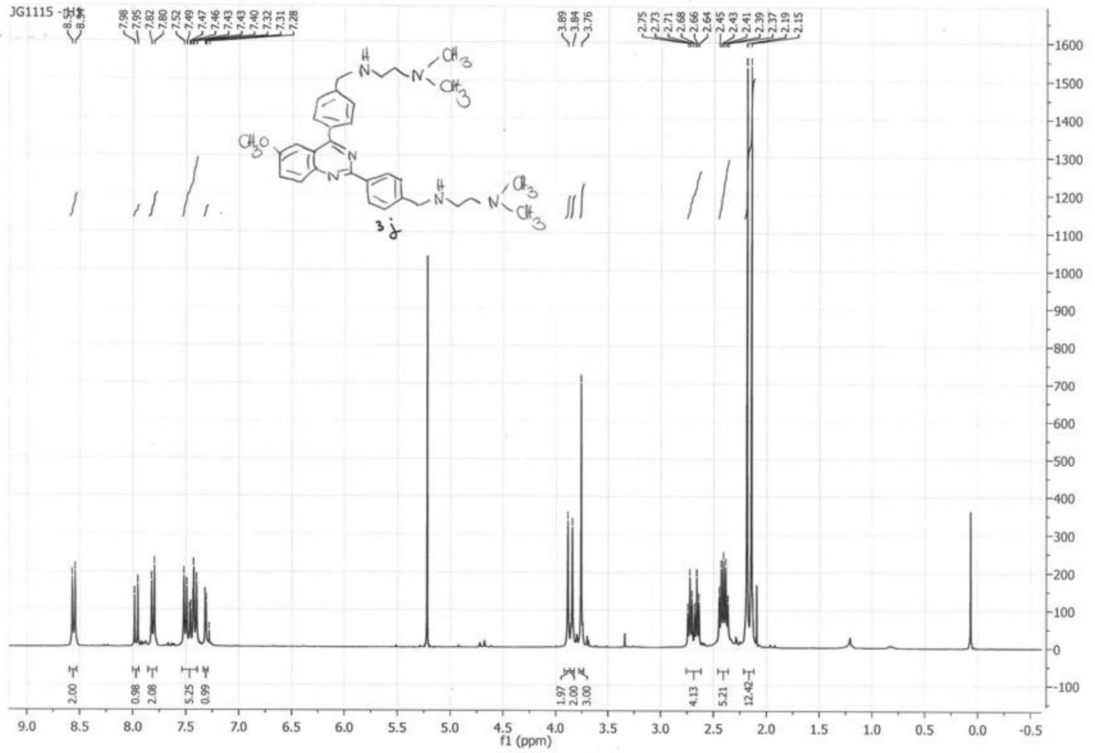

**Fig.S97.** $^{1}$H NMR spectrum of **3j**

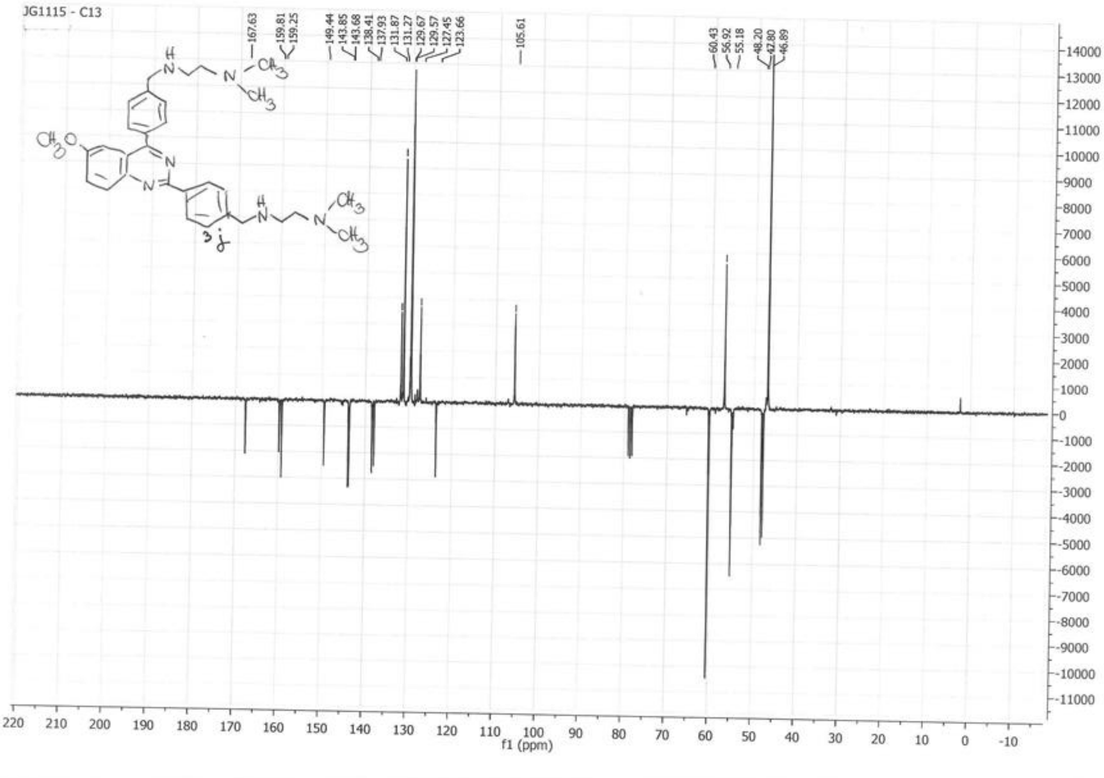

**Fig.S98.** $^{13}$C NMR spectrum of **3j**

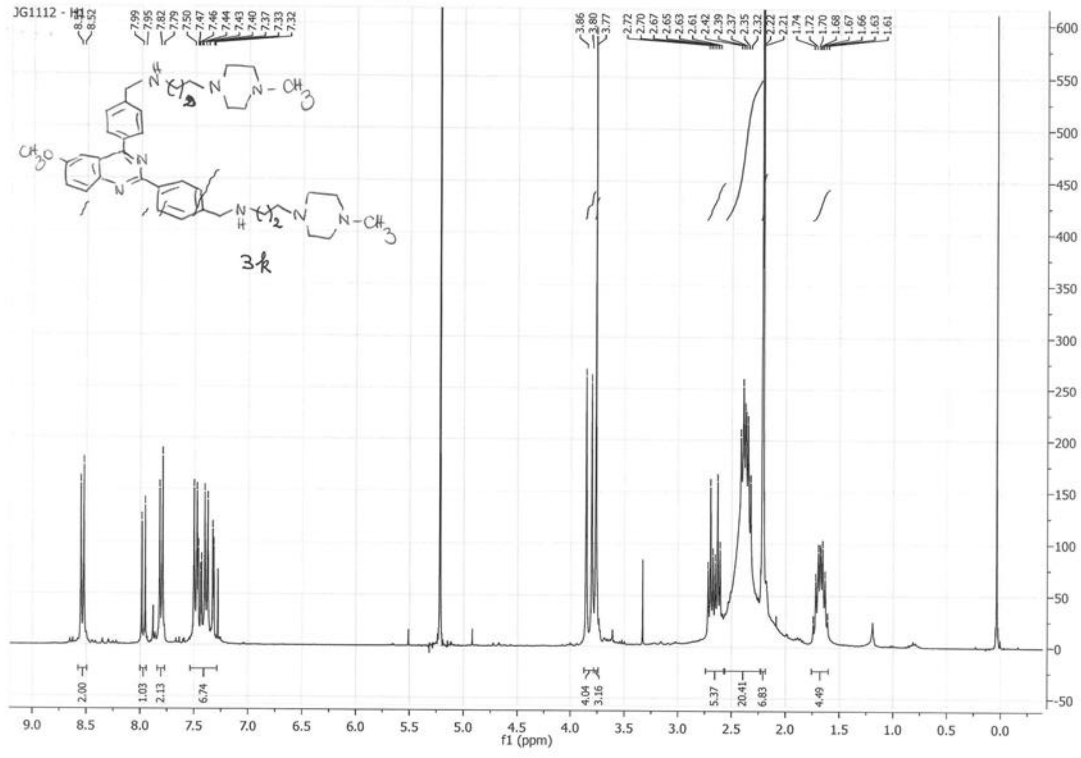

**Fig.S99.** $^{1}$H NMR spectrum of **3k**

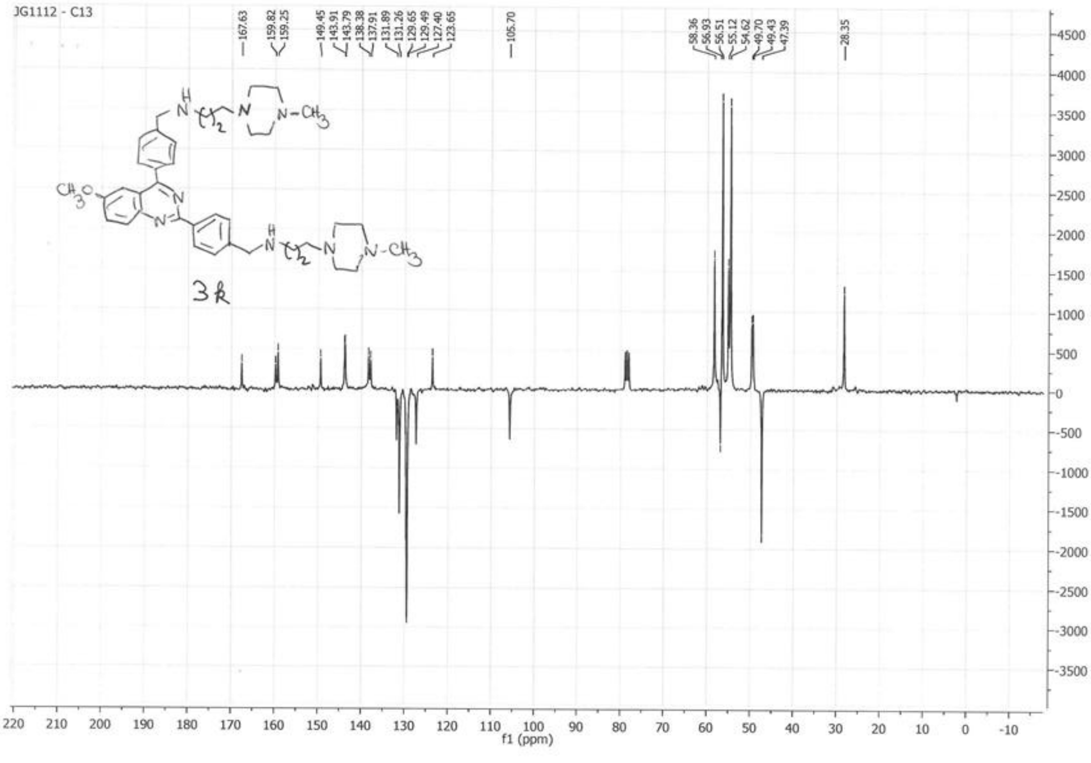

**Fig.S100.** $^{13}$C NMR spectrum of **3k**

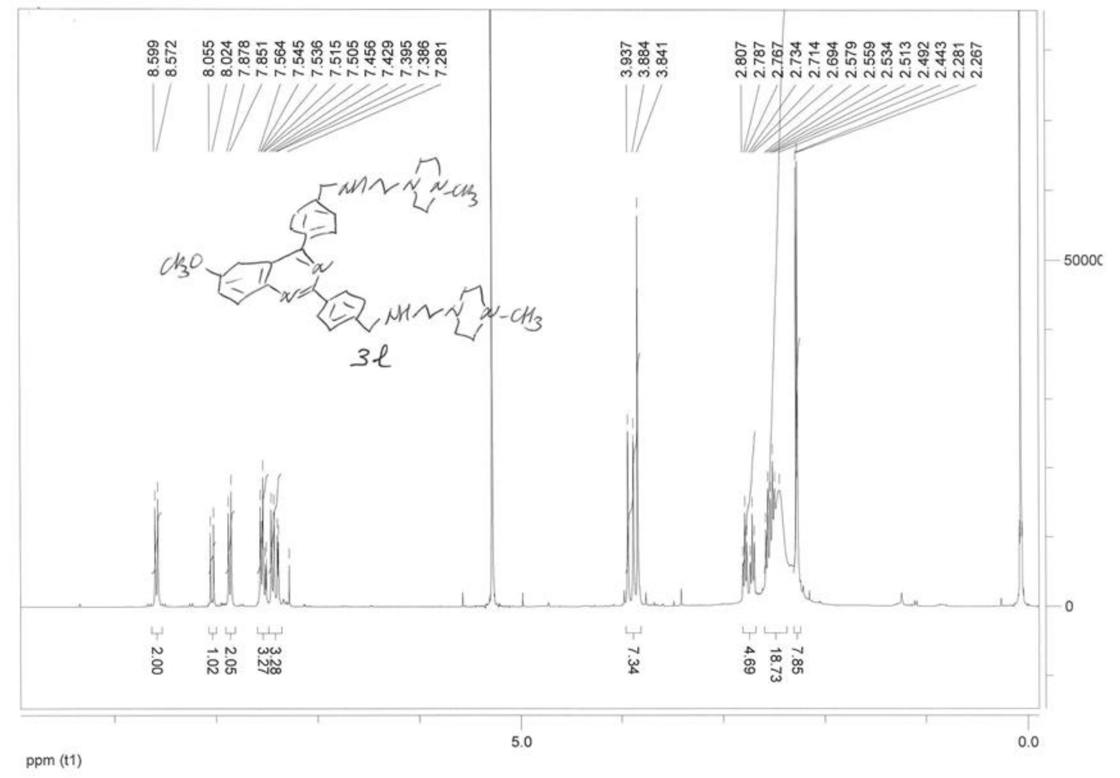


**Fig.S101.** $^{1}$H NMR spectrum of **3l**

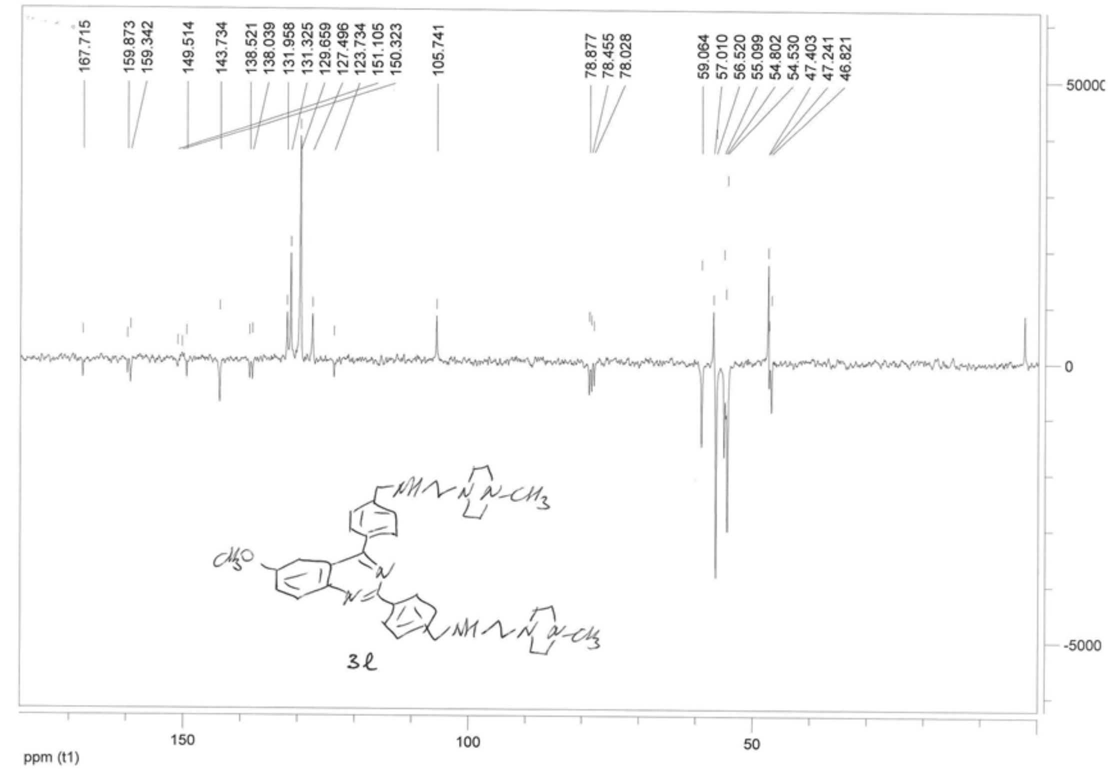


**Fig.S102.** $^{13}$C NMR spectrum of **3l**

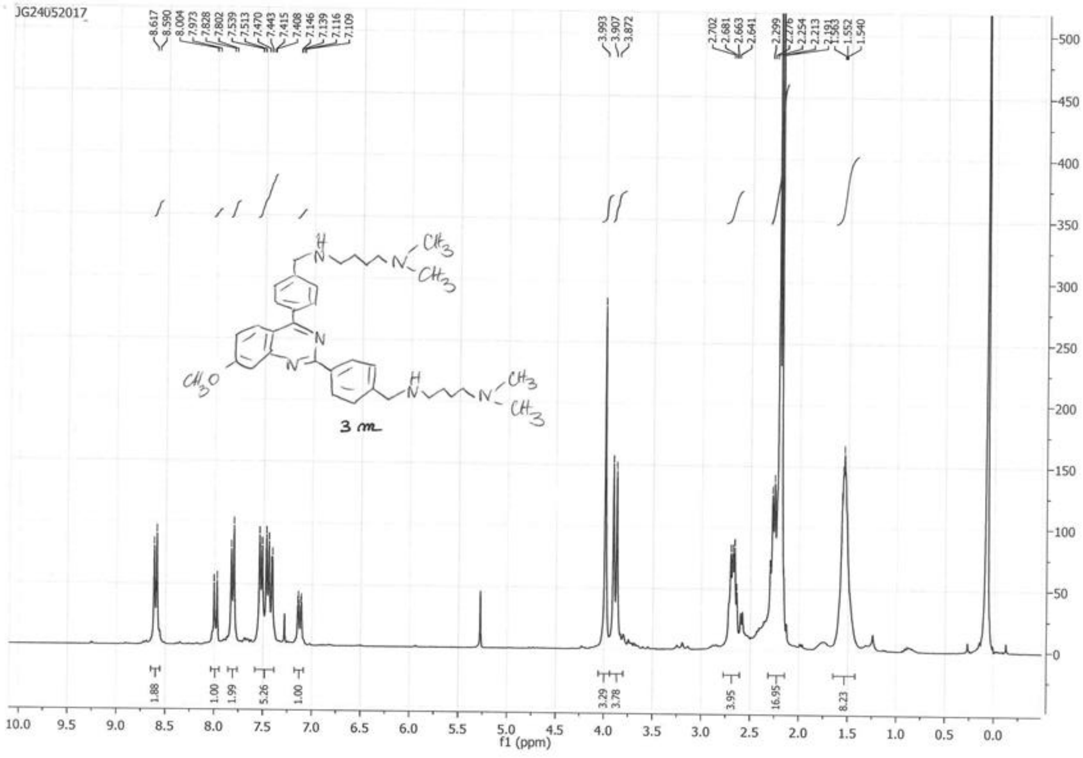

**Fig.S103.** $^{1}$H NMR spectrum of **3m**

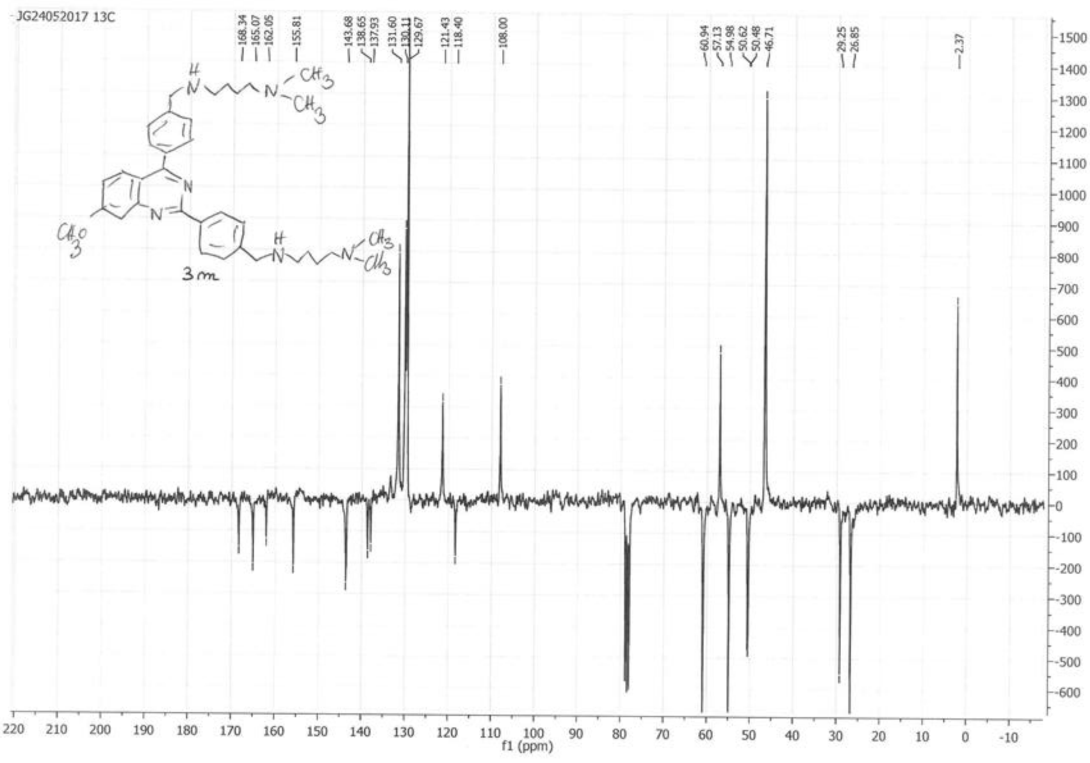

**Fig.S104.** $^{13}$C NMR spectrum of **3m**

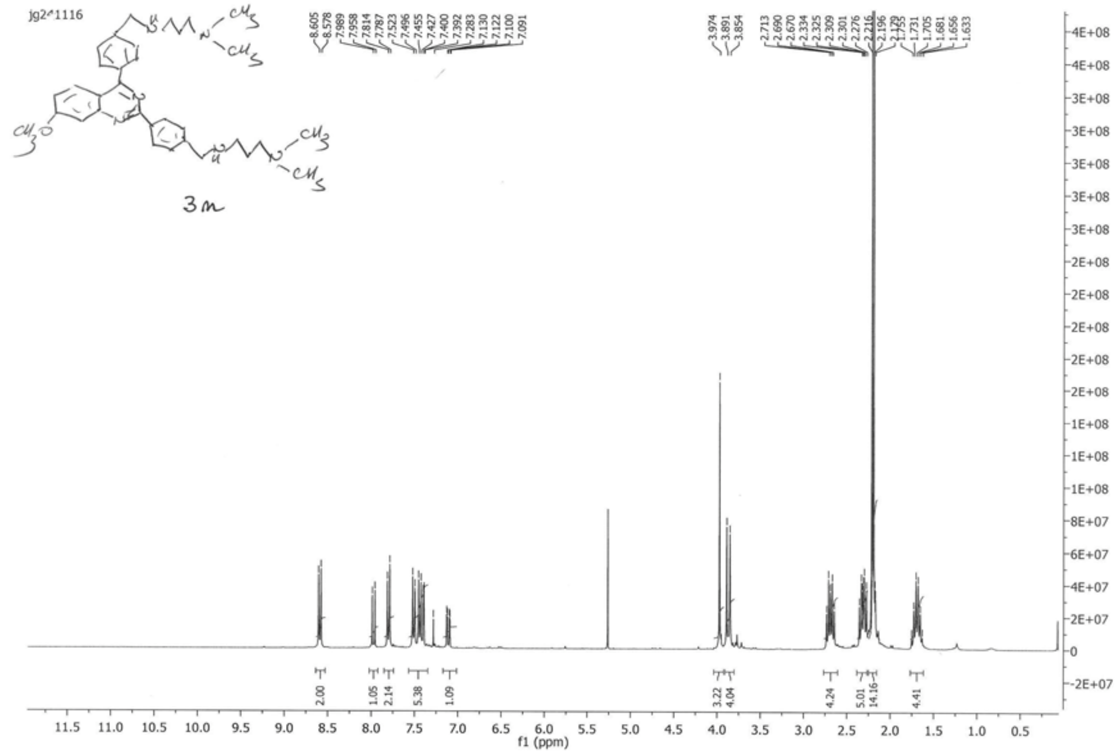

**Fig.S105.** $^{1}$H NMR spectrum of **3n**

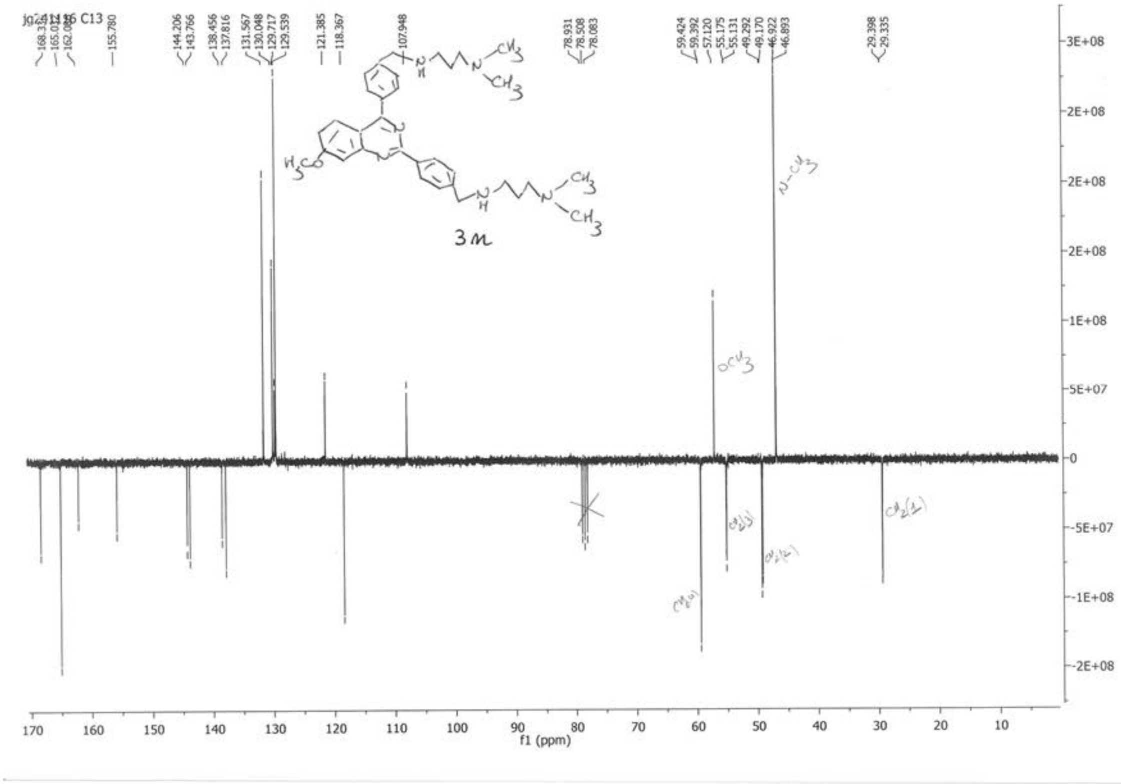

**Fig.S106.** $^{13}$C NMR spectrum of **3n**

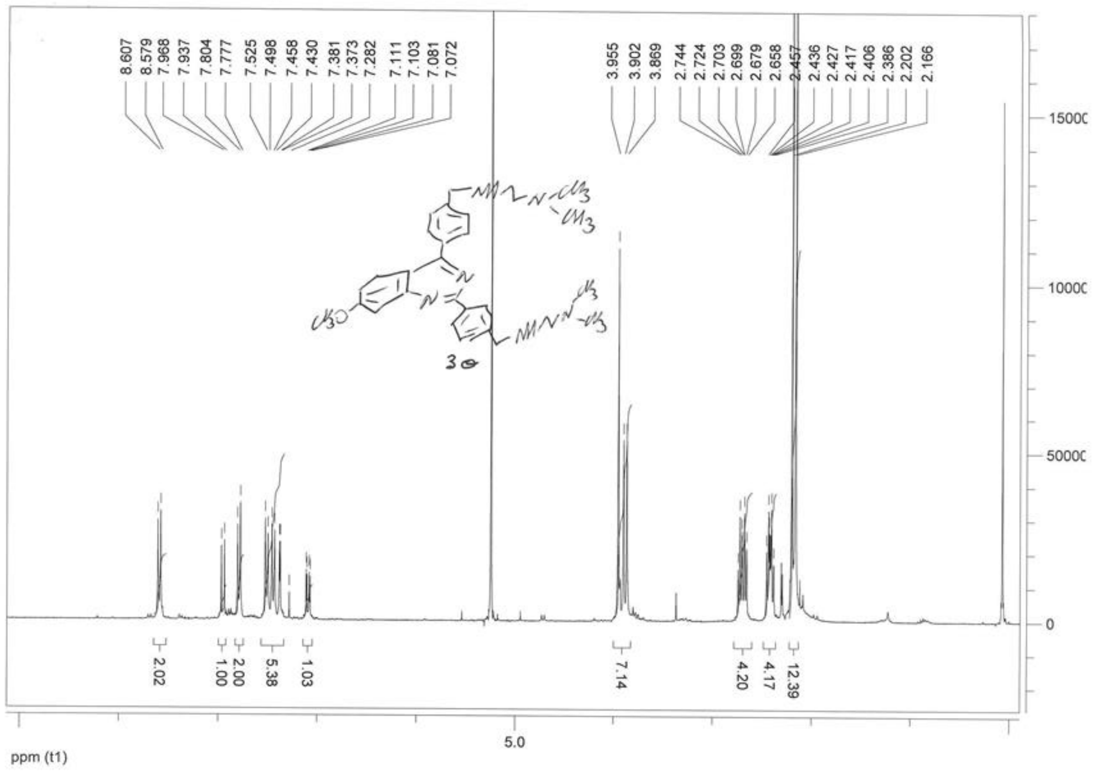

**Fig.S107.** $^{1}$H NMR spectrum of **3o**

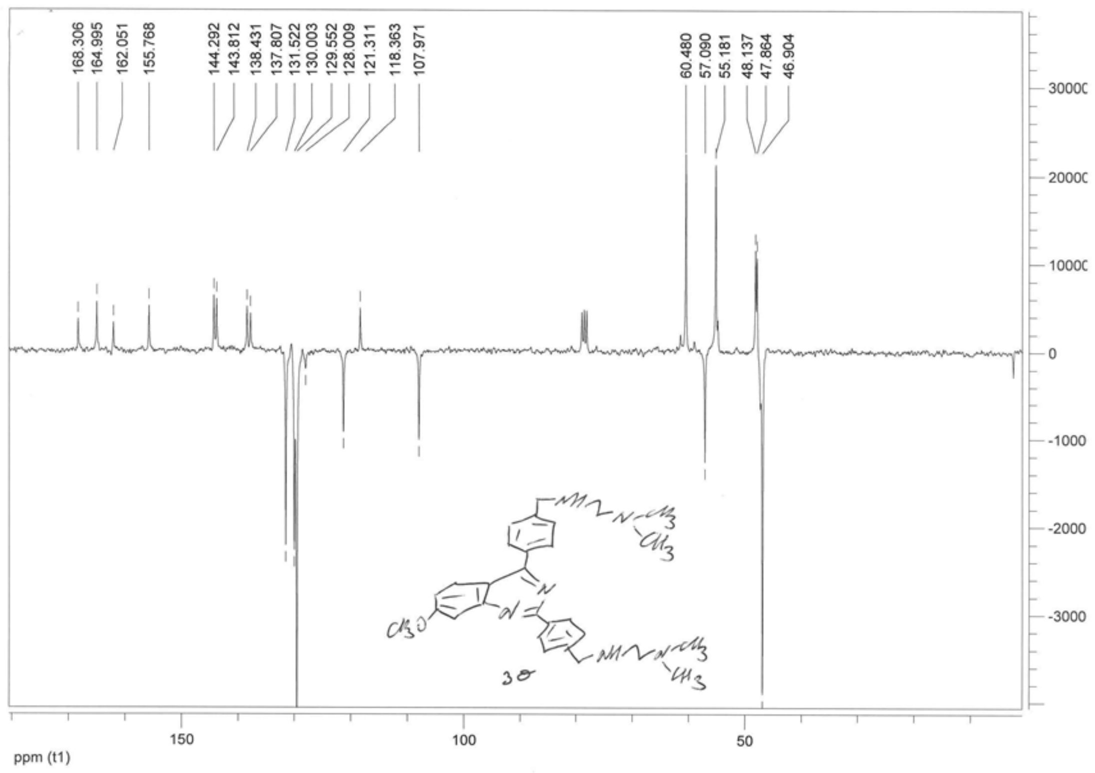

**Fig.S108.** $^{13}$C NMR spectrum of **3o**

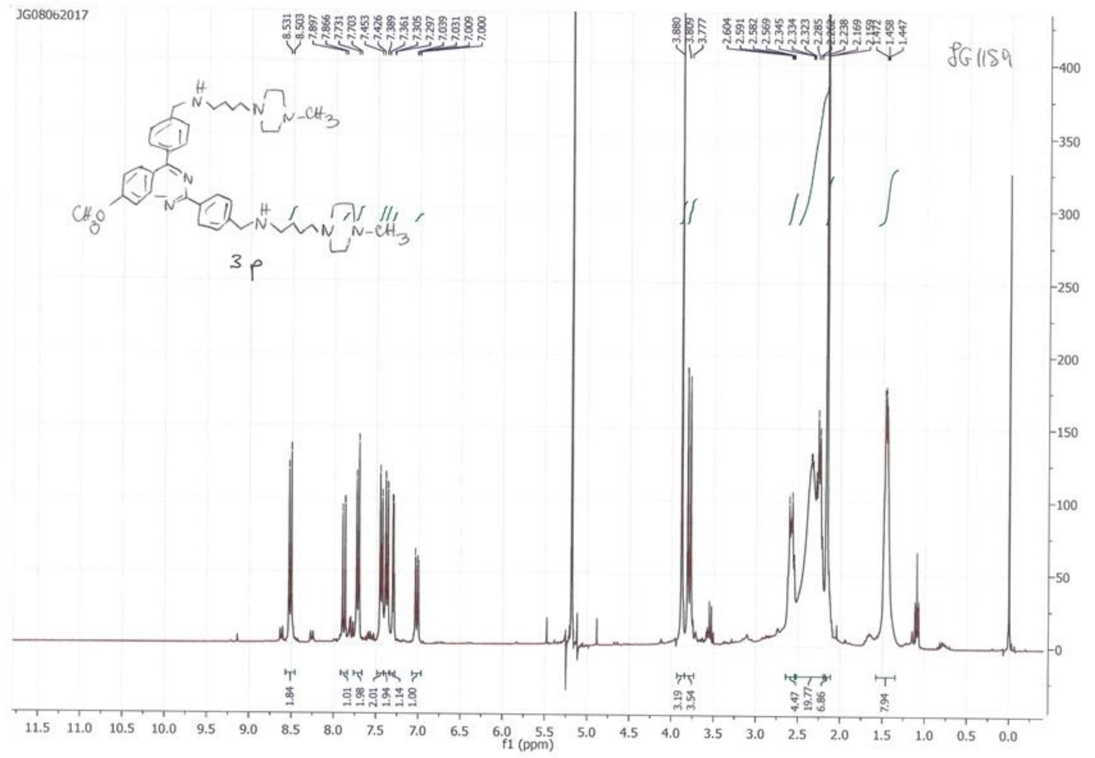

**Fig.S109.** $^{1}$H NMR spectrum of **3p**

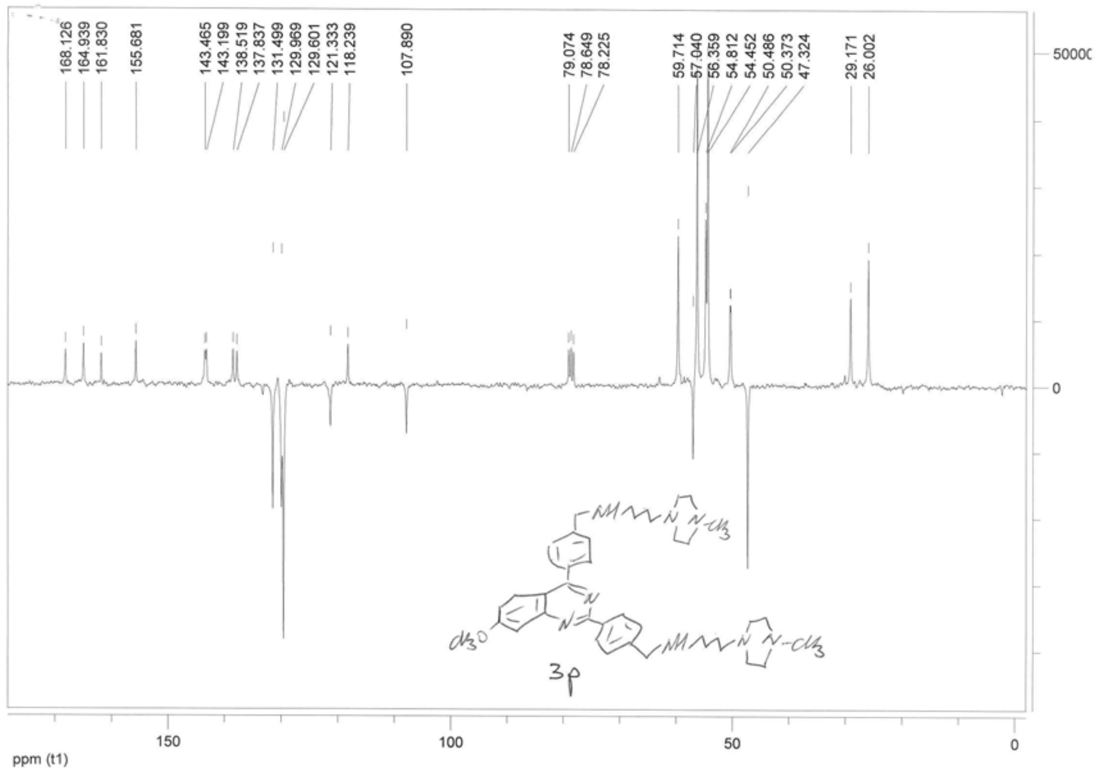

**Fig.S110.** $^{13}$C NMR spectrum of **3p**

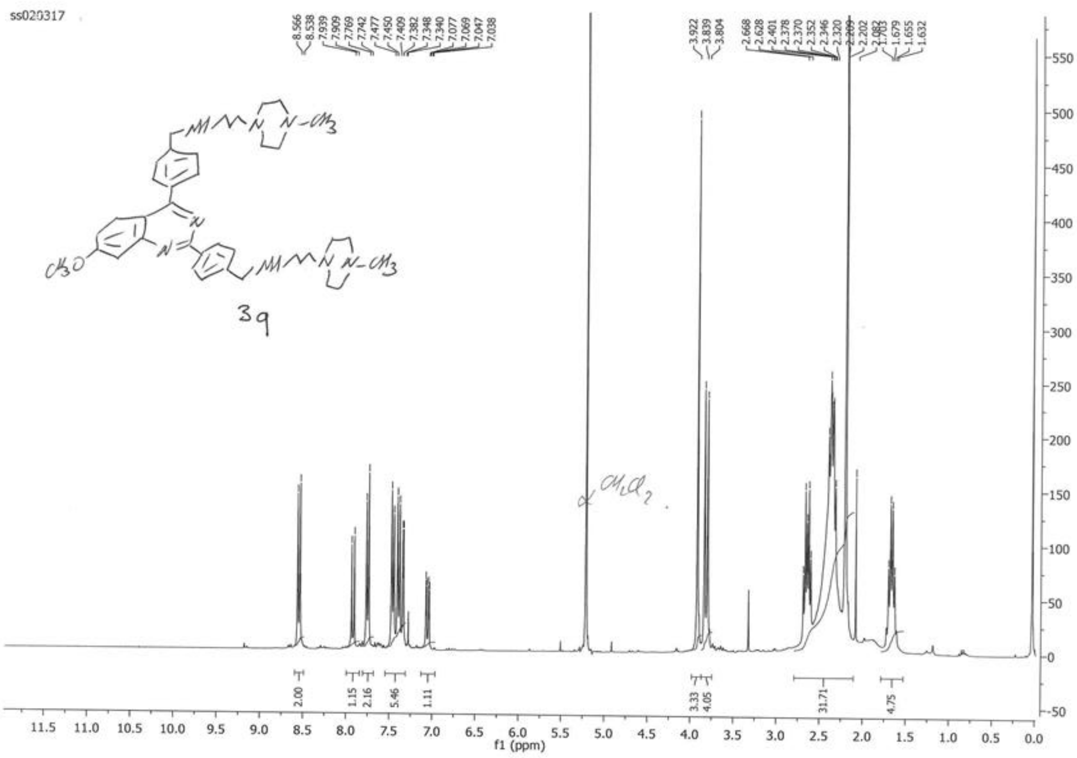

**Fig.S111**. $^{1}$H NMR spectrum of **3q**

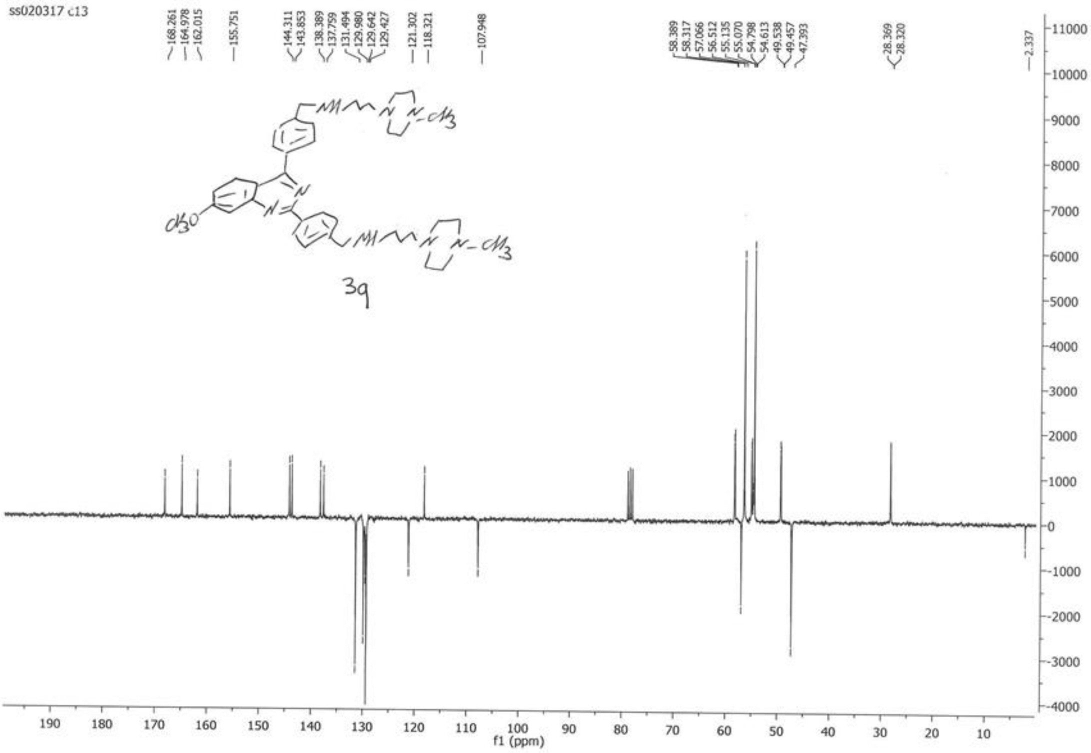

**Fig.S112.** $^{13}$C NMR spectrum of **3q**

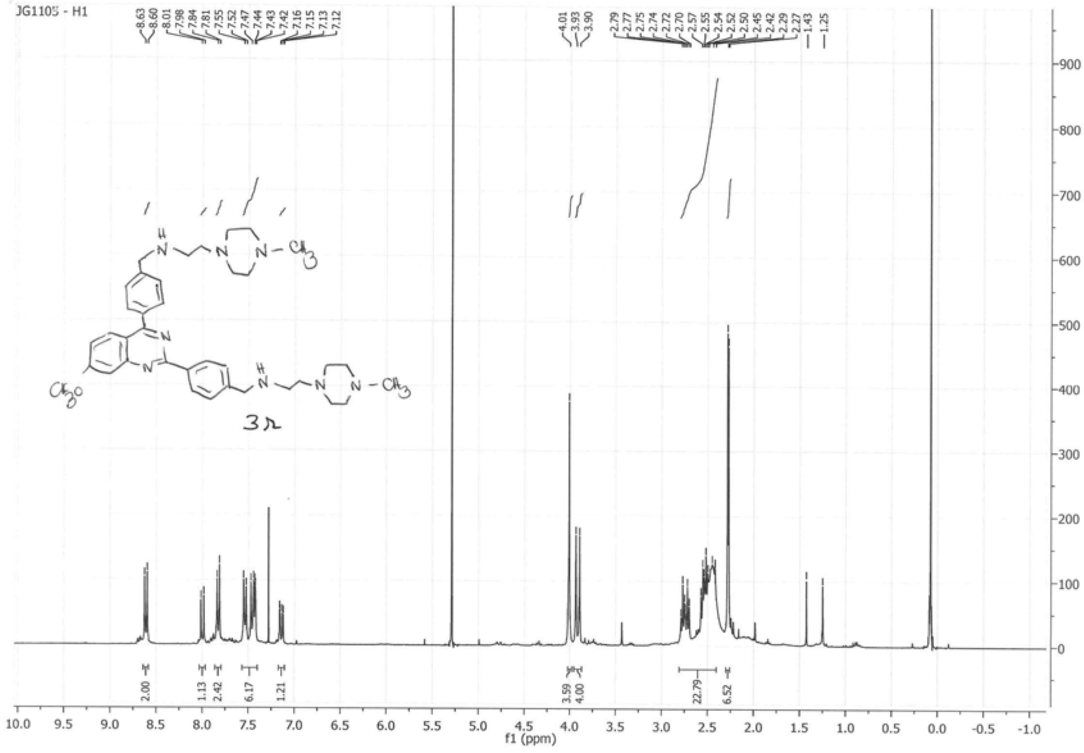

**Fig.S113.** $^{1}$H NMR spectrum of **3r**

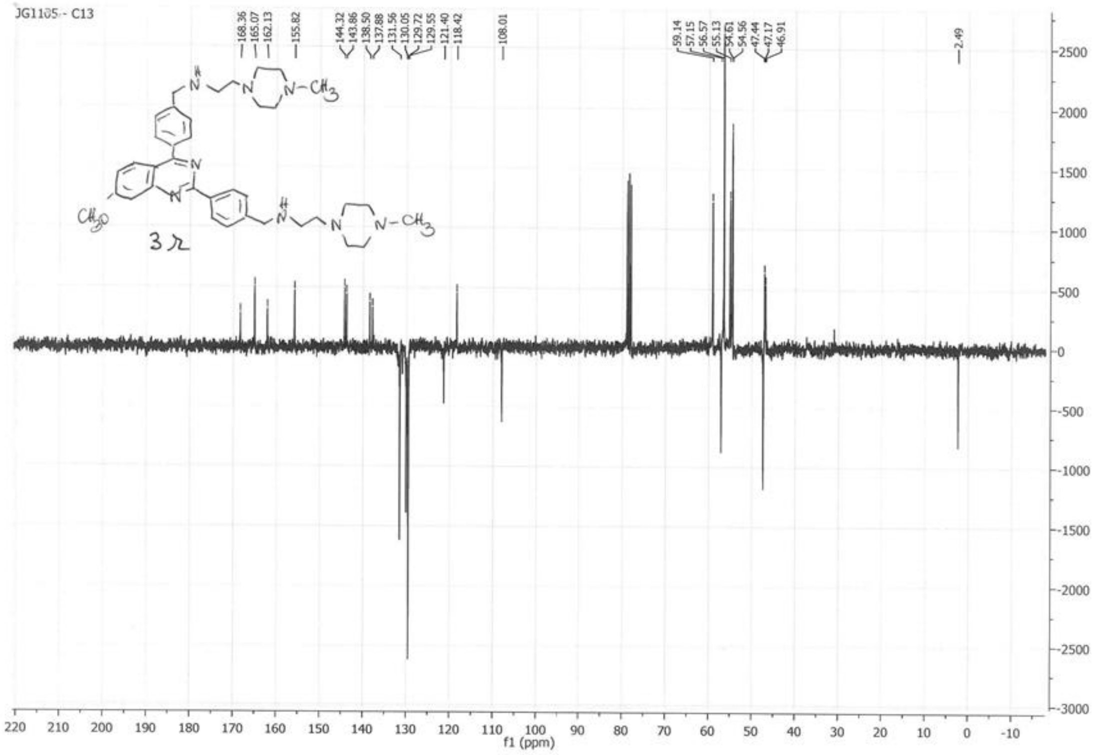

**Fig.S114.** 13C NMR spectrum of **3r**

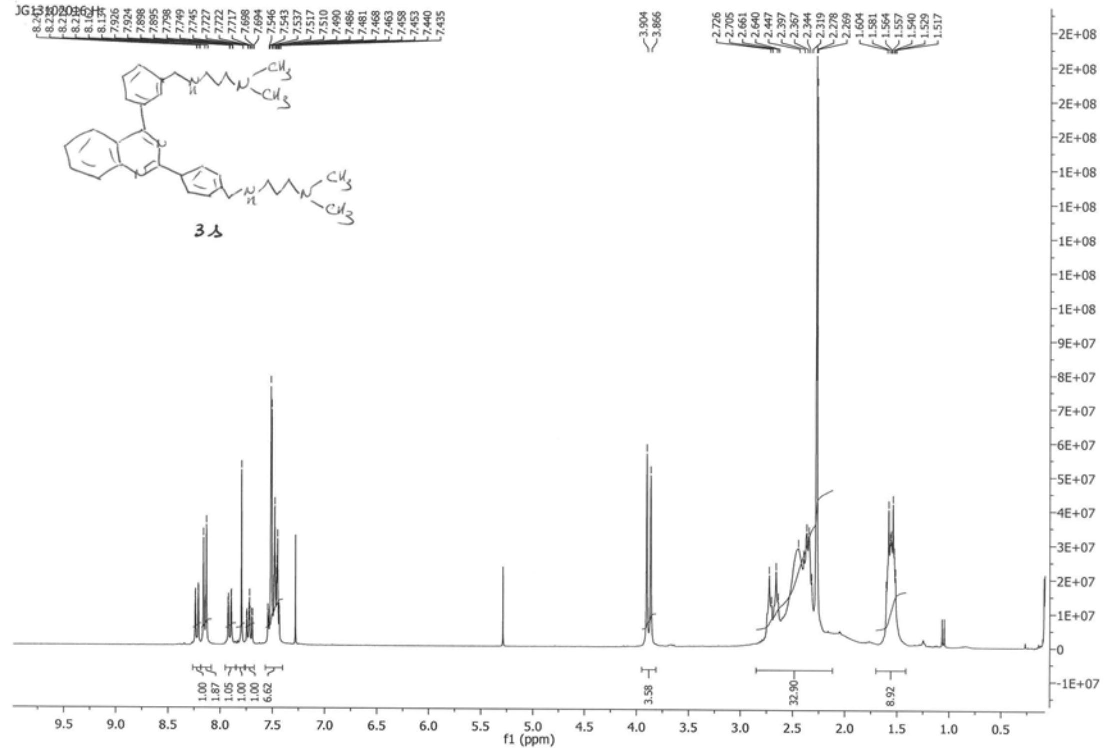

**Fig.S115.** $^{1}$H NMR spectrum of **3s**

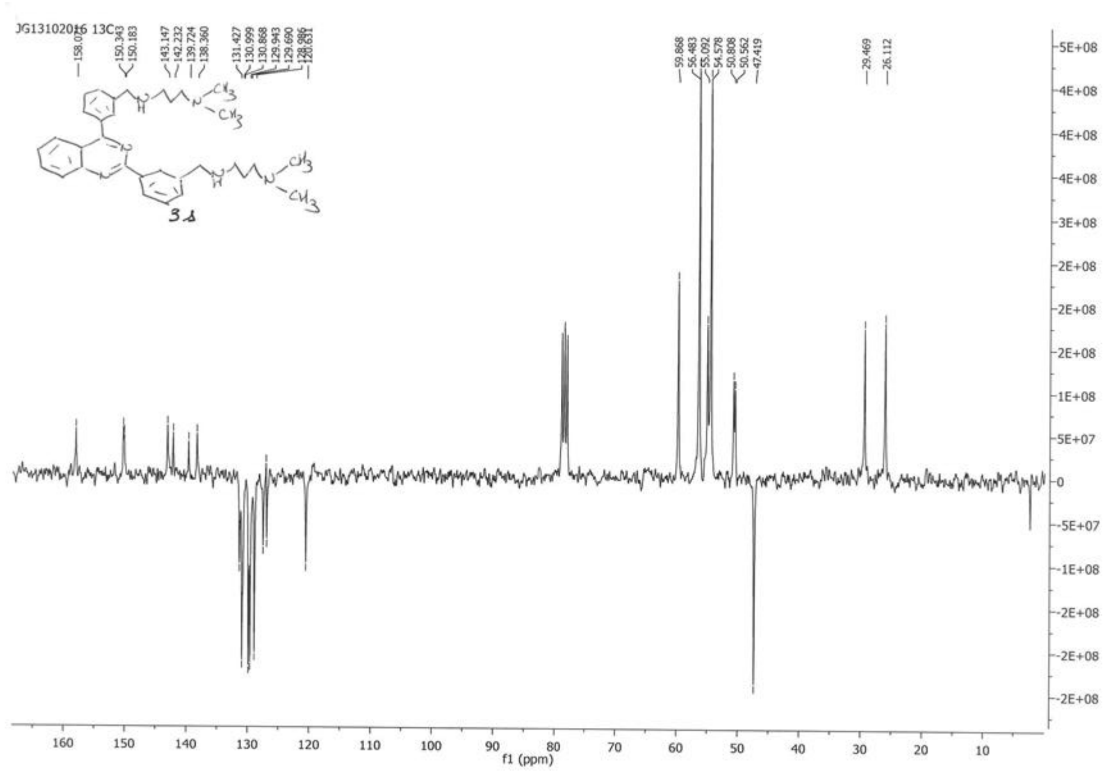

**Fig.S116.** $^{13}$C NMR spectrum of **3s**

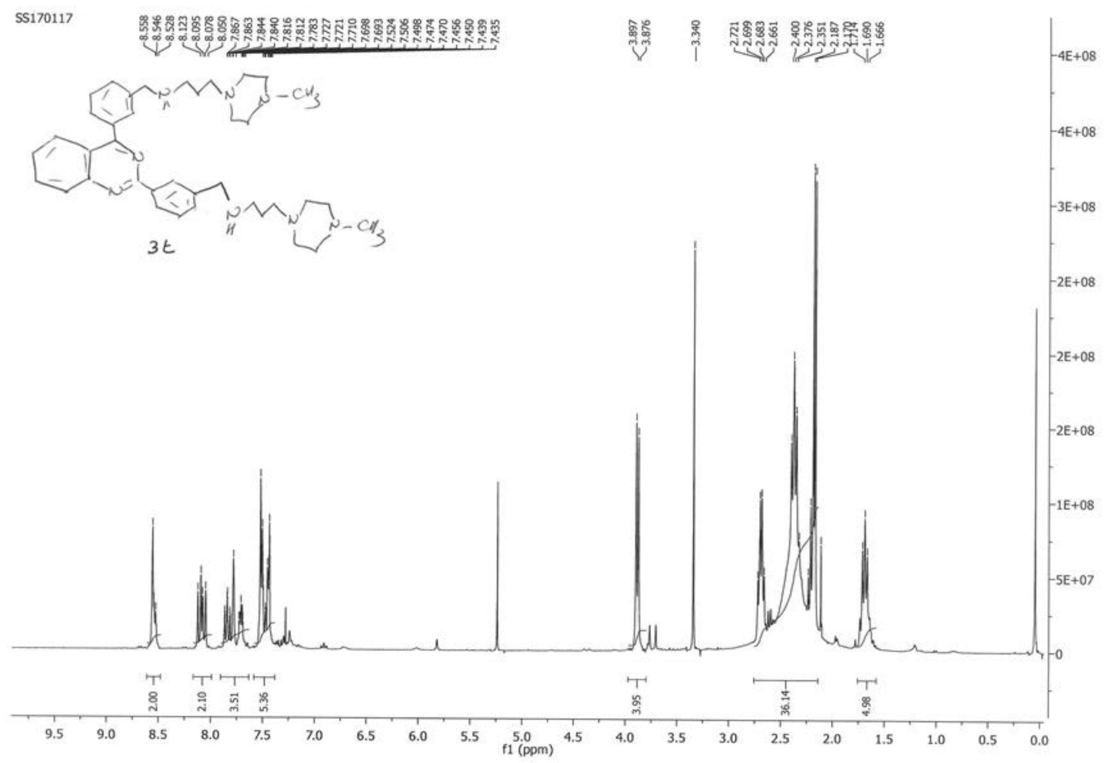

**Fig.S117.** $^{1}$H NMR spectrum of **3t**

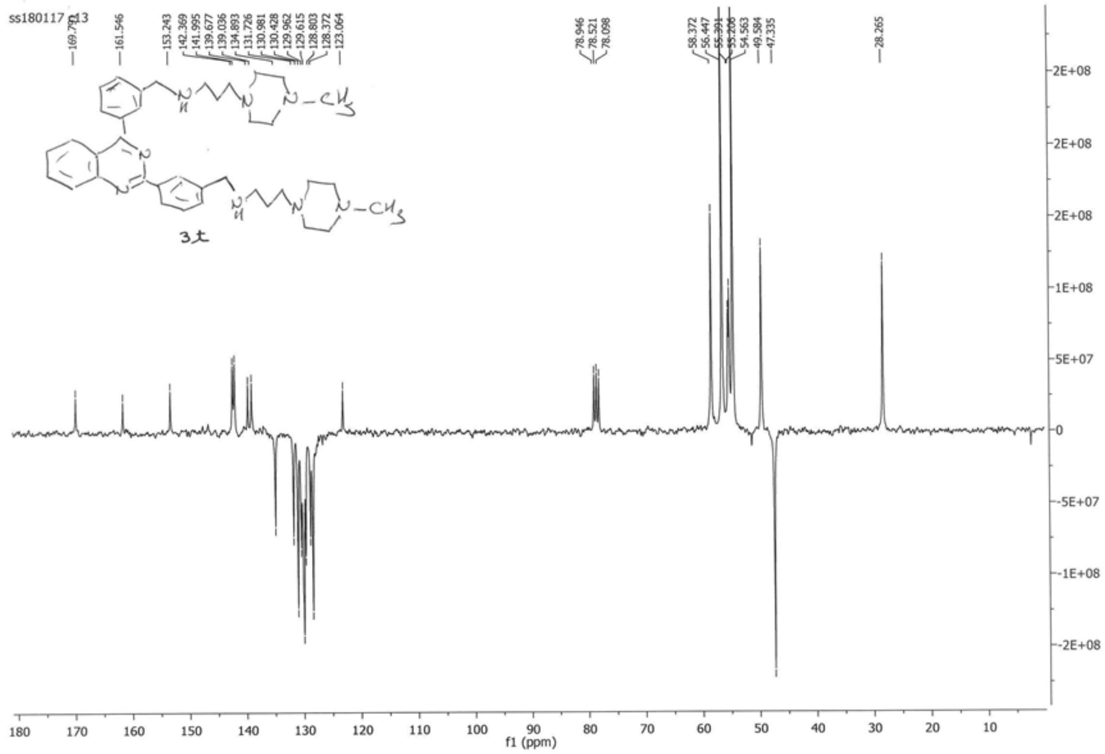

**Fig.S118**. $^{13}$C NMR spectrum of **3t**

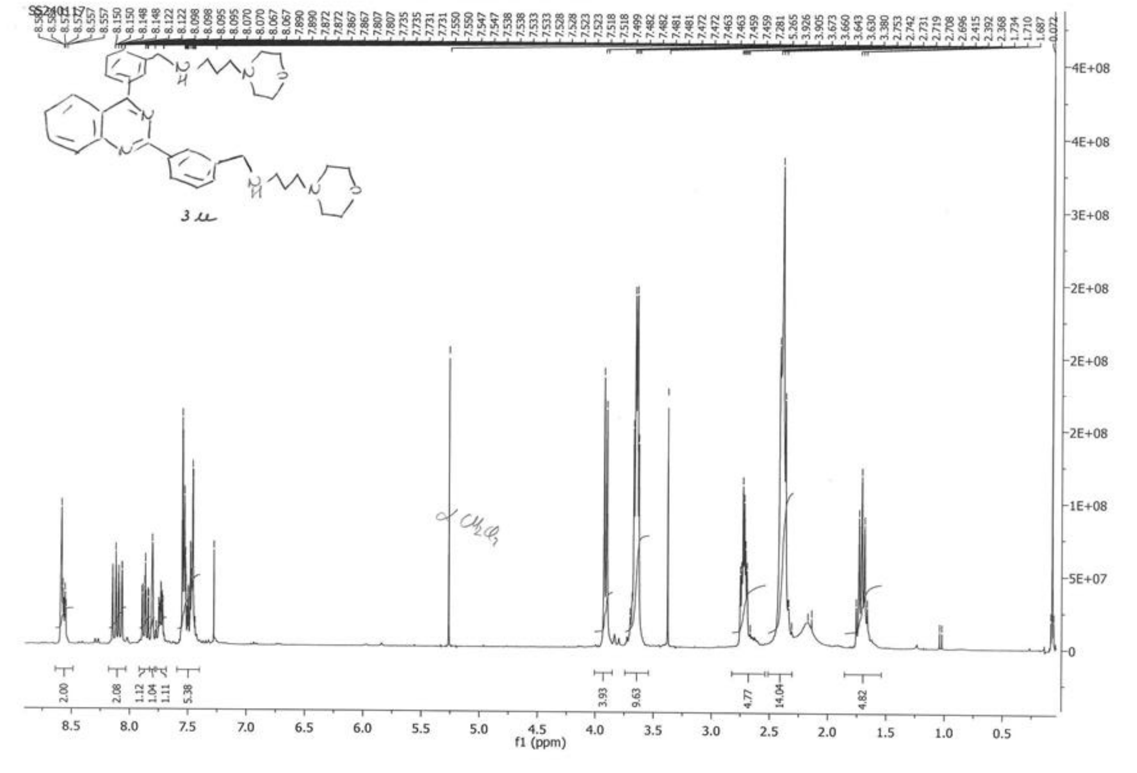

**Fig.S119.** $^{1}H$ NMR spectrum of **3u**

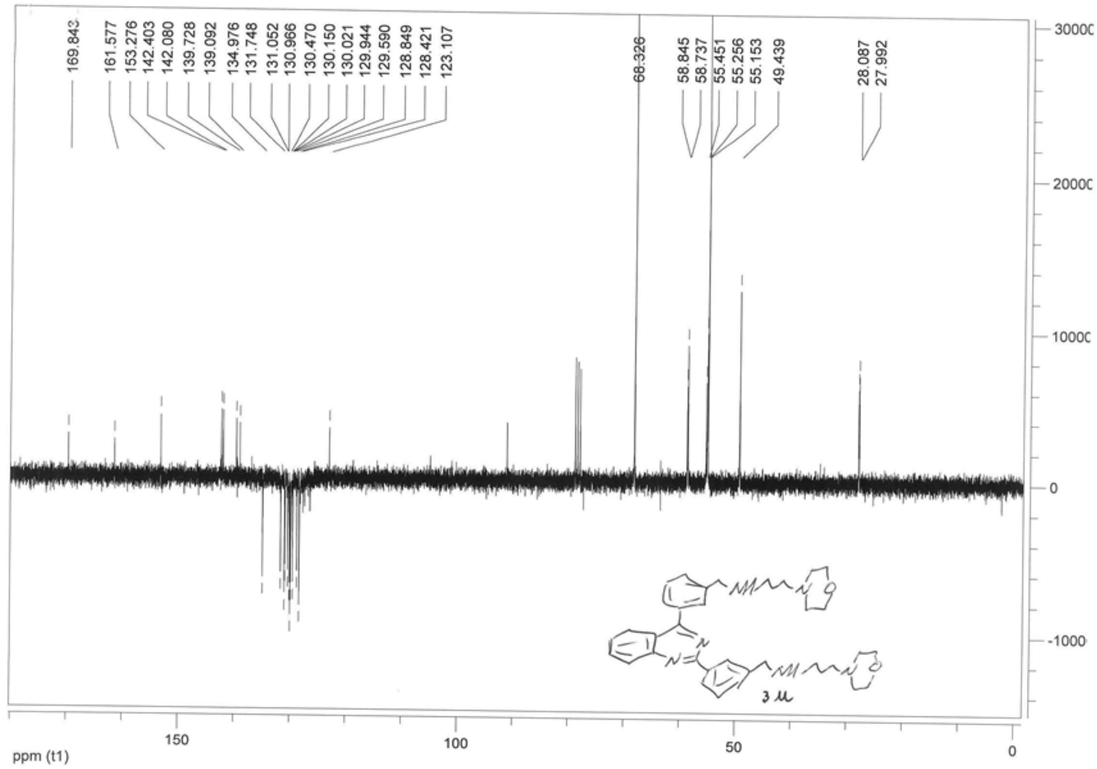

**Fig.S120.** $^{13}C$ NMR spectrum of **3u**

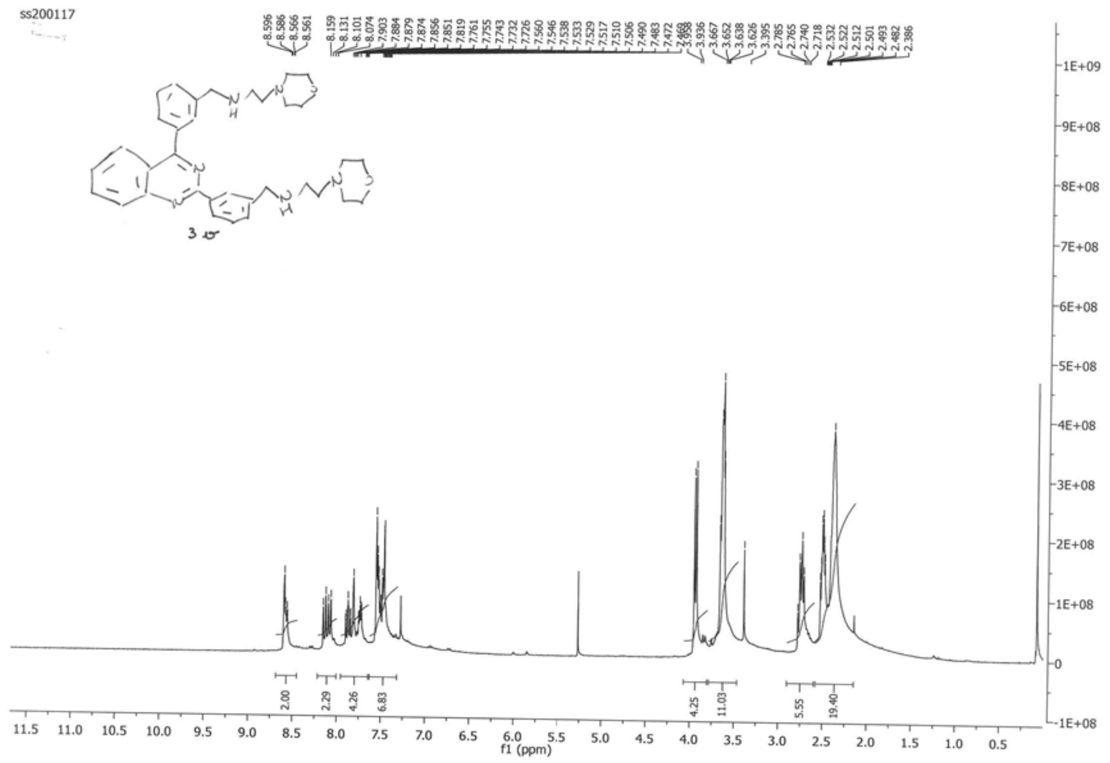

**Fig.S121.** $^{1}$H NMR spectrum of **3v**

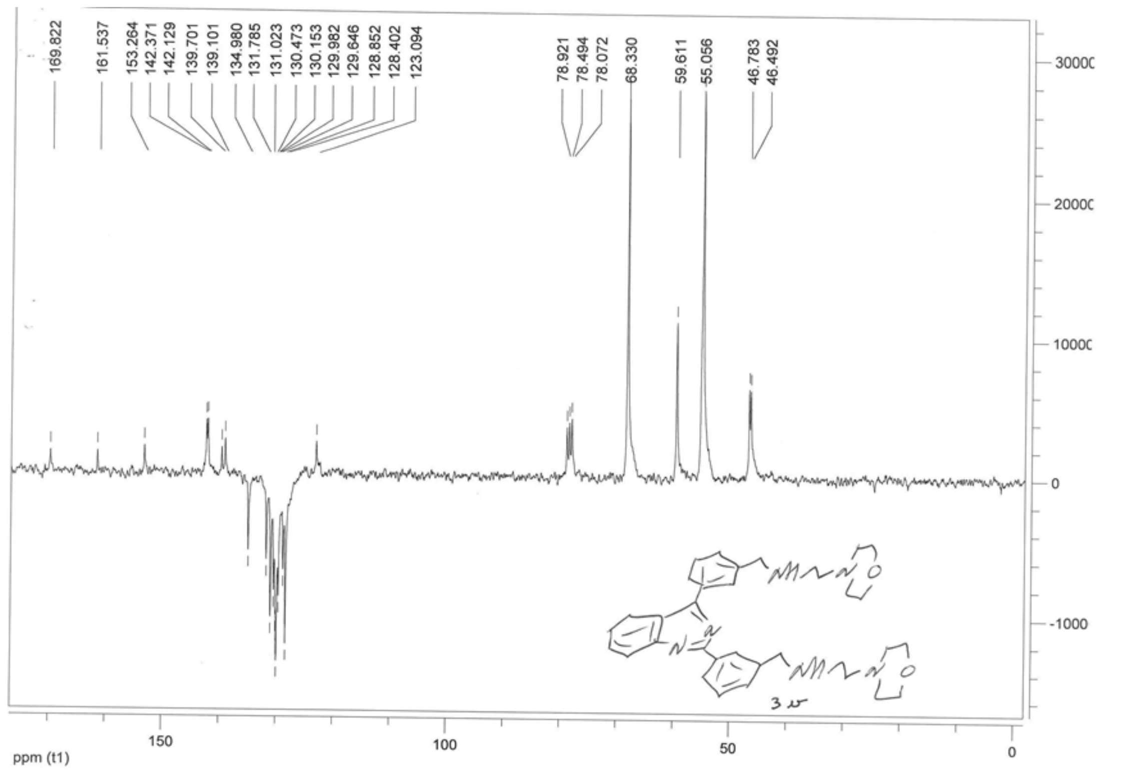

**Fig.S122.** $^{13}$C NMR spectrum of **3v**
